# Cationic Amphiphiles with Five-Membered Heterocyclic Linkers: Synthesis, Self-Assembly, and DNA Complexation Properties

**DOI:** 10.3390/ma19132744

**Published:** 2026-06-26

**Authors:** Anda Sipola, Ksenija Korotkaja, Karlis Pajuste, Aiva Plotniece, Arkadij Sobolev

**Affiliations:** 1 Latvian Institute of Organic Synthesis, Aizkraukles 21, LV-1006 Riga, Latvia; kpajuste@osi.lv (K.P.); aiva@osi.lv (A.P.); 2 Faculty of Medicine and Life Sciences, University of Latvia, Jelgavas 1, LV-1004 Riga, Latvia; 3 Latvian Biomedical Research and Study Centre, Ratsupites 1 k-1, LV-1067 Riga, Latvia; ksenija.korotkaja@biomed.lu.lv; 4 Department of Applied Pharmacy, Faculty of Pharmacy, Riga Stradiņš University, Konsula 21, LV-1007 Riga, Latvia

**Keywords:** cationic amphiphilic lipids, heterocyclic linkers, self-assembly, liposomes, lipoplexes, encapsulation efficiency

## Abstract

Lipid-based nanoparticles are widely explored as non-viral vectors for nucleic acid delivery, where the molecular structure of cationic lipids strongly determines their performance. Five-membered heterocyclic linkers were explored as a new structural motif in cationic amphiphilic lipids for the development of promising gene delivery candidates. Novel lipids incorporating pyrrole, furan, and thiophene linkers were synthesized alongside structurally related aliphatic analogues, enabling systematic evaluation of how linker type influences physicochemical behavior and self-assembly properties. Self-assembly behavior in aqueous media was characterized by dynamic light scattering, and *p*DNA encapsulation efficiency was measured using the Quant-iT Pico-Green method. The resulting liposomes exhibited hydrodynamic diameters ranging from 92 to 1317 nm, while corresponding lipoplexes ranged from 302 to 1159 nm. Amphiphiles containing heterocyclic linkers demonstrated high *p*DNA encapsulation (>80% at optimal N/P ratios), whereas aliphatic analogues showed significantly reduced performance. These results demonstrate that linker structure strongly influences both self-assembly and nucleic acid binding properties. By evaluating structure–activity relationships, five-membered heterocycles are proposed as promising structural elements for the rational development of lipid-based gene delivery candidates.

## 1. Introduction

**Figure 1:**
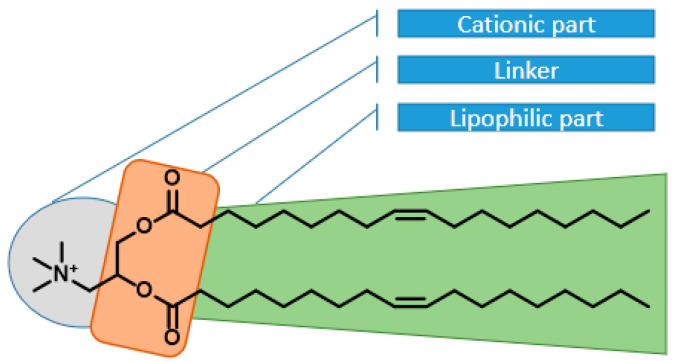
Schematic representation of DOTAP, a commonly used cationic lipid for gene delivery.

Cationic amphiphilic lipids are crucial components of non-viral delivery vehicles for nucleic acids, forming complexes with their anionic counterparts. These carriers spontaneously self-assemble with anionic nucleic acids via electrostatic interactions, forming nano- or microparticles known as lipoplexes and polyplexes, respectively. These complexes protect nucleic acids from nuclease-mediated degradation and facilitate intracellular delivery of the genetic cargo [1]. Lipid-like compounds that form liposomes typically consist of three or four main structural elements. These include: (1) a hydrophilic headgroup, usually cationic, that interacts with the anionic part of the genetic material; (2) a linker connecting the hydrophilic and hydrophobic parts; (3) an optional backbone domain/spacer that increases the distance between the hydrophilic and hydrophobic parts; and (4) a hydrophobic domain, which plays a role in phase transition [2]. A schematic representation of a lipid-like cationic molecule, illustrated based on a 1,2-dioleoyl-3-trimethylammonium-propane (DOTAP) derivative, is shown in Figure 1.

**Figure 2:**
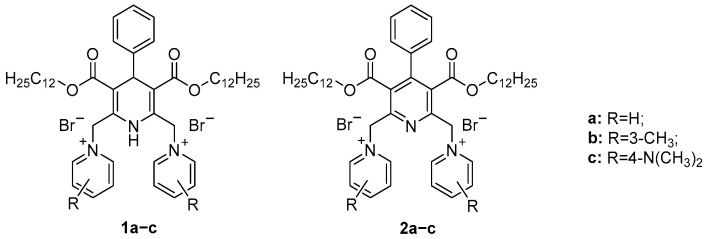
Schematic representation of pyridinium amphiphiles with 1,4-DHP **1** and pyridine **2** linker.

The type, length, and relative orientation of linker bonds influence the transfection efficiency, biodegradability, stability, and cytotoxicity of cationic lipids [3]. Among the earliest studied synthetic lipids were those containing ether and ester linkers; common examples include *N*-(1-(2,3-dioleyloxy)propyl)-*N*,*N*,*N*-trimethylammonium (DOTMA) (ether) [4] and DOTAP (ester) [5]. Another well-known linker group comprises amide linkers, with dioctadecylamidoglycylspermine (DOGS) being a representative example [6]. Several modifications of the mentioned linker types have been introduced by changing the number and length of the linker units, thereby altering the overall properties of the lipids [7,8,9]. Additional linker groups—such as disulfide, carbamate, acylhydrazone, urea, and phosphate—have been studied and recently reviewed by Zhi et al. [10]. Ren et al. reported that aromatic ring-based cationic lipids showed transfection efficiencies comparable to those of DOTAP [11] At the same time, Pennetta et al. demonstrated that triazine-based cationic lipids achieved high gene delivery efficiency, comparable to or exceeding that of branched polyethylenimine (b-PEI) and Lipofectamine 2000 [12]. Among the studied linker types, heterocyclic systems used as amphiphilic lipid linkers have received comparatively little attention in transfection systems. Among the most successful examples are 1,4-dihydropyridine (1,4-DHP) derivatives (Figure 2, compound **1a**), which have also been studied by our research group [13].

In previous studies, we have developed liposome-forming cationic amphiphiles based on a 1,4-DHP core, capable of transfecting *p*DNA into different cell lines in vitro [13,14,15,16]. These studies analyze how structural modifications in different parts of the molecule affect self-assembly and transfection properties. For example, Petrichenko et al. [16] highlighted the crucial importance of the linker between hydrophilic and lipophilic parts. A comparison between analogues **1a**–**c** and **2a**–**c** (Figure 2) demonstrated that replacing the 1,4-DHP linker with pyridine decreased the transfection activity of the compounds by one third.

Direct comparison of five-membered heterocyclic linkers within the same structural scaffold has not been systematically reported. Despite their structural similarity, pyrrole, furan, and thiophene differ in their electronic and physicochemical properties [17], which may affect molecular packing, intermolecular interactions, and lipoplex formation. To extend our knowledge of the significance of the linker, we synthesized five-membered analogues based on the previously identified most active 1,4-DHP derivative **1a**. This approach not only expands the range of studied compounds but also provides insights into the importance of the heterocyclic linker systems—pyrrole, furan, and thiophene. These heterocycles were selected due to their potential as therapeutic agents. Numerous studies have reported a wide range of biological and pharmaceutical activities for pyrrole [18,19,20,21], thiophene [22,23], and furan [24,25]; however, compounds containing cationic moieties have not been studied. The hydrophilic and lipophilic parts were kept the same as in previously developed 1,4-DHP **1a**, with slight variation in alkyl chain length to enable better comparison. As reported previously, transfection activity generally increases with the increasing chain length of saturated aliphatic moieties (C14–C18), whereas shorter chains (C10–C14) enhance membrane fluidity [26,27].

**Figure 3:**
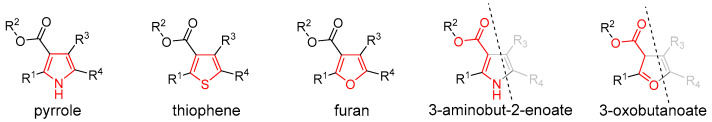
General structures of the studied compounds with five-membered heterocyclic linkers and aliphatic linkers.

In this work, we studied compounds covering both ranges by designing structures with C12, C14, and C16 alkyl chain lengths. Additionally, aliphatic linkers—2-aminobut-2-enoate and 3-oxobutanoate—were established as linear “half products” to better evaluate the role of the linker. Figure 3 depicts the general core structures of the studied compounds.

The effect of heterocyclic linkers in cationic amphiphilic lipids was assessed through analysis of their self-assembly behavior and the parameters of the resulting liposomes and lipoplexes. The study was supplemented with encapsulation efficiency measurements to evaluate the ability of the compounds to bind genetic material—in this case, plasmid DNA pBR322. All obtained data were compared with that of the previously synthesized 1,4-DHP compound **1a** as a control. The obtained results provide preliminary data and may support further evaluation of these compounds for transfection and structure–activity relationship studies. This study introduces a novel approach, as heterocyclic systems of this kind have not been investigated for the development of transfection agent candidates.

## 2. Materials and Methods

### 2.1. Chemistry

More detailed descriptions of the synthetic procedures and characterization of the original unpublished compounds are provided in the Supplementary Materials. Briefly, the heterocyclic key intermediates **14**, **16**, and **17**, for symmetric amphiphiles, and **25** and **33**, for unsymmetric amphiphiles, were synthesized according to procedures described in the literature [28,29,30,31,32,33], with slight modifications for symmetric pyrrole cyclization. Functionalization of these intermediates was performed using similar procedures that involve transesterification (or Steglich esterification), bromination with *N*-bromosuccinimide (NBS) via a radical mechanism, and nucleophilic substitution with pyridine, leading to target symmetric **3a**–**c**, **4a**–**c**, and **5b**–**c** and unsymmetric **6a**–**c** and **7a**–**c** compounds. A similar approach was used to synthesize aliphatic amphiphiles **8a**–**c**, **9a**–**c**, and **10a**–**c**, in combination with methods used in previous studies of related compounds [34].

### 2.2. Self-Assembling Properties of Synthesized Amphiphiles Using Dynamic Light Scattering (DLS) Measurements

Average hydrodynamic diameters (Z_av_) and zeta-potential (Z_pot_) measurements were performed according to previously published procedures, with minor modifications [32,33]. Briefly, samples for the DLS studies were prepared by making 4 mM stock solutions of compounds **3**–**10** in EtOH. Stock solutions of the compounds (25 µL, 4 mM in EtOH 96%) were injected into deionized water (175 µL) under vigorous stirring (IKA Vortex 2, IKA, Staufen, Germany) to produce samples with a final concentration of 0.5 mM. Results of three replicates (*n* = 3) are reported as mean ± standard deviation (±SD). The Z_av_ of the formed nanoparticles, polydispersity index (PDI), and Z_pot_ were determined by DLS using a Zetasizer Nano ZSP (Malvern Instruments Ltd., Malvern, UK). Data were analyzed using the multimodal number distribution software (Malvern Instruments Ltd., Malvern, UK, Software 8.01.4906) included with the instrument. Measurements were performed in triplicate to ensure reproducibility.

The critical micelle concentration (CMC) was determined in aqueous media using a Zetasizer Nano ZSP instrument with Malvern Instruments Ltd. software (Malvern Instruments Ltd., Malvern, UK, Software 8.01.4906). Briefly, 0.5 mM stock solutions of the compounds were serially diluted two-fold with deionized water to obtain final concentrations ranging from 0.5 mM to 0.5 × 10^−3^ mM. The electrical conductivity as a function of amphiphile concentration was analyzed. The electrical conductivity detected for amphiphile concentrations below the CMC has an approximately constant value corresponding to that of water. The conductivity begins to increase linearly with concentration at the CMC, reflecting an increase in the number of nanoparticles in solution. The intersection of the best-fit lines drawn through the data points was taken as the preliminary CMC value of the compounds.

### 2.3. Formation of Lipoplexes and Encapsulation Efficiency Measurements

Compound-*p*DNA complexes (lipoplexes) were prepared by mixing appropriate amounts (N/P ratios 1, 2, 4, and 6) of 0.05 mM lipid solutions in water with 100 ng of *p*DNA pBR322 (purchased from Carl Roth GmbH + Co. KG, Karlsruhe, Germany). Samples were diluted with 1 × Tris-EDTA buffer to a final volume of 300 µL, vortexed, and incubated for 30 min at room temperature.

Quantification of *p*DNA was performed using the Quant-iT PicoGreen *p*DNA Assay Kit (Thermo Fisher Scientific, Waltham, MA, USA), according to the method described by Cui et al. [35], with minor modifications. For sample preparation, 150 μL of each sample was transferred to a new well of a 96-well plate and diluted with 150 μL of 0.1% Triton X-100 to obtain lysed samples, followed by mixing and incubation at 37 °C for 15 min. For control conditions, 150 μL of the same sample was diluted with 150 μL of 1 × Tris-EDTA buffer and vortexed without lysis. Negative amphiphile control samples were processed in parallel using the same lysis and non-lysis procedures to assess background fluorescence originating from the amphiphile formulations. Subsequently, 100 μL aliquots of both control and lysed samples were transferred in duplicate into a black 96-well microplate for fluorescence measurement. The remaining 100 μL of each sample was retained for dynamic light scattering analysis.

For the PicoGreen assay, the commercial PicoGreen reagent was diluted 1:200 in 1 × Tris-EDTA buffer immediately before use. Then, 100 μL of diluted PicoGreen working solution was added to each well containing the sample or standard. The plate was mixed by gentle pipetting or orbital shaking and incubated at room temperature in the dark for 5–10 min. Fluorescence intensity was measured using a Clariostar Plus microplate reader (BMG Labtech, Ortenberg, Germany), with excitation at 475 nm and emission at 500–550 nm.

Encapsulation efficiency (EE%) was determined by measuring the fluorescence of lipoplexes before and after lysis and was calculated using Equation (1):

(1) $$EE\%=\;\frac{\left(Lysed\;sample-Control\;sample\right)}{Lysed\;sample}\;\times 100$$

The DNA concentration in the lipoplex was estimated using a calibration curve obtained from measurements of *p*DNA at known concentrations (Figure S3 in the Supplementary Materials).

An aliquot of the control samples (before PicoGreen addition) was employed to measure the sizes of the formed nanoparticles using DLS.

### 2.4. Gel Retardation Assay

Compound-*p*DNA complexes (lipoplexes) were prepared by mixing appropriate amounts (N/P ratios 0.3, 1, 3, and 5) of 0.5 mM lipid solutions in OptiMEM medium with 1 µg of *p*DNA pEGFP-C1 (purchased from Clontech, Mountain View, CA, USA), with a total volume of 30 μL. The reaction mixture was incubated at room temperature for 25 min to form lipoplexes. Lipoplex formation was determined by agarose gel electrophoresis, taking 10 μL of the reaction mixture. Samples were loaded onto a 1% agarose gel containing ethidium bromide and electrophoresed in Tris-acetate-EDTA (TAE) buffer. Electrophoresis was performed at 120 mA for 40 min. DNA bands were visualized using a UV light. The extent of pDNA complexation was evaluated from the retardation of plasmid migration relative to that of naked pDNA.

### 2.5. Statistical Analysis

Results were expressed as mean standard deviations (±SD). All of the experiments were performed in triplicate (*n* = 3) and analyzed using Microsoft Excel (version 2603). Statistical analysis was performed using GraphPad Prism v10.6.1. Data were analyzed using a Welch’s ANOVA test with Dunnett’s T3 multiple comparisons test. The Pearson’s correlation coefficient was used for correlation analysis. *p*-values less than 0.05 were considered statistically significant.

## 3. Results and Discussion

### 3.1. Synthesis of Symmetric Five-Membered Amphiphiles

**Scheme 1:**
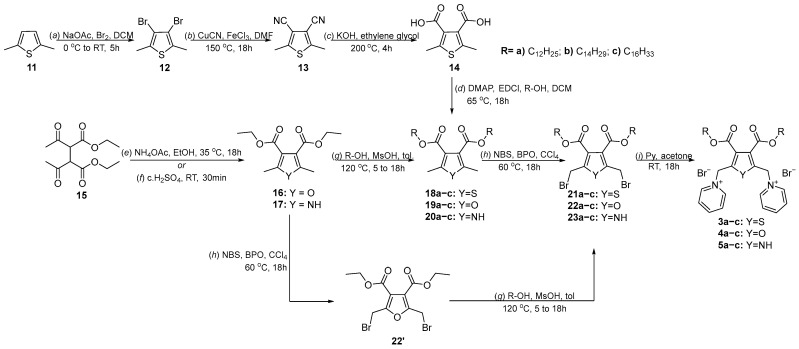
Synthetic route to symmetric five-membered amphiphilic lipid-like compounds **3**–**5**, with pyridinium cations.

For the synthesis of symmetric amphiphiles with a five-membered linker between the hydrophilic head group and lipophilic domain, we propose a reaction sequence depicted in Scheme 1.

Thiophene intermediate **14** was obtained in three steps, according to a procedure described in the literature, starting from commercially available 2,5-dimethylthiophene **11** [28,29,30]. The reaction yields and compound physicochemical properties for these steps were found to comply with those reported in previous studies. Steglich esterification using 1-dodecanol, 1-tetradecanol and 1-hexadecanol was then applied to yield compounds **18a**–**c**, followed by the bromination of the 2,5-methyl groups using NBS in the presence of benzoyl peroxide (BPO). It should be noted that the purification of brominated products **21a**–**c** posed challenges. Therefore, a mixture of a product **21a**–**c** (dialkyl 2,5-bis(bromomethyl)thiophene-3,4-dicarboxylate) and a half-product **21’a**–**c** (dialkyl 2-(bromomethyl)-5-methylthiophene-3,4-dicarboxylate) was used for the last step—nucleophilic substitution of bromide by pyridine. The final products **3a**–**c** were subsequently purified using reversed-phase chromatography. Unpublished reaction steps and detailed descriptions of the obtained compounds are presented in Section S2.1 of the Supplementary Materials. A summary of the synthetic route and overall reaction yields is shown in Table 1, entry 1.

For the synthesis of furan (Scheme 1, steps *f*, *h*, *g*, and *i*) and pyrrole (Scheme 1, steps *e*, *g*, *h*, and *i*) amphiphiles **4a**–**c** and **5b**–**c**, respectively, similar methods were used. In both cases, syntheses were started from commercially available diethyl 2,3-diacetylsuccinate **15.** Under previously published acidic conditions [31], it was cyclized to form furan intermediate **16**, whereas a modified Paal–Knorr pyrrole synthesis yielded pyrrole intermediate **17**. Classical Paal–Knorr pyrrole synthesis usually requires the use of methanolic ammonia solution [36], which is known as a hazardous mixture. To lower the safety risks, we used ammonium acetate, which was easy to handle and also provided a high reaction yield of 96%. Further steps included transesterification of the side-chains with appropriate alcohol (1-dodecanol, 1-tetradecanol, and 1-hexadecanol) and 2,5-methyl group bromination with NBS. For the successful bromination of pyrrole derivatives **20a**–**c**, the protection of the NH group needs to be considered. In our work, we used tert-butyloxycarbonyl (Boc) protection to yield **23a**–**c** because it was stable at bromination conditions and easy to remove afterwards. For the further modifications of furan derivative **16**, a slightly different sequence of reactions was required. Purification of the reaction mixture after the bromination of **19a**–**c** was quite challenging. Therefore, we first performed the bromination of **16**, following the method literature in the [37], to yield product **22’**, and only then did we carry out the transesterification reaction. This resulted in higher reaction yields, and the products were easier to purify. The last step for the pyrrole and furan derivatives was the same as that for the thiophene derivatives. A summary of the syntheses and overall reaction yields of furan amphiphiles **4a**–**c** is shown in Table 1, entry 2, while those of the pyrrole amphiphiles **5a**–**c** are shown in entry 3. Detailed information on the synthesis of the symmetric furan and pyrrole amphiphiles is presented in Sections S2.2 and S2.3, respectively, of the Supplementary Materials.

In summary, the synthesis of furan derivatives required fewer reaction steps (four steps vs. six steps for thiophene and pyrrole), resulting in higher overall reaction yields (Table 1 entry 2, column 5). In all cases, lower reaction yields were obtained for C12 ester derivatives. This could be due to the liquid/oily state of the corresponding intermediates, which makes them unsuitable for purification by crystallization.

### 3.2. Synthesis of Unsymmetric Five-Membered Amphiphiles

**Scheme 2:**
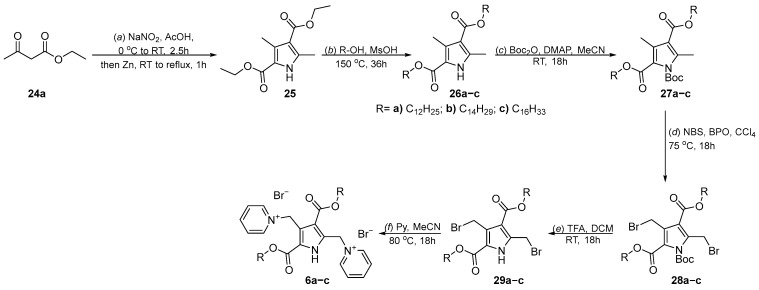
Reaction scheme for the synthesis of unsymmetric pyrrole containing amphiphilic lipid-like compounds **6a**–**c** bearing two lipophilic parts and two hydrophilic parts.

**Scheme 3:**
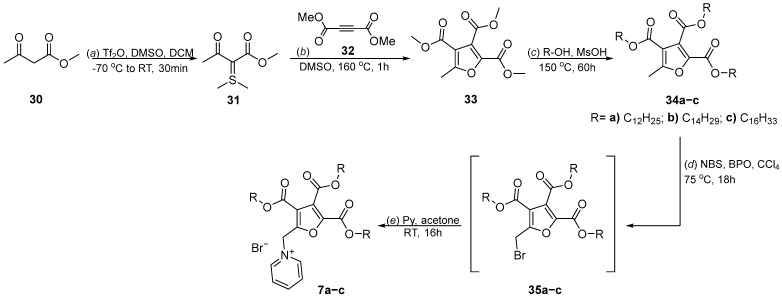
Reaction scheme for synthesis of unsymmetric furan containing amphiphilic lipid-like compounds **7a**–**c** bearing three lipophilic parts and one hydrophilic part.

The amphiphilic compound class with a heterocyclic linker was enriched by the synthesis of similar unsymmetric compounds: bis-cationic moieties containing pyrrole dicarboxylates **6a**–**c** (Scheme 2) and mono-cationic moieties containing furan dicarboxylates **7a**–**c** (Scheme 3). In the first case, changing the position of the lipophilic and hydrophilic parts could result in different liposome properties. In the second case, changing the ratio between the hydrophilic and lipophilic parts could lead to the formation of different types of aggregates in aqueous solution, as described by Israelachvili’s theory of the molecular packing parameter. This concept illustrates how the size and the shape of an aggregate at equilibrium can be predicted based on a combination of molecular packing considerations and general thermodynamic principles [38,39,40].

For the synthesis of unsymmetric pyrrole amphiphiles **6a**–**c**, a seven-step sequence was applied. Reactions are shown in Scheme 2 and described in detail in Section S2.4 of the Supplementary Materials. Briefly, commercially available ethyl 3-oxobutanoate was used in the previously published Knorr reaction to produce intermediate **25** [32]. The reaction yield and compound physicochemical properties for step a are found to comply with those reported in previous studies. Subsequent steps *b*, *c*, *d*, and *e* followed the same methods used for the symmetric pyrrole amphiphiles. Only minor changes were introduced in the final step *f*, where nucleophilic substitution was carried out at a higher temperature, using acetonitrile as the solvent, replacing acetone.

For the synthesis of unsymmetric furan amphiphiles **7a**–**c**, commercially available methyl 3-oxobutanoate was selected as starting material due to easier achievement of intermediate **33** transesterification at *c* step. Also, the synthesis of intermediate **33** had already been demonstrated by Xu et al. [33]. Unlike the procedure used by these authors, we decided not to perform step *b* under microwave irradiation, which resulted in a slight reduction in reaction yield. Further transesterification reaction with the appropriate alcohol (1-dodecanol, 1-tetradecanol or 1-hexadecanol) was monitored by liquid chromatography–mass spectrometry (LC/MS) analysis to determine the appropriate reaction time. Due to the bulky structure of compound **34a**–**c**, transesterification reactions took longer, resulting in lower yields compared to those of symmetric furan intermediates **19a**–**c**. Additionally, the subsequent bromination reaction proved challenging, yielding only an inseparable mixture of product **35a**–**c** and starting material **34a**–**c**. In the final step, nucleophilic substitution was performed with the reaction mixture, and the desired compound **7a**–**c** was purified afterward. The reactions are shown in Scheme 3 and described in detail in Section S2.5 of the Supplementary Materials. A summary of the synthesis of unsymmetric pyrrole and furan amphiphiles is presented in Table 2, entries 1 and 2, respectively.

In summary, the overall reaction yields for the synthesis of unsymmetric furan amphiphiles are lower than those for unsymmetric pyrrole amphiphiles (Table 2 entries 2 and 1, respectively), despite the smaller number of reaction steps. We propose that this difference may be related to steric hindrance arising from the presence of three lipophilic substituents. The overall reaction yields of the unsymmetric pyrrole derivatives (6.9–13.1%) are comparable to those of the symmetric furan derivatives (12.4–16.3%) and higher than those of their symmetric derivatives (thiophene: 2.6–6.5%; pyrrole: 3.1–6.4%). These differences do not relate to the number of reaction steps but may instead be associated with material losses during bromination reactions and related purification difficulties.

**Scheme 4:**
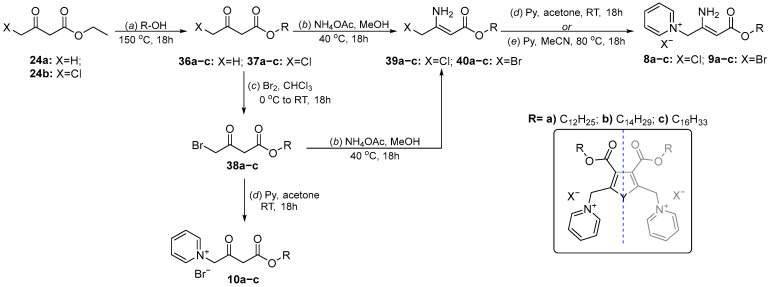
Scheme for synthesis of amphiphiles with aliphatic linkers: 2-aminobut-2-enoates **8a**–**c** and **9a**–**c** and 3-oxobutanoates **10a**–**c**.

For a better understanding of the role of heterocycles in the structure, we chose to synthesize compounds with aliphatic linkers that are structurally similar to heterocycles and resemble linear “half-products”. The proposed reaction sequence consists of three to four reaction steps and is depicted in Scheme 4.

The synthetic route begins with the transesterification of either ethyl 3-oxobutanoate **24a** or ethyl 4-chloroacetoacetate **24b**, depending on the anion desired in the final compound. The reactions were carried out with the appropriate alcohol under solvent-free conditions, with azeotropic removal of ethanol. The further reaction of compounds **37a**–**c** with NH_4_OAc led to the formation of enaminoesters **39a**–**c**. The description of the used method is presented in our previous work [34]. The same procedure was used for the synthesis of compounds **40a**–**c** after bromination of compounds **36a**–**c** with bromine in chloroform. Bromination at the 4-carbon position was based on a previously published method [41]. Formation of the desired compound was confirmed by proton nuclear magnetic resonance (^1^H NMR) spectra in CDCl_3_ solution (Figure S1). The last step yielding the corresponding pyridinium salts **8a**–**c**, **9a**–**c**, and **10a**–**c** involved a nucleophilic attack of pyridine to the halide precursors. The same procedure used for heterocyclic compounds **21**–**23** was applicable to the bromide derivatives **38a**–**c** and **40a**–**c**, whereas the less reactive chloride derivatives **39a**–**c** required modified conditions. Consequently, this reaction was performed under different conditions with higher temperatures. Formation of the *Z*-isomer for compound **9b** was confirmed by NOESY spectra (Figure S2), wherein a cross-peak between protons at the 2-carbon and 4-carbon positions was observed. For the remaining products (**8a**–**c** and **9a**,**c**), the ^1^H NMR spectra were comparable, and their configurations were also assigned as *Z*-isomers. A summary of the synthesis of amphiphiles with aliphatic linkers is demonstrated in Table 3. More detailed descriptions of previously unpublished synthetic procedures and the characterization of the new compounds are described in Section S2.6 of the Supplementary Materials.

During the synthesis of amphiphiles with aliphatic linkers (**8a**–**c**, **9a**–**c**, and **10a**–**c**), it was challenging to identify suitable purification methods for the intermediates. This resulted in complex reaction mixtures and lower overall reaction yields, particularly for compounds **9a**–**c**, which exhibited overall yields of only 0.9−2.0% (Table 3, entry 2). Although the synthesis of aliphatic amphiphiles required a smaller number of reaction steps (Table 3, column 3) compared with those for the heterocyclic amphiphiles (Table 1 and Table 2, column 3), the overall reaction yields were still lower in most cases. In summary, both aliphatic amphiphiles and symmetric heterocyclic amphiphiles tend to give higher overall yields with C16 esters. For example, the overall yield of aliphatic amphiphile with C16 ester **10c** is 9.2%, while for C12 ester **10a**, it is 5.9%; the symmetric heterocyclic amphiphile with C16 ester **3c** is 6.5%, while for C12 ester **3a**, it is 2.6%. The opposite tendency was detected with unsymmetric heterocyclic amphiphiles, where the overall yield of C16 ester **6c** is 6.9%, while for the C12 ester, it is 11.3%.

The structures of all compounds were established and confirmed on the base of the ^1^H NMR and ^13^C NMR (Figures S5–S125, Supplementary Materials). The purities of the studied amphiphiles were at least 97%, according to high-performance liquid chromatography data.

### 3.3. Evaluation of Self-Assembling Properties

The ability to form nanoparticles in an aqueous medium is related to the amphiphilic nature of the synthesized molecules. The dynamic light scattering (DLS) method was used for the evaluation of average hydrodynamic diameters (Z_av_), polydispersity index (PDI), zeta-potential (Z_pot_) and critical micelle concentration (CMC) using the conductometry method. Nanoparticles were prepared using the ethanol injection method, which required dissolving the lipid-like compound in ethanol and then dispersing the resulting lipid solution into water. The final solution concentration for the measurements was 0.5 mM for better comparison, except for the CMC measurements, which required a dilution series (chosen concentration range: 2 mM to 1 × 10^−3^ mM). Data concerning zeta-potential and CMC are summarized in Table 4.

Zeta-potential values provide information about the surface charge of nanoparticles. Typically, for nanoparticle solutions to be considered as relatively stable, their zeta-potential must be above +30 mV or below −30 mV [42,43]. However, even a small amount of ethanol in the solution can significantly affect both the Zeta potential and hydrodynamic diameters of the formed nanoparticles [44,45].

CMC values define the concentration above which micelles are formed. According to Felice et al. [46], CMC values for most amphiphiles are in the range of 10^−4^–10^−2^ M, whereas phospholipids exhibit four to five times lower values, indicating lower aqueous solubility and higher stability compared to those of other micelles [47]. In our case, CMC values of the tested amphiphiles range from 17 to 60 µM for cyclic compounds (symmetric: Table 4, entry 2; unsymmetric: Table 4, entry 4) and from 30 to 75 µM for aliphatic compounds (Table 4, entry 6). The obtained results are in agreement with the observations reported by Felice et al.

**Figure 4:**
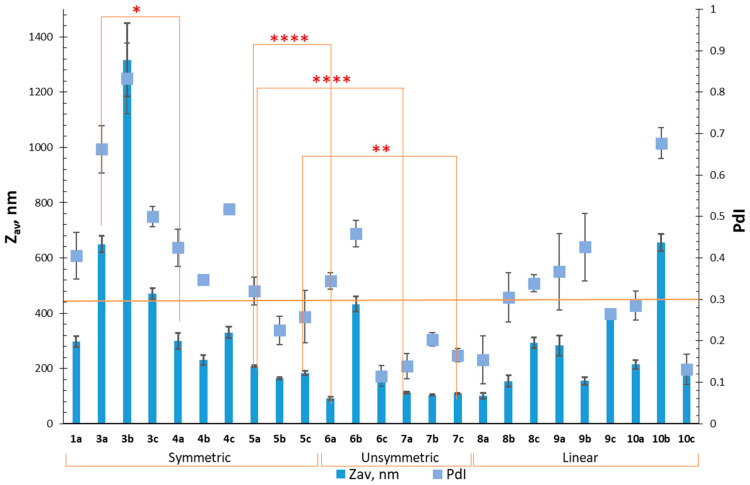
Values of the average hydrodynamic diameters (Z_av_) and polydispersity index (PDI) of freshly prepared liposomes formed by symmetric (**1a**, **3a**–**c**, **4a**–**c**, **5a**–**c**), unsymmetric (**6a**–**c**, **7a**–**c**), and aliphatic (**8a**–**c**, **9a**–**c**, **10a**–**c**) amphiphiles in aqueous solution at 0.5 mM concentration via DLS measurement. Error bars represent ±SD from three independent experiments. Error bars smaller than the data point size are not visible. The yellow line indicates a PDI value of 0.3, commonly considered the upper limit for a relatively homogeneous and stable system. Statistical significance is indicated as follows: *—*p* < 0.05; **—*p* < 0.01; ****—*p* < 0.0001. All calculated *p*-values are summarized in Table S2 (Supplementary Materials).

It is known that the size of nanoparticles significantly influences the pathway of cellular entry, transfection efficiency, cytotoxicity, and in vivo biodistribution. Although smaller nanoparticles are generally considered more efficient for transfection, larger particles may still be relevant for certain applications. Additionally, the optimal size range remains uncertain and may vary depending on the compound itself, as different studies have shown varying effects of particle size [48]. The average hydrodynamic diameters and polydispersity indexes of the nanoparticles formed in our study are summarized in Table S1 (Supplementary Materials) and depicted in Figure 4.

The data show that both the average hydrodynamic diameter and polydispersity index depend on the compound structure. Similar trends were observed among structurally related compounds. For example, the sizes of nanoparticles formed from symmetric amphiphiles ranged from 164 nm to 1317 nm. The largest nanoparticles were formed from amphiphiles containing a thiophene linker (**3a** Z_av_ = 650 nm; **3b** Z_av_ = 1317 nm; **3c** Z_av_ = 470 nm). Amphiphiles containing a furan linker formed smaller nanoparticles (**4a** Z_av_ = 299 nm, **4b** Z_av_ = 230 nm, **4c** Z_av_ = 330 nm), comparable in size to those formed from amphiphile **1a** (Z_av_ = 298 nm). The smallest nanoparticles were formed from amphiphiles containing a pyrrole linker (**5a** Z_av_ = 208 nm, **5b** Z_av_ = 164 nm, **5c** Z_av_ = 183 nm). However, the results did not reach statistical significance (see Table S2 in Supplementary Materials). Meanwhile, the aliphatic amphiphiles—**8a**–**c**, **9a**–**c**, and **10a**–**c**—form nanoparticles ranging in size from 100 nm to 656 nm. The sizes of nanoparticles formed from unsymmetric amphiphiles—**6a**–**c** and **7a**–**c**—are generally smaller than those formed from compounds **5a**–**c** (**5a** vs. **6a**: *p* = 0.0001; **5a** vs. **7a**: *p* < 0.0001), except for the case of compound **6b**, which forms nanoparticles with an average hydrodynamic diameter of 433 nm. Overall, the most homogeneous nanoparticles were formed by symmetric amphiphiles **5b** and **5c**, unsymmetric amphiphiles **6c** and **7a**–**c**, and aliphatic amphiphiles **8a**, **9c**, and **10c**, with PDI values below 0.3.

### 3.4. Encapsulation Efficiency and Characterization of Lipoplexes

**Figure 5:**
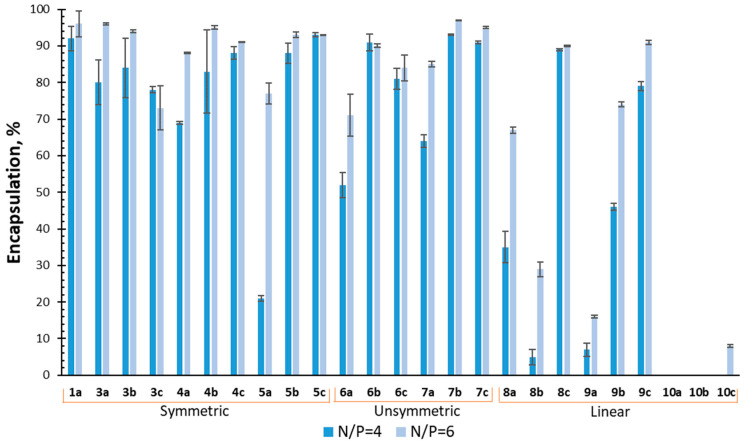
Encapsulation of plasmid DNA pBR322 in liposomes formed by symmetric (**1a**, **3a**–**c**, **4a**–**c**, **5a**–**c**), unsymmetric (**6a**–**c**, **7a**–**c**), and aliphatic (**8a**–**c**, **9a**–**c**, **10a**–**c**) amphiphiles at N/P = 4 and N/P = 6 ratios detected by the PicoGreen assay. Error bars represent ±SD from three independent experiments.

Cationic lipid–DNA complexes (lipoplexes) were prepared at four different N/P ratios (1, 2, 4, and 6) using symmetric (**1a**, **3a**–**c**, **4a**–**c**, **5a**–**c**), unsymmetric (**6a**–**c**, **7a**–**c**), and aliphatic (**8a**–**c**, **9a**–**c**, **10a**–**c**) amphiphiles, together with plasmid DNA pBR322. The N/P ratio governs the electrostatic self-assembly of the nanoparticles. A higher N/P ratio provides more positively charged centers to condense and fully encapsulate the negatively charged nucleic acids. At lower N/P ratios, the cationic lipid may not fully bind the negatively charged DNA, leading to a low encapsulation percentage (Table S3, entries 1, 2, 5, 6, 9, 10). However, excessively high N/P ratios may compromise the structural integrity of the complex, increase toxicity, or hinder the release of the nucleic acid inside the target cell. To assess possible interference of the amphiphile formulations with the PicoGreen assay, control samples containing only amphiphiles were prepared and analyzed under identical conditions. These controls exhibited fluorescence signals at background levels, indicating that the amphiphiles themselves did not interfere with the PicoGreen fluorescence measurements. Encapsulation experiment results are summarized in Table S3 (Supplementary Materials) and depicted in Figure 5.

As expected, higher encapsulation efficiency (EE ≥ 78%) was achieved at N/P ratios of at least 4, except for complexes with compounds **4c** and **5c**—already at an N/P ratio of 2—which reached EE values of 78% and 80%, respectively (Table S3, entry 2). Additionally, when analyzing lipoplexes formed from cyclic amphiphiles, we observed that the best results were mostly obtained with amphiphiles containing C14 ester side-chains. Only two lipoplexes formed from aliphatic amphiphiles (**8c** and **9c**) were able to encapsulate more than 80% *p*DNA. Additionally, aliphatic amphiphiles **10a** and **10b** showed no encapsulation at any of the tested N/P ratios, whereas aliphatic amphiphile **10c** displayed only 8% EE at an N/P ratio of 6. Based on these results, aliphatic structures appear to be less effective under the studied conditions and may therefore be less suitable for further studies of transfection activity. Additionally, the results highlight the crucial role of the NH_2_ group for complexation with genetic material in aliphatic amphiphiles.

**Figure 6:**
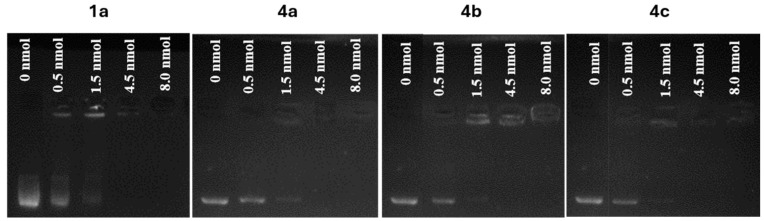
Gel retardation assay of amphiphiles **1a** and **4a**–**c** incubated with 1 µg *p*DNA pEGFP-C1 at different concentrations.

To provide direct evidence for DNA binding and plasmid integrity, the ability of selected amphiphiles **1a**, **4a**–**c** to form lipoplexes with *p*DNA was evaluated using an agarose gel retardation assay. The results are shown in Figure 6.

The selected amphiphiles included 1,4-DHP compound **1a** (used as a reference) and the symmetric furan amphiphiles **4a**–**c**. In all cases, increasing the amount of the transfection agent decreased the amount of free DNA. All tested compounds completely bound 1 µg of *p*DNA at concentrations ranging from 1.5 to 4.5 nmol (N/P ratios of 1 to 3). Interestingly, as the amount of the compound increased, DNA bound within the complexes (lipoplex DNA band) was no longer detectable for compound **1a** at 8.0 nmol, whereas for compounds **4a** and **4c**, it already became undetectable at 4.5 nmol. In contrast, for compound **4b**, the lipoplex DNA band remained detectable even at 8.0 nmol.

**Figure 7:**
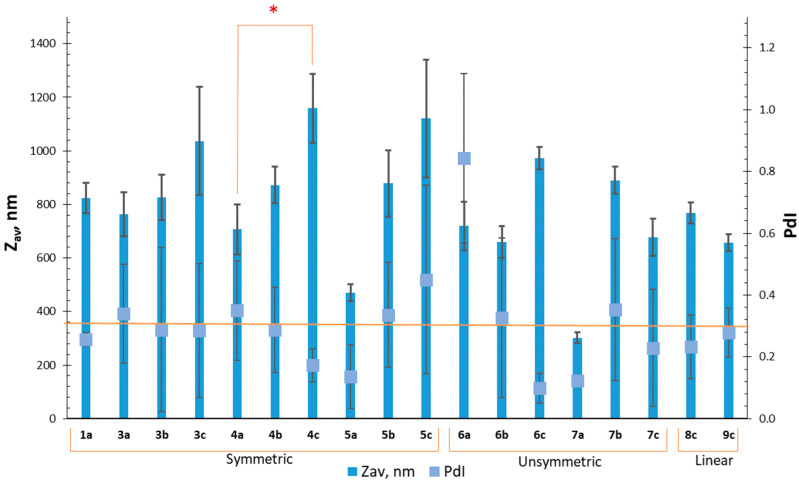
Values of the average hydrodynamic diameters (Z_av_) and polydispersity indexes (PDI) of the lipoplexes formed from symmetric (**1a**, **3a**–**c**, **4a**–**c**, **5a**–**c**), unsymmetric (**6a**–**c**, **7a**–**c**), and aliphatic (**8c**, **9c**) amphiphiles with plasmid DNA pBR322 at N/P ratio = 6. Error bars represent ±SD from three independent experiments. Error bars smaller than the data point size are not visible. The yellow line indicates a PDI value of 0.3, commonly considered the upper limit for a relatively homogeneous and stable system. Statistical significance is indicated as follows: *—*p* < 0.05. All calculated *p*-values are summarized in Table S2 (Supplementary Materials).

Lipoplexes exhibiting an encapsulation efficiency of at least 78% at an N/P ratio of 6 were also evaluated for their average hydrodynamic diameters and polydispersity indexes. Results are summarized in Table S4 (Supplementary Materials) and depicted in Figure 7.

The obtained data demonstrate that most of the lipoplexes have similar average hydrodynamic diameters. Additionally, a trend can be observed with changes in the ester side-chain length: Z_av_ increases as the ester side-chain in the amphiphile increases from C12 to C16. For example, the Z_av_ values of symmetric thiophene amphiphiles **3a** (C12), **3b** (C14), and **3c** (C16) increased from 762 nm to 826 nm and 1037 nm, respectively (Pearson’s correlation coefficient r = 0.96; *p* = 0.09). The corresponding correlation plot is shown in Figure S4 (Supplementary Materials). A different trend was observed for unsymmetric amphiphiles. The Z_av_ values of unsymmetric pyrrole amphiphiles **6a**–**c** were 720, 660, and 972 nm, respectively, whereas the Z_av_ values of unsymmetric furan amphiphiles **7a**–**c** were 302, 890, and 677 nm, respectively.

**Figure 8:**
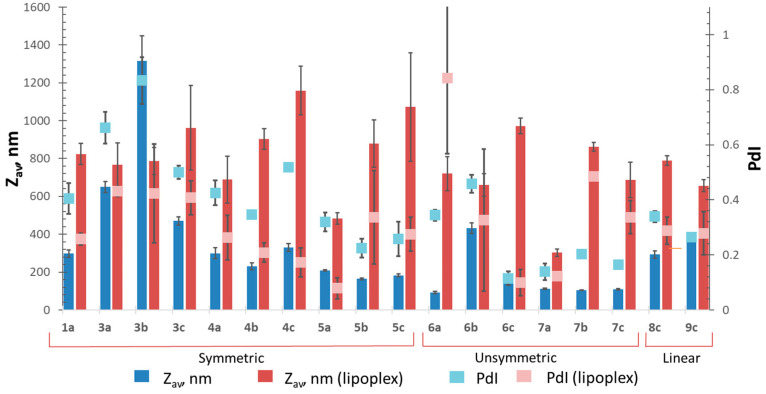
Comparison of the average hydrodynamic diameters (Z_av_) and polydispersity indexes (PDI) of the freshly prepared liposomes (blue) and lipoplexes with *p*DNA pBR322 at N/P ratio = 6 (red) formed from symmetric (**1a**, **3a**–**c**, **4a**–**c**, **5a**–**c**), unsymmetric (**6a**–**c**, **7a**–**c**), and aliphatic (**8c**, **9c**) amphiphiles. Error bars represent ±SD from three independent experiments. Error bars smaller than data point size are not visible. A PDI value of 0.3 (yellow line) or lower is considered a standard benchmark for a relatively homogeneous and stable system.

When comparing the Z_av_ values of lipoplexes with those of nanoparticles formed without encapsulated *p*DNA, an overall tendency toward increased Z_av_ values was observed in most cases, while PDI values generally decreased, indicating more homogeneous systems (Figure 8).

For example, symmetric furan amphiphiles **4a**–**c** formed nanoparticles (liposomes) in aqueous solution, with Z_av_ values of 299, 230, and 330 nm, respectively. Upon complexation with *p*DNA, the corresponding lipoplexes showed more than a twofold increase in size, with Z_av_ values of 707, 872, and 1159 nm, respectively. Additionally, PDI values of liposomes formed from compounds **4a**–**c** were 0.425, 0.347, and 0.518, respectively, whereas the corresponding lipoplexes exhibited lower values—0.350, 0.287, and 0.173, respectively. A similar tendency was also observed for amphiphile **1a**, where Z_av_ values were 298 and 284 nm, but PDI values were 0.405 and 0.257, respectively. The data for comp. **1a** are in agreement with our previous findings, where Z_av_ values of nanoparticles and lipoplexes were 398 and 806 nm (N/P ratio 1), respectively [49]. A decrease in Z_av_ value after lipoplex formation with *p*DNA was observed only for the compound **3b**–Z_av_ (liposome) = 1317 nm; Z_av_ (lipoplex) = 826 nm. An increase in PDI values after lipoplex formation was observed for symmetric compounds **5b** (0.225 vs. 0.336) and **5c** (0.258 vs. 0.450), as well as for the unsymmetric compounds **6a**, **7b**, and **7c**, indicating greater heterogeneity of the samples (**6a**: 0.344 vs. 0.842; **7b**: 0.203 vs. 0.353; **7c**: 0.165 vs. 0.229). The larger sizes observed for lipoplexes when compared with the corresponding liposomes are consistent with the formation of lipid–DNA complexes. Electrostatic interactions between cationic amphiphiles and *p*DNA may promote particle association and the formation of larger supramolecular assemblies, resulting in increased hydrodynamic diameters [50]. However, excessively large complexes may be less favorable for biological applications and could require further formulation optimization.

Overall, these observations suggest that, in aqueous solution, liposomes formed from unsymmetric amphiphiles are more homogenous than lipoplexes formed from the same compounds after complexation with *p*DNA. In some cases, the unsymmetric structure may influence *p*DNA complexation. However, this effect is not consistent across all compounds. For example, the lipoplex formed from compound **6a** exhibits the highest PDI value (0.842) and a comparatively lower encapsulation efficiency (71%), suggesting less efficient complex formation, in this case.

## 4. Conclusions

A series of novel cationic amphiphilic lipids incorporating five-membered heterocyclic linkers (pyrrole, furan, and thiophene) were designed, synthesized, and evaluated alongside structurally related aliphatic analogues.

All compounds formed nanoparticles in aqueous media, although particle size, dispersity, and CMC were strongly dependent on linker type, molecular symmetry, and alkyl chain length. Formation of lipoplexes with *p*DNA generally resulted in increased particle size, while effects on polydispersity varied, depending on molecular architecture. The encapsulation studies showed that heterocyclic linkers enhance *p*DNA binding efficiency compared to that of aliphatic systems, with optimal performance typically observed at an N/P ratio of 4 to 6. In contrast, several aliphatic derivatives showed limited or negligible complexation under the investigated conditions. The incorporation of five-membered heterocyclic linkers influences the physicochemical properties of amphiphilic lipids and enhances *p*DNA binding efficiency. The obtained results provide structure–property relationship data that may support the design of lipid-based nucleic acid delivery materials in the future.

## Figures and Tables

**Table 1:** Summary of the synthetic route to yield five-membered amphiphiles **3**–**5**.

Entry	Starting Material	Reaction Steps	Final Products	Overall Reaction Yield, %
1	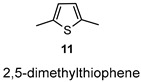	6	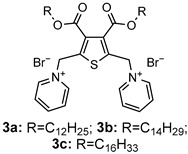	**3a:** 2.6 **3b:** 4.2 **3c:** 6.5
2	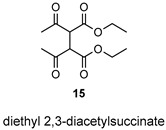	4	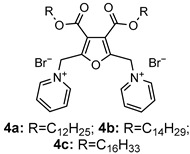	**4a:** 12.4 **4b:** 14.4 **4c:** 16.3
3	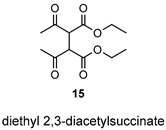	6	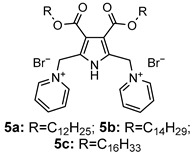	**5a:** 3.1 **5b:** 6.4 **5c:** 4.0

**Table 2:** Summary of the synthetic route to yield unsymmetric five-membered amphiphiles **6a**–**c** and **7a**–**c**.

Entry	Starting Material	Reaction Steps	Final Products	Overall Reaction Yield, %
1	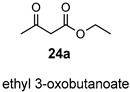	7	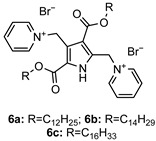	**6a:** 11.3 **6b:** 13.1 **6c:** 6.9
2	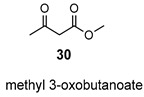	5	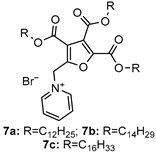	**7a:** 1.0 **7b:** 1.4 **7c:** 0.7

**Table 3:** Summary of the synthetic route to yield amphiphiles with aliphatic linkers **8a**–**c**, **9a**–**c**, and **10a**–**c**.

Entry	Starting Material	Reaction Steps	Final Products	Overall Reaction Yield, %
1	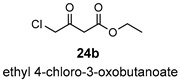	3	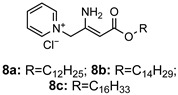	**8a:** 6.0 **8b:** 8.2 **8c:** 10.6
2	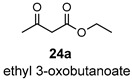	4	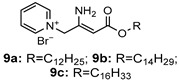	**9a:** 0.9 **9b:** 1.4 **9c:** 2.0
3	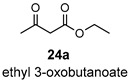	3	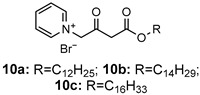	**10a:** 5.9 **10b:** 7.8 **10c:** 9.2

**Table 4:** Values of the zeta-potentials (Z_pot_) and critical micelle concentrations (CMC) of freshly prepared nanoparticles formed by symmetric (**1a**, **3a**–**c**, **4a**–**c**, **5a**–**c**), unsymmetric (**6a**–**c**, **7a**–**c**), and aliphatic (**8a**–**c**, **9a**–**c**, **10a**–**c**) amphiphiles in aqueous solution via DLS measurements.

Entry			1a	3a	3b	3c	4a	4b	4c	5a	5b	5c
1	**Symmetric comp.**	Z_pot_, mV	19 ± 0.9	22 ± 1.4	17 ± 1.5	28 ± 1.5	20 ± 1.1	15 ± 1.2	28 ± 0.3	11 ± 1.1	21 ± 1.1	17 ± 0.9
2		CMC, µM	17–25	22–30	23–28	15–21	45–60	27–40	21–40	12–20	10–20	12–18
			**6a**	**6b**	**6c**	**7a**	**7b**	**7c**				
3	**Unsymmetric comp.**	Z_pot_, mV	16 ± 1.9	14 ± 1.7	16 ± 1.9	29 ± 2.8	21 ± 1.9	23 ± 1.4				
4		CMC, µM	30–45	15–20	25–28	30–50	30–45	25–32				
			**8a**	**8b**	**8c**	**9a**	**9b**	**9c**	**10a**	**10b**	**10c**	
5	**Aliphatic comp.**	Z_pot_, mV	17 ± 2.5	18 ± 0.2	24 ± 1.0	15 ± 6.3	15 ± 1.3	25 ± 3.4	14 ± 1.3	19 ± 1.4	23 ± 2.4	
6		CMC, µM	55–65	50–60	45–60	30–54	45–50	30–40	48–75	35–55	32–40	

## Data Availability

The original contributions presented in this study are included in the article/Supplementary Materials. Further inquiries can be directed to the corresponding authors.

## Supplementary Materials

The following supporting information can be downloaded at: https://www.mdpi.com/article/10.3390/ma19132744/s1.
