# Supplementary material for: Cationic Amphiphiles with Five-Membered Heterocyclic Linkers: Synthesis, Self-Assembly, and DNA Complexation Properties
Source: Materials (Basel). 2026 Jun 26;19(13):2744. doi: 10.3390/ma19132744 (PMC13362763; doi:10.3390/ma19132744)
Supplement: Supplementary file 1 [file materials-19-02744-s001.zip › materials-4345793-supplementary.pdf]

Supplementary Materials

Cationic Amphiphiles with Five-Membered Heterocyclic Linkers: Synthesis, Self-Assembly, and DNA Complexation Properties

Anda Sipola [1,2,*], Ksenija Korotkaja [3], Karlis Pajuste [1], Aiva Plotniece [1,4], Arkadij Sobolev [1,*]

[1] Latvian Institute of Organic Synthesis, Aizkraukles 21, LV-1006 Riga, Latvia; anda.sipola@gmail.com (An.S.); kpajuste@osi.lv (K.P.); arkady@osi.lv (Ar.S.); aiva@osi.lv (A.P.)

[2] Faculty of Medicine and Life Sciences, University of Latvia, Jelgavas 1, LV-1004 Riga, Latvia

[3] Latvian Biomedical Research and Study Centre, Ratsupites 1 k-1, LV-1067 Riga, Latvia; ksenija.korotkaja@biomed.lu.lv (K.K.)

[4] Department of Applied Pharmacy, Faculty of Pharmacy, Riga Stradiņš University, Konsula 21, LV-1007 Riga, Latvia

* Correspondence: anda.sipola@gmail.com (An.S.); arkady@osi.lv (Ar.S.)

S1. General

All reagents were purchased from Acros Organics (Geel, Belgium), TCI Europe (Zwijndrecht, Belgium), Fluorochem (Hadfield, UK) or Alfa Aesar (Lancashire, UK) and used without further purification. Silica gel of particle size 35–70 μm (Merck KGaA, Darmstadt, Germany) and Biotage SNAP cartridge KP-C18-HS together with flash chromatograph IsoleraOne 3.0 (Biotage Sweden AB, Uppsala, Sweden) were used for column chromatography. The ^{1}H-NMR and ^{13}C-NMR spectra were recorded with a Bruker Avance Neo spectrometers at 300, 400 or 600 MHz (^{1}H) and 101 or 151 MHz (^{13}C) (Bruker BioSpin GmbH, Rheinstetten, Germany). ^{1}H-NMR spectra were calibrated to the residual solvent peaks of undeuterated chloroform $CDCl_3$ (δ: 7.26 ppm) or methanol CD_3OD (δ: 4.87 or 3.31 ppm). ^{13}C-NMR spectra were calibrated to the ^{13}C solvent signal ($CDCl_3$ δ: 77.16 ppm; CD_3OD δ: 49.00 ppm). Chemical shifts (δ) are given in parts per million (ppm), and coupling constants (*J*) are given in hertz (Hz). Abbreviations: s – singlet, br. s – broad singlet, d – doublet, t – triplet, q – quartet, m – multiplet, dd – doublet of doublets, tt – triplet of triplets. High-resolution mass spectra (HRMS) were determined on an Acquity UPLC H-Class system (Waters, Milford, MA, USA) connected to a Waters Synapt GII Q-ToF operating in the ESI positive or negative ion mode on a Waters Acquity UPLC® BEH C18 column (1.7 μm, 2.1 × 50 mm, using gradient elution with acetonitrile (0.1% formic acid) in water (0.1% formic acid). Elemental analyses were determined on an Elemental Combustion System ECS 4010 (Costech International S.p.A., Milano, Italy) at the Laboratory of Chromatography of the Latvian Institute of Organic Synthesis. Melting points were determined on an OptiMelt digital melting point apparatus (Stanford Research Systems, Sunnyvale, CA, USA) and are uncorrected.

S2. Synthesis

S2.1. Symmetric thiophenes 3a-3c

<u>*General procedure for Steglich esterification:*</u>

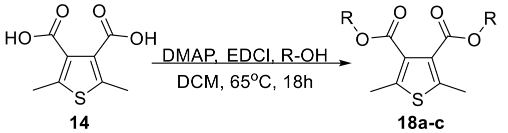

2,5-Dimethylthiophene-3,4-dicarboxylic acid **14** (1.0 eq), DMAP (2.1 eq) and EDCl (2.1 eq) were dissolved in anhydrous DCM. The appropriate alcohol (3.5 eq) was then added and the reaction mixture was stirred at 65 °C for 18 h. The reaction mixture was cooled to room temperature, washed with H_2O (2 × 5 mL) and brine (1 × 5 mL). The organic layers were combined, dried over Na_2SO_4, filtered and evaporated. The crude product was purified with column chromatography (gradient: 0 to 50 % EtOAc in PE) and crystallized from EtOH.

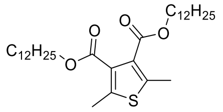

Didodecyl 2,5-dimethylthiophene-3,4-dicarboxylate (18a): Light-yellow solid, mp. 26.7-27.5 °C (EtOH), yield 75 %. ^{1}H-NMR (400 MHz, $CDCl_3$, δ): 4.21 (t, *J*=6.8 Hz, 4H), 2.49 (s, 6H), 1.73-1.64 (m, 4H), 1.43-1.19 (m, 36H), 0.86 (t, *J*=6.7 Hz, 6H) ppm. ^{13}C-NMR (101 MHz, $CDCl_3$, δ): 164.6, 141.3, 130.0, 65.4, 32.1, 29.80, 29.78, 29.74, 29.69, 29.49, 29.45, 28.8, 26.2, 22.8, 14.4, 14.3 ppm.

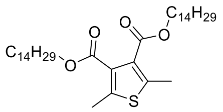

Ditetradecyl 2,5-dimethylthiophene-3,4-dicarboxylate (18b): Light-yellow solid, mp. 39.1-39.5 °C (EtOH), yield 68 %. ^{1}H-NMR (400 MHz, $CDCl_3$, δ): 4.21 (t, *J*=6.8 Hz, 4H), 2.49 (s, 6H), 1.72-1.63 (m, 4H), 1.42-1.19 (m, 44H), 0.88 (t, *J*=6.7 Hz, 6H) ppm. ^{13}C-NMR (101 MHz, $CDCl_3$, δ): 164.6, 141.3, 130.0, 65.4, 32.1, 29.84, 29.83, 29.80, 29.75, 29.69, 29.51, 29.46, 28.8, 26.2, 22.8, 14.4, 14.3 ppm. Anal. calcd for $C_{36}H_{64}O_4S$: C, 72.92; H, 10.88; S, 5.41; found: C, 72.90; H, 11.07; S, 5.29.

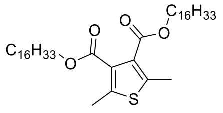

Dihexadecyl 2,5-dimethylthiophene-3,4-dicarboxylate (18c): Light-yellow solid, mp. 46.5-47.0 °C (EtOH), yield 36 %. ^{1}H-NMR (400 MHz, $CDCl_3$, δ): 4.22 (t, *J*=6.8 Hz, 4H), 2.49 (s, 6H), 1.72-1.64 (m, 4H), 1.40-1.21 (m, 52H), 0.87 (t, *J*=6.8 Hz, 6H) ppm. ^{13}C-NMR (101 MHz, $CDCl_3$, δ): 164.6, 141.3, 130.0, 65.4, 32.1, 29.85, 29.83, 29.81, 29.70, 29.51, 29.46, 28.8, 26.2, 22.8, 14.4, 14.3 ppm. Anal. calcd for $C_{40}H_{72}O_4S$: C, 74.02; H, 11.18; S, 4.94; found: C, 74.01; H, 11.46; S, 4.80.

General procedure for the bromination of methyl groups and bromine nucleophilic substitution in thiophene derivatives:

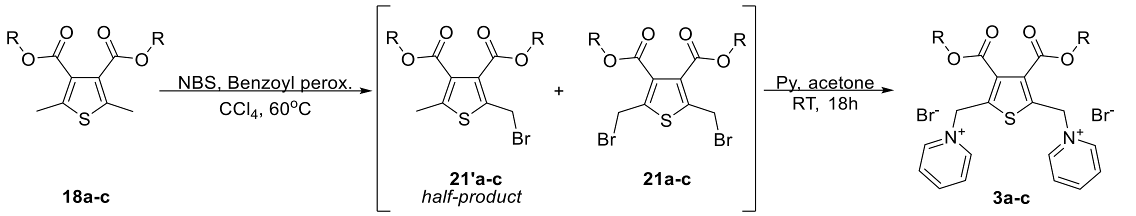

Dialkyl 2,5-dimethylthiophene-3,4-dicarboxylate **18** (1.0 eq), NBS (2.1 eq) and benzoyl peroxide (0.2 eq) were suspended in CCl_4 and stirred at 60 °C for 18 h. Followed by quenching with 10 mL H_2O and extraction with DCM (3 × 5 mL). The crude product was purified with column chromatography (gradient: 0 to 20 % EtOAc in PE). Half-product **21′** and product **21** could not be fully separated; therefore, the mixture was used in next steps without further purification. The formation of compounds **21a-c** was confirmed by ^{1}H-NMR through the observation of the main peaks. Mixture containing dibrominated compound **21** (1.2 eq) was dissolved in dry acetone and then pyridine (3.0 eq) was added. Reaction mixture was stirred at room temperature for 18 h. The resulting mixture was evaporated and purified with reverse phase chromatography (gradient: 0 to 80 % MeOH in H_2O), followed by crystallization from acetone.

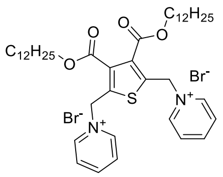

1,1'-((3,4-Bis((dodecyloxy)carbonyl)thiophene-2,5-diyl)bis(methylene))bis(pyridin-1-ium) dibromide (3a): White solid, mp. 164.1-164.9 °C (acetone), yield: 19 %. ^{1}H-NMR (400 MHz, CD_3OD, δ): 9.12 (m, 4H), 8.68 (tt, *J* = 7.8, 1.4 Hz, 2H), 8.22-8.15 (m, 4H), 6.23 (s, 4H), 4.28 (t, *J*=6.8 Hz, 4H), 1.70-1.60 (m, 4H), 1.39-1.24 (m, 36H), 0.90 (t, *J*=7.0 Hz, 6H) ppm. ^{13}C-NMR (101 MHz, CD_3OD, δ): 163.9, 148.0, 146.2, 140.8, 136.2, 129.7, 67.9, 57.4, 33.1, 30.81, 30.78, 30.75, 30.70, 30.50, 30.44, 29.6, 27.0, 23.8, 14.5 ppm. Anal. calcd for $C_{42}H_{64}Br_2N_2O_4S$: C, 59.15; H, 7.56; N, 3.28; S, 3.76; found: C, 58.95; H, 7.75; N, 3.20; S, 3.59.

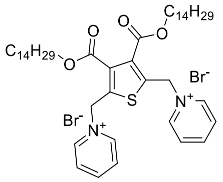

1,1'-((3,4-Bis((tetradecyloxy)carbonyl)thiophene-2,5-diyl)bis(methylene))bis(pyridin-1-ium) dibromide (3b): White solid, mp. 160.8-161.5 °C (acetone), yield: 24 %. ^{1}H-NMR (400 MHz, CD_3OD, δ): 9.14 (dd, *J*=6.8, 1.4 Hz, 4H), 8.68 (tt, *J*=7.7, 1.4 Hz, 2H), 8.21-8.14 (m, 4H), 6.24 (s, 4H), 4.28 (t, *J*=6.8 Hz, 4H), 1.70-1.60 (m, 4H), 1.38-1.24 (m, 44 H), 0.90 (t, *J*=7.0 Hz, 6H) ppm. ^{13}C-NMR (101 MHz, CD_3OD, δ): 163.9, 148.0, 146.2, 140.8, 136.2, 129.7, 67.9, 57.4, 33.1, 30.84, 30.83, 30.80,

30.77, 30.71, 30.51, 30.45, 29.6, 27.0, 23.8, 14.5 ppm. Anal. calcd for $C_{46}H_{72}Br_2N_2O_4S$ × $1.3H_2O$: C, 59.26; H, 8.06; N, 3.00; S, 3.44; found: C, 59.14; H, 7.95; N, 2.99; S, 3.35.

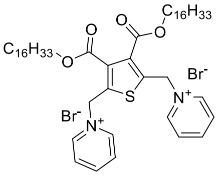

1,1'-((3,4-Bis((hexadecyloxy)carbonyl)thiophene-2,5-diyl)bis(methylene))bis(pyridin-1-ium) dibromide (3c): White solid, mp. 158.1-158.9 °C (acetone), yield: 26 %. ^{1}H-NMR (400 MHz, CD_3OD, δ): 9.15 (dd, *J*=6.8; 1.3 Hz, 4H), 8.68 (tt, *J*=7.8; 1.3 Hz, 2H), 8.21-8.15 (m, 4H), 6.26 (s, 4H), 4.27 (t, *J*=6.8 Hz, 4H), 1.68-1.60 (m, 4H), 1.38-1.23 (m, 52H), 0.90 (t, *J*=6.7 Hz, 6H) ppm. ^{13}C-NMR (101 MHz, CD_3OD, δ): 163.9, 148.0, 146.2, 140.7, 136.1, 129.7, 67.9, 57.4, 33.1, 30.83, 30.79, 30.77, 30.71, 30.50, 30.46, 29.6, 27.0, 23.8, 14.5 ppm. Anal. calcd for $C_{50}H_{80}Br_2N_2O_4S$ × $0.5H_2O$: C, 61.65; H, 8.38; N, 2.88; S, 3.29; found: C, 61.58; H, 8.49; N, 2.86; S, 3.05.

S2.2. Symmetric furans 4a-c

<u>*General procedure for transesterification of furan derivatives:*</u>

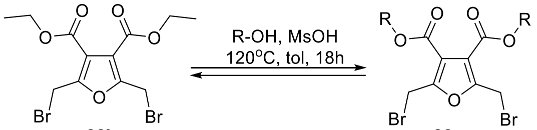

22' **22a-c**

Diethyl 2,5-bis(bromomethyl)furan-3,4-dicarboxylate **22'** (1.0 eq) was dissolved in anhydrous toluene. The appropriate alcohol (3.0 eq) and methanesulfonic acid (0.2 eq) were then added, and the reaction mixture was stirred at 120 °C for 18 h. The solvent was evaporated and the crude product was purified with column chromatography (gradient: 0 to 30 % EtOAc in PE) and crystallized from MeCN.

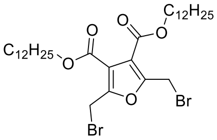

Didodecyl 2,5-bis(bromomethyl)furan-3,4-dicarboxylate (22a): White solid, mp. 36.5-37.3 °C (MeCN), yield: 52 %. ^{1}H-NMR (400 MHz, $CDCl_3$, δ): 4.64 (s, 4H), 4.28 (t, *J*=6.8 Hz, 4H), 1.76-1.67 (m, 4H), 1.44-1.22 (m, 36H), 0.88 (t, *J*=7.0 Hz, 6H) ppm. ^{13}C-NMR (101 MHz, $CDCl_3$, δ): 161.8, 154.2, 117.2, 65.9, 32.1, 29.81, 29.79, 29.74, 29.69, 29.5, 29.4, 28.7, 26.1, 22.8, 20.2, 14.3 ppm. Anal. calcd for $C_{32}H_{54}Br_2O_5$: C, 56.64; H, 8.02; found: C, 57.14; H, 8.19.

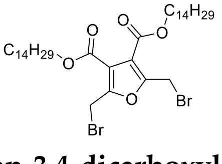

Ditetradecyl 2,5-bis(bromomethyl)furan-3,4-dicarboxylate (22b): White solid, mp. 50.0-50.6 °C (MeCN), yield: 50 %. ^{1}H-NMR (400 MHz, $CDCl_3$, δ): 4.64 (s, 4H), 4.28 (t, *J*=6.8 Hz, 4H), 1.75-1.68

(m, 4H), 1.44-1.20 (m, 44H), 0.88 (t, *J*=7.0 Hz, 6H) ppm. ^{13}C-NMR (101 MHz, $CDCl_3$, δ): 161.8, 154.2, 117.2, 65.9, 32.1, 29.85, 29.83, 29.81, 29.75, 29.69, 29.5, 29.4, 28.7, 26.1, 22.8, 20.2, 14.3 ppm. Anal. calcd for $C_{36}H_{62}Br_2O_5$: C, 58.85; H, 8.51; found: C, 59.33; H, 8.61.

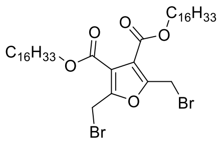

Dihexadecyl 2,5-bis(bromomethyl)furan-3,4-dicarboxylate (22c): White solid, mp. 56.6-57.4 °C (MeCN), yield: 52 %; ^{1}H-NMR (400 MHz, $CDCl_3$, δ): 4.64 (s, 4H), 4.27 (t, *J*=6.7 Hz, 4H), 1.76-1.66 (m, 4H), 1.43-1.19 (m, 52H), 0.87 (t, *J*=7.0 Hz, 6H) ppm. ^{13}C-NMR (101 MHz, $CDCl_3$, δ): 161.8, 154.2, 117.2, 65.9, 32.1, 29.86, 29.81, 29.75, 29.69, 29.5, 29.4, 28.7, 26.1, 22.8, 20.2, 14.3 ppm. Anal. calcd for $C_{40}H_{70}Br_2O_5$: C, 60.75; H, 8.92; found: C, 61.25; H, 9.16.

General procedure for bromine nucleophilic substitution in furan derivatives:

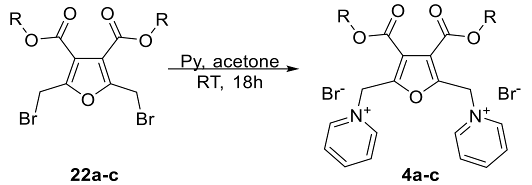

22a-c **4a-c**

Dialkyl 2,5-bis(bromomethyl)furan-3,4-dicarboxylate **22a-c** (1.0 eq) was dissolved in dry acetone and then pyridine (3.0 eq) was added. The reaction mixture was stirred at room temperature for 18 h. The resulting suspension was filtered, and the precipitate was crystallized from acetone to yield the pure product **4a-c**.

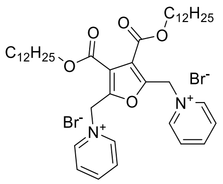

1,1'-((3,4-Bis((dodecyloxy)carbonyl)furan-2,5-diyl)bis(methylene))bis(pyridin-1-ium) dibromide (4a): Light-yellow solid, mp. 180.9-181.6 °C (acetone), yield: 46 %. ^{1}H-NMR (400 MHz, CD_3OD, δ): 9.18 (dd, *J*=6.9, 1.3 Hz, 4H), 8.68 (tt, *J*=7.8, 1.3 Hz, 2H), 8.22-8.17 (m, 4H), 6.14 (s, 4H), 4.34 (t, *J*=6.8 Hz, 4H), 1.77-1.68 (m, 4H), 1.42-1.26 (m, 36 H), 0.90 (t, *J*=7.0 Hz, 6H) ppm. ^{13}C-NMR (101 MHz, CD_3OD, δ): 162.9, 152.5, 148.1, 146.6, 129.8, 120.9, 67.6, 56.5, 33.1, 30.83, 30.79, 30.77, 30.74, 30.51, 30.47, 29.7, 27.1, 23.8, 14.5 ppm. Anal. calcd for $C_{42}H_{64}Br_2N_2O_5 \times H_2O$: C, 59.01; H, 7.78; N, 3.28; found: C, 58.46; H, 7.64; N, 3.26.

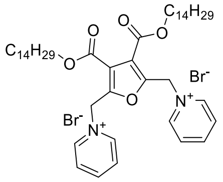

1,1'-((3,4-Bis((tetradecyloxy)carbonyl)furan-2,5-diyl)bis(methylene))bis(pyridin-1-ium) dibromide (4b): Light-yellow solid, mp. 178.7-179.3 °C (acetone), yield: 49 %. ^{1}H-NMR (400 MHz, CD_3OD, δ): 9.19 (dd, *J*=6.6, 1.4 Hz, 4H), 8.68 (tt, *J*=7.9, 1.4 Hz, 2H), 8.20 (dd, *J*=7.9, 6.6 Hz, 4H), 6.15 (s, 4H), 4.34 (t, *J*=6.8 Hz, 4H), 1.76-1.69 (m, 4H), 1.41-1.26 (m, 44 H), 0.90 (t, *J*=7.0 Hz, 6H) ppm. ^{13}C-NMR (101 MHz, CD_3OD, δ): 162.9, 152.5, 148.1, 146.6, 129.8, 120.9, 67.6, 56.5, 33.1, 30.84, 30.83, 30.80, 30.79, 30.74, 30.51, 30.48, 29.7, 27.1, 23.8, 14.5 ppm. Anal. calcd for $C_{46}H_{72}Br_2N_2O_5 \times 0.7H_2O$: C, 61.02; H, 8.17; N, 3.09; found: C, 60.60; H, 8.10; N, 3.00.

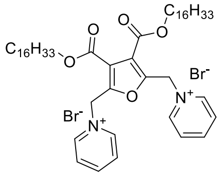

1,1'-((3,4-Bis((hexadecyloxy)carbonyl)furan-2,5-diyl)bis(methylene))bis(pyridin-1-ium) dibromide (4c): White solid, mp. 175.8-176.6 °C (acetone), yield: 50 %. ^{1}H-NMR (400 MHz, CD_3OD, δ): 9.18 (dd, *J*=6.7, 1.5 Hz, 4H), 8.68 (tt, *J*=7.8, 1.5 Hz, 2H), 8.20 (dd, *J*=7.8, 6.7 Hz, 4H), 6.15 (s, 4H), 4.34 (t, *J*=6.8 Hz, 4H), 1.77-1.68 (m, 4H), 1.44-1.23 (m, 52H), 0.90 (t, *J*=7.0 Hz, 6H) ppm. ^{13}C-NMR (101 MHz, CD_3OD, δ): 162.9, 152.5, 148.1, 146.6, 129.8, 120.9, 67.6, 56.5, 33.1, 30.84, 30.79, 30.76, 30.51, 30.49, 29.7, 27.1, 23.8, 14.5 ppm. Anal. calcd for $C_{50}H_{80}Br_2N_2O_5 \times 0.9H_2O$: C, 62.22; H, 8.54; N, 2.90; found: C, 61.86; H,8.40; N, 2.76.

S2.3. Symmetric pyrroles 5a-c

<u>*Modified Paal-Knorr pyrrole cyclization:*</u>

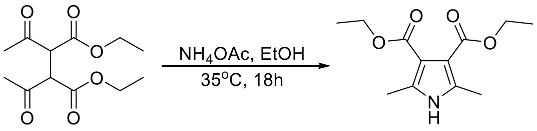

Diethyl 2,5-dimethyl-1*H*-pyrrole-3,4-dicarboxylate (17): Diethyl 2,3-diacetylsuccinate **15** (1.0 g, 1.0 eq) and NH_4OAc (1.3 eq) were suspended in 10 mL EtOH and heated at 35 °C. After 18 h, the solvent was evaporated and light-yellow precipitate was washed with H_2O (2 × 5 mL) to yield pure product **17** as white solid. Yield 0.89 g, 96 %. ^{1}H-NMR (400 MHz, $CDCl_3$, δ): 8.17 (br. s, 1H), 4.26 (q, *J*=7.1 Hz, 4H), 2.35 (s, 6H), 1.31 (t, *J*=7.1 Hz, 6H) ppm, spectrum is in agreement with published data [36].

General procedure for transesterification of pyrrole derivatives:

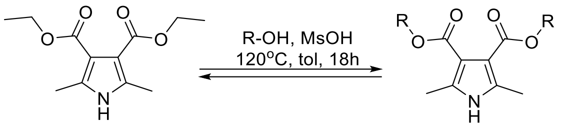

17 **20a-c**

Diethyl 2,5-dimethyl-1*H*-pyrrole-3,4-dicarboxylate **17** (1.0 eq) was dissolved in anhydrous toluene. Then appropriate alcohol (3.0 eq) and methanesulfonic acid (0.2 eq) were added. Reaction mixture was stirred at 120 °C for 18 h after which the solvent was evaporated and the crude product was purified with column chromatography (gradient: 0 to 30 % EtOAc in PE) and crystallized from MeCN.

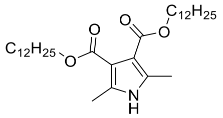

Didodecyl 2,5-dimethyl-1*H*-pyrrole-3,4-dicarboxylate (20a): Light yellow solid, mp. 45.3-46.1 °C (MeCN), yield: 32 %. ^{1}H-NMR (400 MHz, $CDCl_3$, δ): 8.07-7.80 (br. s 1H), 4.20 (t, *J*=6.8 Hz, 4H), 2.38 (s, 6H), 1.73-1.62 (m, 4H), 1.42-1.20 (m, 36H), 0.87 (t, *J*=7.0 Hz, 6H) ppm. ^{13}C-NMR (101 MHz, $CDCl_3$, δ): 165.6, 131.9, 113.0, 65.5, 32.1, 29.82, 29.79, 29.77, 29.73, 29.53, 29.50, 29.0, 26.2, 22.8, 14.2, 12.6 ppm. HRMS TOF ES+ of $C_{32}H_{58}NO_4$ $[M+H]^+$ (m/z) 520,4364; calcd: 520,4366.

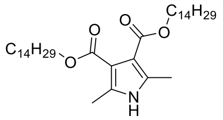

Ditetradecyl 2,5-dimethyl-1*H*-pyrrole-3,4-dicarboxylate (20b): White solid, mp. 56.8-57.6 °C (MeCN), yield: 41 %. ^{1}H-NMR (400 MHz, $CDCl_3$, δ): 8.09 (br. s, 1H), 4.19 (t, *J*=6.8 Hz, 4H), 2.36 (s, 6H), 1.72-1.63 (m, 4H), 1.43-1.20 (m, 44H), 0.87 (t, *J*=6.7 Hz, 6H) ppm. ^{13}C-NMR (101 MHz, $CDCl_3$, δ): 165.6, 132.0, 112.9, 64.6, 32.1, 29.85, 29.84, 29.82, 29.81, 29.78, 29.73, 29.53, 29.53, 29.52, 28.95, 26.2, 22.8, 14.3, 12.6 ppm. HRMS TOF ES+ of $C_{36}H_{65}NO_4Na$ $[M+Na]^+$ (m/z) 598,4828; calcd: 598,4811.

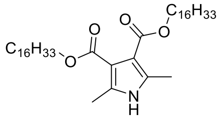

Dihexadecyl 2,5-dimethyl-1*H*-pyrrole-3,4-dicarboxylate (20c): Light-grey solid, mp. 62.3-63.0 °C (MeCN), yield: 33 %. ^{1}H-NMR (400 MHz, $CDCl_3$, δ): 8.14-7.93 (br. s, 1H), 4.19 (t, *J*=6.8 Hz, 4H), 2.36 (s, 6H), 1.73-1.60 (m, 4H), 1.42-1.19 (m, 52H), 0.88 (t, *J*=7.0 Hz, 6H) ppm. ^{13}C-NMR (101 MHz, $CDCl_3$, δ): 165.7, 132.0, 112.9, 64.6, 32.1, 29.86, 29.83, 29.78, 29.74, 29.5, 29.0, 26.2, 22.8, 14.3, 12.6 ppm. HRMS TOF ES- of $C_{40}H_{72}NO_4$ $[M-H]^-$ (m/z) 630,5466; calcd: 630,5461.

General procedure for N-Boc protection:

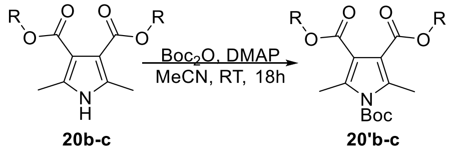

Dialkyl 2,5-dimethyl-1*H*-pyrrole-3,4-dicarboxylate **20b-c** (1.0 eq) was dissolved in anhydrous MeCN, after which Boc_2O (2.0 eq) and catalytic amount of DMAP (0.1 eq) were added. The reaction mixture was stirred at room temperature for 18 h. The reaction mixture was quenched with 10 mL H_2O and extracted with DCM (3 × 5 mL). The organic layers were combined, dried over Na_2SO_4, filtered and evaporated. The crude product was purified with column chromatography (eluent 0 to 30 % EtOAc in PE).

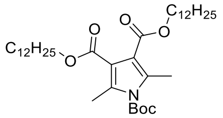

1-(Tert-butyl) 3,4-didodecyl 2,5-dimethyl-1*H*-pyrrole-1,3,4-tricarboxylate (20′a): Light yellow oil, yield: 69 %. ^{1}H-NMR (400 MHz, $CDCl_3$, δ): 4.18 (t, *J*=6.8 Hz, 4H), 2.50 (s, 6H), 1.71-1.63 (m, 4H), 1.60 (s, 9H), 1.41-1.20 (m, 36H), 0.87 (t, *J*=7.0 Hz, 6H) ppm. ^{13}C-NMR (101 MHz, $CDCl_3$, δ): 165.3, 149.4, 134.4, 115.7, 85.6, 65.0, 32.1, 29.81, 29.79, 29.75, 29.71, 29.50, 29.49, 28.8, 28.1, 26.2, 22.8, 14.3, 13.8 ppm. HRMS TOF ES+ of $C_{37}H_{65}NO_6Na$ $[M+Na]^+$ (m/z) 642,4730; calcd: 642,4710.

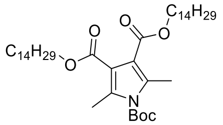

1-(Tert-butyl) 3,4-ditetradecyl 2,5-dimethyl-1*H*-pyrrole-1,3,4-tricarboxylate (20′b): Light yellow solid, yield: 75 %. ^{1}H-NMR (400 MHz, $CDCl_3$, δ): 4.19 (t, *J*=6.8 Hz, 4H), 2.50 (s, 6H), 1.71-1.61 (m, 4H), 1.60 (s, 9H), 1.40-1.22 (m, 44H), 0.88 (t, *J*=7.0 Hz, 6H) ppm. ^{13}C-NMR (101 MHz, $CDCl_3$, δ): 165.3, 149.4, 134.4, 115.7, 85.6, 65.0, 32.1, 29.85, 29.84, 29.82, 29.77, 29.72, 29.52, 29.50, 28.8, 28.1, 26.2, 22.8, 14.3, 13.8 ppm. HRMS TOF ES+ of $C_{41}H_{73}NO_6Na$ $[M+Na]^+$ (m/z) 698.5320; calcd: 698.5336.

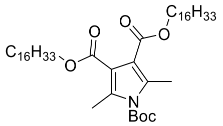

1-(Tert-butyl) 3,4-dihexadecyl 2,5-dimethyl-1*H*-pyrrole-1,3,4-tricarboxylate (20′c): Light-yellow solid, yield: 56 %. ^{1}H-NMR (400 MHz, $CDCl_3$, δ): 4.19 (t, *J*=6.8 Hz, 4H), 2.50 (s, 6H), 1.72-1.63 (m, 4H), 1.60 (s, 9H), 1.41-1.20 (m, 52H), 0.87 (t, *J*=7.0 Hz, 6H) ppm. ^{13}C-NMR (101 MHz, $CDCl_3$, δ): 165.3, 149.4, 134.5, 115.7, 85.6, 65.0, 32.1, 29.86, 29.82, 29.77, 29.72, 29.52, 29.50, 28.8, 28.1, 26.2, 22.8, 14.3, 13.8 ppm. HRMS TOF ES+ of $C_{45}H_{81}NO_6Na$ $[M+Na]^+$ (m/z) 754.5975; calcd: 754.5962.

General procedure for the bromination of methyl groups in pyrrole derivatives:

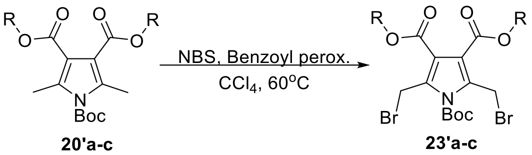

1-(Tert-butyl) 3,4-dialkyl 2,5-dimethyl-1*H*-pyrrole-1,3,4-tricarboxylate **20'a-c** (1.0 eq), NBS (2.1 eq) and benzoyl peroxide (0.2 eq) were suspended in CCl_4 and stirred at 60 °C for 18 h. Followed by quenching with H_2O and extraction with DCM. The crude product was purified with column chromatography (gradient: 0 to 10 % EtOAc in PE).

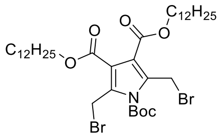

1-(*tert*-Butyl) 3,4-didodecyl 2,5-bis(bromomethyl)-1*H*-pyrrole-1,3,4-tricarboxylate (23'a): Light yellow oil, yield: 41 %. ^{1}H-NMR (400 MHz, $CDCl_3$, δ): 5.00 (s, 4H), 4.25 (t, *J*=6.8 Hz, 4H), 1.75-1.63 (m, 13H), 1.42-1.20 (m, 36H), 0.87 (t, *J*=7.0 Hz, 6H) ppm. ^{13}C-NMR (101 MHz, $CDCl_3$, δ): 163.6, 147.6, 134.8, 118.2, 88.2, 65.8, 32.1, 29.80, 29.78, 29.74, 29.69, 29.50, 29.45, 28.7, 27.8, 26.1, 22.8, 21.9, 14.3 ppm. HRMS TOF ES+ of $C_{37}H_{63}NO_6Br_2Na$ $[M+Na]^+$ (m/z) 798,2942; calcd: 798,2920.

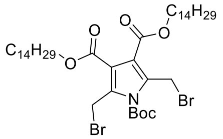

1-(*tert*-Butyl) 3,4-ditetradecyl 2,5-bis(bromomethyl)-1*H*-pyrrole-1,3,4-tricarboxylate (23'b): Light-grey solid, yield: 39 %. ^{1}H-NMR (400 MHz, $CDCl_3$, δ): 5.00 (s, 4H), 4.25 (t, *J*=6.8 Hz, 4H), 1.75-1.63 (m, 13H), 1.43-1.19 (m, 44H), 0.88 (t, *J*=7.0 Hz, 6H) ppm. ^{13}C-NMR (101 MHz, $CDCl_3$, δ): 163.6, 147.6, 134.8, 118.2, 88.2, 65.8, 32.1, 29.85, 29.84, 29.81, 29.76, 29.70, 29.52, 29.46, 28.75, 27.78, 26.1, 22.8, 21.9, 14.3 ppm. HRMS TOF ES+ of $C_{41}H_{71}NO_6Br_2Na$ $[M+Na]^+$ (m/z) 854.3542; calcd: 854.3546.

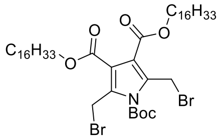

1-(*tert*-Butyl) 3,4-dihexadecyl 2,5-bis(bromomethyl)-1*H*-pyrrole-1,3,4-tricarboxylate (23'c): Light-grey solid, yield: 34 %. ^{1}H-NMR (400 MHz, $CDCl_3$, δ): 5.00 (s, 4H), 4.25 (t, *J*=6.8 Hz, 4H), 1.74-1.64 (m, 13H), 1.43-1.21 (m, 52 H), 0.87 (t, *J*=7.0 Hz, 6H) ppm. ^{13}C-NMR (101 MHz, $CDCl_3$, δ): 163.6, 147.6, 134.8, 118.2, 88.2, 65.7, 32.1, 29.85, 29.84, 29.81, 29.75, 29.70, 29.51, 29.45, 28.7, 27.8, 26.1, 22.8, 21.9, 14.3 ppm. Anal. calcd for $C_{45}H_{79}Br_2NO_6$: C, 60.73; H, 8.95; N, 1.57; found: C, 60.66; H, 8.97; N, 1.53.

General procedure for N-Boc deprotection:

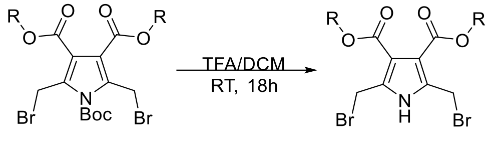

N-Boc protected derivative **23’a-c** was dissolved in DCM/TFA solution (2:1) and stirred at room temperature for 18 h before evaporation. Precipitate was crystallized from MeCN to yield pure product **23a-c**.

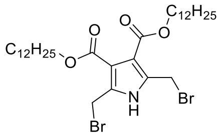

Didodecyl 2,5-bis(bromomethyl)-1*H*-pyrrole-3,4-dicarboxylate (23a): White solid, mp. 69.4-70.6 °C (MeCN), yield: 70 %. ^{1}H-NMR (400 MHz, $CDCl_3$, δ): 8.82 (br. s, 1H), 4.70 (s, 4H), 4.24 (t, *J*=6.8 Hz, 4H), 1.76-1.64 (m, 4H), 1.42-1.18 (m, 36H), 0.87 (t, *J*=7.0 Hz, 6H) ppm. ^{13}C-NMR (101 MHz, $CDCl_3$, δ): 163.9, 132.1, 115.5, 65.4, 32.1, 29.81, 29.79, 29.76, 29.51, 29.48, 28.8, 26.2, 22.8, 22.4, 14.3 ppm. Anal. calcd for $C_{32}H_{55}Br_2NO_4$: C, 56.72; H, 8.18; N, 2.07; found: C, 56.23; H, 8.37; N, 2.05

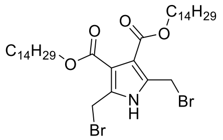

Ditetradecyl 2,5-bis(bromomethyl)-1*H*-pyrrole-3,4-dicarboxylate (23b): Light brown solid, mp. 72.3-73.5 °C (MeCN), yield: 82 %. ^{1}H-NMR (400 MHz, $CDCl_3$, δ): 8.84 (br. s, 1H), 4.70 (s, 4H), 4.24 (t, *J*=6.8 Hz, 4H), 1.76-1.62 (m, 4H), 1.45-1.19 (m, 44H), 0.87 (t, *J*=7.0 Hz, 6H) ppm. ^{13}C-NMR (101 MHz, $CDCl_3$, δ): 163.9, 132.1, 115.5, 65.4, 32.1, 29.86, 29.84, 29.82, 29.77, 29.72, 29.52, 29.49, 28.8, 26.2, 22.8, 22.4, 14.3 ppm. Anal. calcd for $C_{36}H_{63}Br_2NO_4$: C, 58.93; H, 8.66; N, 1.91; found: C, 59.41; H, 8.77; N, 1.95.

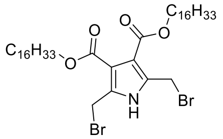

Dihexadecyl 2,5-bis(bromomethyl)-1*H*-pyrrole-3,4-dicarboxylate (23c): Light brown solid, mp. 75.7-77.0 °C (MeCN), yield: 88 %. ^{1}H-NMR (400 MHz, $CDCl_3$, δ): 8.74 (br. s, 1H), 4.70 (s, 4H), 4.24 (t, *J*=6.8 Hz, 4H), 1.76-1.65 (m, 4H), 1.43-1.20 (m, 52H), 0.87 (t, *J*=7.0 Hz, 6H) ppm. ^{13}C-NMR (101 MHz, $CDCl_3$, δ): 163.9, 132.1, 115.5, 65.4, 32.1, 29.86, 29.82, 29.77, 29.72, 29.52, 29.49, 28.8, 26.2, 22.9, 22.4, 14.3 ppm. Anal. calcd for $C_{40}H_{71}Br_2NO_4$: C, 60.81; H, 9.06; N, 1.77; found: C, 61.12; H, 9.16, N, 1.75.

General procedure for bromine nucleophilic substitution of pyrrole derivatives:

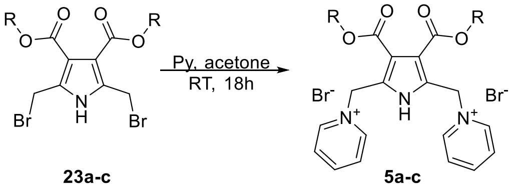

Dialkyl 2,5-bis(bromomethyl)-1*H*-pyrrole-3,4-dicarboxylate **23a-c** (1.0 eq) was dissolved in dry acetone after which pyridine (3.0 eq) was added. The reaction mixture was stirred at room temperature for 18 h. The resulting suspension was filtered and precipitate was crystallized from acetone to yield pure product **5a-c**.

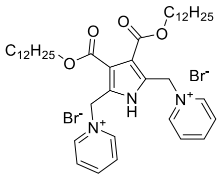

1,1'-((3,4-Bis((dodecyloxy)carbonyl)-1H-pyrrole-2,5-diyl)bis(methylene))bis(pyridin-1-ium) dibromide (5a): White solid, mp. 135.2-135.7 °C (acetone), yield: 51 %. ^{1}H-NMR (400 MHz, $CDCl_3$, δ): 14.07 (br. s, 1H), 9.61 (d, *J*=6.2 Hz, 4H), 8.60 (t, *J*=7.7 Hz, 2H), 8.18 (dd, *J*=7.7, 6.2 Hz, 4H), 6.19 (s,4H), 4.25 (t, *J*=7.0 Hz, 4H), 1.67 (qv, *J*=7.0 Hz, 4H), 1.42-1.16 (m, 36H), 0.87 (t, *J*=7.0 Hz, 6H) ppm. ^{13}C-NMR (101 MHz, $CDCl_3$, δ): 164.5, 146.4, 145.6, 128.93, 128.90, 117.0, 66.1, 55.6, 32.1, 29.81, 29.79, 29.77, 29.72, 29.5, 28.8, 26.1, 22.8, 14.3 ppm. Anal. calcd for $C_{42}H_{65}Br_2N_3O_4$: C, 60.36; H, 7.84; N, 5.03; found: C, 59.97; H, 7.88, N, 4.79.

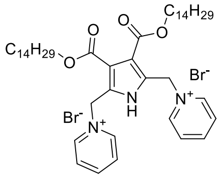

1,1'-((3,4-Bis((tetradecyloxy)carbonyl)-1*H*-pyrrole-2,5-diyl)bis(methylene))bis(pyridin-1-ium) dibromide (5b): White solid, mp. 143.7-144.5 °C (acetone), yield: 64 %. ^{1}H-NMR (400 MHz, $CDCl_3$, δ): 14.07 (s, 1H), 9.62 (d, *J*= 7.1, Hz, 4H), 8.61 (t, *J*=7.7 Hz, 2H), 8.18 (dd, *J*=7.7, 7.1 Hz, 4H), 6.20 (s, 4H), 4.24 (t, *J*=7.1 Hz, 4H), 1.66 (qv, *J*=7.1 Hz, 4H), 1.40-1.17 (m, 44H), 0.86 (t, *J*=7.0 Hz, 6H) ppm. ^{13}C-NMR (101 MHz, $CDCl_3$, δ): 164.5, 146.5, 145.5, 129.04, 128.95, 117.2, 66.1, 55.7, 32.1, 29.84, 29.82, 29.80, 29.73, 29.5, 28.8, 26.1, 22.8, 14.3 ppm. Anal. calcd for $C_{46}H_{73}Br_2N_3O_4$: C, 61.95; H, 8.25; N, 4.71; found: C, 61.54; H, 8.25, N, 4.55.

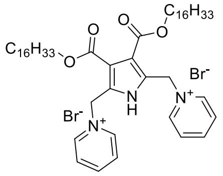

1,1'-((3,4-Bis((hexadecyloxy)carbonyl)-1*H*-pyrrole-2,5-diyl)bis(methylene))bis(pyridin-1-ium) dibromide (5c): White solid, mp. 147.8-148.3 °C (acetone), yield: 75 %. ^{1}H-NMR (400 MHz, $CDCl_3$, δ): 14.05 (s, 1H), 9.61 (d, *J*=6.3 Hz, 4H), 8.62 (t, *J*=7.7 Hz, 2H), 8.18 (dd, *J*=7.7, 6.3 Hz, 4H), 6.19 (s, 4H), 4.24 (t, *J*=6.8 Hz, 4H), 1.66 (qv, *J*=7.1 Hz, 4H), 1.46-1.17 (m, 52H), 0.86 (t, *J*=7.0 Hz, 6H) ppm. ^{13}C-NMR (101 MHz, $CDCl_3$, δ): 164.4, 146.4, 145.5, 128.91, 128.86, 117.0, 66.1, 55.3, 32.0, 29.84, 29.81, 29.78, 29.72, 29.5, 28.8, 26.1, 22.8, 14.3 ppm. Anal. calcd for $C_{50}H_{81}Br_2N_3O_4$: C, 53.35; H, 8.61; N, 4.43; found: C, 62.86; H, 8.66, N, 4.22.

S2.4. Unsymmetric pyrroles 7a-c

<u>*General procedure for transesterification of unsymmetric pyrrole derivatives:*</u>

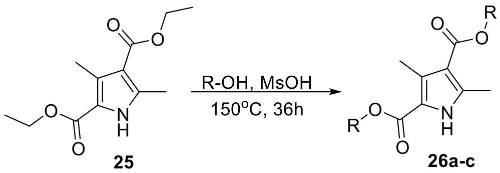

Diethyl 3,5-dimethyl-1*H*-pyrrole-2,4-dicarboxylate **25** (1.0 eq) was placed in round bottom flask. Then the appropriate alcohol (3.0 eq) and methanesulfonic acid (0.1 eq) were added, and the reaction mixture was stirred at 150 °C for 36 h. The solvent was evaporated and the crude product was purified with column chromatography (gradient: 0 to 30 % EtOAc in PE) and crystallized from MeCN.

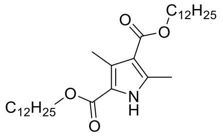

Didodecyl 3,5-dimethyl-1*H*-pyrrole-2,4-dicarboxylate (26a): Light brown solid, mp. 59.9-60.7 °C (MeCN), yield: 43 %. ^{1}H-NMR (400 MHz, $CDCl_3$, δ): 8.85 (br. s, 1H), 4.26 (t, *J*=6.6 Hz, 2H)4.23 (t, *J*=6.6 Hz, 2H), 2.56 (s, 3H), 2.53 (s, 3H), 1.78-1.66 (m, 4H), 1.47-1.18 (m, 36H), 0.87 (t, *J*=6.9 Hz, 6H) ppm. ^{13}C-NMR (101 MHz, $CDCl_3$, δ): 165.7, 161.9, 138.9, 130.9, 118.2, 113.9, 64.6, 63.9, 32.1, 29.78, 29.72, 29.68, 29.66, 29.49, 29.42, 29.40, 28.97, 28.96, 26.3, 26.2, 22.8, 14.6, 14.3, 12.2 ppm. Anal. calcd for $C_{32}H_{57}NO_4$: C, 73.94; H, 11.05; N, 2.69; found: C, 74.14; H, 11.06, N, 2.69.

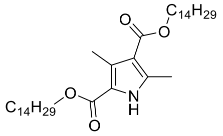

Ditetradecyl 3,5-dimethyl-1*H*-pyrrole-2,4-dicarboxylate (26b): Light brown solid, mp. 66.6-67.2 °C (MeCN), yield: 51 %. ^{1}H-NMR (400 MHz, $CDCl_3$, δ): 8.86 (br. s, 1H), 4.26 (t, *J*=6.7 Hz, 2H)4.22

(t, J=6.6 Hz, 2H), 2.56 (s, 3H), 2.51 (s, 3H), 1.80-1.67 (m, 4H), 1.47-1.16 (m, 44H), 0.88 (t, J=6.9 Hz, 6H) ppm. ^{13}C-NMR (101 MHz, $CDCl_3$, δ): 165.7, 161.9, 138.9, 130.9, 118.2, 113.9, 64.7, 63.9, 32.1, 29.84, 29.82, 29.80, 29.73, 29.69, 29.67, 29.51, 29.43, 29.40, 28.98, 28.96, 26.4, 26.2, 22.8, 14.6, 14.3, 12.2 ppm. Anal. calcd for $C_{36}H_{65}NO_4$: C, 75.08; H, 11.38; N, 2.43; found: C, 75.06; H, 11.37, N, 2.43.

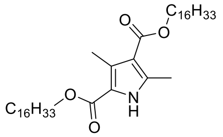

Dihexadecyl 3,5-dimethyl-1*H*-pyrrole-2,4-dicarboxylate (26c): White solid, mp. 70.1-70.5 °C (MeCN), yield: 36 %. ^{1}H-NMR (400 MHz, $CDCl_3$, δ): 8.88 (s, 1H), 4.26 (t, J=6.7 Hz, 2H), 4.22 (t, J=6.6 Hz, 2H), 2.56 (s, 3H), 2.51 (s, 3H), 1.77-1.66 (m, 4H), 1.45-1.19 (m, 52H), 0.88 (t, J=6.9 Hz, 6H) ppm. ^{13}C-NMR (101 MHz, $CDCl_3$, δ): 165.7, 161.9, 138.9, 130.9, 118.2, 113.9, 64.7, 63.9, 32.1, 29.85, 29.81, 29.73, 29.70, 29.67, 29.52, 29.43, 29.41, 28.98, 28.96, 26.4, 26.2, 22.9, 14.6, 14.3, 12.2 ppm. Anal. calcd for $C_{40}H_{73}NO_4$: C, 76.02; H, 11.64; N, 2.22; found: C, 76.39; H, 11.71, N, 2.06.

General procedure for N-Boc protection of unsymmetric pyrroles:

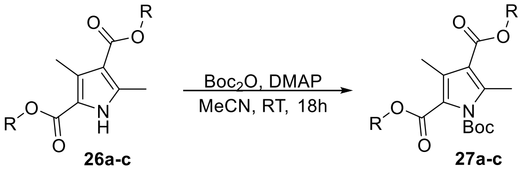

Dialkyl 3,5-dimethyl-1*H*-pyrrole-2,4-dicarboxylate **26a-c** (1.0 eq) was suspended in anhydrous MeCN, after which Boc_2O (2.0 eq) and catalytic amount of DMAP (0.1 eq) were added. The reaction mixture was stirred at room temperature for 18 h. The reaction mixture was quenched with 10 mL H_2O and extracted with DCM (3 × 5 mL). Organic layers were combined, dried over Na_2SO_4, filtered and evaporated. The crude product was purified with column chromatography (eluent 0 to 20 % EtOAc in PE).

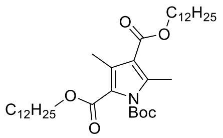

1-(*tert*-Butyl) 2,4-didodecyl 3,5-dimethyl-1*H*-pyrrole-1,2,4-tricarboxylate (27a): Colourless oil, yield: 90 %. ^{1}H-NMR (400 MHz, $CDCl_3$, δ): 4.25 (t, J=6.8 Hz, 2H), 4.23 (t, J=6.7 Hz, 2H), 2.59 (s, 3H), 2.45 (s, 3H), 1.76-1.67 (m, 4H), 1.58 (s, 9H), 1.44-1.20 (m, 36H), 0.88 (t, J=6.9 Hz, 6H) ppm. ^{13}C-NMR (101 MHz, $CDCl_3$, δ): 165.3, 161.6, 149.4, 140.7, 130.3, 121.2, 114.6, 85.8, 65.1, 64.3, 32.1, 29.78, 29.71, 29.67, 29.64, 29.49, 29.40, 29.38, 28.90, 28.88, 27.7, 26.3, 26.1, 22.8, 14.3, 12.8, 12.2 ppm.

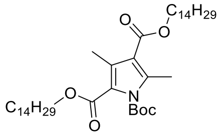

1-(*tert*-Butyl) 2,4-ditetradecyl 3,5-dimethyl-1*H*-pyrrole-1,2,4-tricarboxylate (27b): Yellow oil, yield: 95 %. ^{1}H-NMR (400 MHz, $CDCl_3$, δ): 4.25 (t, *J*=6.8 Hz, 2H), 4.23 (t, *J*=6.7 Hz, 2H), 2.59 (s, 3H), 2.45 (s, 3H), 1.76-1.67 (m, 4H), 1.58 (s, 9H), 1.43-1.21 (m, 44H), 0.88 (t, *J*=6.9 Hz, 6H) ppm. ^{13}C-NMR (101 MHz, $CDCl_3$, δ): 165.3, 161.6, 149.4, 140.7, 130.3, 121.2, 114.6, 85.8, 65.1, 64.3, 32.1, 29.78, 29.71, 29.67, 29.64, 29.49, 29.40, 29.38, 28.90, 28.88, 27.7, 26.3, 26.1, 22.8, 14.3, 12.8, 12.2 ppm.

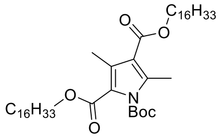

1-(*tert*-Butyl) 2,4-dihexadecyl 3,5-dimethyl-1*H*-pyrrole-1,2,4-tricarboxylate (27c): Light brown solid, yield: 75 %. ^{1}H-NMR (400 MHz, $CDCl_3$, δ): 4.25 (t, *J*=6.8 Hz, 2H), 4.23 (t, *J*=6.7 Hz, 2H), 2.59 (s, 3H), 2.45 (s, 3H), 1.76-1.67 (m, 4H), 1.58 (s, 9H), 1.44-1.20 (m, 52H), 0.88 (t, *J*=6.9 Hz, 6H) ppm. ^{13}C-NMR (101 MHz, $CDCl_3$, δ): 165.3, 161.6, 149.4, 140.7, 130.3, 121.3, 114.6, 85.8, 65.1, 64.3, 32.1, 29.85, 29.81, 29.79, 29.73, 29.68, 29.65, 29.51, 29.42, 29.39, 28.9, 27.7, 26.3, 26.1, 22.8, 14.3, 12.2 ppm.

General procedure for the bromination of methyl groups in unsymmetric pyrrole derivatives:

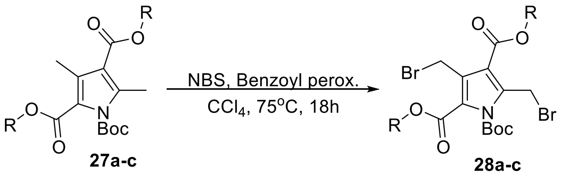

1-(*tert*-Butyl) 2,4-dialkyl 3,5-dimethyl-1*H*-pyrrole-1,2,4-tricarboxylate **27a-c** (1.0 eq), NBS (2.1 eq) and benzoyl peroxide (0.2 eq) were suspended in CCl_4 and stirred at 75 °C for 18 h. The reaction mixture was filtered, then filtrate was evaporated and the crude product was purified with column chromatography (gradient: 0 to 70 % DCM in PE).

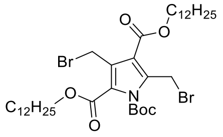

1-(*tert*-Butyl) 2,4-didodecyl 3,5-bis(bromomethyl)-1*H*-pyrrole-1,2,4-tricarboxylate (28a): Light yellow oil, yield: 68 %. ^{1}H-NMR (400 MHz, $CDCl_3$, δ): 5.08 (s, 2H), 4.92 (s, 2H), 4.36-4.31 (m, 4H), 1.84-1.73 (m, 4H), 1.63 (s, 9H), 1.50-1.18 (m, 36H), 0.88 (t, *J*=6.9 Hz, 6H) ppm. ^{13}C-NMR (101 MHz, $CDCl_3$, δ): 163.3, 160.2, 147.7, 138.2, 128.4, 124.7, 114.3, 87.7, 66.2, 65.4, 32.1, 29.77, 29.71, 29.66, 29.62, 29.49, 29.40, 29.36, 28.77, 28.75, 27.6, 26.3, 26.1, 22.8, 22.7, 20.3, 14.3 ppm.

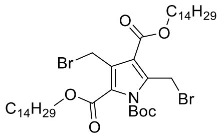

1-(*tert*-Butyl) 2,4-ditetradecyl 3,5-bis(bromomethyl)-1*H*-pyrrole-1,2,4-tricarboxylate (28b): Light gray solid, yield: 79 %. ^{1}H-NMR (400 MHz, $CDCl_3$, δ): 5.08 (s, 2H), 4.92 (s, 2H), 4.36-4.31 (m, 4H), 1.85-1.73 (m, 4H), 1.63 (s, 9H), 1.48-1.18 (m, 44H), 0.88 (t, *J*=6.8 Hz, 6H) ppm. ^{13}C-NMR (101 MHz, $CDCl_3$, δ): 163.3, 160.2, 147.7, 138.2, 128.5, 124.7, 114.3, 87.7, 66.2, 65.4, 32.1, 29.84, 29.80, 29.72, 29.67, 29.64, 29.5, 29.42, 29.37, 28.78, 28.76, 27.6, 26.3, 26.1, 22.8, 22.7, 20.3, 14.3 ppm.

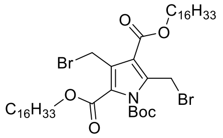

1-(*tert*-Butyl) 2,4-dihexadecyl 3,5-bis(bromomethyl)-1*H*-pyrrole-1,2,4-tricarboxylate (28c): White solid, yield: 61 %. ^{1}H-NMR (400 MHz, $CDCl_3$, δ): 5.08 (s, 2H), 4.92 (s, 2H), 4.36-4.31 (m, 4H), 1.85-1.73 (m, 4H), 1.62 (s, 9H), 1.50-1.20 (m, 52H), 0.88 (t, *J*=6.9 Hz, 6H) ppm. ^{13}C-NMR (101 MHz, $CDCl_3$, δ): 163.3, 160.2, 147.7, 138.1, 128.4, 124.7, 114.3, 87.7, 66.2, 65.4, 32.1, 29.84, 29.80, 29.78, 29.71, 29.66, 29.63, 29.5, 29.41, 29.36, 29.2, 28.77, 28.75, 27.7, 27.6, 26.3, 26.1, 22.8, 20.3, 14.3 ppm.

General procedure for N-Boc deprotection of unsymmetric pyrroles:

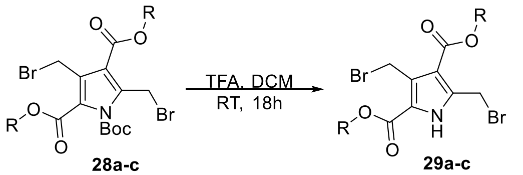

N-Boc protected derivative **28a-c** was dissolved in DCM/TFA solution (2:1) and stirred at room temperature for 18 h before evaporation. Precipitate was crystallized from MeCN to yield pure product **29a-c**.

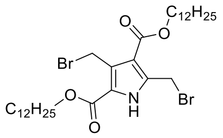

Didodecyl 3,5-bis(bromomethyl)-1*H*-pyrrole-2,4-dicarboxylate (29a): White solid, mp. 76.1-76.9 °C (MeCN), yield: 82 %. ^{1}H-NMR (400 MHz, $CDCl_3$, δ): 9.79 (br. s, 1H), 5.05 (s, 2H), 4.85 (s, 2H), 4.38 (t, *J*=6.8 Hz, 2H), 4.33z (t, *J*=6.7 Hz, 2H), 1.92-1.68 (m, 4H), 1.59-1.16 (m, 36H), 0.88 (t, *J*=6.8 Hz, 6H) ppm. ^{13}C-NMR (101 MHz, $CDCl_3$, δ): 163.8, 160.8, 136.6, 128.9, 121.0, 113.6, 66.0, 65.1, 32.1, 29.8, 29.72, 29.68, 29.65, 29.49, 29.43, 29.38, 28.8, 26.3, 26.2, 23.4, 22.8, 14.3 ppm. Anal. calcd for $C_{32}H_{55}NO_4Br_2$: C, 56.72; H, 8.18; N, 2.07; found: C, 56.44; H, 8.07, N, 2.00.

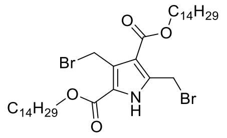

Ditetradecyl 3,5-bis(bromomethyl)-1*H*-pyrrole-2,4-dicarboxylate (29b): White solid, mp. 78.7-79.4 °C (MeCN), yield: 65 %. ^{1}H-NMR (400 MHz, $CDCl_3$, δ): 9.60 (br. s, 1H), 5.05 (s, 2H), 4.84 (s, 2H), 4.37 (t, *J*=6.8 Hz, 2H), 4.32 (t, *J*=6.7 Hz, 2H), 1.88-1.72 (m, 4H), 1.52-1.17 (m, 44H), 0.88 (t, *J*=6.9 Hz, 6H) ppm. ^{13}C-NMR (101 MHz, $CDCl_3$, δ): 163.8, 160.7, 136.5, 128.9, 121.0, 113.6, 65.9, 65.1, 32.1, 29.84, 29.83, 29.80, 29.73, 29.68, 29.66, 29.5, 29.43, 29.39, 28.8, 26.3, 26.2, 23.4, 22.8, 14.3 ppm. Anal. calcd for $C_{36}H_{63}NO_4Br_2 \times 0.8H_2O$: C, 57.80; H, 8.70; N, 1.87; found: C, 57.42; H, 8.44, N, 1.85.

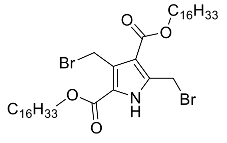

Dihexadecyl 3,5-bis(bromomethyl)-1*H*-pyrrole-2,4-dicarboxylate (29c): White solid, mp. 66.0-66.5 °C (MeCN), yield: 90 %. ^{1}H-NMR (400 MHz, $CDCl_3$, δ): 9.65 (br. s, 1H), 5.05 (s, 2H), 4.84 (s, 2H), 4.37 (t, *J*=6.8 Hz, 2H), 4.32 (t, *J*=6.7 Hz, 2H), 1.90-1.70 (m, 4H), 1.55-1.13 (m, 52H), 0.87 (t, *J*=6.9 Hz, 6H) ppm. ^{13}C-NMR (101 MHz, $CDCl_3$, δ): 163.8, 160.7, 136.5, 128.9, 121.0, 113.6, 65.9, 65.1, 32.1, 29.85, 29.81, 29.73, 29.69, 29.66, 29.5, 29.44, 29.39, 28.8, 26.3, 26.2, 23.4, 22.8, 14.3 ppm. Anal. calcd for $C_{40}H_{71}NO_4Br_2 \times 1.7H_2O$: C, 58.56; H, 9.14; N, 1.71; found: C, 58.25; H, 8.70, N, 1.59.

General procedure for bromine nucleophilic substitution of unsymmetric pyrrole derivatives:

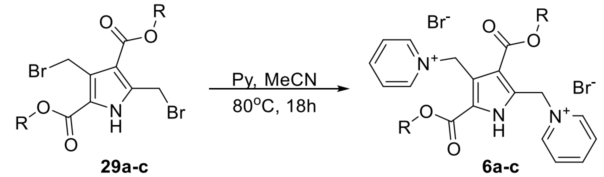

29a-c **6a-c**

Dialkyl 3,5-bis(bromomethyl)-1*H*-pyrrole-2,4-dicarboxylate **29a-c** (1.0 eq) was dissolved in anhydrous acetonitrile and then pyridine (3.0 eq) was added. The reaction mixture was stirred at 80 °C for 18 h. The solvent was evaporated and the crude product was lyophilized.

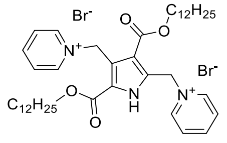

1,1'-((3,5-Bis((dodecyloxy)carbonyl)-1*H*-pyrrole-2,4-diyl)bis(methylene))bis(pyridin-1-ium) dibromide (6a): Light brown solid, yield: 99 %. ^{1}H-NMR (600 MHz, CD_3OD, δ): 9.10 (d, *J*=6.3 Hz, 2H), 9.07 (d, *J*=6.3 Hz, 2H), 8.65 (t, *J*=7.8 Hz, 1H), 8.59 (t, *J*=7.8 Hz, 1H), 8.16 (dd, *J*=7.8, 6.3 Hz, 2H), 8.11 (t, *J*=7.8, 6.3 Hz, 2H), 6.28 (s, 2H), 6.16 (s, 2H), 4.36 (t, *J*=7.1 Hz, 2H), 4.24 (t, *J*=7.1 Hz, 2H), 1.82-1.69 (m, 2H), 1.58-1.47 (m, 2H), 1.43-1.11 (m, 36H), 0.95-0.83 (m, 6H) ppm. ^{13}C-NMR (151 MHz, CD_3OD, δ): 164.5, 161.0, 147.6, 147.0, 146.1, 146.0, 132.3, 129.6, 129.4, 129.2, 126.5, 122.8, 118.2, 67.0,

66.6, 56.6, 55.9, 33.1, 30.75, 30.73, 30.72, 30.68, 30.64, 30.61, 30.5, 30.4, 30.3, 29.8, 29.7, 27.0, 26.9, 23.7, 14.4 ppm. HRMS TOF ES+ of $C_{42}H_{65}N_3O_4$ $[M]^{2+}$ (m/z) 337,7494; calcd:337.7482.

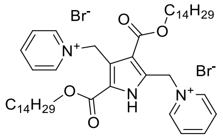

1,1'-((3,5-Bis((tetradecyloxy)carbonyl)-1*H*-pyrrole-2,4-diyl)bis(methylene))bis(pyridin-1-ium) dibromide (6b): Light brown solid, yield: 99 %. ^{1}H-NMR (600 MHz, CD_3OD, δ): 9.09 (d, *J*=6.5 Hz, 2H), 9.06 (d, *J*=6.5 Hz, 2H), 8.65 (t, *J*=7.9 Hz, 1H), 8.59 (t, *J*=7.9 Hz, 1H), 8.16 (dd, *J*=7.8, 6.5 Hz, 2H), 8.10 (t, *J*=7.9, 6.5 Hz, 2H), 6.27 (s, 2H), 6.15 (s, 2H), 4.37 (t, *J*=7.1 Hz, 2H), 4.25 (t, *J*=7.1 Hz, 2H), 1.82-1.72 (m, 2H), 1.57-1.50 (m, 2H), 1.41-1.13 (m, 44H), 0.93-0.87 (m, 6H) ppm. ^{13}C-NMR (151 MHz, CD_3OD, δ): 164.5, 161.0, 147.6, 147.0, 146.1, 146.0, 132.3, 129.6, 129.2, 126.5, 122.8, 118.2, 67.0, 66.6, 56.6, 55.9, 33.1, 30.79, 30.77, 30.75, 30.69, 30.65, 30.62, 30.5, 30.4, 30.3, 26.9, 23.7, 14.4 ppm. HRMS TOF ES+ of $C_{46}H_{73}N_3O_4$ $[M]^{2+}$ (m/z) 365,7798; calcd: 365.7795.

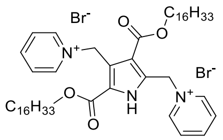

1,1'-((3,5-Bis((hexadecyloxy)carbonyl)-1*H*-pyrrole-2,4-diyl)bis(methylene))bis(pyridin-1-ium) dibromide (6c): Light brown solid, yield: 78 %. ^{1}H-NMR (600 MHz, CD_3OD, δ): 9.07 (d, *J*=6.5 Hz, 2H), 9.02 (d, *J*=6.5 Hz, 2H), 8.63 (t, *J*=7.8 Hz, 1H), 8.57 (t, *J*=7.8 Hz, 1H), 8.13 (dd, *J*=7.8, 6.5 Hz, 2H), 8.08 (t, *J*=7.8, 6.5 Hz, 2H), 6.27 (s, 2H), 6.13 (s, 2H), 4.36 (t, *J*=6.8 Hz, 2H), 4.23 (t, *J*=6.9 Hz, 2H), 1.82 – 1.69 (m, 2H), 1.58-1.48 (m, 2H), 1.45-1.10 (m, 52H), 0.94-0.86 (m, 6H) ppm. ^{13}C-NMR (151 MHz, CD_3OD, δ): 164.5, 161.1, 147.6, 147.0, 146.1, 146.0, 132.5, 129.5, 129.2, 126.6, 122.8, 118.1, 67.0, 66.5, 56.7, 55.9, 30.1, 30.79, 30.76, 30.73, 30.70, 30.65, 30.63, 30.5, 30.4, 30.3, 26.9, 23.7, 14.5 ppm. HRMS TOF ES+ of $C_{50}H_{81}N_3O_4$ $[M]^{2+}$ (m/z) 393,8113; calcd: 393.8108.

S2.5. Unsymmetric furans 7a-c

<u>*General procedure for transesterification of unsymmetric furan derivatives:*</u>

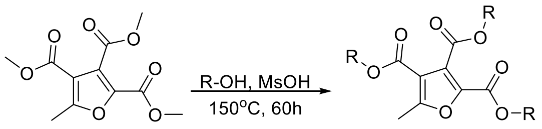

33 34a-c

Trimethyl 5-methylfuran-2,3,4-tricarboxylate **33** (1.0 eq) was placed in round bottom flask, after which an appropriate alcohol (3.0 eq) and methanesulfonic acid (0.1 eq) were added. The reaction mixture was stirred at 150 °C for 60 h (monitored by LC/MS). The crude product was purified with column chromatography (gradient: 0 to 20 % EtOAc in PE).

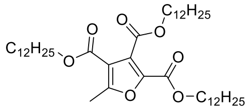

Tridodecyl 5-methylfuran-2,3,4-tricarboxylate (34a): Yellow oil, yield: 24 %. ^{1}H-NMR (400 MHz, $CDCl_3$, δ): 4.29 (t, *J*=6.8 Hz, 2H), 4.27 (t, *J*=6.7 Hz, 2H) 4.22 (t, *J*=6.7 Hz, 2H), 2.65 (s, 3H), 1.78-1.61 (m, 6H), 1.44-1.18 (m, 54H), 0.89 (t, *J*=6.9 Hz, 9H) ppm. ^{13}C-NMR (101 MHz, $CDCl_3$, δ): 163.5, 162.4, 162.0, 157.6, 139.2, 126.2, 114.3, 66.5, 65.9, 65.4, 32.1, 29.83, 29.80, 29.78, 29.75, 29.67, 29.5, 29.4, 28.7, 28.6, 26.1, 26.0, 25.9, 22.8, 14.33, 14.26 ppm.

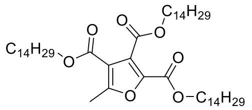

Tritetradecyl 5-methylfuran-2,3,4-tricarboxylate (34b): Light yellow oil, yield: 21 %. ^{1}H-NMR (400 MHz, $CDCl_3$, δ): 4.29 (t, *J*=6.8 Hz, 2H), 4.27 (t, *J*=6.7 Hz, 2H), 4.22 (t, *J*=6.8 Hz, 2H), 2.65 (s, 3H), 1.78-1.61 (m, 6H), 1.43-1.17 (m, 66H), 0.88 (t, *J*=7.0 Hz, 9H) ppm. ^{13}C-NMR (101 MHz, $CDCl_3$, δ): 163.5, 162.4, 162.0, 157.6, 139.2, 126.2, 114.3, 66.6, 65.9, 65.4, 32.1, 29.85, 29.84, 29.81, 29.79, 29.76, 29.68, 29.52, 29.49, 29.43, 28.7, 28.6, 26.1, 26.0, 25.9, 22.8, 14.33, 14.27 ppm.

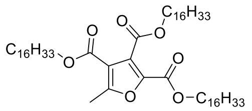

Trihexadecyl 5-methylfuran-2,3,4-tricarboxylate (34c): Light yellow oil, yield: 13 %. ^{1}H-NMR (400 MHz, $CDCl_3$, δ): 4.29 (t, *J*=6.8 Hz, 2H), 4.27 (t, *J*=6.7 Hz, 2H), 4.22 (t, *J*=6.7 Hz, 2H), 2.65 (s, 3H), 1.77-1.61 (m, 6H), 1.45-1.19 (m, 78H), 0.87 (t, *J*=6.9 Hz, 9H) ppm. ^{13}C-NMR (101 MHz, $CDCl_3$, δ): 163.5, 162.4, 162.0, 157.6, 139.2, 126.2, 114.3, 66.6, 65.9, 65.4, 32.1, 29.86, 29.82, 29.7, 29.75, 29.69, 29.52, 29.50, 29.4, 28.7, 28.6, 26.1, 26.0, 25.9, 22.9, 14.33, 14.27 ppm.

General procedure for the bromination of methyl groups and bromine nucleophilic substitution in unsymmetric furan derivatives:

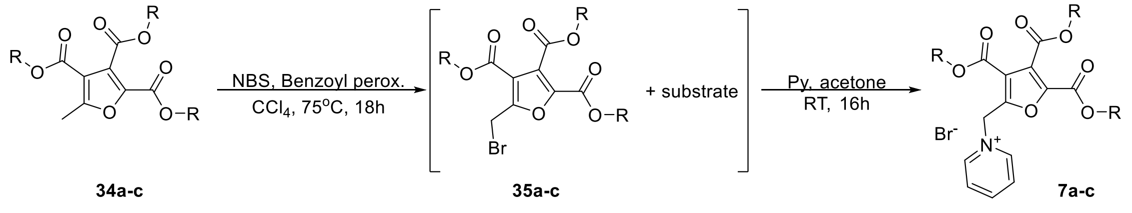

Trialkyl 5-methylfuran-2,3,4-tricarboxylate **34a-c** (1.0 eq), NBS (1.2 eq) and benzoyl peroxide (0.2 eq) were suspended in CCl_4 and stirred at 75 °C for 18 h. The reaction mixture was evaporated and the crude product was purified with column chromatography (gradient: 0 to 15 % EtOAc in PE). The product and residual starting material could not be fully separated; therefore the mixture was used in next steps without complete purification. The formation of compounds **35a-c** was confirmed by ^{1}H-NMR through the observation of the main peaks. Mixture containing brominated compound **35a-c** (1.2 eq) was dissolved in dry acetone and then pyridine (2.5 eq) was

added. Reaction mixture was stirred at room temperature for 18 h. The resulting mixture was evaporated and purified with column chromatography (gradient: 0 to 20 % MeOH in DCM), then lyophilized giving compound **7a-c**.

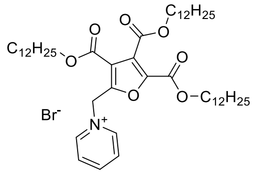

1-((3,4,5-Tris((dodecyloxy)carbonyl)furan-2-yl)methyl)pyridin-1-ium bromide (7a): Yellow solid, hygroscopic, yield: 50 %. ^{1}H-NMR (600 MHz, CD_3OD, δ): 9.14 (d, *J*=5.9 Hz, 2H), 8.68 (t, *J*=7.8 Hz, 1H), 8.18 (dd, *J*=7.8, 5.9 Hz, 2H), 6.28 (s, 2H), 4.37-4.25 (m, 6H), 1.83-1.64 (m, 6H), 1.48-1.22 (m, 54H), 0.90 (t, *J*=6.9 Hz, 9H) ppm. ^{13}C-NMR (151 MHz, CD_3OD, δ): 163.5, 162.3, 157.9, 154.7, 148.2, 146.8, 143.4, 129.8, 126.3, 119.4, 67.8, 67.4, 56.7, 33.1, 30.84, 30.80, 30.75, 30.70, 30.52, 30.46, 30.39, 29.73, 29.71, 29.6, 27.11, 27.03, 26.96, 23.8, 14.5 ppm. HRMS TOF ES+ of $C_{49}H_{82}NO_7$ $[M]^+$ (m/z) 796,6106; calcd: 796,6091.

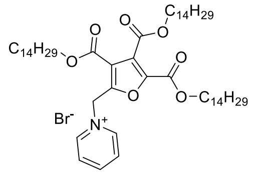

1-((3,4,5-Tris((tetradecyloxy)carbonyl)furan-2-yl)methyl)pyridin-1-ium bromide (7b): White solid, hygroscopic, yield: 72 %. ^{1}H-NMR (600 MHz, CD_3OD, δ): 9.14 (d, *J*=6.5 Hz, 2H), 8.68 (t, *J*=7.8 Hz, 1H), 8.18 (dd, *J*=7.8, 6.5 Hz, 2H), 6.27 (s, 2H), 4.39-4.24 (m, 6H), 1.87-1.64 (m, 6H), 1.49-1.21 (m, 66H), 0.90 (t, *J*=6.9 Hz, 9H) ppm. ^{13}C-NMR (151 MHz, CD_3OD, δ): 163.5, 162.3, 157.9, 154.8, 148.2, 146.8, 143.4, 129.8, 126.3, 119.4, 67.8, 67.4, 56.7, 33.1, 30.86, 30.84, 30.83, 30.82, 30.79, 30.77, 30.74, 30.72, 30.71, 30.53, 30.47, 30.4, 29.8, 29.7, 29.6, 27.12, 27.05, 27.0, 23.8, 14.5 ppm. HRMS TOF ES+ of $C_{55}H_{94}NO_7$ $[M]^+$ (m/z) 880,7002; calcd: 880,7030.

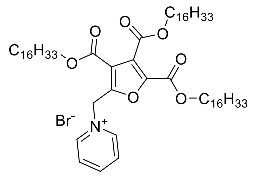

1-((3,4,5-Tris((hexadecyloxy)carbonyl)furan-2-yl)methyl)pyridin-1-ium bromide (7c): White solid, hygroscopic, yield: 35 %. ^{1}H-NMR (600 MHz, CD_3OD, δ): 9.14 (d, *J*=6.4 Hz, 2H), 8.68 (t, *J*=7.9 Hz, 1H), 8.18 (dd, *J*=7.9, 6.5 Hz, 2H), 6.28 (s, 2H), 4.39-4.27 (m, 6H), 1.91-1.63 (m, 10H), 1.30 (d, *J*=11.7 Hz, 74H), 0.97-0.84 (m, 9H) ppm. ^{13}C-NMR (151 MHz, CD_3OD, δ): 163.5, 162.3, 157.9 ,154.8, 148.2, 146.8, 143.4, 129.9, 126.3, 119.4, 67.8, 67.4, 33.1, 30.87, 30.83, 30.79, 30.76, 30.72, 30.53, 30.50, 30.49, 30.43, 29.8, 29.7, 29.6, 27.1, 23.8, 14.5 ppm. HRMS TOF ES+ of $C_{61}H_{106}NO_7$ $[M]^+$ (m/z) 964,7931; calcd: 964.7969.

S2.6. Amphiphiles with aliphatic linker (half-products) 8a-c, 9a-c, and 10a-c

Bromination of 4-carbon in acetoacetate derivatives

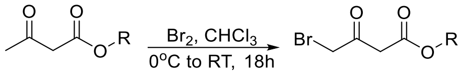

36a-c **38a-c**

Alkyl 3-oxobutanoate **36a-c** (1.0 eq) was dissolved in $CHCl_3$ and cooled to -5 °C in ice-salt bath. Then a solution of bromine (1.0 eq) in $CHCl_3$ was added dropwise under argon atmosphere. The reaction mixture was stirred for 16 h at room temperature and then air was blown in via bubbler for 2 h. The reaction mixture was washed with 10 mL cold water (~5 °C), and then extracted with DCM (3 × 5 mL). The organic layers were combined, dried over anhydrous Na_2SO_4, filtered and evaporated. The crude product was checked with ^{1}H-NMR to approve bromination at 4-carbon and used in next step without additional purification. The formation of compounds **38a-c** was confirmed by ^{1}H-NMR through the observation of the main peaks, as depicted in Figure S1.

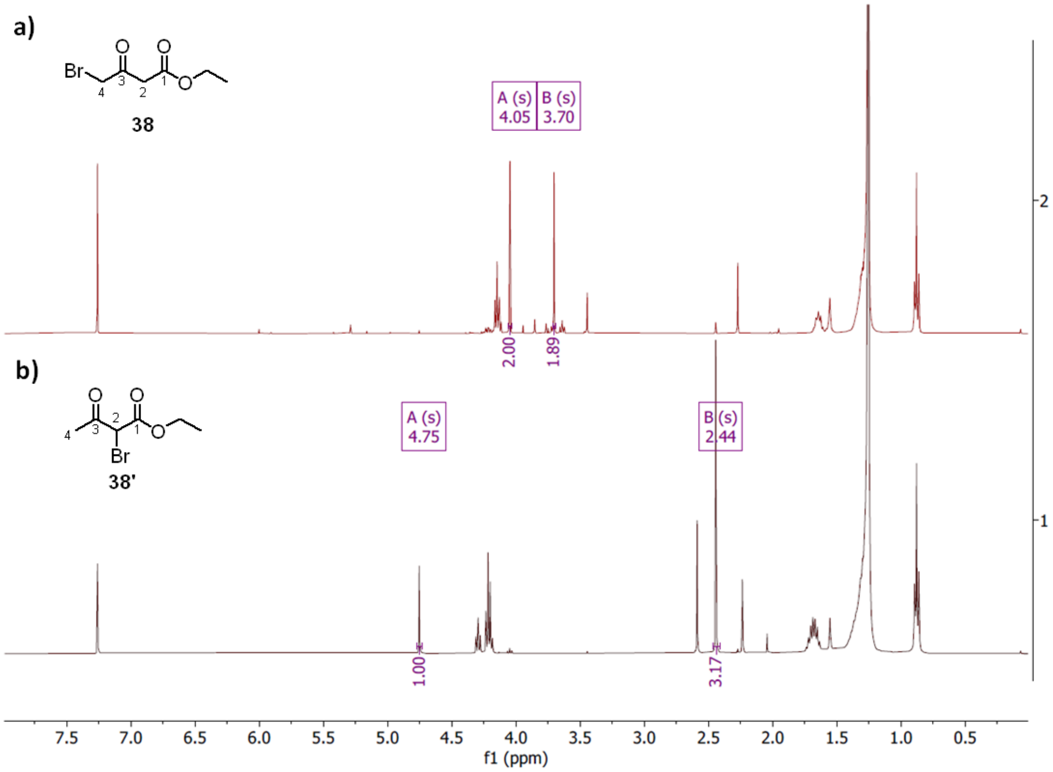

Figure S1: ^{1}H-NMR (400 MHz, $CDCl_3$) spectral data comparison: **a**) bromination at 4-carbon yielding compound **38** and **b**) bromination at 2-carbon yielding compound **38'**.

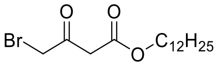

Crude intermediate of dodecyl 4-bromo-3-oxobutanoate (38a): ^{1}H-NMR (400 MHz, $CDCl_3$, δ): 4.12 (t, *J*=6.8 Hz, 2H), 4.03 (s, 2H), 3.68 (s, 2H), 1.65-1.58 (m, 2H), 1.33-1.20 (m, 18H), 0.85 (t, *J*=7.0 Hz, 3H) ppm.

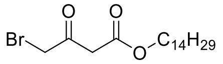

Crude intermediate of tetradecyl 4-bromo-3-oxobutanoate (38b): ^{1}H-NMR (400 MHz, $CDCl_3$, δ): 4.13 (t, *J*=6.8 Hz, 2H), 4.04 (s, 2H), 3.68 (s, 2H), 1.65-1.57 (m, 2H), 1.35-1.18 (m, 22H), 0.86 (t, *J*=7.0 Hz, 3H) ppm.

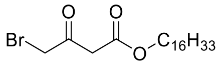

Crude intermediate of hexadecyl 4-bromo-3-oxobutanoate (38c): ^{1}H-NMR (300 MHz, $CDCl_3$, δ): 4.15 (t, *J*=6.8 Hz, 2H), 4.05 (s, 2H), 3.70 (s, 2H), 1.69-1.58 (m, 2H), 1.38-1.19 (m, 26H), 0.88 (t, *J*=7.0 Hz, 3H) ppm.

General procedure for synthesis of enaminoesters:

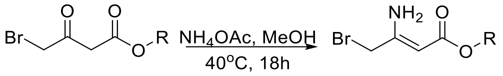

Alkyl 4-bromo-3-oxobutanoate **38a-c** (1.0 eq) was dissolved in MeOH, after which NH_4OAc (1.1 eq) was added and reaction mixture was stirred at 40 °C for 18 h. The resulting orange solution was cooled in freezer to -18 °C and filtered. The precipitate was used in the next step without additional purification. The formation of compounds **40a-c** was confirmed by ^{1}H-NMR through the observation of the main peaks.

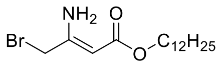

Crude intermediate of dodecyl (*Z*)-3-amino-4-bromobut-2-enoate (40a): ^{1}H-NMR (400 MHz, $CDCl_3$, δ): 4.78 (s, 1H), 4.07 (t, *J*=6.8 Hz, 2H), 3.88 (s, 2H), 1.66-1.56 (m, 2H), 1.37-1.21 (m, 18H), 0.87 (t, *J*=7.0 Hz, 3H) ppm.

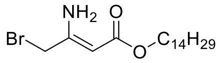

Crude intermediate of tetradecyl (*Z*)-3-amino-4-bromobut-2-enoate (40b): ^{1}H-NMR (400 MHz, $CDCl_3$, δ): 4.78 (s, 1H), 4.06 (t, *J*=6.7 Hz, 2H), 3.89 (s, 2H), 1.66-1.57 (m, 2H), 1.39-1.22 (m, 22H), 0.88 (t, *J*=7.0 Hz, 3H) ppm.

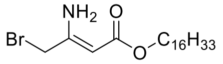

Crude intermediate of hexadecyl (*Z*)-3-amino-4-bromobut-2-enoate (40c): ^{1}H-NMR (400 MHz, $CDCl_3$, δ): 4.78 (s, 1H), 4.05 (t, *J*=6.8 Hz, 2H), 3.88 (s, 2H), 1.71-1.51 (m, 2H), 1.39-1.19 (m, 26H), 0.87 (t, *J*=7.0 Hz, 3H) ppm.

General procedure for nucleophilic substitution of bromide:

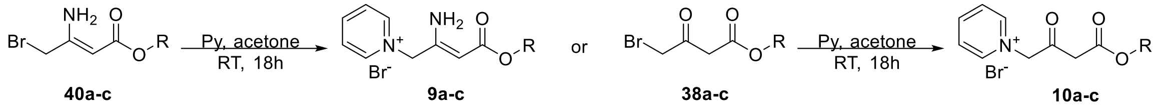

Alkyl (*Z*)-3-amino-4-bromobut-2-enoate **40a-c** or alkyl 4-bromo-3-oxobutanoate **38a-c** (1.0 eq) was dissolved in distilled acetone and pyridine (1.1 eq) was added. The reaction mixture was stirred at room temperature for 18 h. The resulting suspension was filtered and the precipitate was crystallized from acetone to yield pure product **9a-c** or **10a-c**.

a)

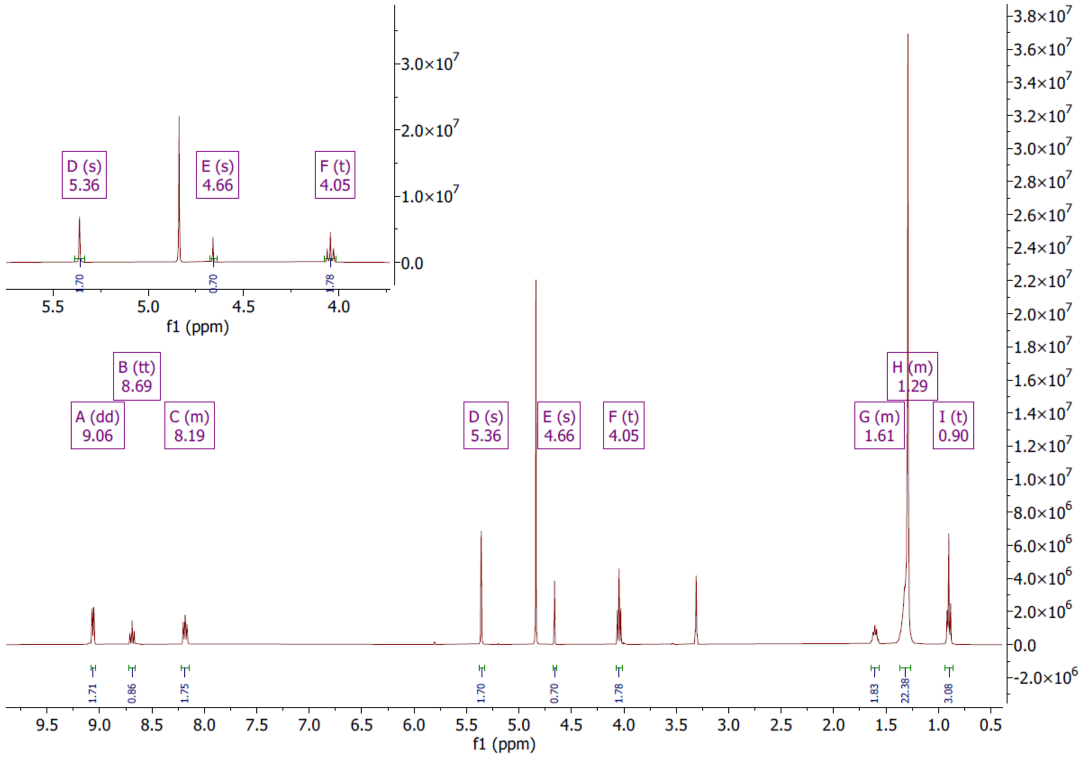

b)

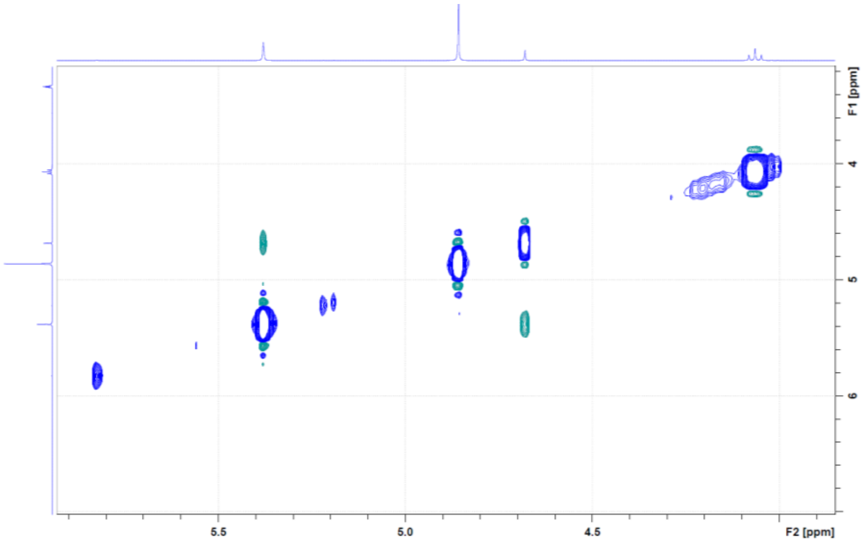

Figure S2: Determination of *E*/*Z* isomer for compound **9b**; ^{1}H-NMR (400 MHz, CD_3OD) spectra (**a**) and NOESY spectra to prove *Z*-isomer formation (**b**).

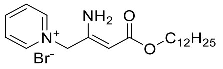

(*Z*)-1-(2-Amino-4-(dodecyloxy)-4-oxobut-2-en-1-yl)pyridin-1-ium bromide (9a): Brown solid, mp. 110.8-111.4 °C (acetone), yield: 27 %. ^{1}H-NMR (400 MHz, CD_3OD, δ): 9.04-9.01 (m, 2H), 8.69 (tt, *J*=7.8, 1.4 Hz, 1H), 8.22-8.14 (m, 2H), 5.33 (s, 2H), 4.64 (s, 1H), 4.05 (t, *J*=6.6 Hz, 2H), 1.64-1.55 (m, 2H), 1.37-1.25 (m, 18H), 0.90 (t, *J*=7.0 Hz, 3H) ppm. ^{13}C-NMR (101 MHz, CD_3OD, δ): 170.9, 154.9, 148.1, 146.4, 129.6, 87.4, 64.4, 33.1, 30.77, 30.74, 30.70, 30.68, 30.47, 30.41, 30.0, 27.1, 23.7, 14.4 ppm. Anal. calcd for $C_{21}H_{35}BrN_2O_2$: C, 59.01; H, 8.25; N, 6.55; found: C, 58.88; H, 8.22; N, 6.43.

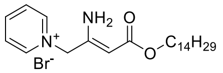

(*Z*)-1-(2-Amino-4-oxo-4-(tetradecyloxy)but-2-en-1-yl)pyridin-1-ium bromide (9b): Light-brown solid, mp. 112.9-113.6 °C (acetone), yield: 30 %. ^{1}H-NMR (400 MHz, CD_3OD, δ): 9.06-9.03 (m, 2H), 8.69 (tt, *J*=7.8, 1.4 Hz, 1H) 8.21-8.14 (m, 2H), 5.34 (s, 2H), 4.65 (s, 1H), 4.05 (t, *J*=6.6 Hz, 2H), 1.64-1.56 (m, 2H), 1.38-1.23 (m, 22H), 0.90 (t, *J*=7.0 Hz, 3H) ppm. ^{13}C-NMR (101 MHz, CD_3OD, δ): 170.9, 155.1, 148.0, 146.4, 129.6, 87.40, 87.38, 64.4, 33.1, 30.79, 30.75, 30.71, 30.68, 30.47, 30.42, 30.0, 27.1, 14.4 ppm. Anal. calcd for $C_{23}H_{39}BrN_2O_2$: C, 60.65; H, 8.63; N, 6.15; found: C, 60.83; H, 8.70; N, 6.00.

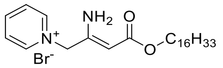

(*Z*)-1-(2-Amino-4-(hexadecyloxy)-4-oxobut-2-en-1-yl)pyridin-1-ium bromide (9c): Light-brown solid, mp. 114.4-115.1 °C (acetone), yield: 33 %. ^{1}H-NMR (400 MHz, CD_3OD, δ): 9.06-9.03 (m, 2H), 8.69 (tt, *J*=7.8, 1.3 Hz, 1H), 8.22-8.13 (m, 2H), 5.34 (s, 2H), 4.65 (s, 1H), 4.05 (t, *J*=6.6 Hz, 2H), 1.64-1.55 (m, 2H), 1.40-1.23 (m, 26H), 0.90 (t, *J*=7.0 Hz, 3H) ppm. ^{13}C-NMR (101 MHz, CD_3OD, δ): 170.9, 154.9, 148.0, 146.4, 129.6, 87.41, 87.39, 64.4, 33.1, 30.78, 30.75, 30.70, 30.68, 30.47, 30.42, 30.0, 27.1, 23.7, 14.4 ppm. Anal. calcd for $C_{25}H_{43}BrN_2O_2$: C, 62.10; H, 8.96; N, 5.79; found: C, 61.90; H, 8.89; N, 5.52.

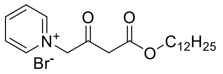

1-(4-(Dodecyloxy)-2,4-dioxobutyl)pyridin-1-ium bromide (10a): White solid, mp. 119.5-120.1 °C (acetone), yield: 54 %. ^{1}H-NMR (400 MHz, $CDCl_3$, δ): 9.29 (d, *J*=6.4 Hz, 2H), 8.51 (t, *J*=7.8 Hz, 1H), 8.08 (dd, *J*=7.6, *J*=6.4 Hz, 2H), 6.72 (s, 2H), 4.12 (t, *J*=6.8 Hz, 2H), 4.01 (s, 2H), 1.69-1.58 (m, 2H), 1.37-1.19 (s, 18H), 0.86 (t, *J*=7.0 Hz, 3H) ppm. ^{13}C-NMR (101 MHz, $CDCl_3$, δ): 194.4, 167.1, 146.5, 145.8, 127.9, 68.8, 66.4, 47.1, 32.0, 29.78, 29.76, 29.72, 29.64, 29.47, 29.37, 28.6, 25.9, 22.8, 14.3 ppm. Anal. calcd for $C_{21}H_{34}BrNO_3$: C, 58.88; H, 8.00; N, 3.27; found: C, 58.75; H, 7.99; N, 3.27.

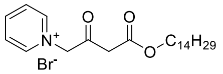

1-(2,4-Dioxo-4-(tetradecyloxy)butyl)pyridin-1-ium bromide (10b): White solid, mp. 119.7-120.5 ^{o}C (acetone), yield: 66 %. ^{1}H-NMR (400 MHz, CDCl$_3$, δ): 9.29 (d, *J*=6.5 Hz, 2H), 8.50 (t, *J*=8.0 Hz, 1H), 8.08 (dd, *J*=8.0, *J*=6.5 Hz, 2H), 4.13 (t, *J*=6.8 Hz, 2H), 4.02 (s, 2H), 1.68-1.57 (m, 2H), 1.36-1.19 (m, 22H), 0.87 (t, *J*=7.0 Hz, 3H) ppm. ^{13}C-NMR (101 MHz, CDCl$_3$, δ): 194.4, 167.1, 146.5, 145.8, 127.9, 68.8, 66.4, 47.1, 32.1, 29.83, 29.81, 29.79, 29.73, 29.65, 29.49, 29.38, 28.6, 25.9, 22.8, 14.3 ppm. Anal. calcd for $C_{23}H_{38}BrNO_3$: C, 60.52; H, 8.39; N, 3.07; found: C,60.62; H, 8.45; N, 2.99.

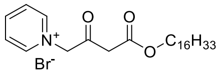

1-(4-(Hexadecyloxy)-2,4-dioxobutyl)pyridin-1-ium bromide (10c): White solid, mp. 120.5-121.2 ^{o}C (acetone), yield: 72 %. ^{1}H-NMR (400 MHz, CDCl$_3$, δ): 9.29 (d, *J*=6.3 Hz, 2H), 8.51 (t, *J*=7.8 Hz, 1H), 8.08 (t, , *J*=7.8, *J*=6.3 Hz, 2H), 6.72 (s, 2H), 4.12 (t, *J*=6.8 Hz, 2H), 4.01 (s, 2H), 1.68-1.58 (m, 2H), 1.36-1.19 (m, 26H), 0.87 (t, *J*=7.0 Hz, 3H) ppm. ^{13}C-NMR (101 MHz, CDCl$_3$, δ): 194.4, 167.0, 146.5, 145.8, 127.9, 68.8, 66.4, 47.1, 32.0, 29.83, 29.78, 29.74, 29.65, 29.48, 29.38, 28.6, 25.9, 22.8, 14.3 ppm. Anal. calcd for $C_{25}H_{42}BrNO_3$: C, 61.97; H, 8.74; N, 2.89; found: C, 61.93; H, 8.79; N, 2.79.

General procedure for nucleophilic substitution of chloride:

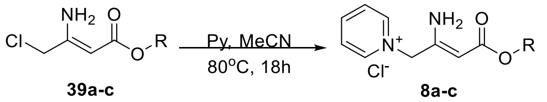

Alkyl (*Z*)-3-amino-4-chlorobut-2-enoate **39a-c** (1.0 eq) was dissolved in anhydrous MeCN after which pyridine (3.0 eq) was added. The reaction mixture was stirred at 80 ^{o}C for 18 h. The resulting mixture was cooled in freezer to -18 ^{o}C and filtered. The precipitate was crystallized from MeCN to yield pure product **8a-c**.

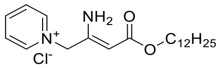

(*Z*)-1-(2-Amino-4-(dodecyloxy)-4-oxobut-2-en-1-yl)pyridin-1-ium chloride (8a): Light brown solid, mp. 122.8-123.5 ^{o}C (MeCN), yield: 25 %. ^{1}H-NMR (400 MHz, CD$_3$OD, δ): 9.06 (dd, *J*=6.8, 1.3 Hz, 2H), 8.69 (tt, *J*=7.8, 1.3 Hz, 1H), 8.21-8.13 (m, 2H), 5.34 (s, 2H), 4.65 (s, 1H), 4.05 (t, *J*=6.6 Hz, 2H), 1.64-1.56 (m, 2H), 1.41-1.25 (m, 18H), 0.90 (t, *J*=6.7 Hz, 3H) ppm. ^{13}C-NMR (101 MHz, CD$_3$OD, δ): 170.9, 155.1, 148.0, 146.4, 129.6, 87.3, 64.4, 33.1, 30.77, 30.74, 30.70, 30.68, 30.47, 30.41, 30.0, 27.1, 23.7, 14.5 ppm. Anal. calcd for $C_{21}H_{35}ClN_2O_2$: C, 65.86; H, 9.21; N, 7.31; found: C, 65.75; H, 9.30; N, 7.14.

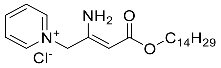

(*Z*)-1-(2-Amino-4-oxo-4-(tetradecyloxy)but-2-en-1-yl)pyridin-1-ium chloride (8b): Light brown solid, mp. 126.4-127.0 °C (MeCN), yield: 29 %. ^{1}H-NMR (400 MHz, CD_3OD, δ): 9.04 (dd, *J*=6.6, 1.4 Hz, 2H), 8.69 (tt, *J*=7.8, 1.4 Hz, 1H), 8.21-8.14 (m, 2H), 5.343 (s, 2H), 4.64 (s, 1H), 4.05 (t, *J*=6.6 Hz, 2H), 1.64-1.56 (m, 2H), 1.37-1.24 (m, 22H), 0.91 (t, *J*=6.7 Hz, 3H) ppm. ^{13}C-NMR (101 MHz, CD_3OD, δ): 170.9, 155.1, 148.0, 146.4, 129.6, 87.3, 64.4, 33.1, 30.79, 30.76, 30.70, 30.68, 30.47, 30.42, 30.0, 27.1, 23.7, 14.4 ppm. Anal. calcd for $C_{23}H_{39}ClN_2O_2 \times H_2O$: C, 64.39; H, 9.63; N, 6.53; found: C, 64.52; H, 9.32; N, 6.59.

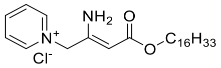

(*Z*)-1-(2-Amino-4-(hexadecyloxy)-4-oxobut-2-en-1-yl)pyridin-1-ium chloride (8c): Light brown solid, mp. 129.7-130.6 °C (MeCN), yield: 31 %. ^{1}H-NMR (400 MHz, CD_3OD, δ): 9.04 (dd, *J*=6.6, 1.4 Hz, 2H), 8.69 (tt, *J*=7.8, 1.4 Hz, 1H), 8.22-8.12 (m, 2H), 5.34 (s, 2H), 4.65 (s, 1H), 4.05 (t, *J*=6.6 Hz, 2H), 1.67-1.54 (m, 2H), 1.40-1.23 (m, 26H), 0.90 (t, *J*=6.8 Hz, 3H) ppm. ^{13}C-NMR (101 MHz, CD_3OD, δ): 170.9, 155.1, 148.0, 146.4, 129.6, 87.3, 64.4, 33.1, 30.77, 30.75, 30.70, 30.68, 30.47, 30.42, 30.0, 27.1, 23.7, 14.4 ppm. Anal. calcd for $C_{25}H_{43}ClN_2O_2 \times 0.5H_2O$: C, 67.01; H, 9.90; N, 6.25; found: C, 67.01; H, 9.90; N, 6.04.

S3. Self-assembling

Table S1. Average hydrodynamic diameters (Z_{av}) and polydispersity indexes (PdI) of the nanoparticles formed from heterocyclic and aliphatic amphiphiles.

Entry		Symmetric compounds									
		1a	3a	3b	3c	4a	4b	4c	5a	5b	5c
1	Z_{av}, nm	298 ± 19	650 ± 29	1317 ± 132	470 ± 21	299 ± 29	230 ± 18	330 ± 21	208 ± 4	164 ± 4	183 ± 7
2	PdI	0.405 ± 0.056	0.662 ± 0.057	0.833 ± 0.085	0.500 ± 0.024	0.425 ± 0.045	0.347 ± 0.009	0.518 ± 0.001	0.320 ± 0.034	0.225 ± 0.034	0.258 ± 0.063
		Unsymmetric compounds									
		6a	6b	6c	7a	7b	7c				
3	Z_{av}, nm	92 ± 6	433 ± 28	173 ± 2	112 ± 4	104 ± 2	109 ± 3				
4	PdI	0.344 ± 0.020	0.458 ± 0.032	0.115 ± 0.025	0.139 ± 0.030	0.203 ± 0.016	0.165 ± 0.016				
		Aliphatic compounds ("half products")									
		8a	8b	8c	9a	9b	9c	10a	10b	10c	
5	Z_{av}, nm	100 ± 10	154 ± 20	293 ± 20	283 ± 37	155 ± 14	389 ± 7	214 ± 16	656 ± 31	196 ± 4	
6	PdI	0.154 ± 0.058	0.305 ± 0.059	0.339 ± 0.020	0.367 ± 0.092	0.426 ± 0.081	0.265 ± 0.013	0.285 ± 0.035	0.677 ± 0.037	0.131 ± 0.036	

Table S2. Results of Dunnett's T3 multiple comparison test for Z_{av} values of nanoparticles formed from heterocyclic amphiphiles.

Entry	1	2	3	4	5	6	7	8	9	10	11	12
Cmpd.	3a vs. 4a	3b vs. 4b	3c vs. 4c	4a vs. 5a	4b vs. 5b	4c vs. 5c	5a vs. 6a	5b vs. 6b	5c vs. 6c	5a vs. 7a	5b vs. 7b	5c vs. 7c
Summary	*	ns	ns	ns	ns	ns	****	ns	ns	****	ns	**
Adjusted P Value	0.028	0.135	0.0863	0.3501	0.3062	0.1585	<0,0001	0.1168	0.4877	<0,0001	0.0694	0.0028

S4. Encapsulation

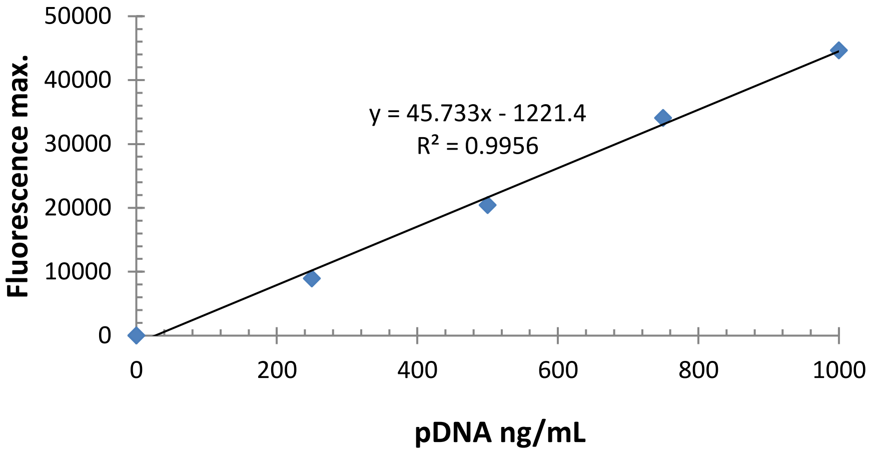

Figure S3. Calibration curve for encapsulated pDNA concentration calculations.

Table S3. Encapsulation of DNA plasmid in liposomes formed by symmetric (**1a**, **3a-c**, **4a-c**, **5a-c**), unsymmetric (**6a-c**, **7a-c**), and aliphatic (**8a-c**, **9a-c**, **10a-c**) amphiphiles at four different N/P ratios.

Encapsulation, %

Entry		Symmetric compounds									
		1a	3a	3b	3c	4a	4b	4c	5a	5b	5c
1	N/P=1	–	24 ± 4.6	49 ± 5.4	23 ± 0.6	–	34 ± 5.1	39 ± 4.4	–	30 ± 7.7	30 ± 2.0
2	N/P=2	37 ± 1.4	47 ± 4.3	75 ± 0.8	70 ± 2.9	31 ± 1.1	69 ± 6.3	78 ± 1.0	–	75 ± 2.3	80 ± 1.7
3	N/P=4	92 ± 3.3	80 ± 6.1	84 ± 8.1	78 ± 0.8	69 ± 0.4	83 ± 11.4	88 ± 1.7	21 ± 0.8	88 ± 2.8	93 ± 0.6
4	N/P=6	96 ± 3.6	96 ± 0.2	94 ± 0.3	73 ± 6.0	88 ± 0.2	95 ± 0.5	91 ± 0.1	77 ± 2.9	93 ± 0.8	93 ± 0.1
		Unsymmetric compounds									
		6a	6b	6c	7a	7b	7c				
5	N/P=1	–	32 ± 3.4	–	6 ± 8.2	31 ± 6.0	21 ± 4.1				
6	N/P=2	–	75 ± 6.9	21 ± 0.8	26 ± 1.1	75 ± 1.2	75 ± 0.5				
7	N/P=4	52 ± 3.4	91 ± 2.3	81 ± 2.8	64 ± 1.8	93 ± 0.2	91 ± 0.4				
8	N/P=6	71 ± 5.7	90 ± 0.5	84 ± 3.5	85 ± 0.8	97 ± 0.1	95 ± 0.3				
		Aliphatic compounds ("half products")									
		8a	8b	8c	9a	9b	9c	10a	10b	10c	
9	N/P=1	–	–	–	–	–	–	–	–	–	
10	N/P=2	–	–	59 ± 0.7	–	–	41 ± 2.4	–	–	–	
11	N/P=4	35 ± 4.3	5 ± 2.1	89 ± 0.3	7 ± 1.8	46 ± 0.9	79 ± 1.3	–	–	–	
12	N/P=6	67 ± 0.9	29 ± 2.0	90 ± 0.2	16 ± 0.4	74 ± 0.7	91 ± 0.6	–	–	8 ± 0.4	

"—" no encapsulation

Table S4. Average hydrodynamic diameters (Z_{av}) and polydispersity indexes (PdI) of the lipoplexes formed from symmetric (**1a**, **3a-c**, **4a-c**, **5a-c**), unsymmetric (**6a-c**, **7a-c**), and aliphatic (**8c**, **9c**) amphiphiles with encapsulated plasmid DNA pBR322 at N/P=6 ratio.

Entry		Symmetric compounds									
		1a + pDNA	3a + pDNA	3b + pDNA	3c + pDNA	4a + pDNA	4b + pDNA	4c + pDNA	5a + pDNA	5b + pDNA	5c + pDNA
1	Z_{av}, nm	824 ± 56	762 ± 82	826 ± 85	1037 ± 203	707 ± 93	872 ± 68	1159 ± 128	470 ± 32	879 ± 124	1121 ± 220
2	PdI	0.257 ± 0.022	0.340 ± 0.159	0.288 ± 0.266	0.285 ± 0.217	0.350 ± 0.161	0.287 ± 0.138	0.173 ± 0.053	0.136 ± 0.102	0.336 ± 0.170	0.450 ± 0.306
		Unsymmetric compounds									
		6a + pDNA	6b + pDNA	6c + pDNA	7a + pDNA	7b + pDNA	7c + pDNA				
3	Z_{av}, nm	720 ± 91	660 ± 59	972 ± 41	302 ± 20	890 ± 50	677 ± 69				
4	PdI	0.842 ± 0.274	0.327 ± 0.258	0.099 ± 0.048	0.122 ± 0.009	0.353 ± 0.230	0.229 ± 0.190				
		Aliphatic compounds									
		8c + pDNA	9c + pDNA								
5	Z_{av}, nm	768 ± 40	657 ± 31								
6	PdI	0.232 ± 0.103	0.278 ± 0.079								

Table S5. Results of Dunnett's T3 multiple comparison test for Z_{av} values of lipoplexes formed from heterocyclic amphiphiles with encapsulated pDNA pBR322 at N/P=6 ratio..

Entry	1	2	3	4	5	6	7	8	9
Cmpd.	3a vs. 3b	3b vs. 3c	3a vs. 3c	4a vs. 4b	4b vs. 4c	4a vs. 4c	5a vs. 5b	5b vs. 5c	5a vs. 5c
Summary	ns	ns	ns	ns	ns	*	ns	ns	ns
Adjusted P Value	0.9495	0.6543	0.4501	0.3153	0.182	0.0434	0.3119	0.6915	0.1289

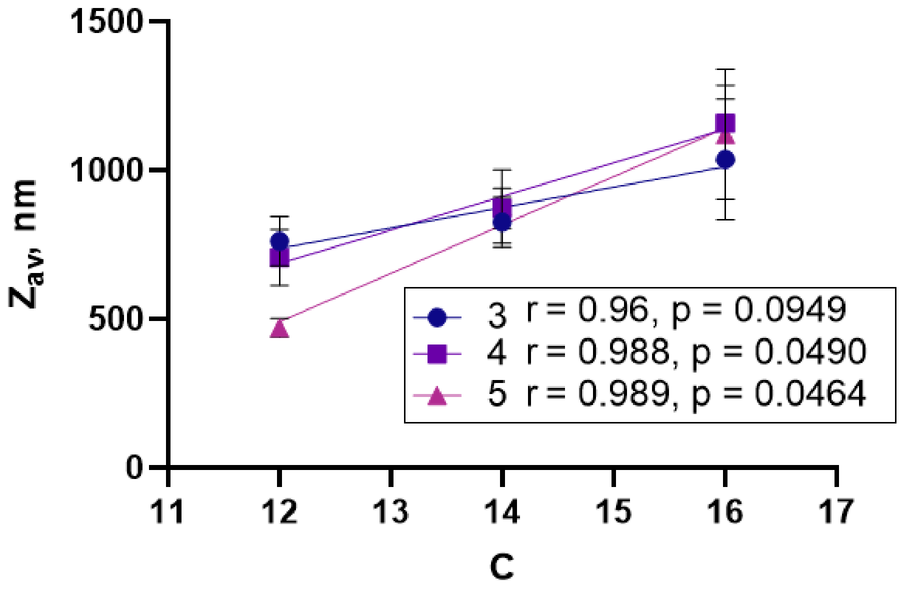

Figure S4: Correlation between hydrodynamic diameter (Z_{av}) and side-chain length of symmetric amphiphiles.

S5. ¹H-NMR spectra and ¹³C-NMR spectra

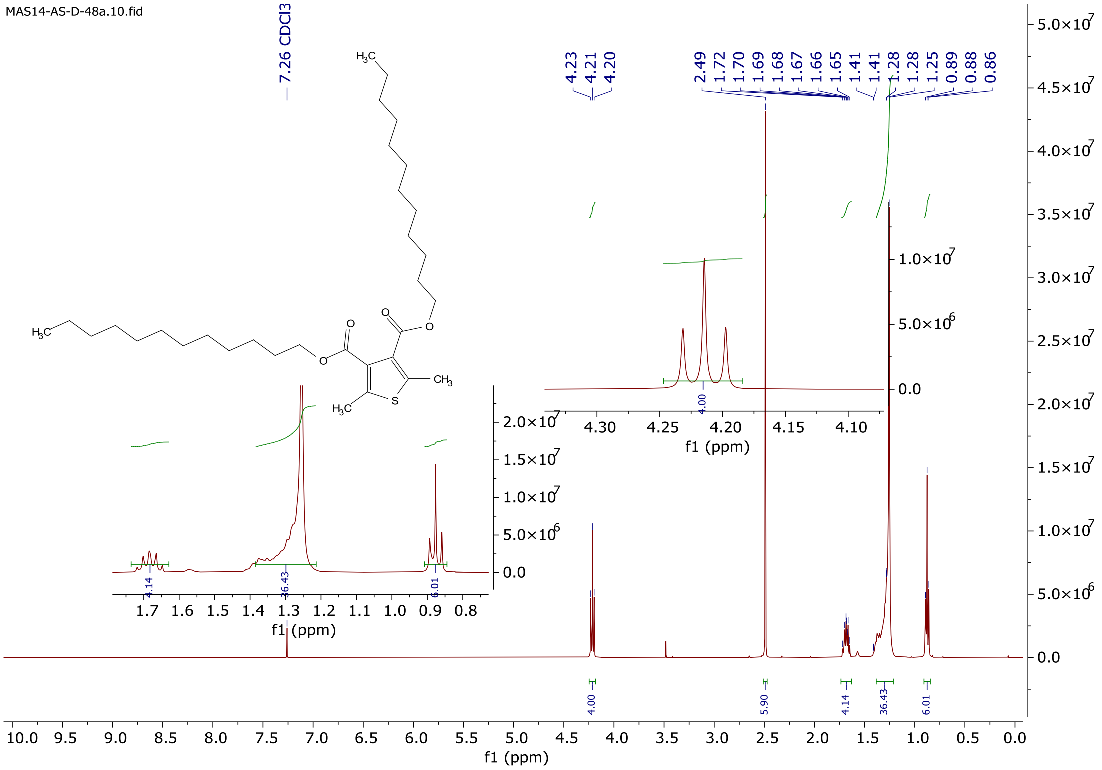

Figure S5: ¹H-NMR spectrum of didodecyl 2,5-dimethylthiophene-3,4-dicarboxylate (**18a**)

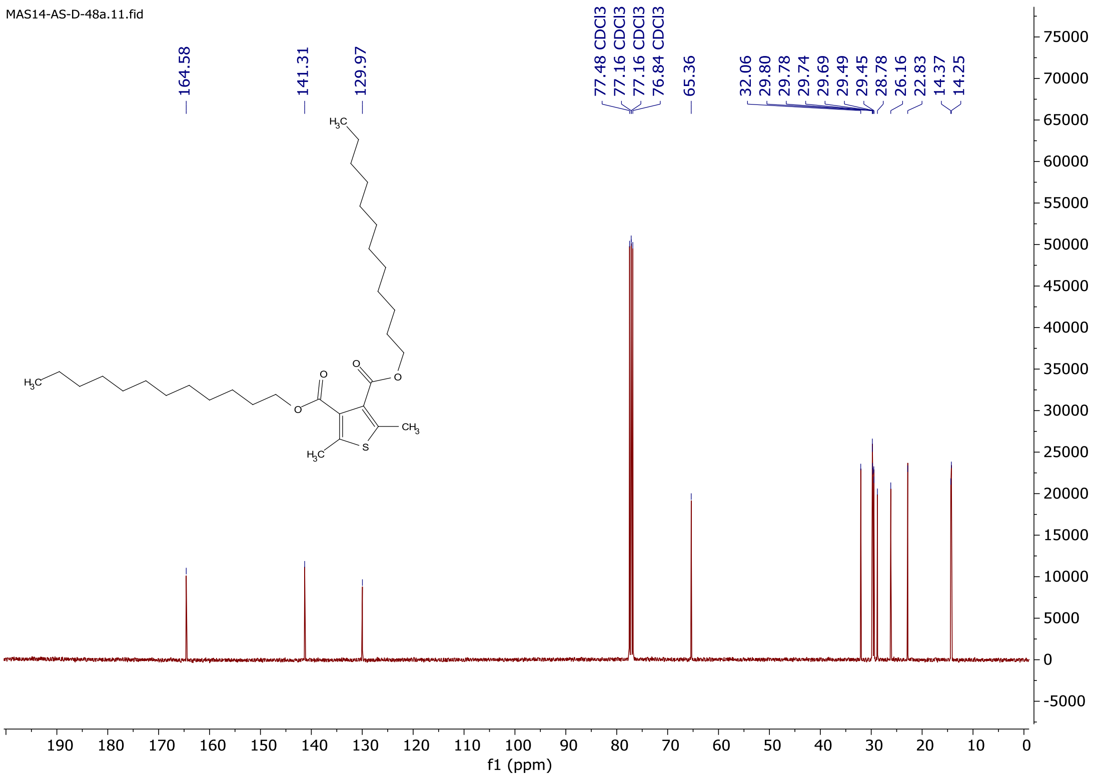

Figure S6: ¹³C-NMR spectrum of didodecyl 2,5-dimethylthiophene-3,4-dicarboxylate (**18a**)

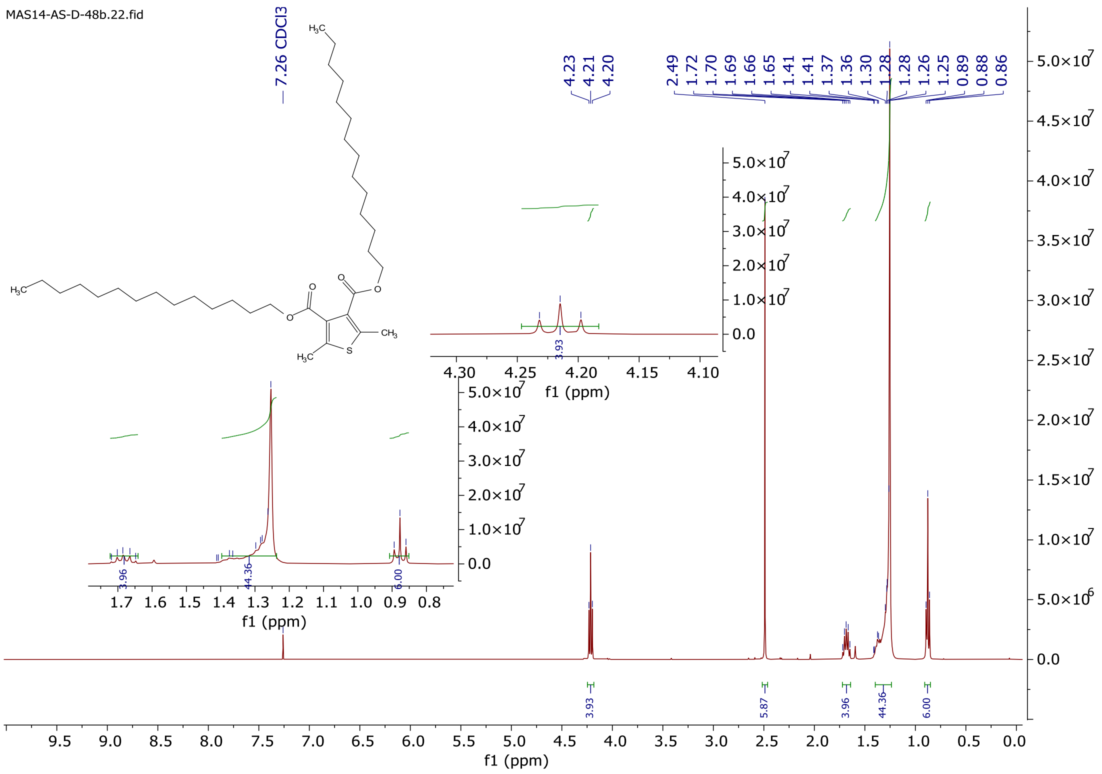

Figure S7: ^{1}H-NMR spectrum of ditetradecyl 2,5-dimethylthiophene-3,4-dicarboxylate (**18b**)

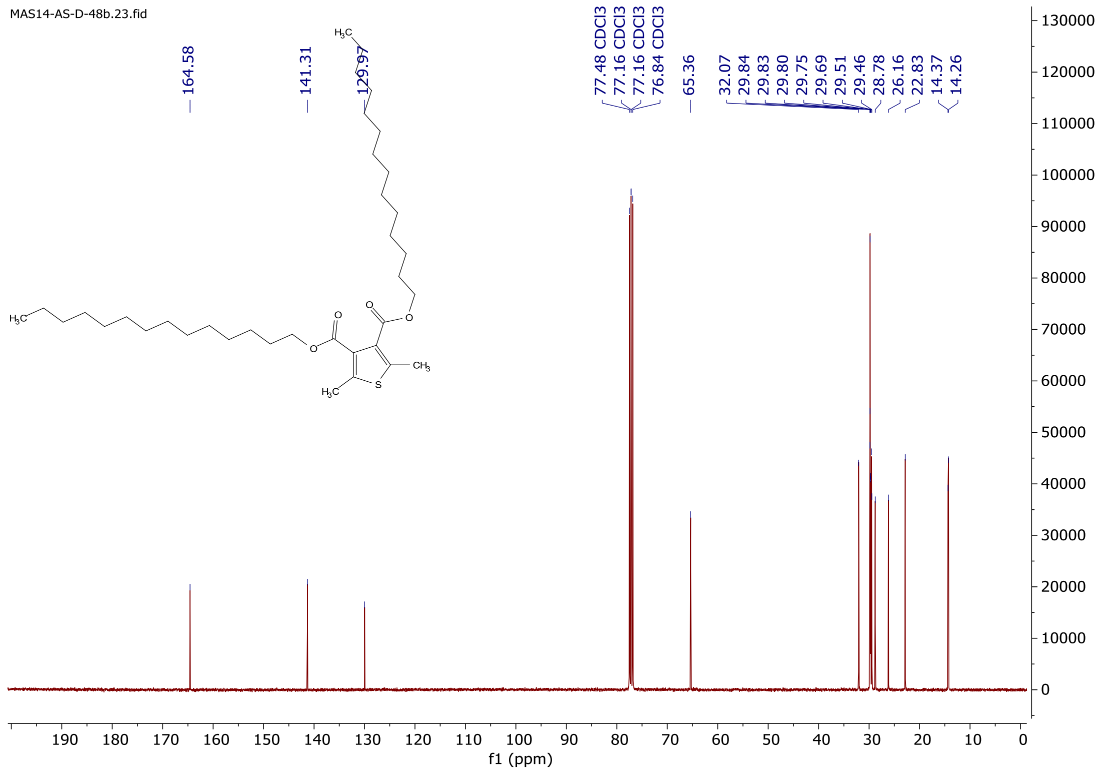

Figure S8: ^{13}C-NMR spectrum of ditetradecyl 2,5-dimethylthiophene-3,4-dicarboxylate (**18b**)

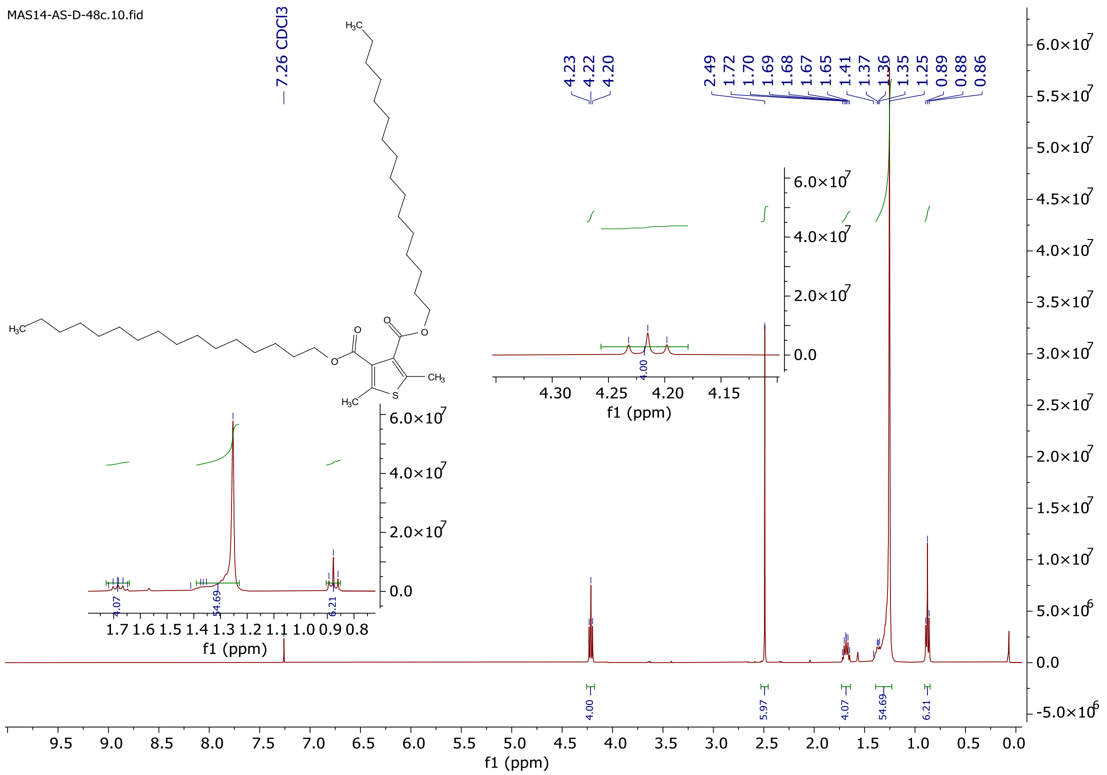

Figure S9: ^{1}H-NMR spectrum of dihexadecyl 2,5-dimethylthiophene-3,4-dicarboxylate (**18c**)

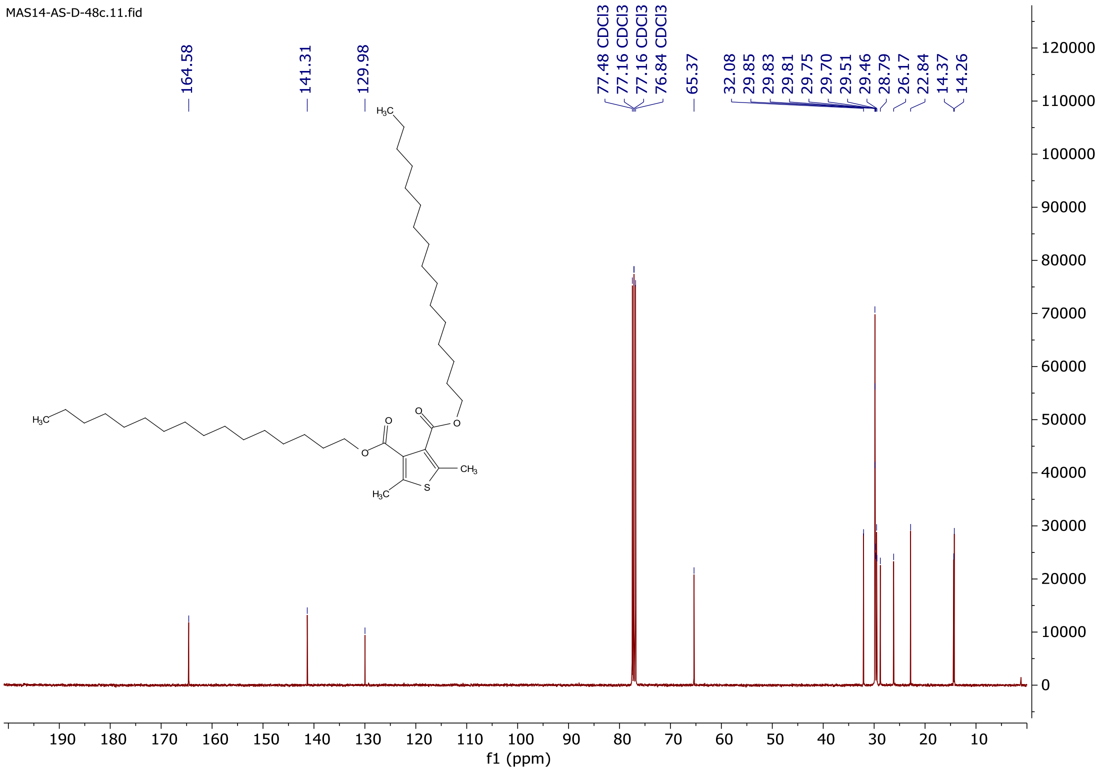

Figure S10: ^{13}C-NMR spectrum of dihexadecyl 2,5-dimethylthiophene-3,4-dicarboxylate (**18c**)

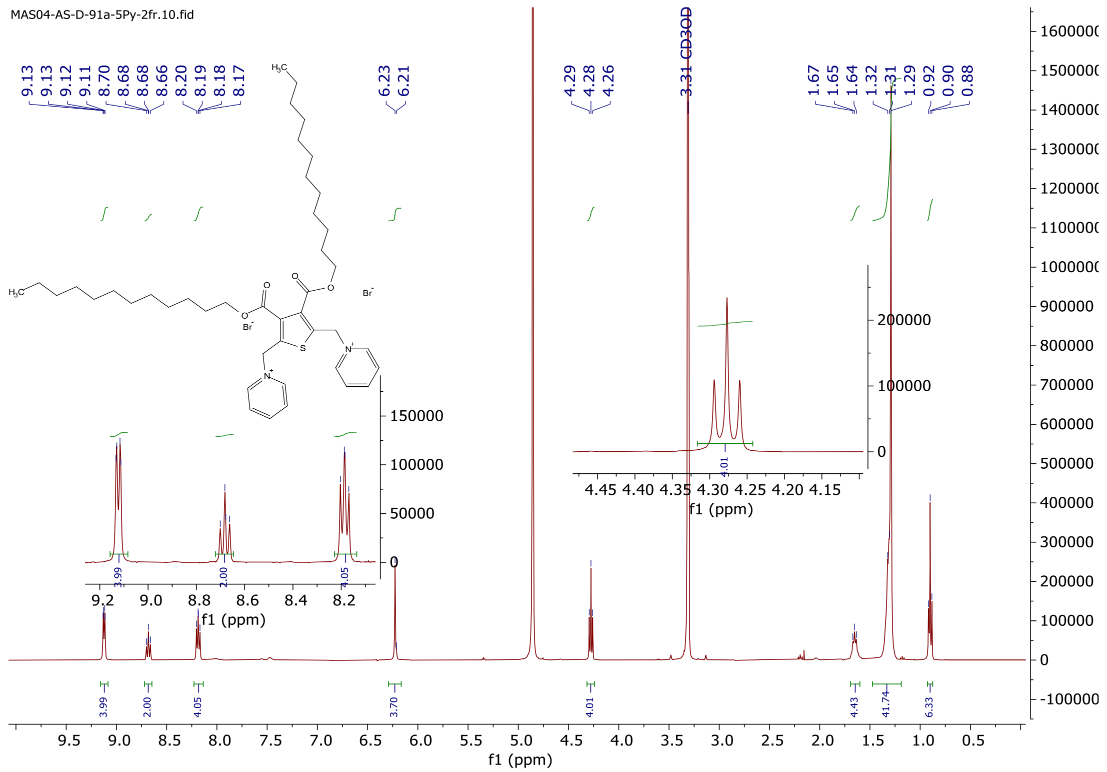

Figure S11: ^{1}H-NMR spectrum of 1,1'-((3,4-bis((dodecyloxy)carbonyl)thiophene-2,5-diyl)bis(methylene))bis(pyridin-1-ium) dibromide (**3a**)

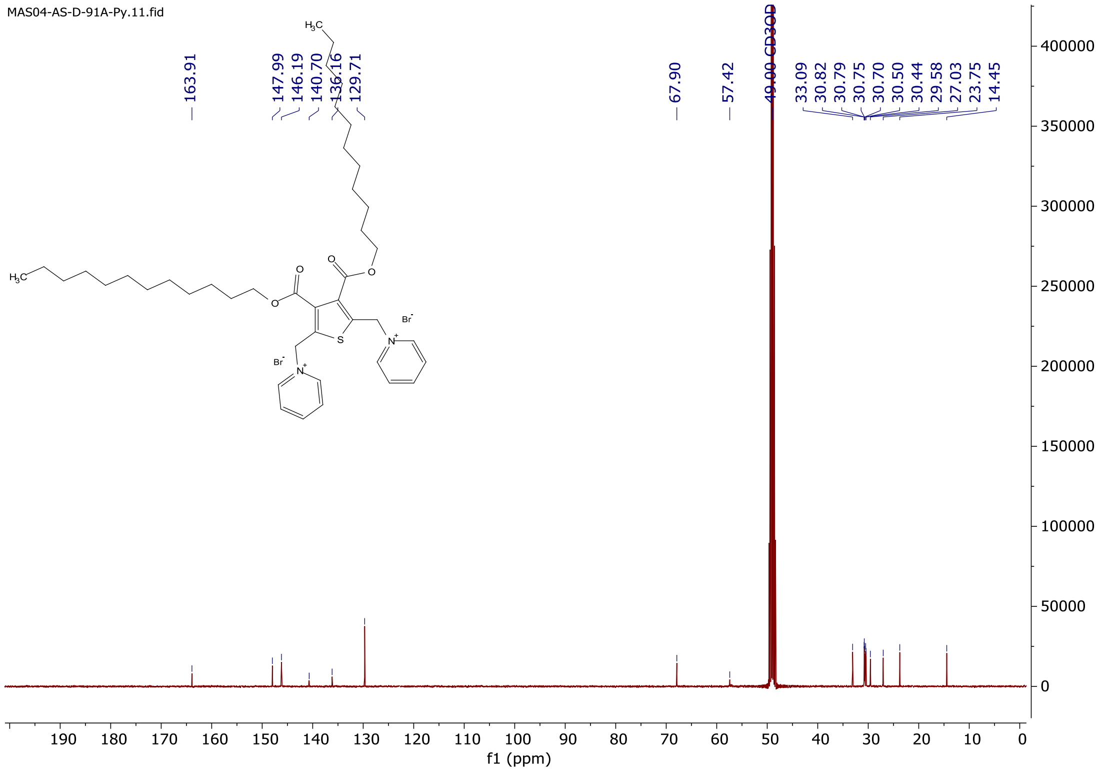

Figure S12: ^{13}C-NMR spectrum of 1,1'-((3,4-bis((dodecyloxy)carbonyl)thiophene-2,5-diyl)bis(methylene))bis(pyridin-1-ium) dibromide (**3a**)

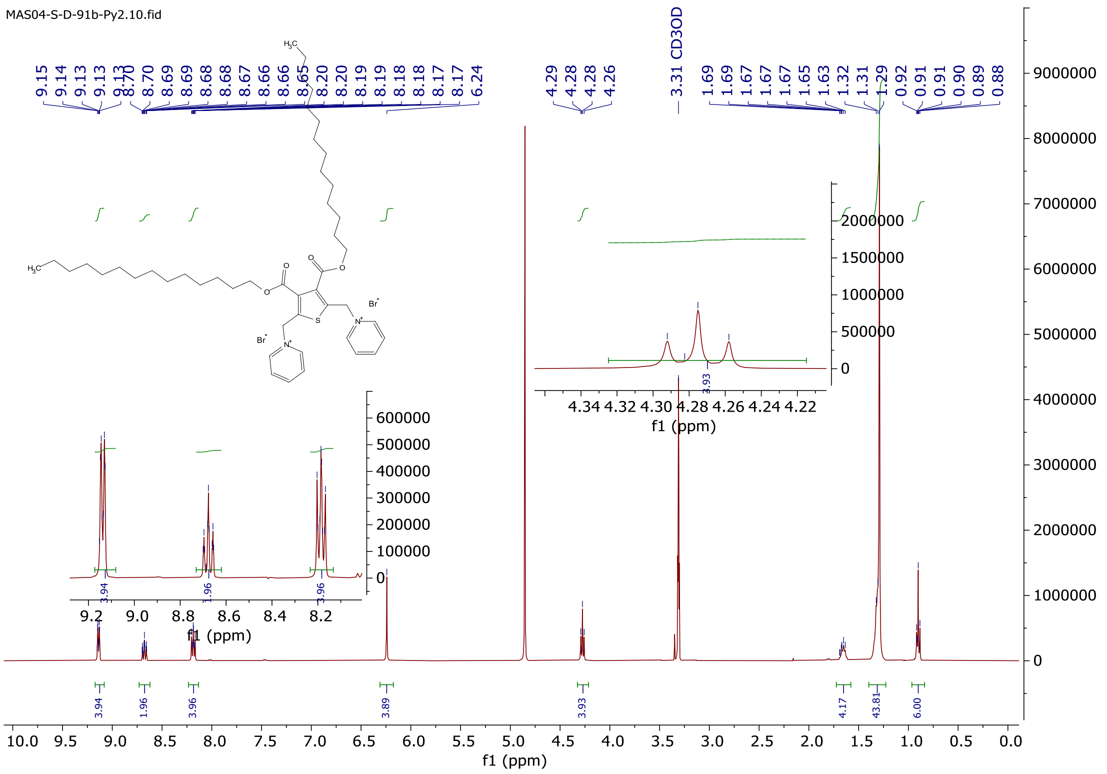

Figure S13: ^{1}H-NMR spectrum of 1,1'-((3,4-bis((tetradecyloxy)carbonyl)thiophene-2,5-diyl)bis(methylene))bis(pyridin-1-ium) dibromide (**3b**)

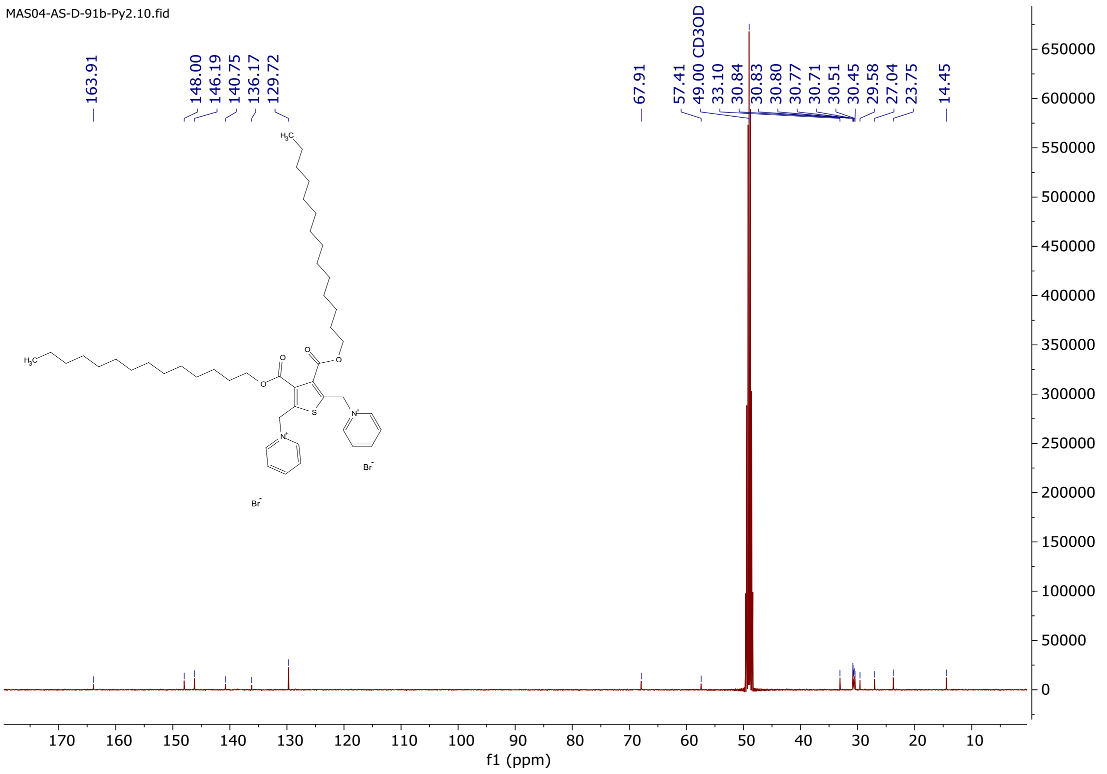

Figure S14: ^{13}C-NMR spectrum of 1,1'-((3,4-bis((tetradecyloxy)carbonyl)thiophene-2,5-diyl)bis(methylene))bis(pyridin-1-ium) dibromide (**3b**)

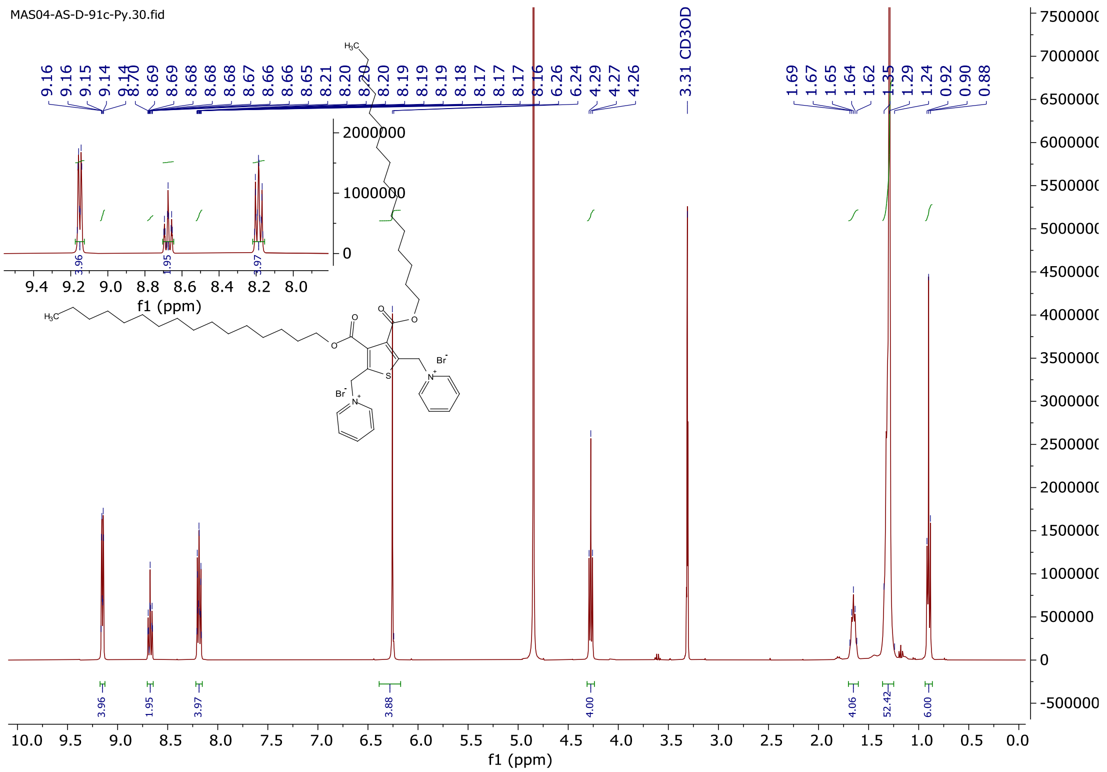

Figure S15: ^{1}H-NMR spectrum of 1,1'-((3,4-bis((hexadecyloxy)carbonyl)thiophene-2,5-diyl)bis(methylene))bis(pyridin-1-ium) dibromide (**3c**)

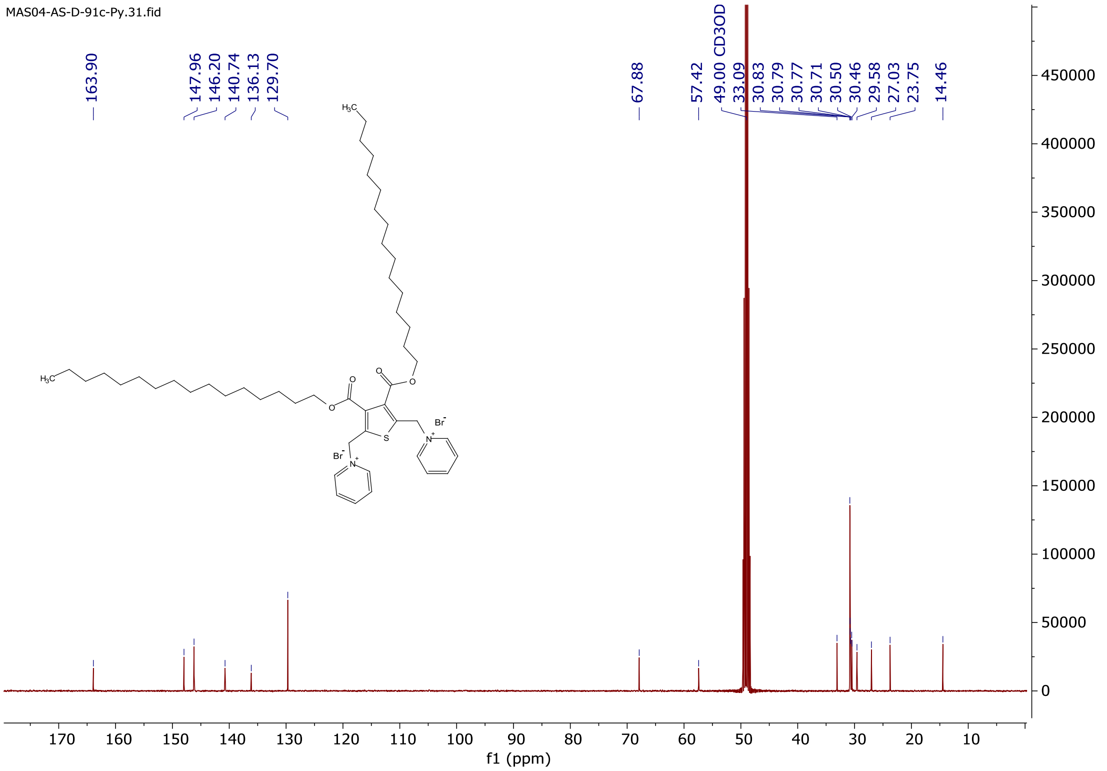

Figure S16: ^{13}C-NMR spectrum of 1,1'-((3,4-bis((hexadecyloxy)carbonyl)thiophene-2,5-diyl)bis(methylene))bis(pyridin-1-ium) dibromide (**3c**)

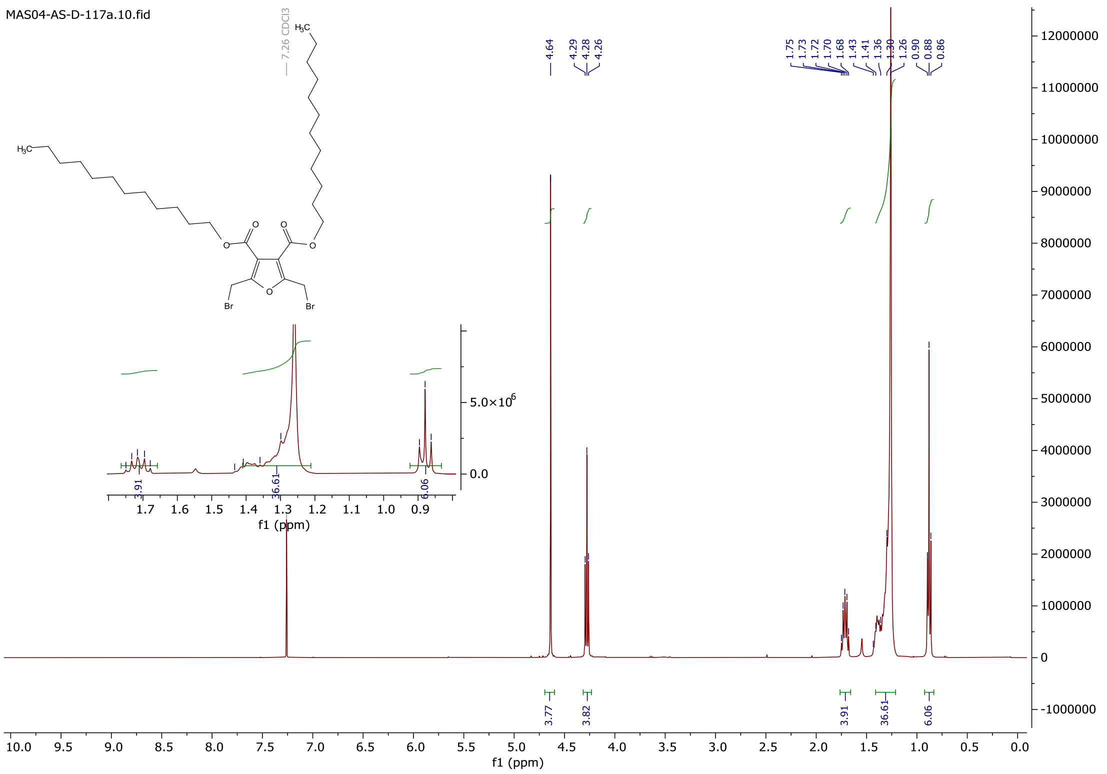

Figure S17: ^{1}H-NMR spectrum of didodecyl 2,5-bis(bromomethyl)furan-3,4-dicarboxylate (**22a**)

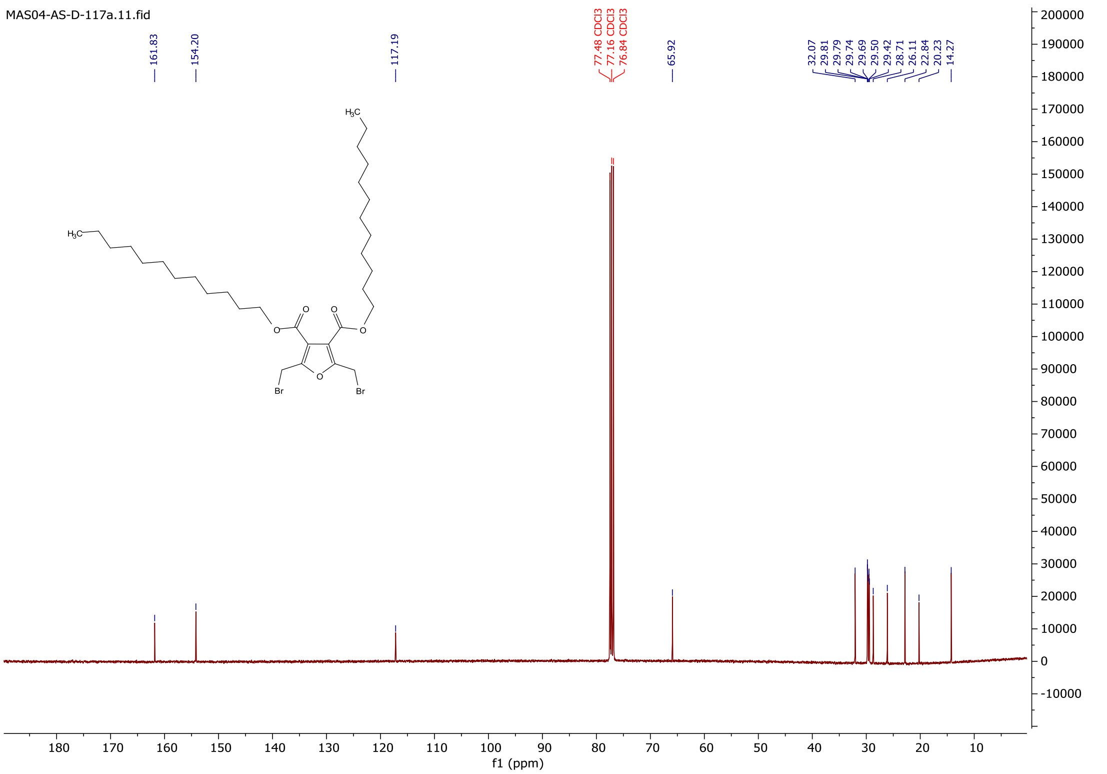

Figure S18: ^{13}C-NMR spectrum of didodecyl 2,5-bis(bromomethyl)furan-3,4-dicarboxylate (**22a**)

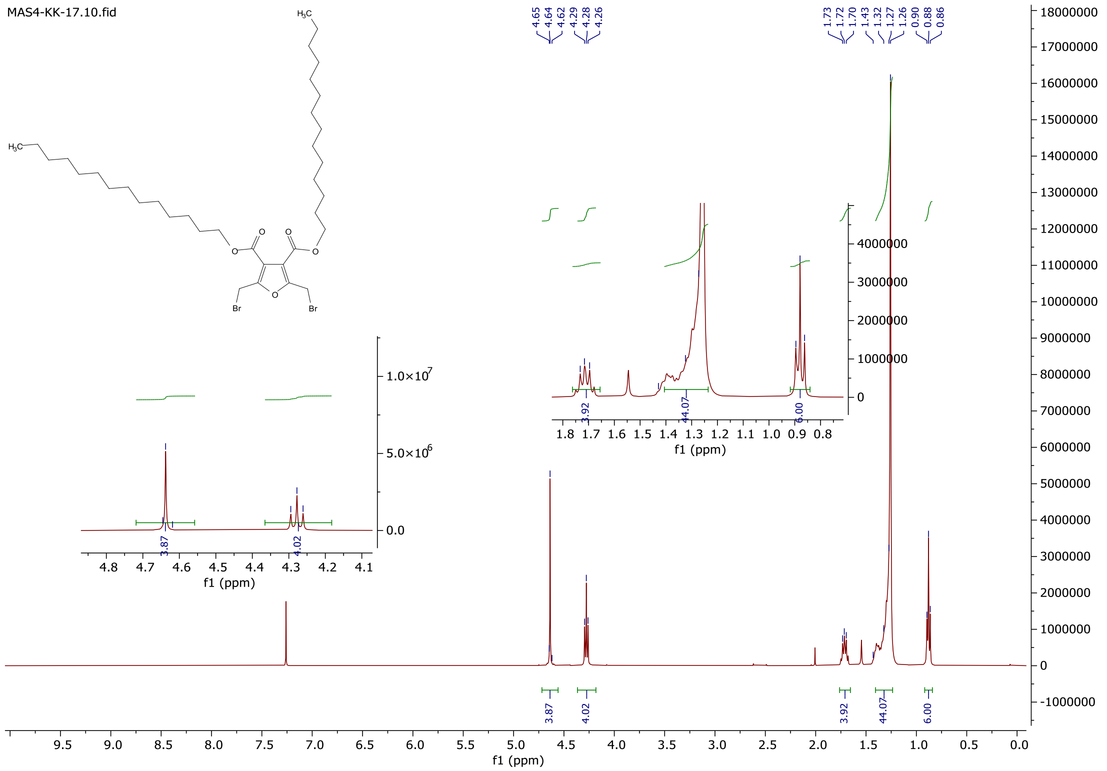

Figure S19: ^{1}H-NMR spectrum of ditetradecyl 2,5-bis(bromomethyl)furan-3,4-dicarboxylate (**22b**)

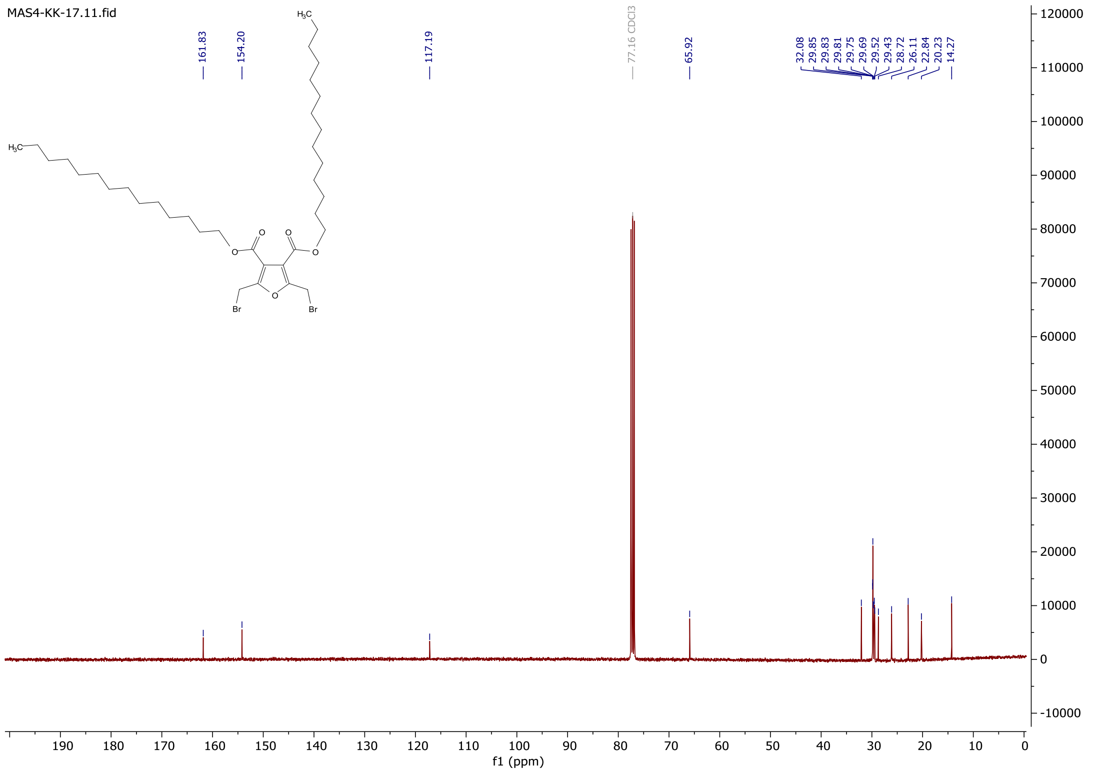

Figure S20: ^{13}C-NMR spectrum of ditetradecyl 2,5-bis(bromomethyl)furan-3,4-dicarboxylate (**22b**)

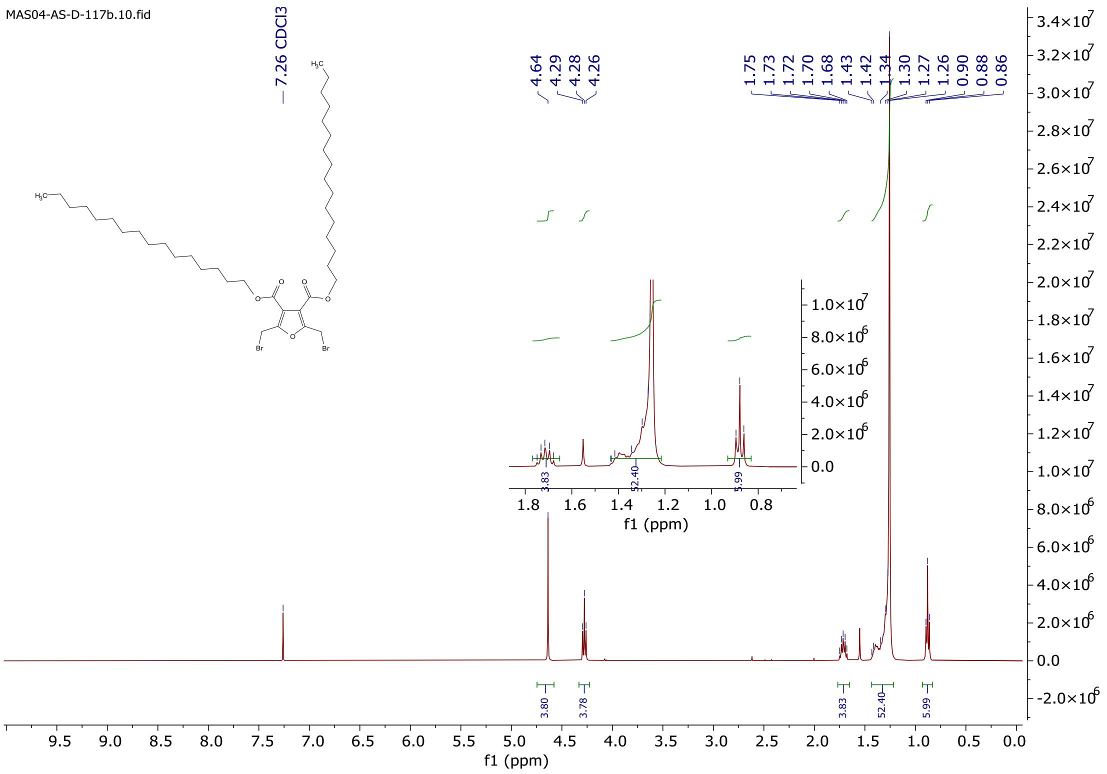

Figure S21: ^{1}H-NMR spectrum of dihexadecyl 2,5-bis(bromomethyl)furan-3,4-dicarboxylate (**22c**)

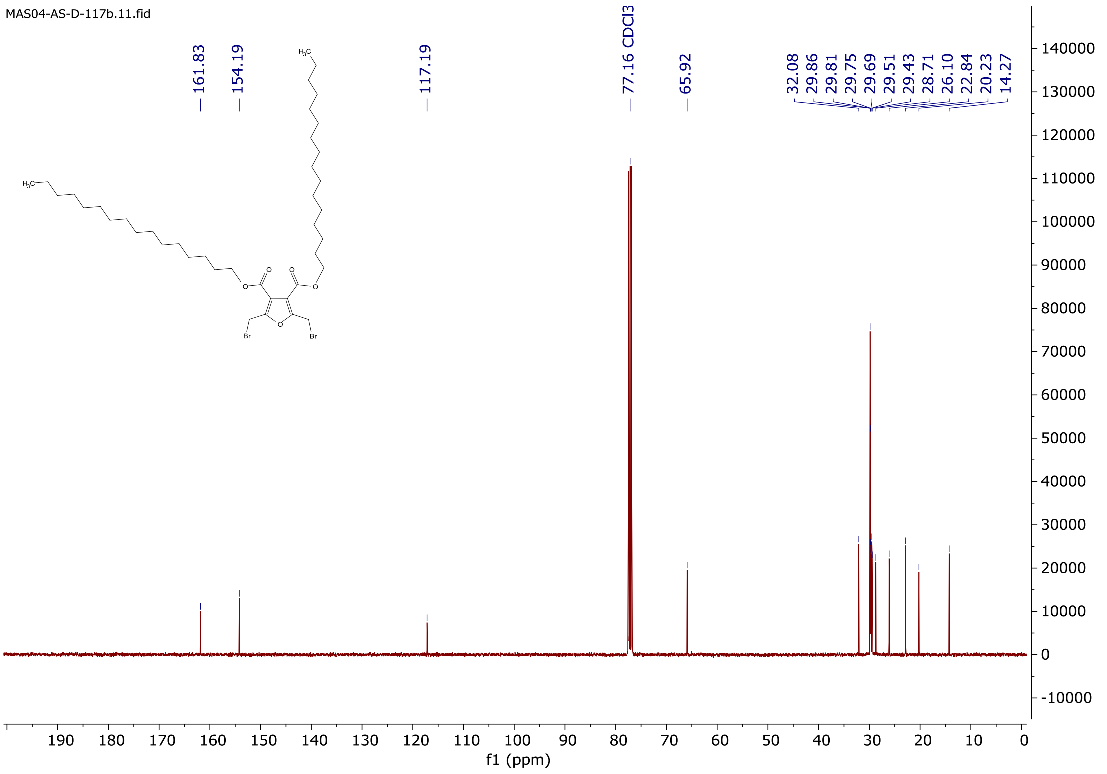

Figure S22: ^{13}C-NMR spectrum of dihexadecyl 2,5-bis(bromomethyl)furan-3,4-dicarboxylate (**22c**)

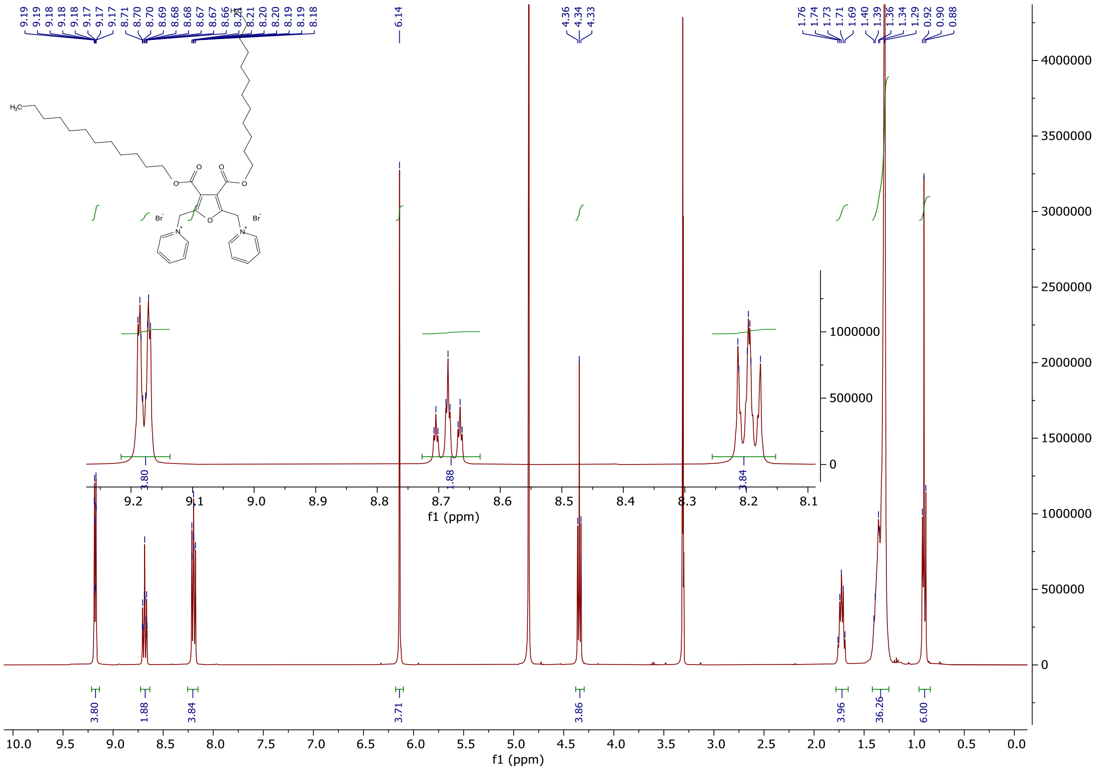

Figure S23: ^{1}H-NMR spectrum of 1,1'-((3,4-bis((dodecyloxy)carbonyl)furan-2,5-diyl)bis(methylene))bis(pyridin-1-ium) dibromide (**4a**)

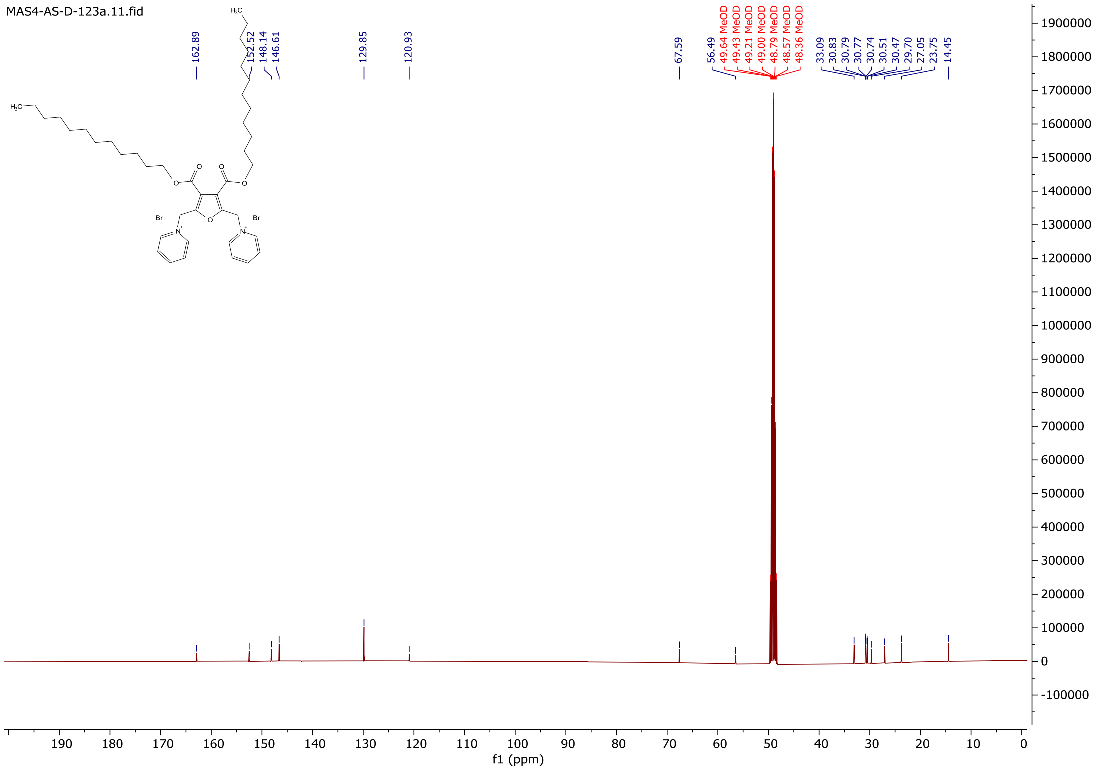

Figure S24: ^{13}C-NMR spectrum of 1,1'-((3,4-bis((dodecyloxy)carbonyl)furan-2,5-diyl)bis(methylene))bis(pyridin-1-ium) dibromide (**4a**)

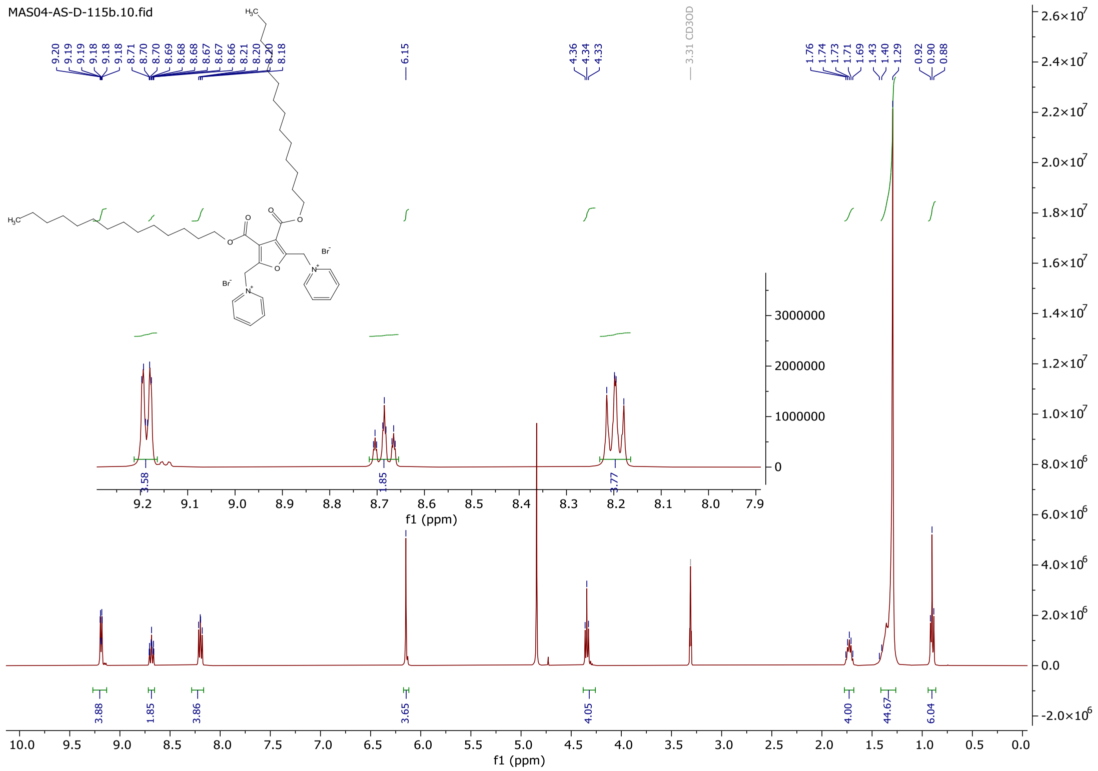

Figure S25: ^{1}H-NMR spectrum of 1,1'-((3,4-bis((tetradecyloxy)carbonyl)furan-2,5-diyl)bis(methylene))bis(pyridin-1-ium) dibromide (**4b**)

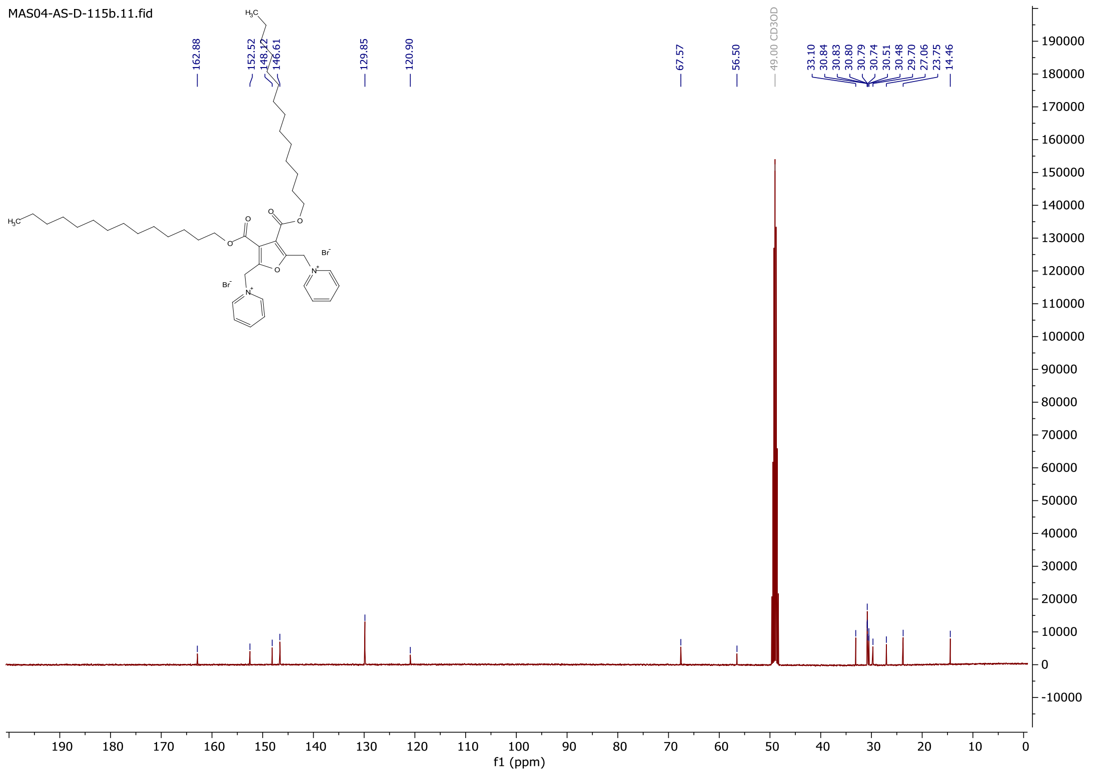

Figure S26: ^{13}C-NMR spectrum of 1,1'-((3,4-bs((tetradecyloxy)carbonyl)furan-2,5-diyl)bis(methylene))bis(pyridin-1-ium) dibromide (**4b**)

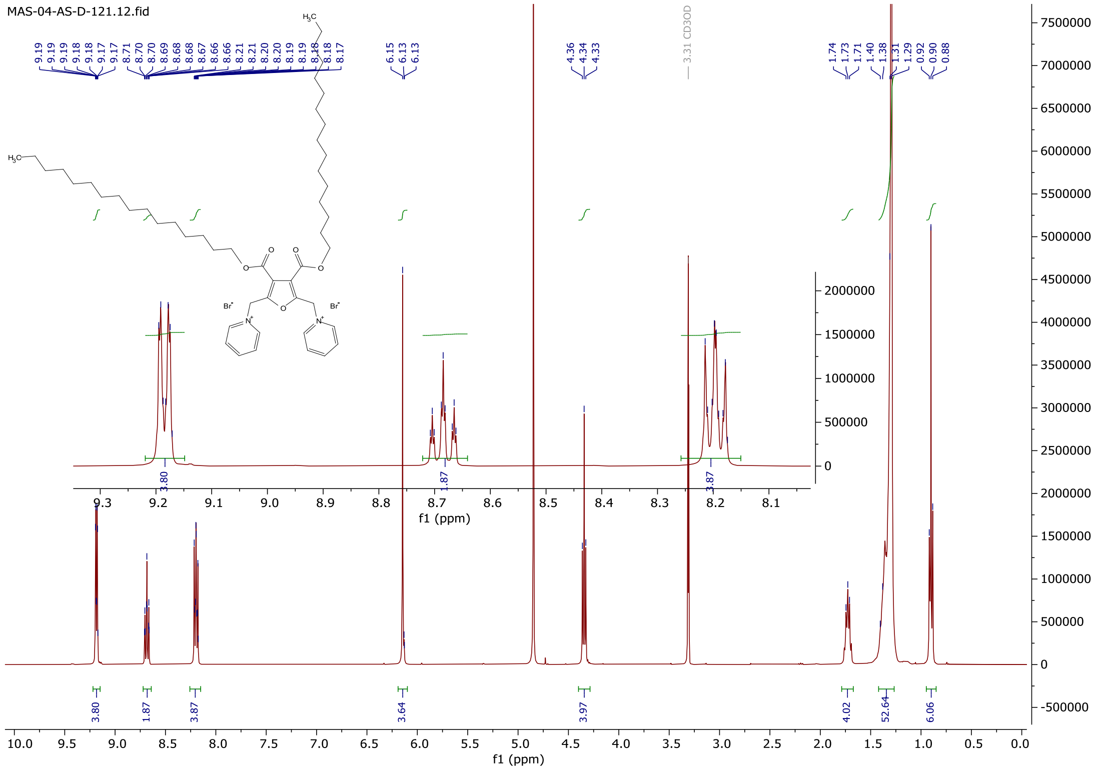

Figure S27: ^{1}H-NMR spectrum of 1,1'-((3,4-bis((hexadecyloxy)carbonyl)furan-2,5-diyl)bis(methylene))bis(pyridin-1-ium) dibromide (**4c**)

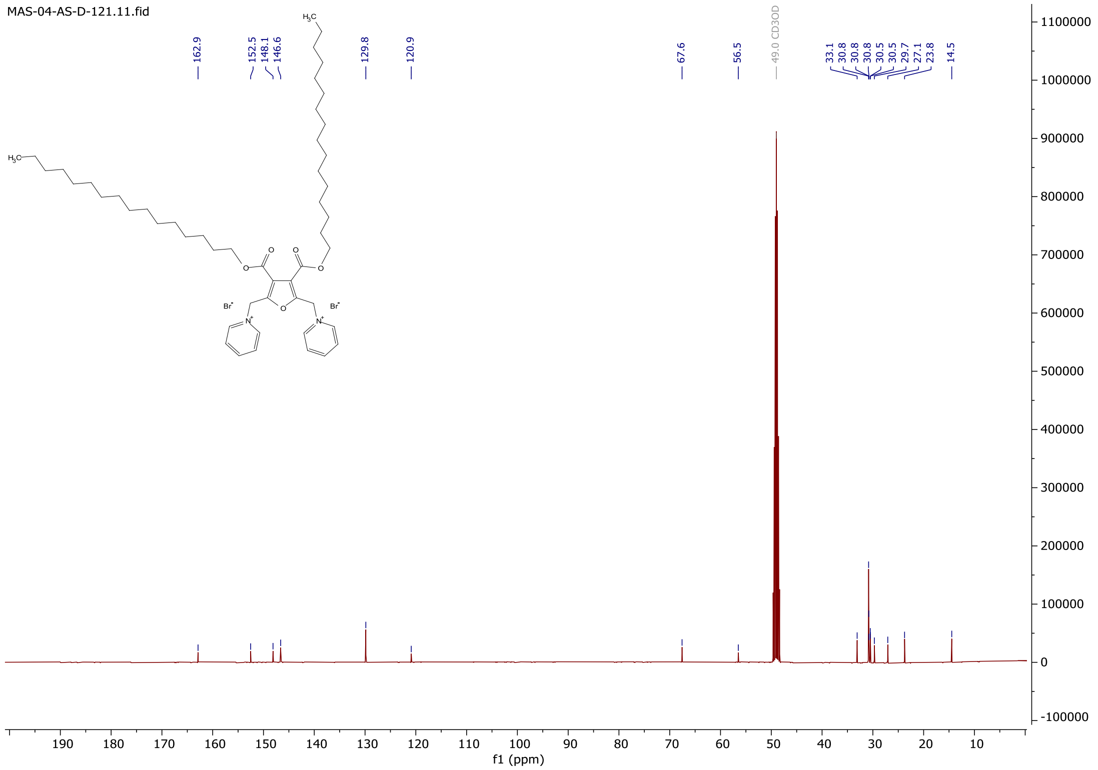

Figure S28: ^{13}C-NMR spectrum of 1,1'-((3,4-bis((hexadecyloxy)carbonyl)furan-2,5-diyl)bis(methylene))bis(pyridin-1-ium) dibromide (**4c**)

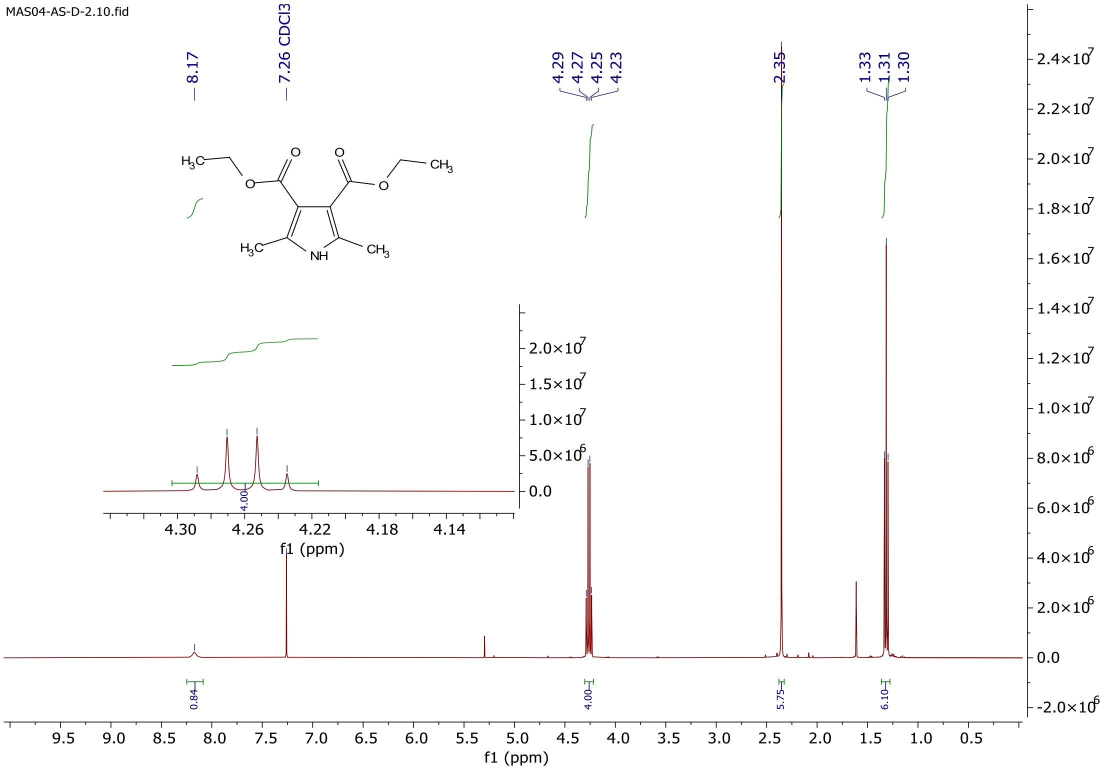

Figure S29: ^{1}H-NMR spectrum of diethyl 2,5-dimethyl-1*H*-pyrrole-3,4-dicarboxylate (**17**)

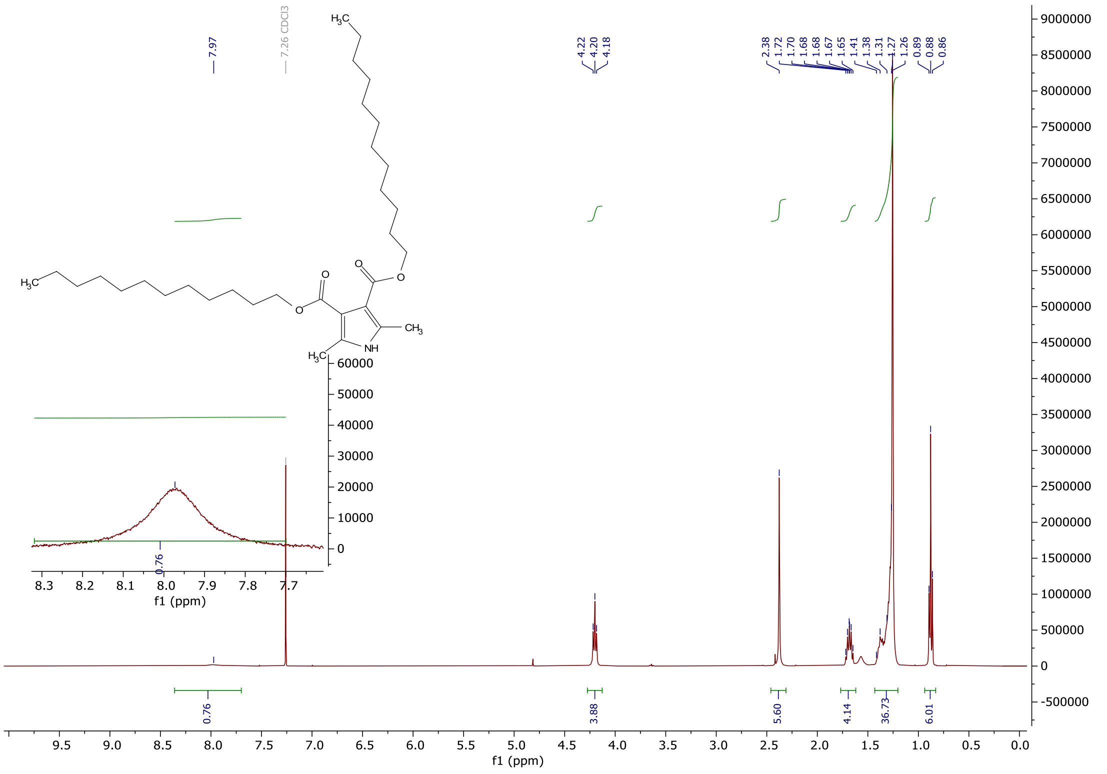

Figure S30: ^{1}H-NMR spectrum of didodecyl 2,5-dimethyl-1*H*-pyrrole-3,4-dicarboxylate (**20a**)

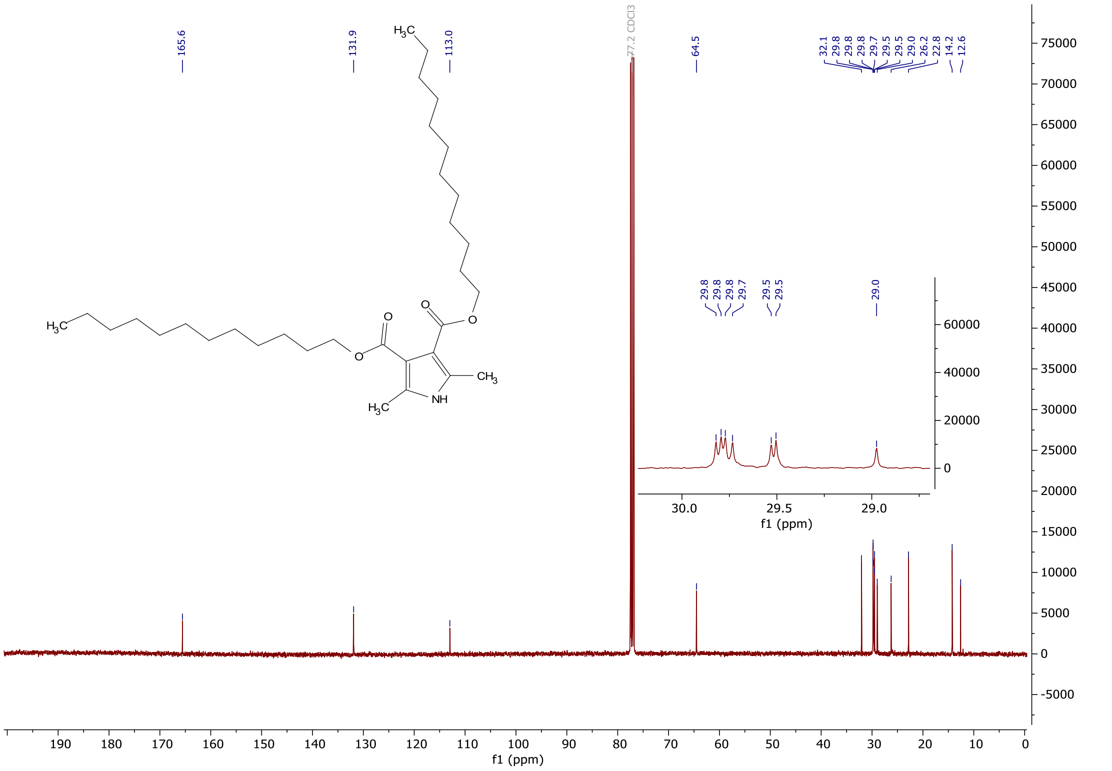

Figure S31: ^{13}C-NMR spectrum of didodecyl 2,5-dimethyl-1*H*-pyrrole-3,4-dicarboxylate (**20a**)

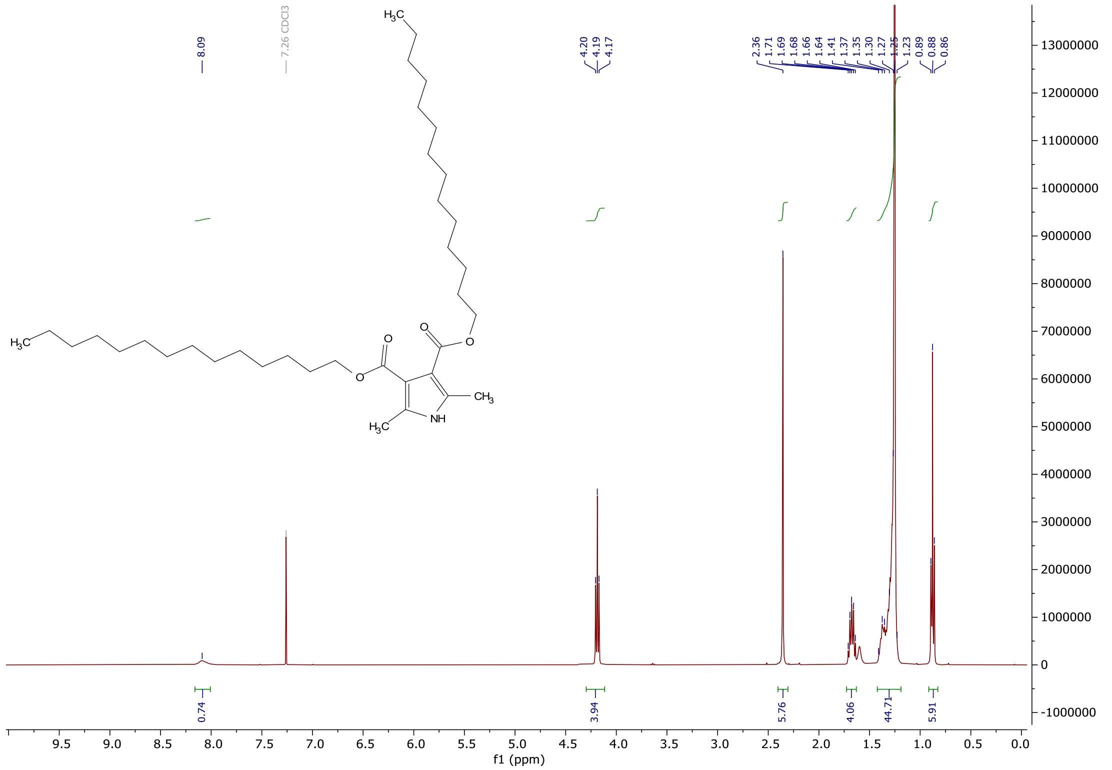

Figure S32: ^{1}H-NMR spectrum of ditetradecyl 2,5-dimethyl-1*H*-pyrrole-3,4-dicarboxylate (**20b**)

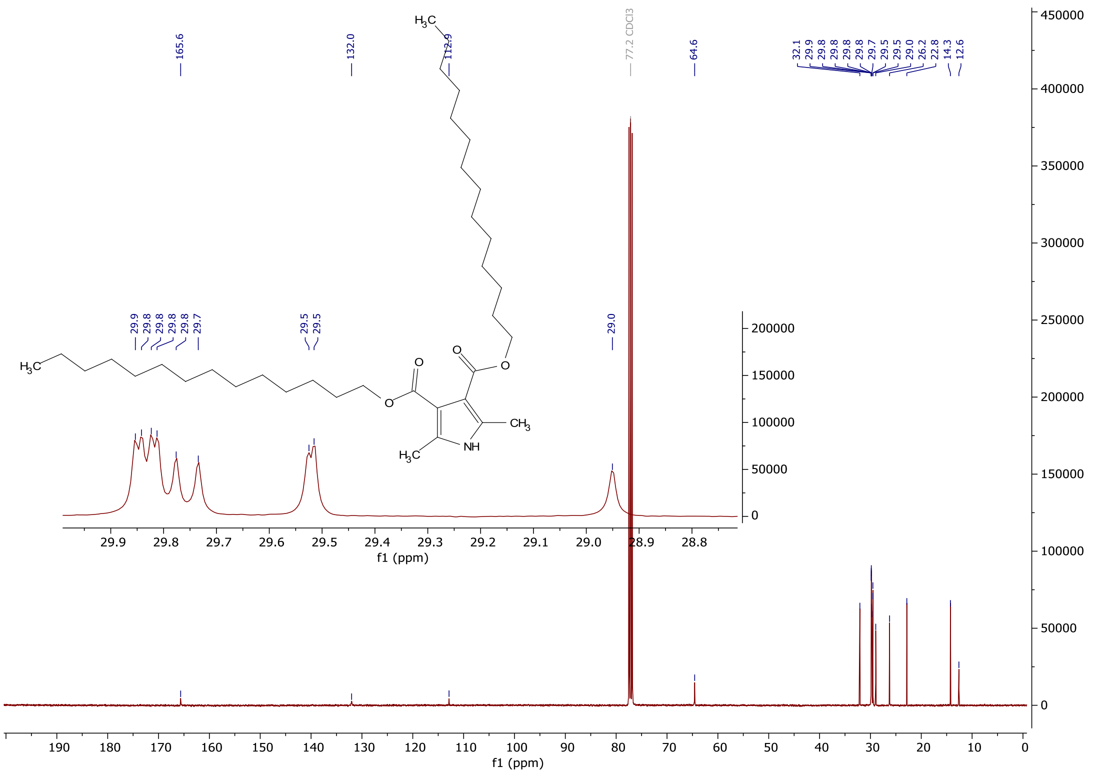

Figure S33: ^{13}C-NMR spectrum of ditetradecyl 2,5-dimethyl-1*H*-pyrrole-3,4-dicarboxylate (**20b**)

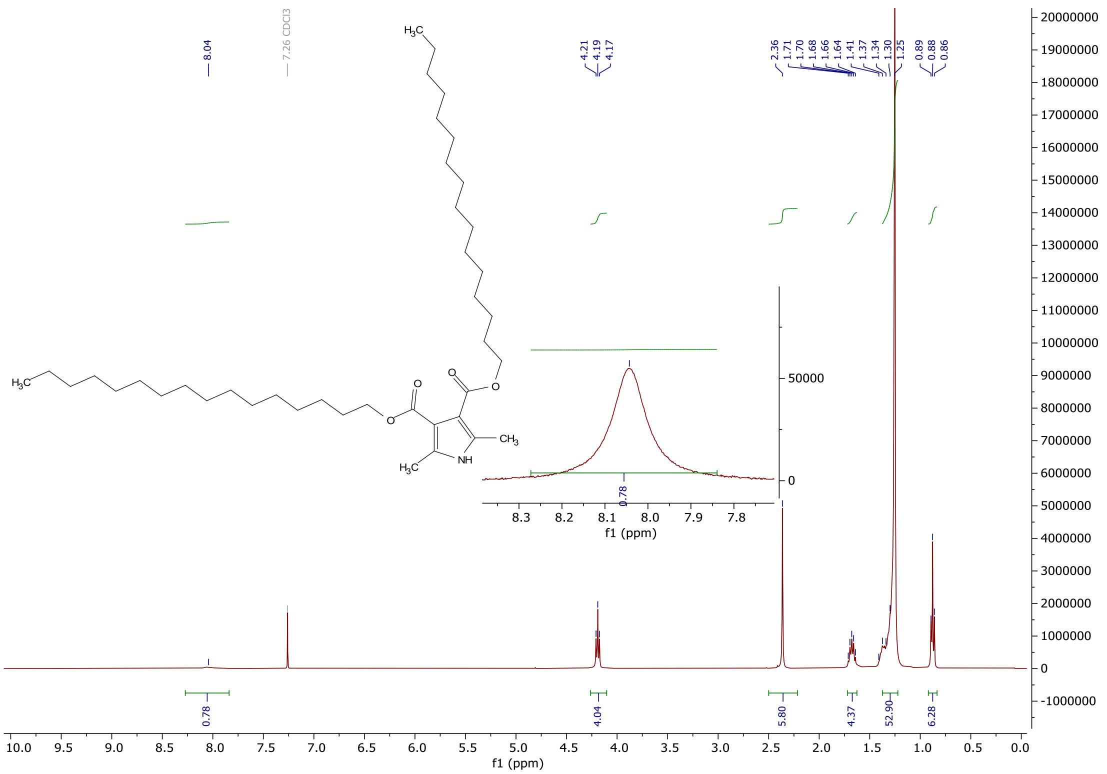

Figure S34: ^{1}H-NMR spectrum of dihexadecyl 2,5-dimethyl-1*H*-pyrrole-3,4-dicarboxylate (**20c**)

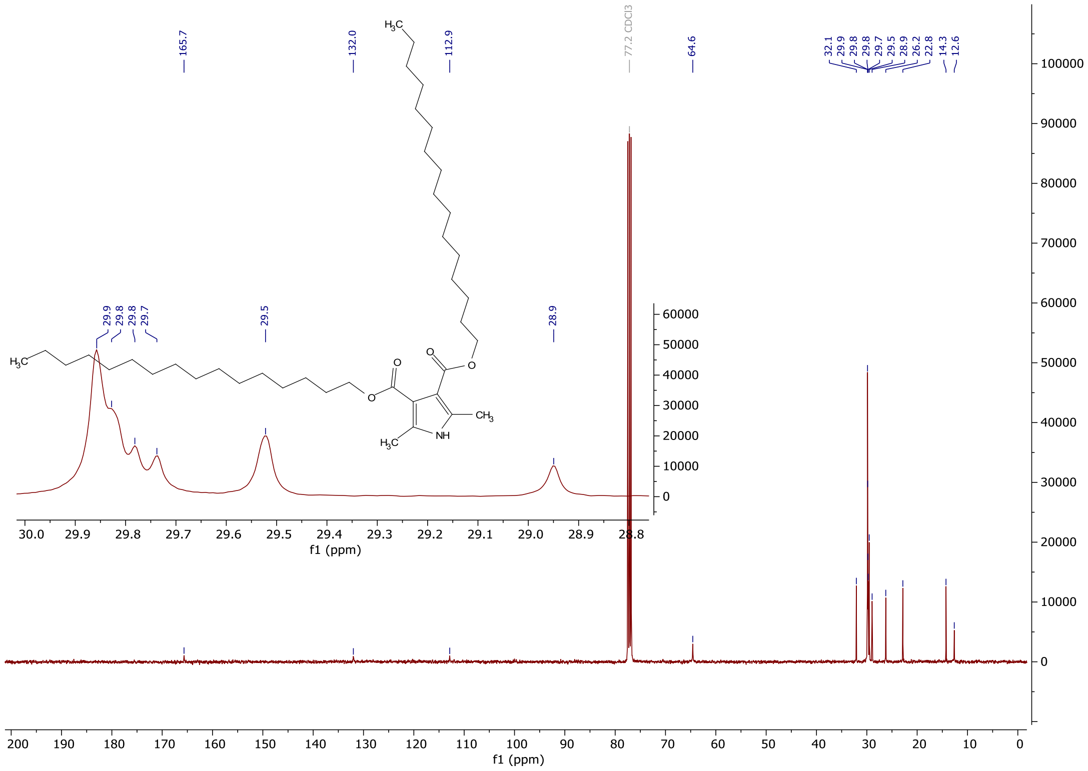

Figure S35: ^{13}C-NMR spectrum of dihexadecyl 2,5-dimethyl-1*H*-pyrrole-3,4-dicarboxylate (**20c**)

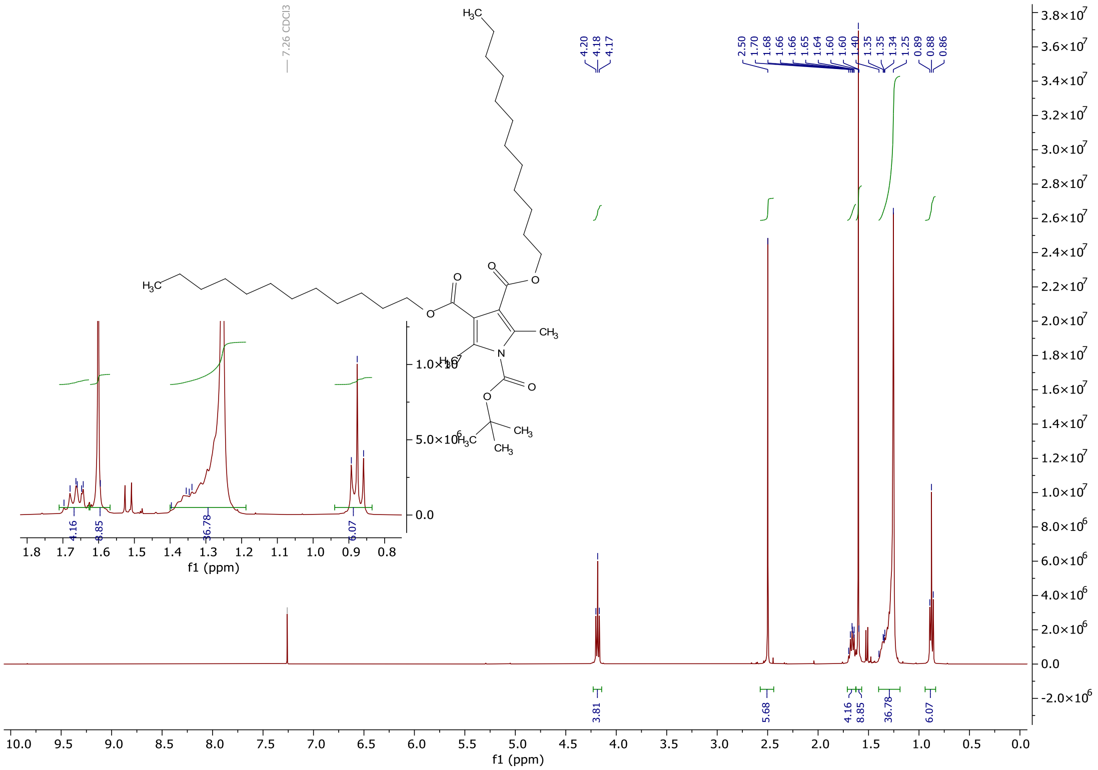

Figure S36: ^{1}H-NMR spectrum of 1-(tert-butyl) 3,4-didodecyl 2,5-dimethyl-1*H*-pyrrole-1,3,4-tricarboxylate (**20’a**)

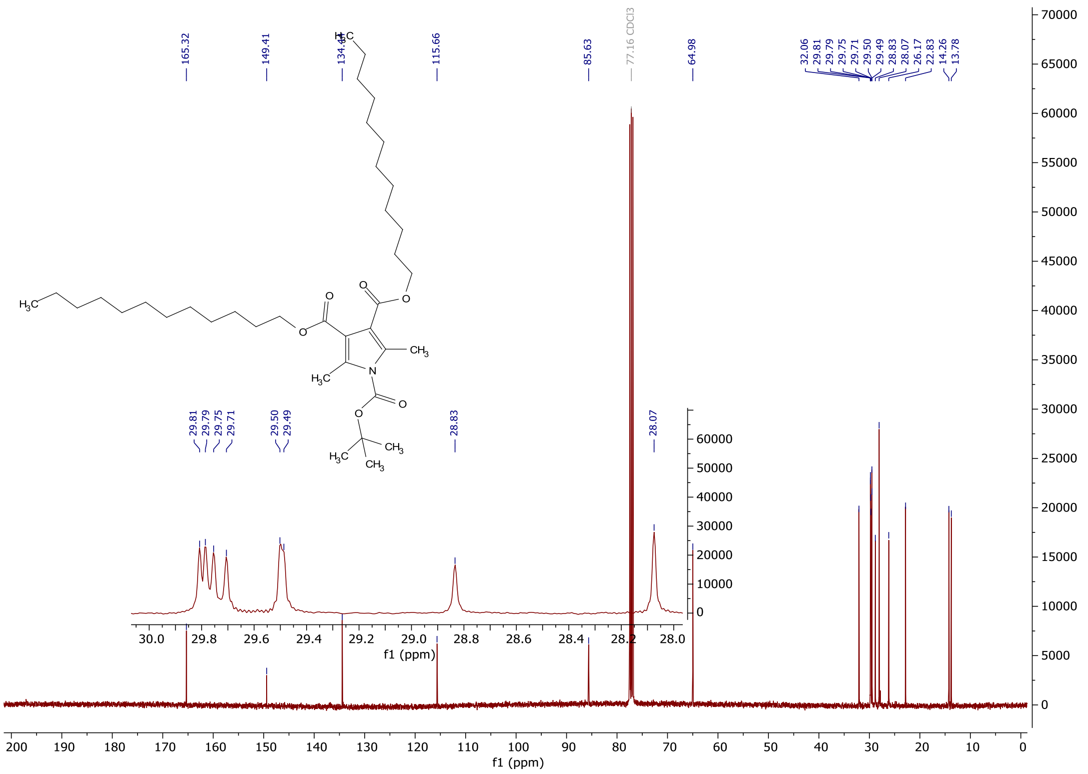

Figure S37: ^{13}C-NMR spectrum of 1-(tert-butyl) 3,4-didodecyl 2,5-dimethyl-1*H*-pyrrole-1,3,4-tricarboxylate (**20'a**)

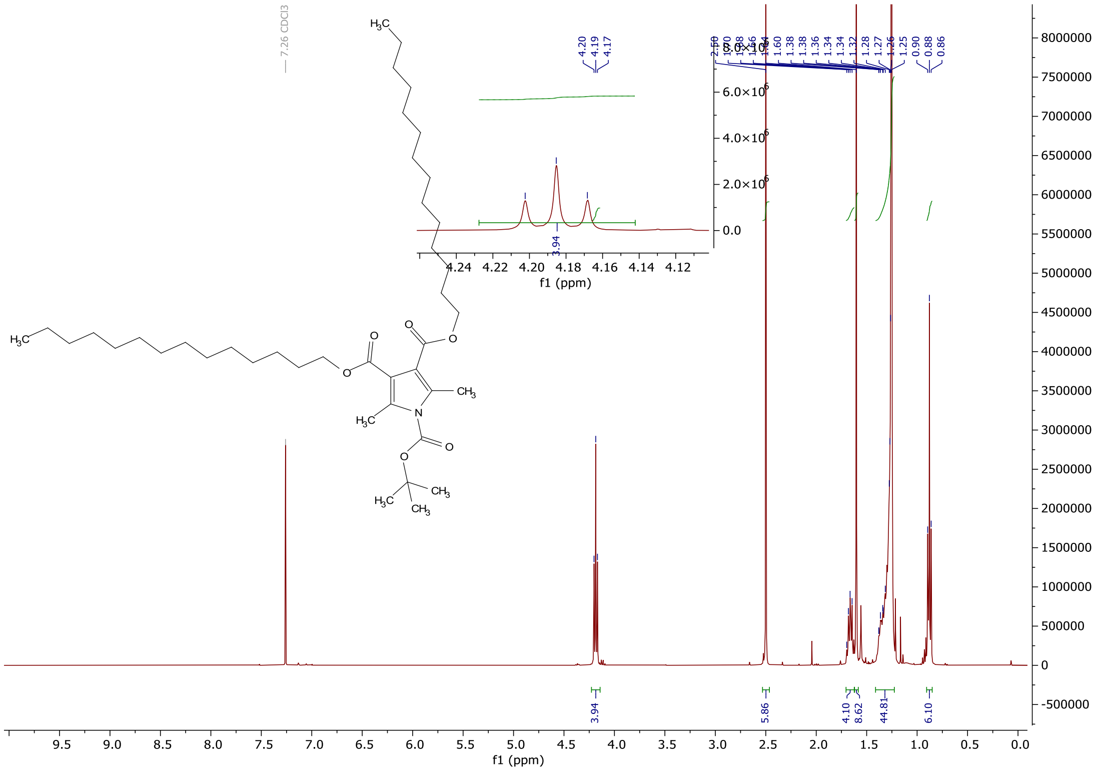

Figure S38: ^{1}H-NMR spectrum of 1-(tert-butyl) 3,4-ditetradecyl 2,5-dimethyl-1*H*-pyrrole-1,3,4-tricarboxylate (**20'b**)

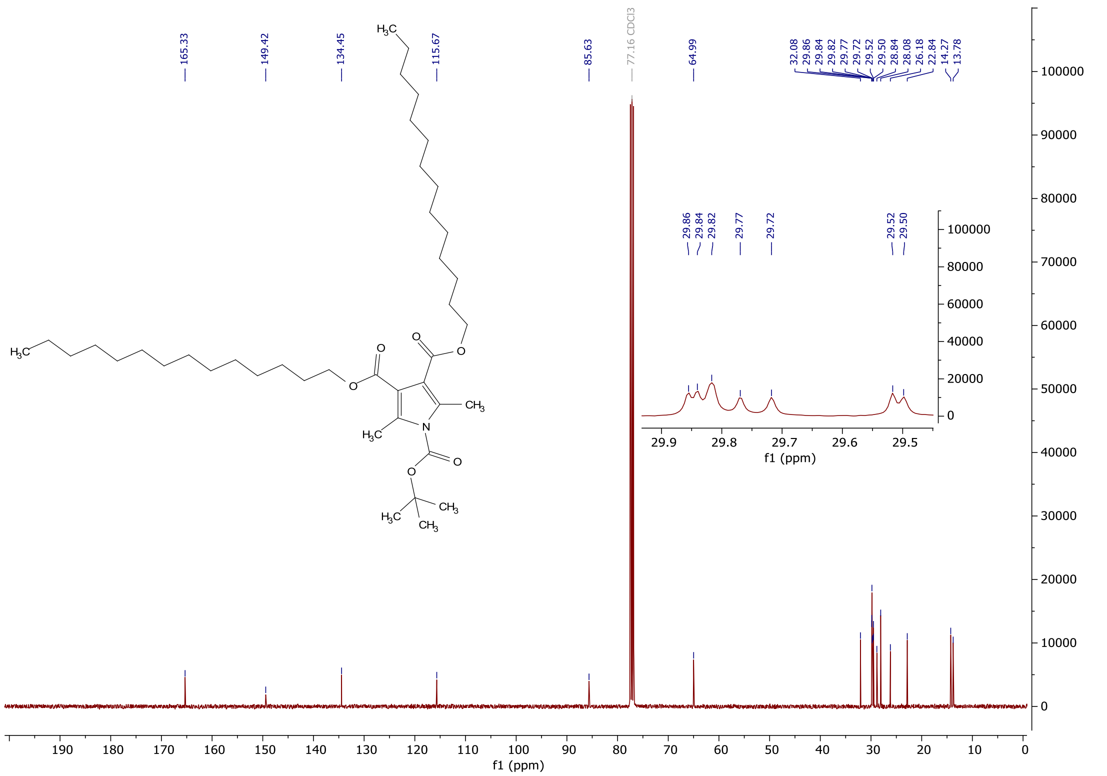

Figure S39: ^{13}C-NMR spectrum of 1-(tert-butyl) 3,4-ditetradecyl 2,5-dimethyl-1*H*-pyrrole-1,3,4-tricarboxylate (**20’b**)

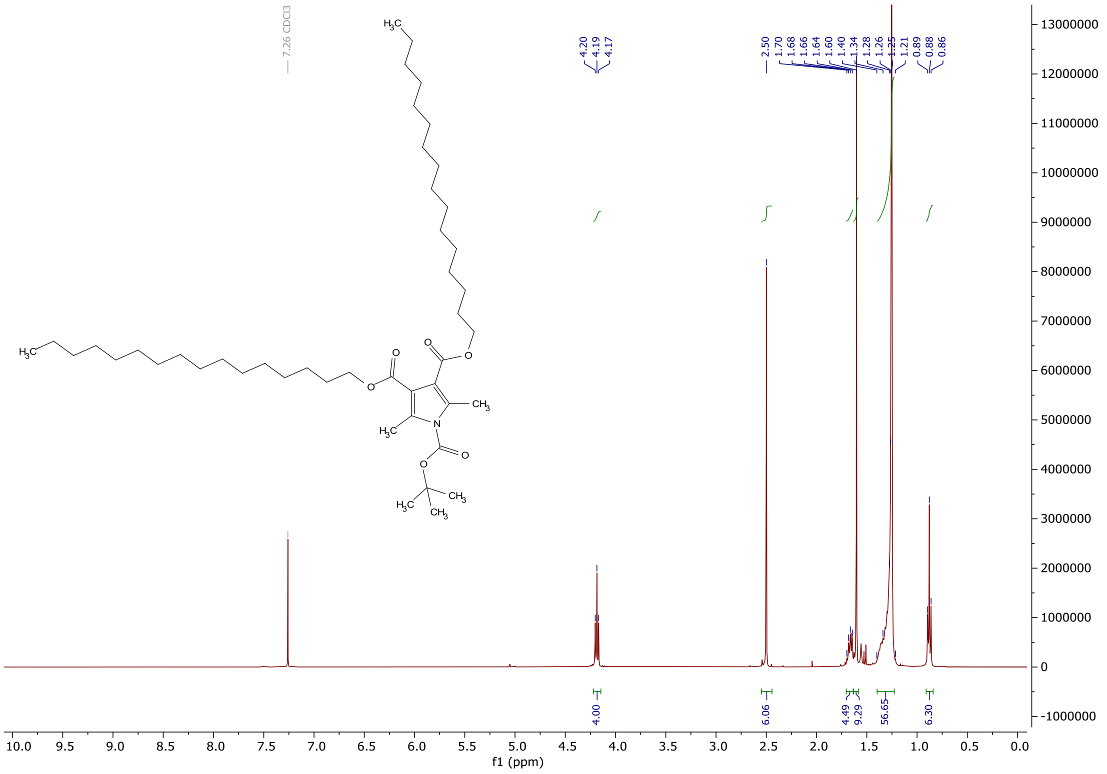

Figure S40: ^{1}H-NMR spectrum of 1-(tert-butyl) 3,4-dihexadecyl 2,5-dimethyl-1*H*-pyrrole-1,3,4-tricarboxylate (**20’c**)

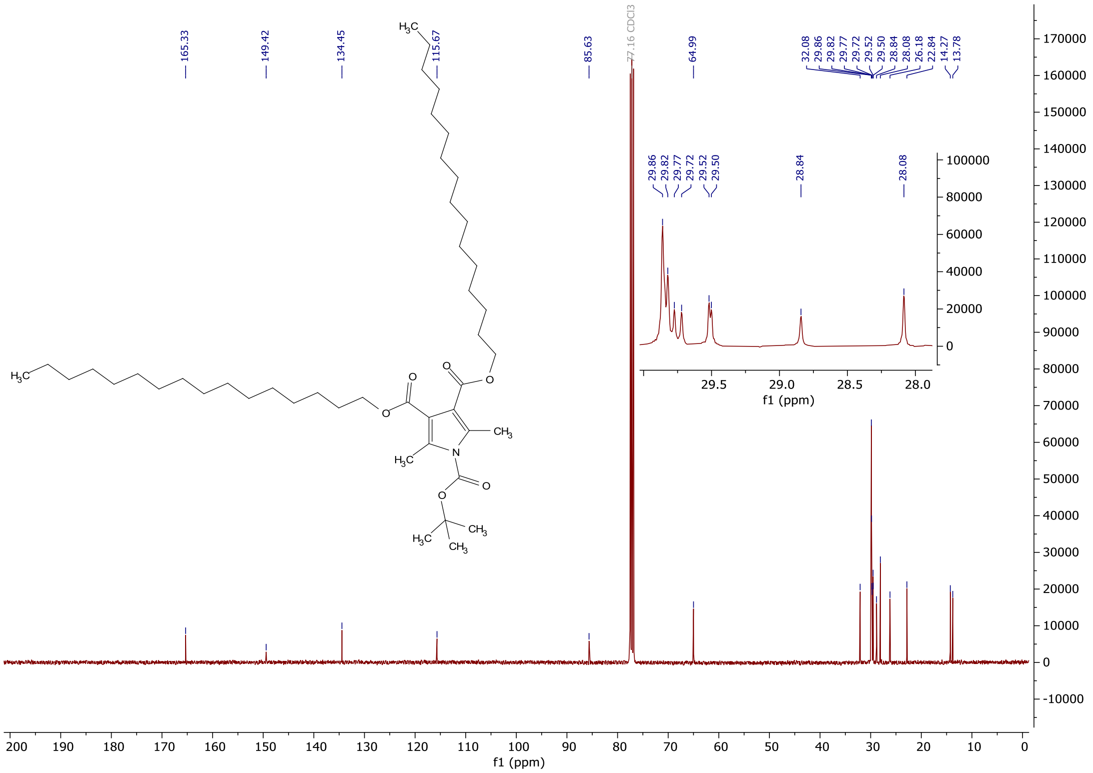

Figure S41: ^{13}C-NMR spectrum of 1-(tert-butyl) 3,4-dihexadecyl 2,5-dimethyl-1*H*-pyrrole-1,3,4-tricarboxylate (**20’c**)

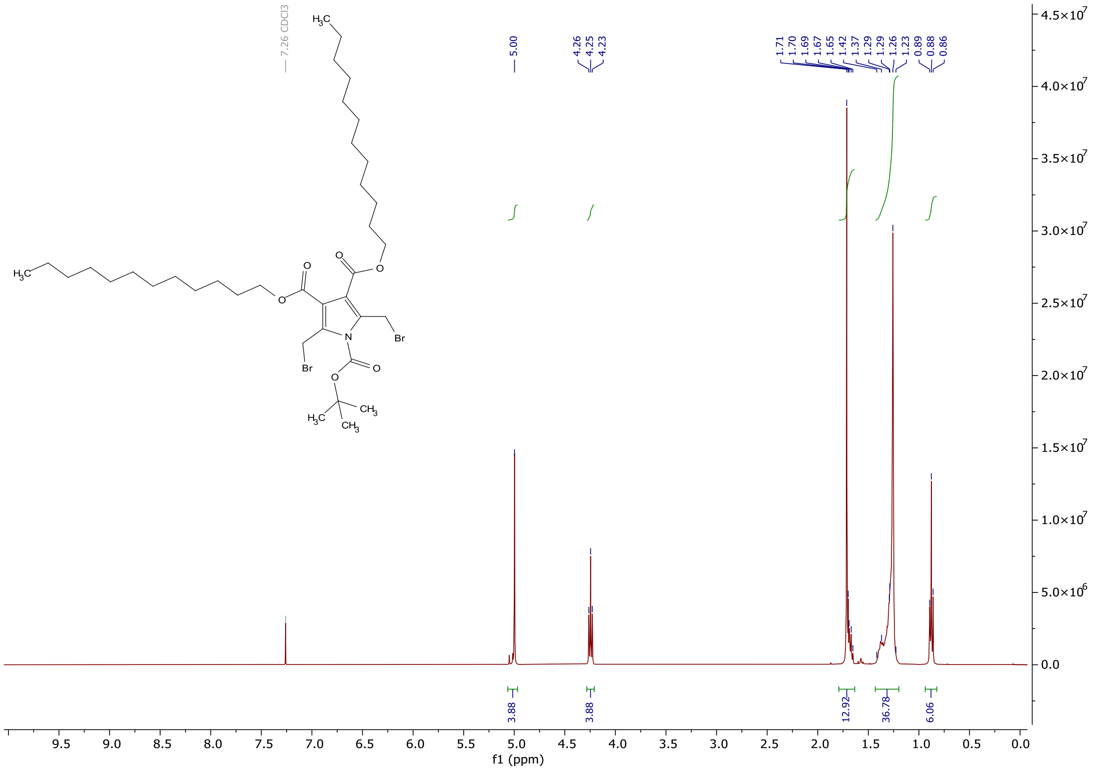

Figure S42: ^{1}H-NMR spectrum of 1-(*tert*-butyl) 3,4-didodecyl 2,5-bis(bromomethyl)-1*H*-pyrrole-1,3,4-tricarboxylate (**23’a**)

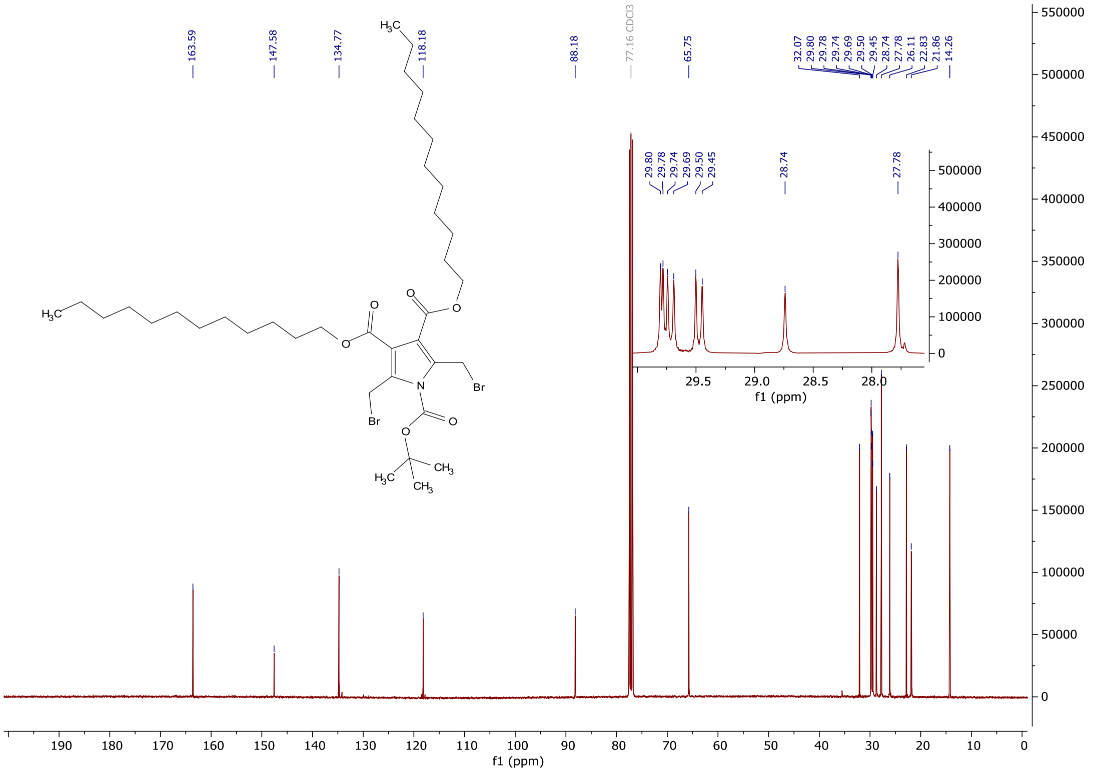

Figure S43: ^{13}C-NMR spectrum of 1-(*tert*-butyl) 3,4-didodecyl 2,5-bis(bromomethyl)-1*H*-pyrrole-1,3,4-tricarboxylate (**23'a**)

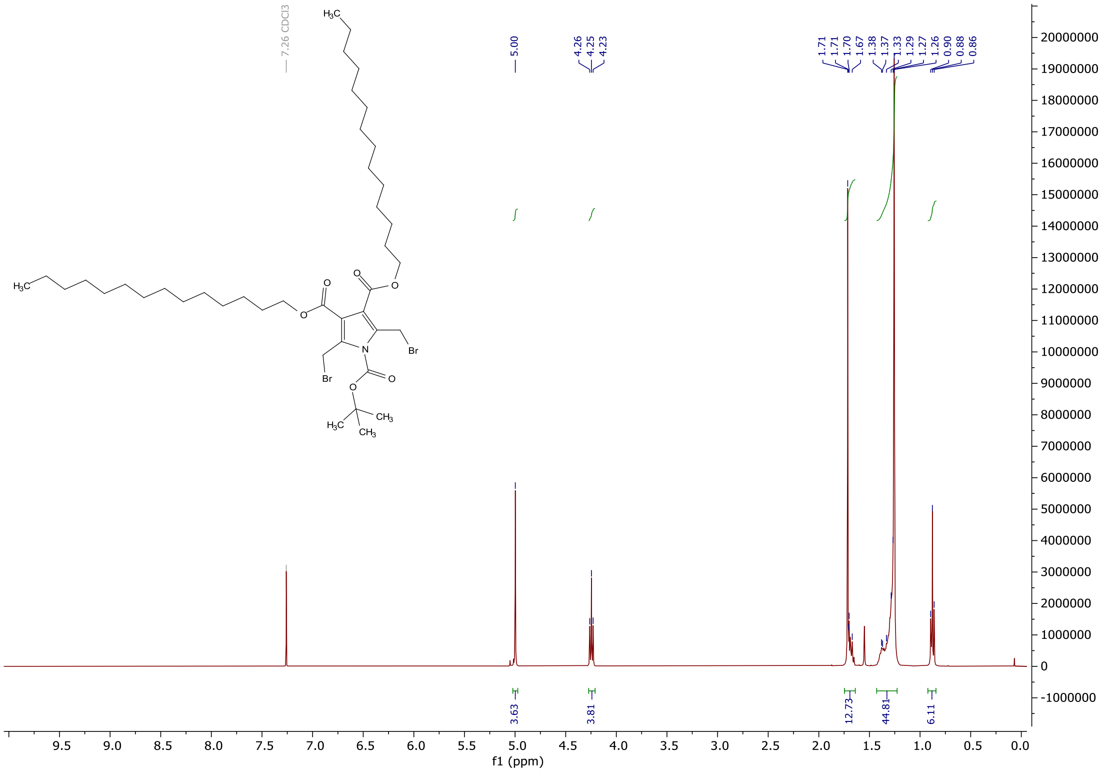

Figure S44: ^{1}H-NMR spectrum of 1-(*tert*-butyl) 3,4-ditetradecyl 2,5-bis(bromomethyl)-1*H*-pyrrole-1,3,4-tricarboxylate (**23'b**)

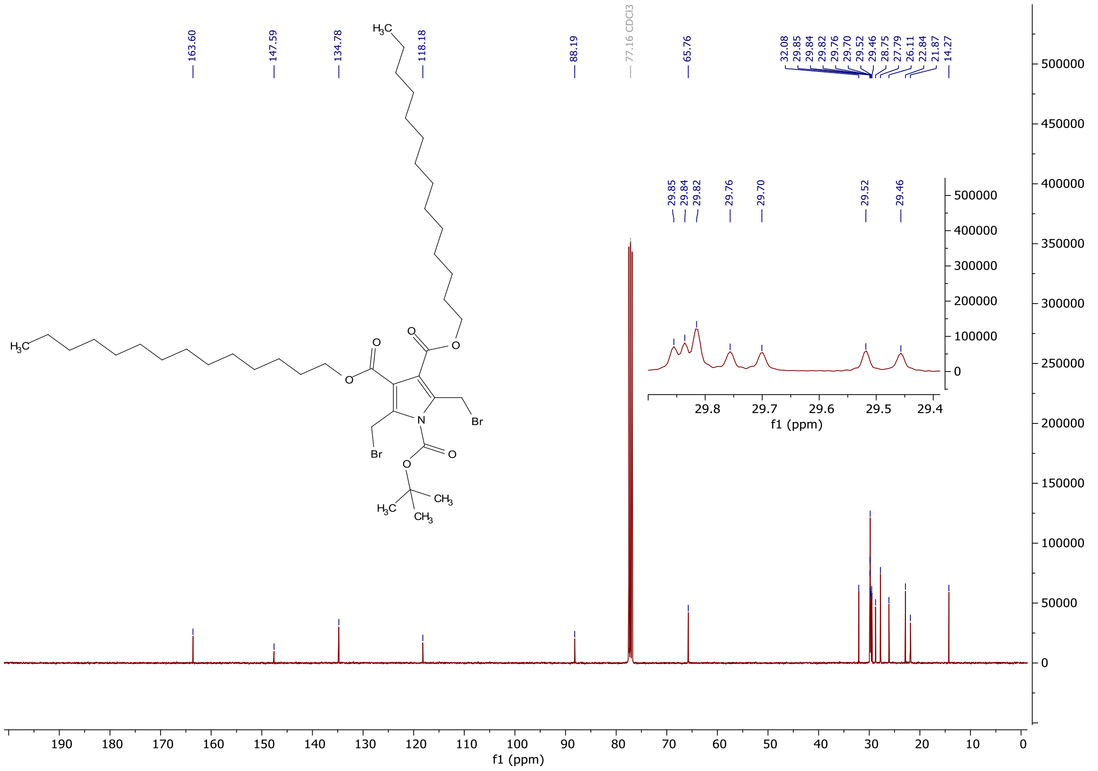

Figure S45: ^{13}C-NMR spectrum of 1-(*tert*-butyl) 3,4-ditetradecyl 2,5-bis(bromomethyl)-1*H*-pyrrole-1,3,4-tricarboxylate (**23’b**)

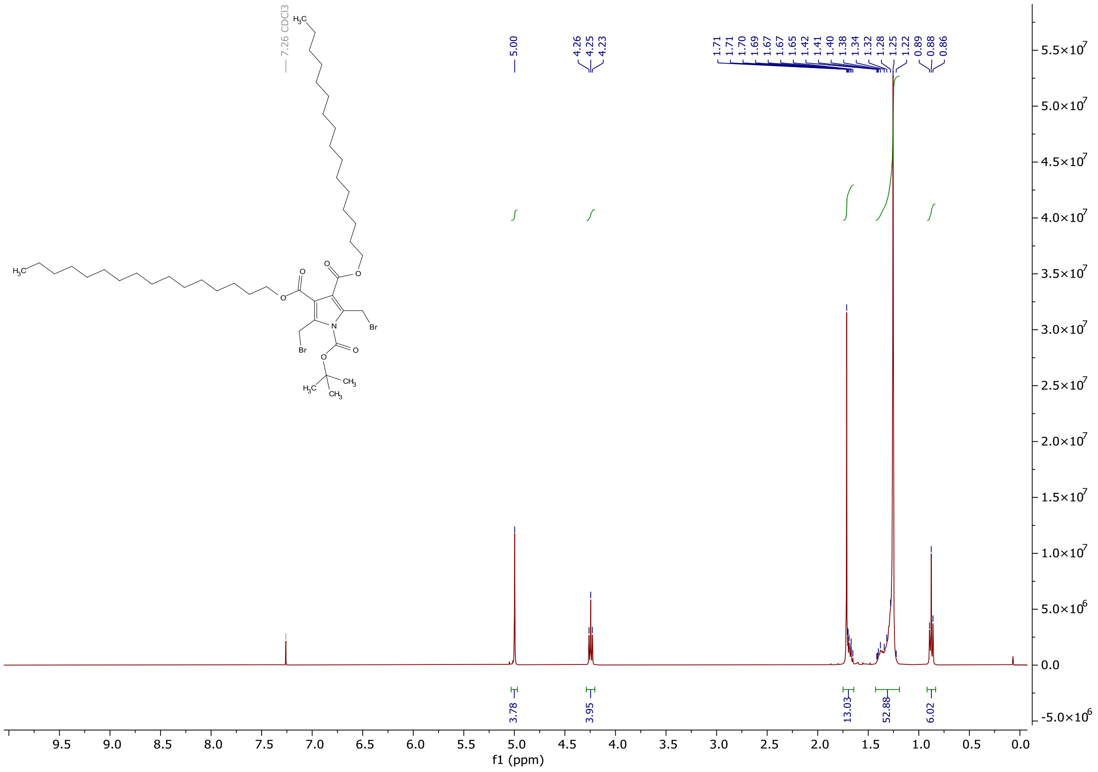

Figure S46: ^{1}H-NMR spectrum of 1-(*tert*-butyl) 3,4-dihexadecyl 2,5-bis(bromomethyl)-1*H*-pyrrole-1,3,4-tricarboxylate (**23’c**)

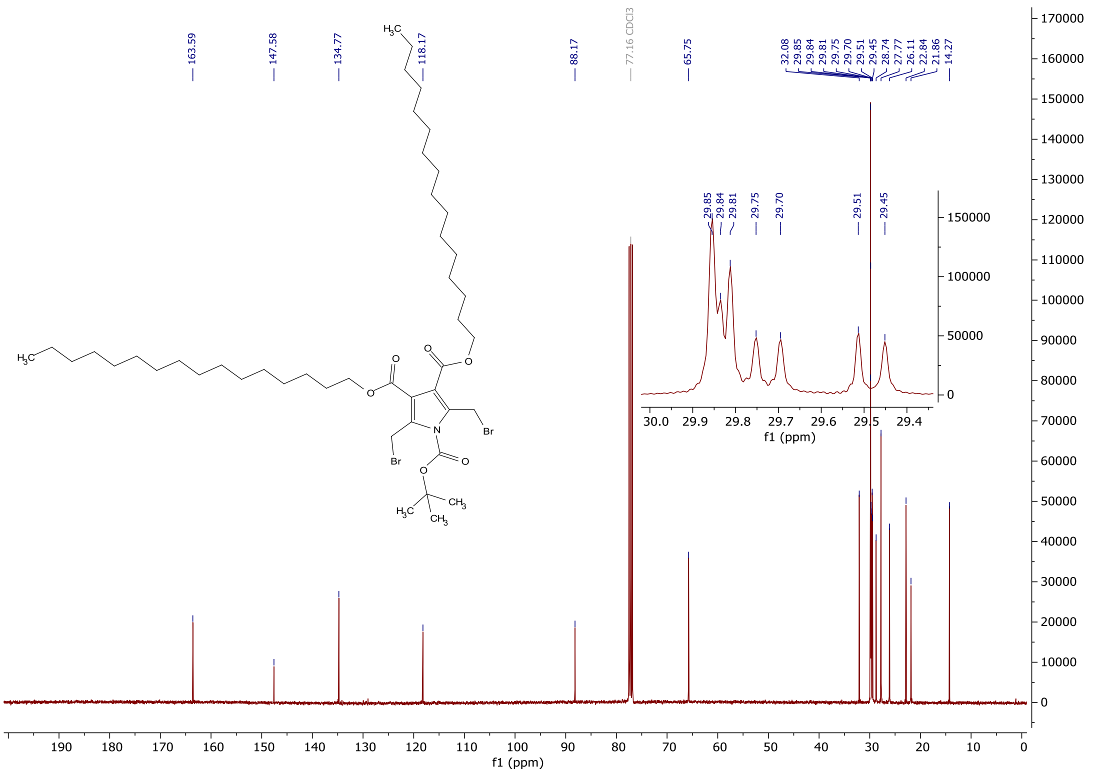

Figure S47: ^{13}C-NMR spectrum of 1-(*tert*-butyl) 3,4-dihexadecyl 2,5-bis(bromomethyl)-1*H*-pyrrole-1,3,4-tricarboxylate (**23'c**)

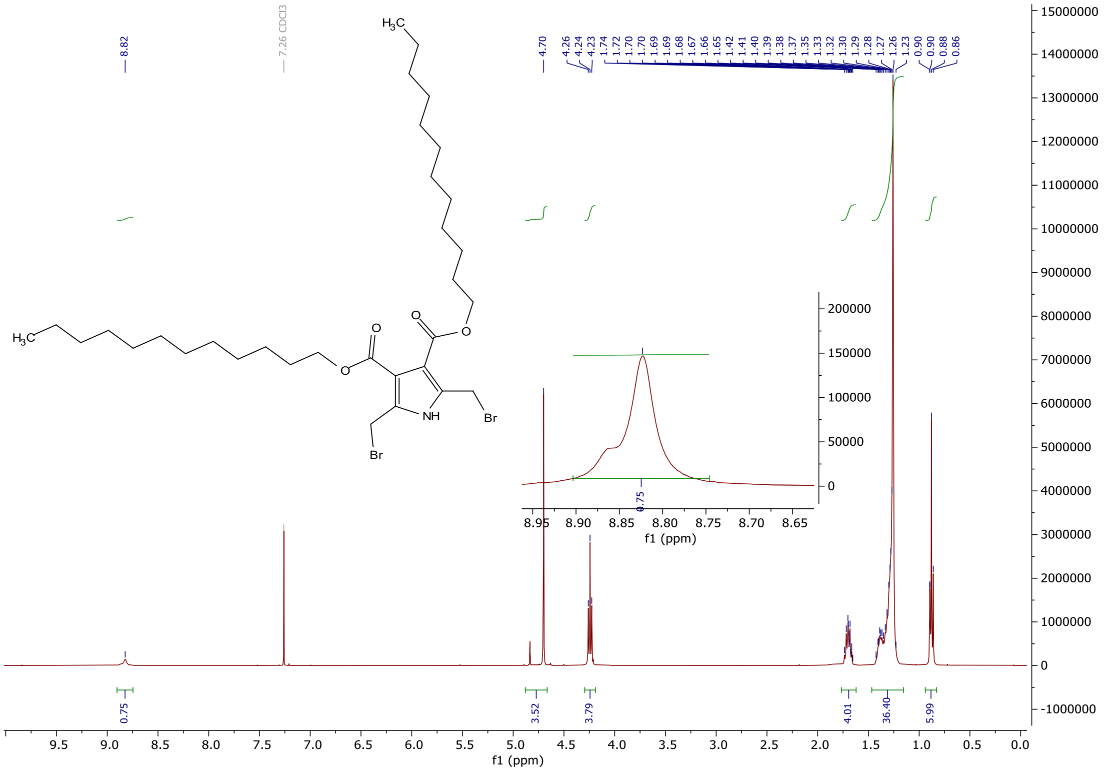

Figure S48: ^{1}H-NMR spectrum of didodecyl 2,5-bis(bromomethyl)-1*H*-pyrrole-3,4-dicarboxylate (**23a**)

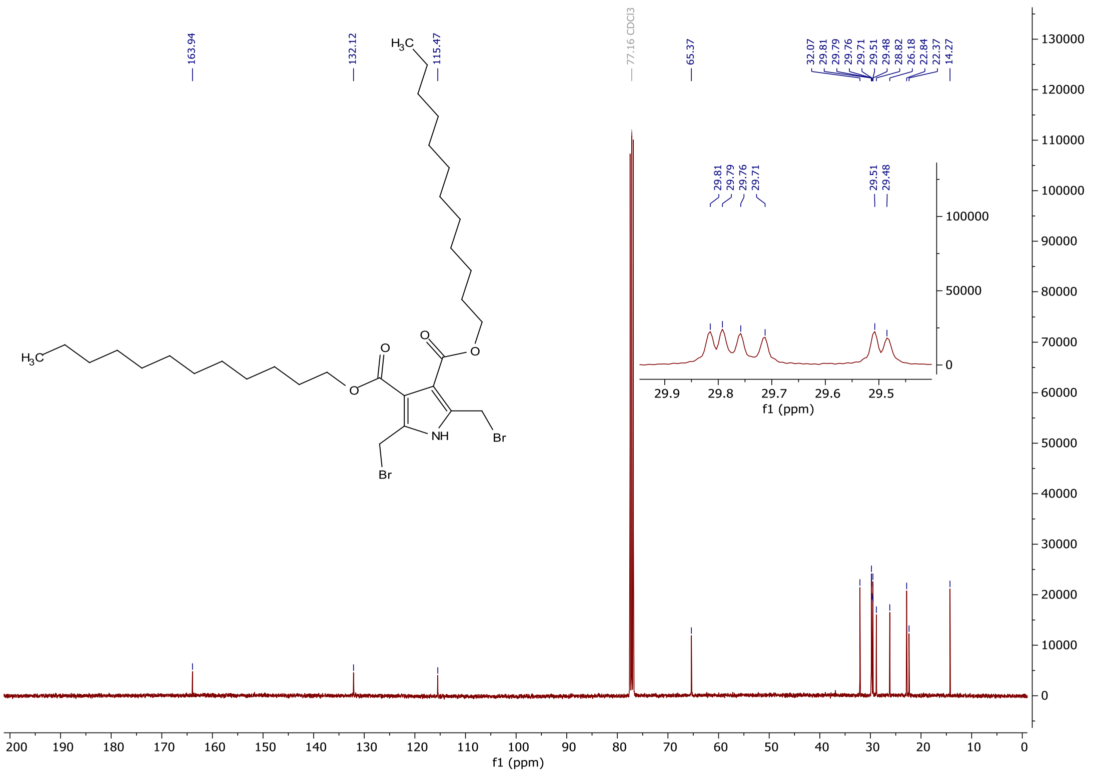

Figure S49: ^{13}C-NMR spectrum of didodecyl 2,5-bis(bromomethyl)-1*H*-pyrrole-3,4-dicarboxylate (**23a**)

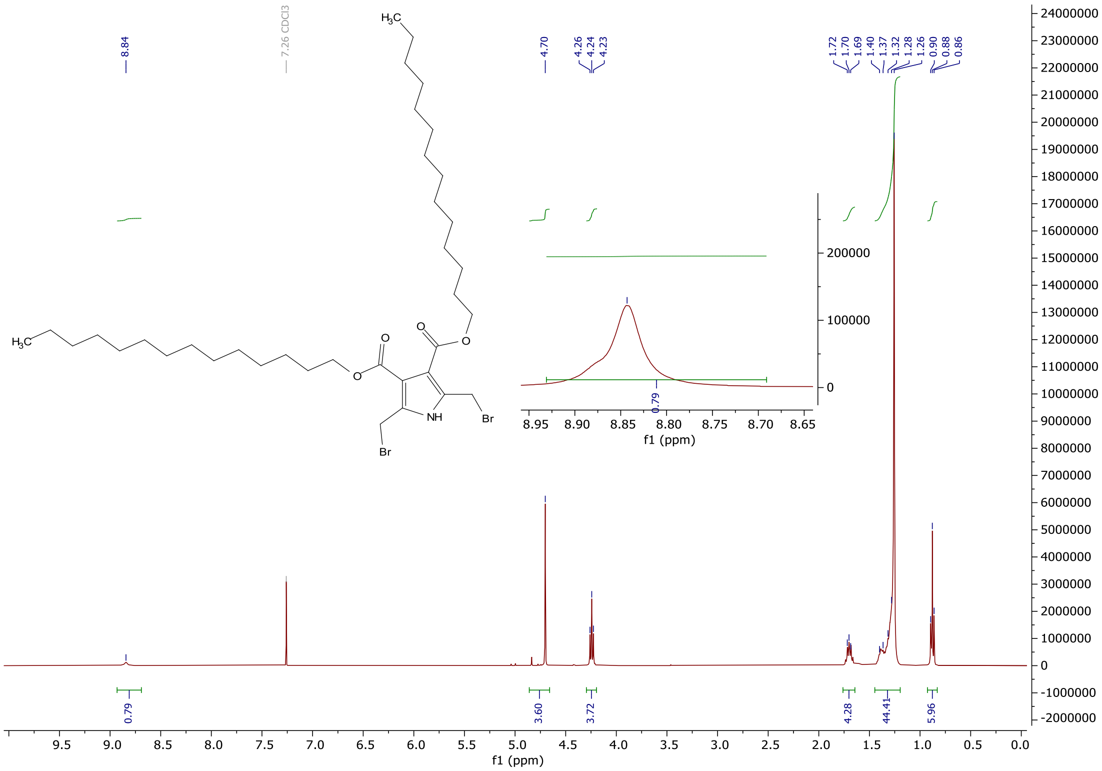

Figure S50: ^{1}H-NMR spectrum of ditetradecyl 2,5-bis(bromomethyl)-1*H*-pyrrole-3,4-dicarboxylate (**23b**)

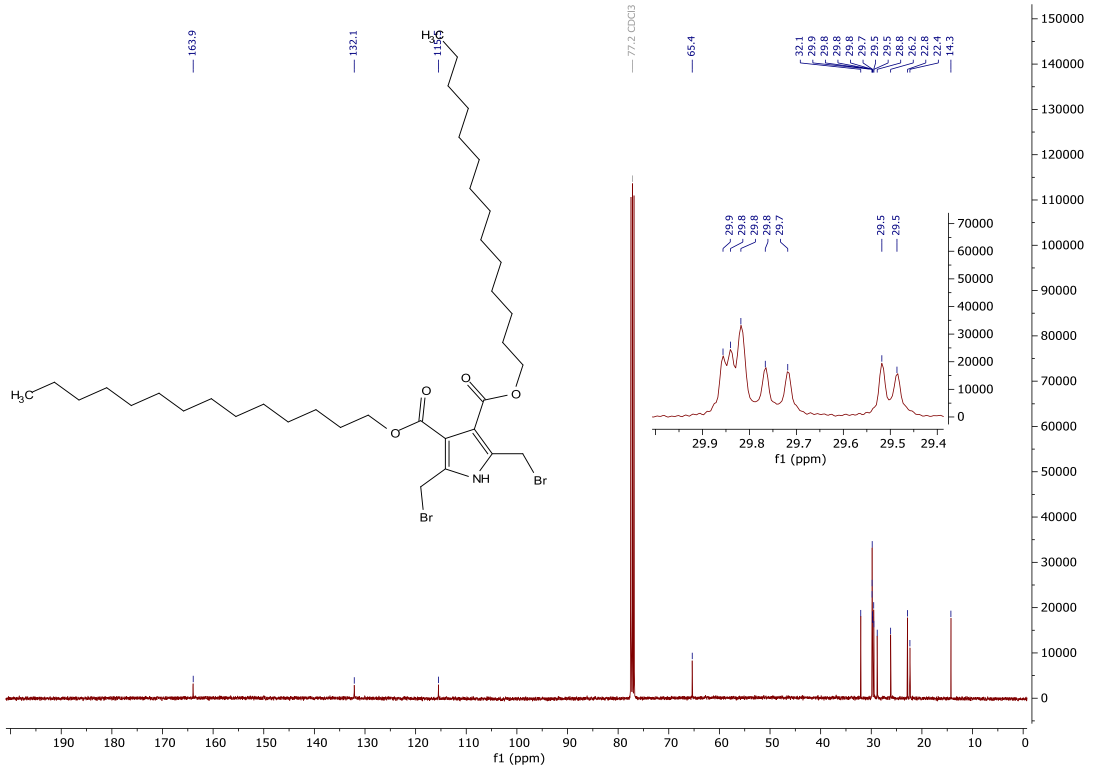

Figure S51: ^{13}C-NMR spectrum of ditetradecyl 2,5-bis(bromomethyl)-1*H*-pyrrole-3,4-dicarboxylate (**23b**)

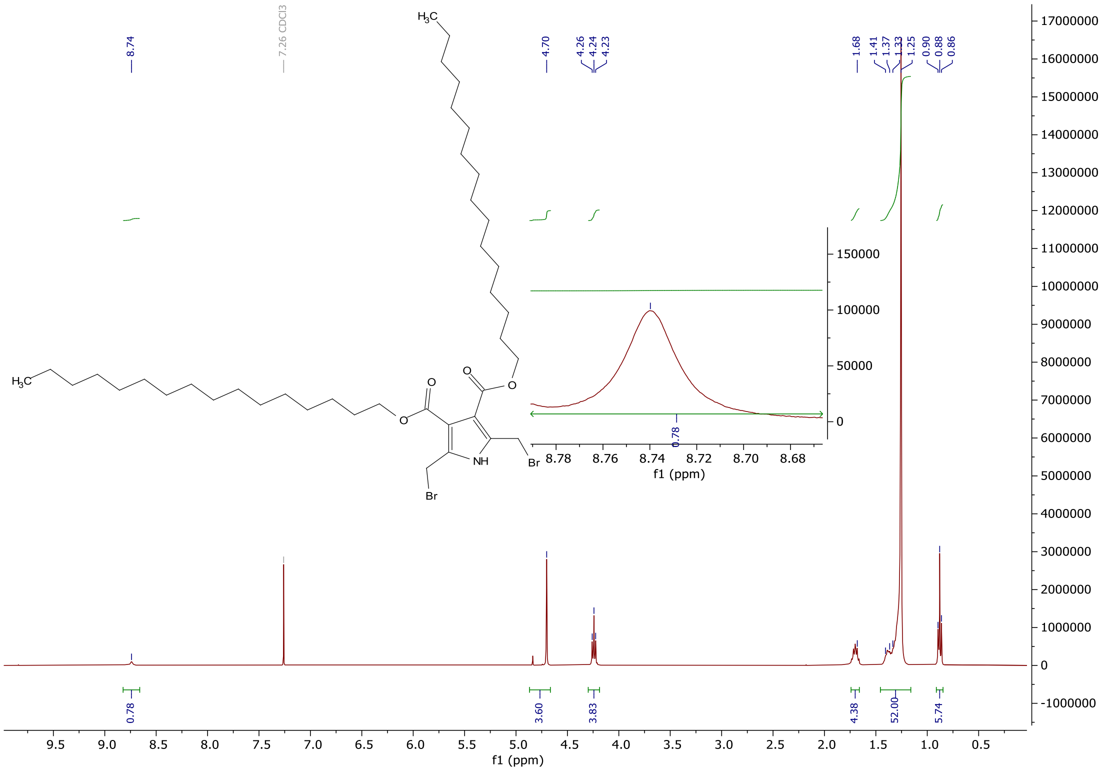

Figure S52: ^{1}H-NMR spectrum of dihexadecyl 2,5-bis(bromomethyl)-1*H*-pyrrole-3,4-dicarboxylate (**23c**)

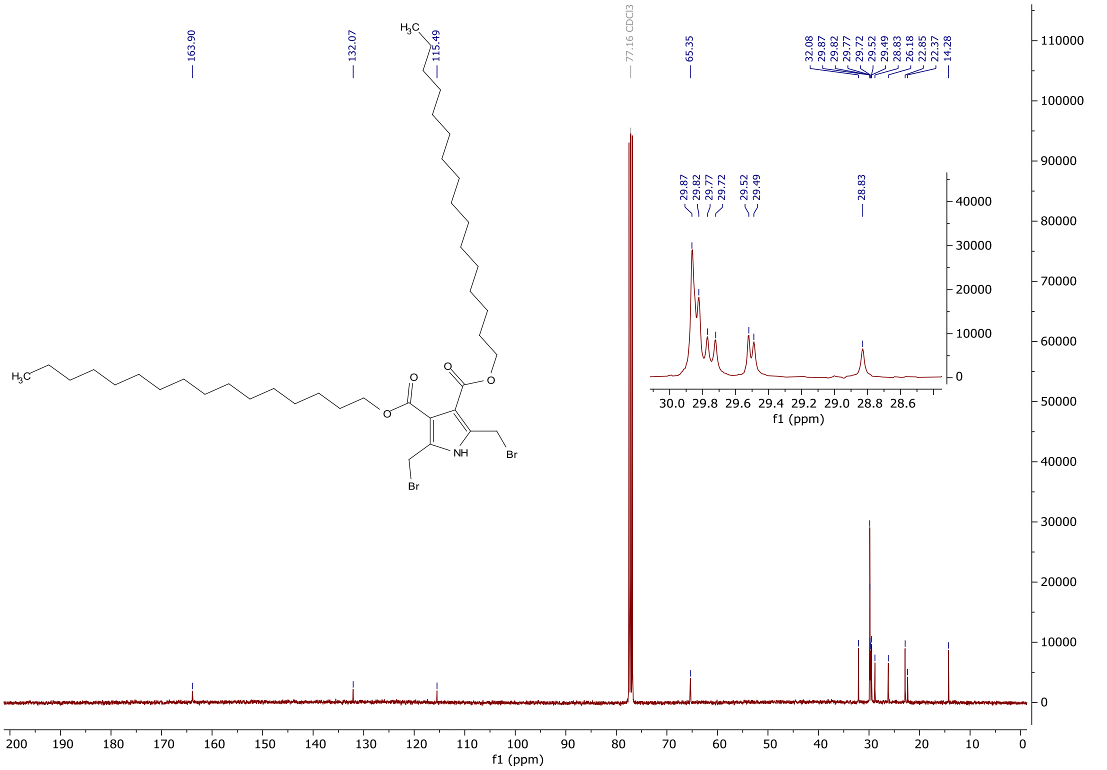

Figure S53: ^{13}C-NMR spectrum of dihexadecyl 2,5-bis(bromomethyl)-1*H*-pyrrole-3,4-dicarboxylate (**23c**)

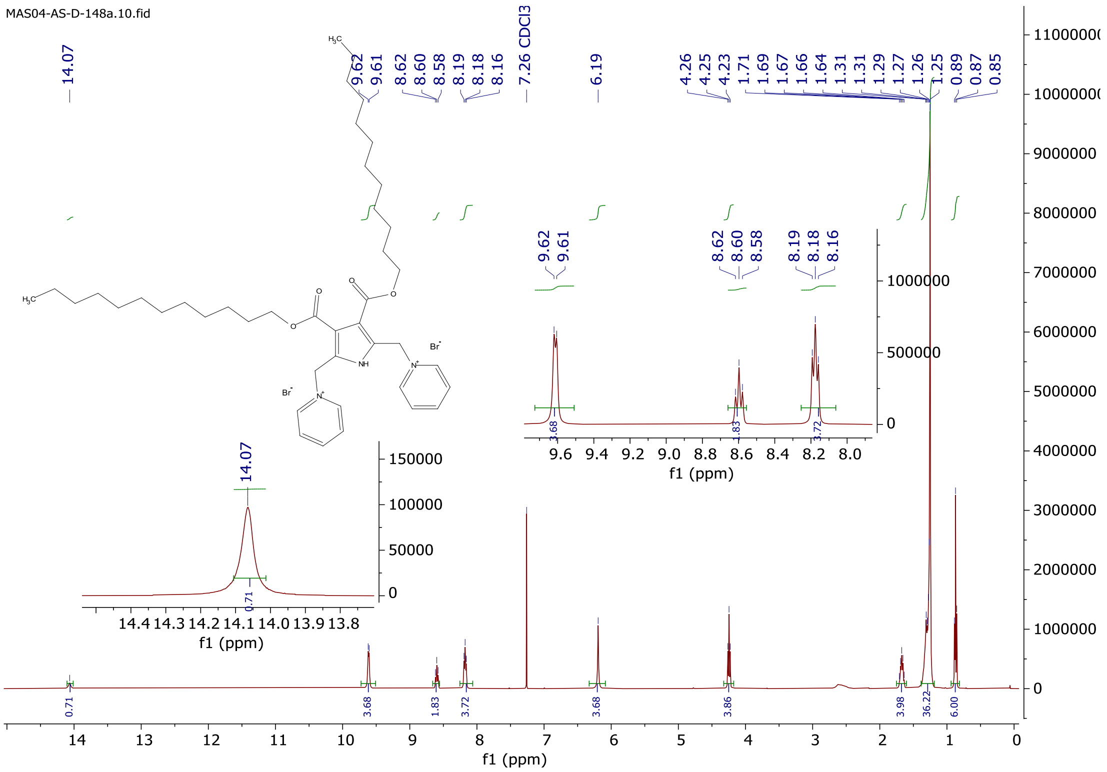

Figure S54: ^{1}H-NMR spectrum of 1,1'-((3,4-bis((dodecyloxy)carbonyl)-1H-pyrrole-2,5-diyl)bis(methylene))bis(pyridin-1-ium) dibromide (**5a**)

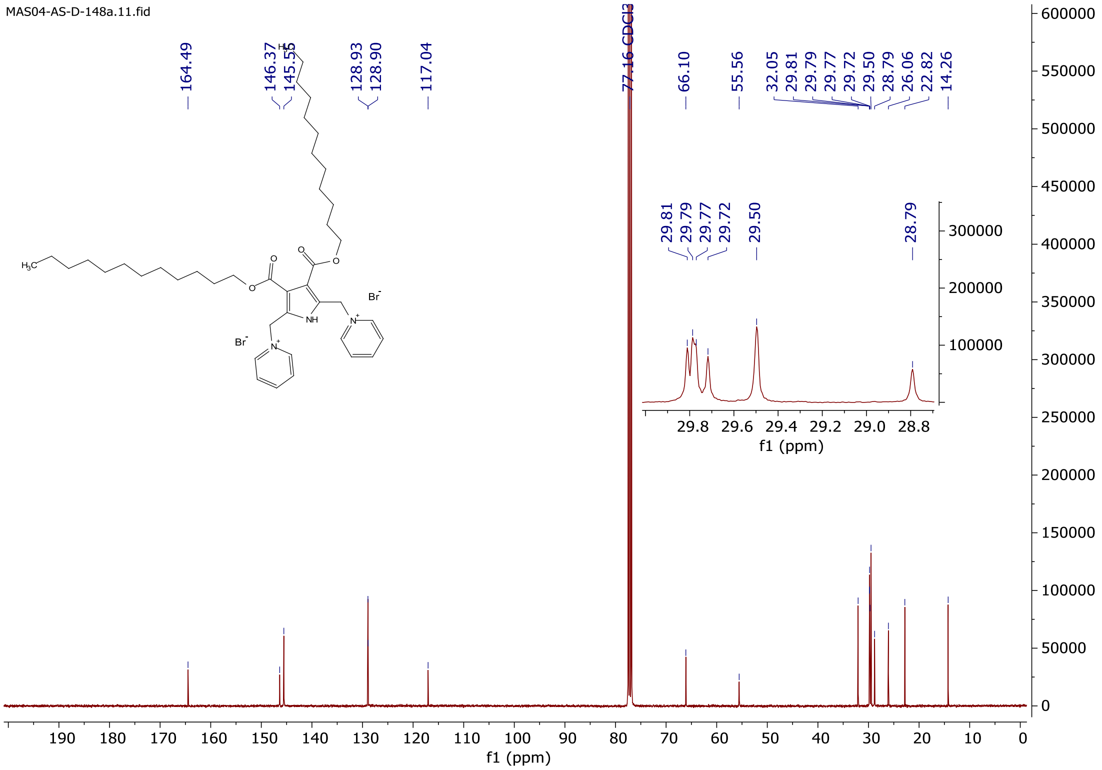

Figure S55: ^{13}C-NMR spectrum of 1,1'-((3,4-bis((dodecyloxy)carbonyl)-1H-pyrrole-2,5-diyl)bis(methylene))bis(pyridin-1-ium) dibromide (**5a**)

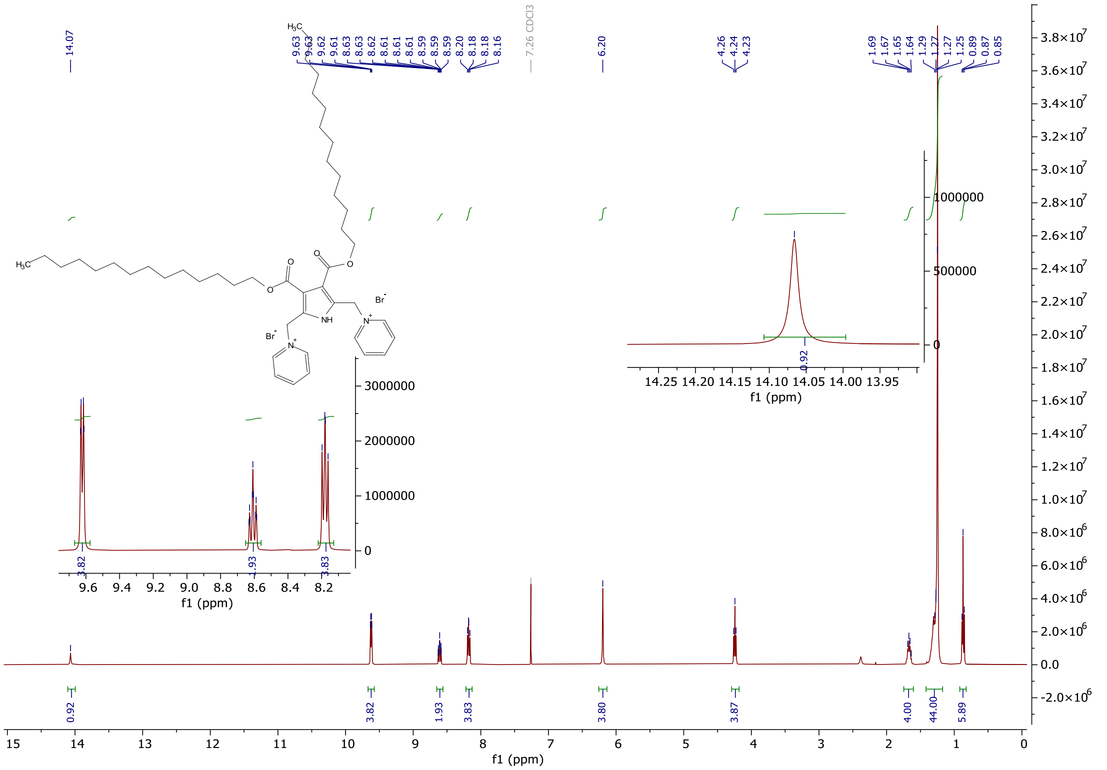

Figure S56: ^{1}H-NMR spectrum of 1,1'-((3,4-bis((tetradecyloxy)carbonyl)-1*H*-pyrrole-2,5-diyl)bis(methylene))bis(pyridin-1-ium) dibromide (**5b**)

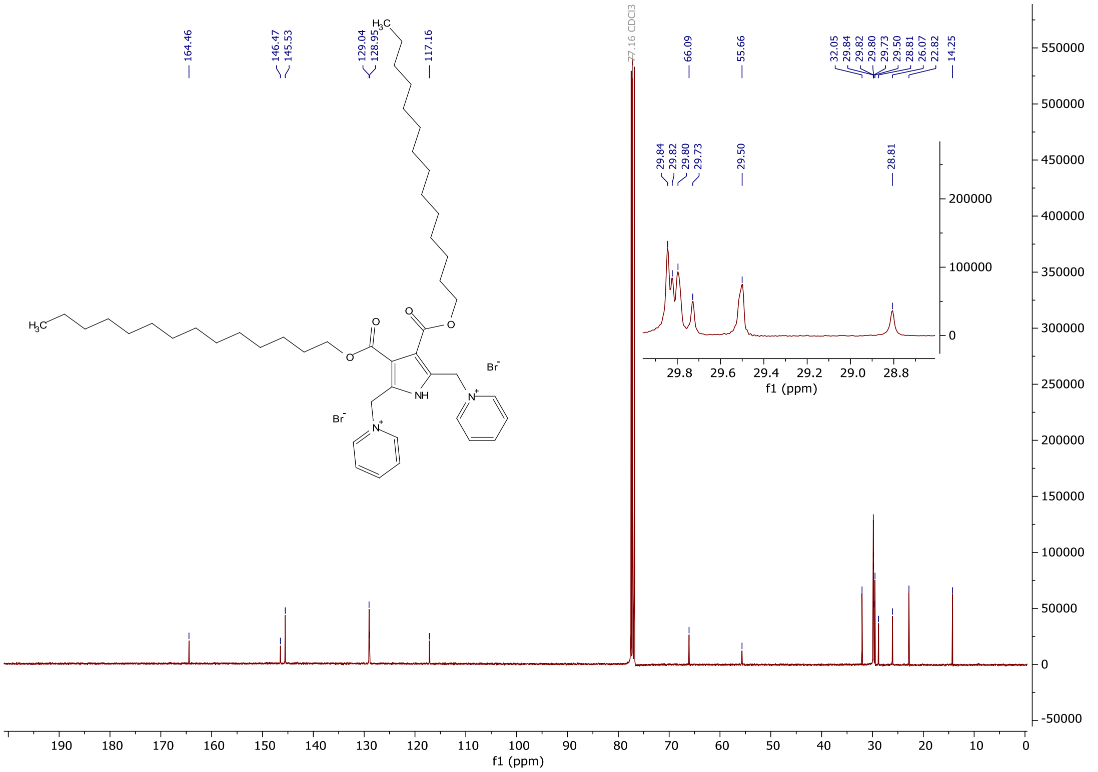

Figure S57: ^{13}C-NMR spectrum of 1,1'-((3,4-bis((tetradecyloxy)carbonyl)-1*H*-pyrrole-2,5-diyl)bis(methylene))bis(pyridin-1-ium) dibromide (**5b**)

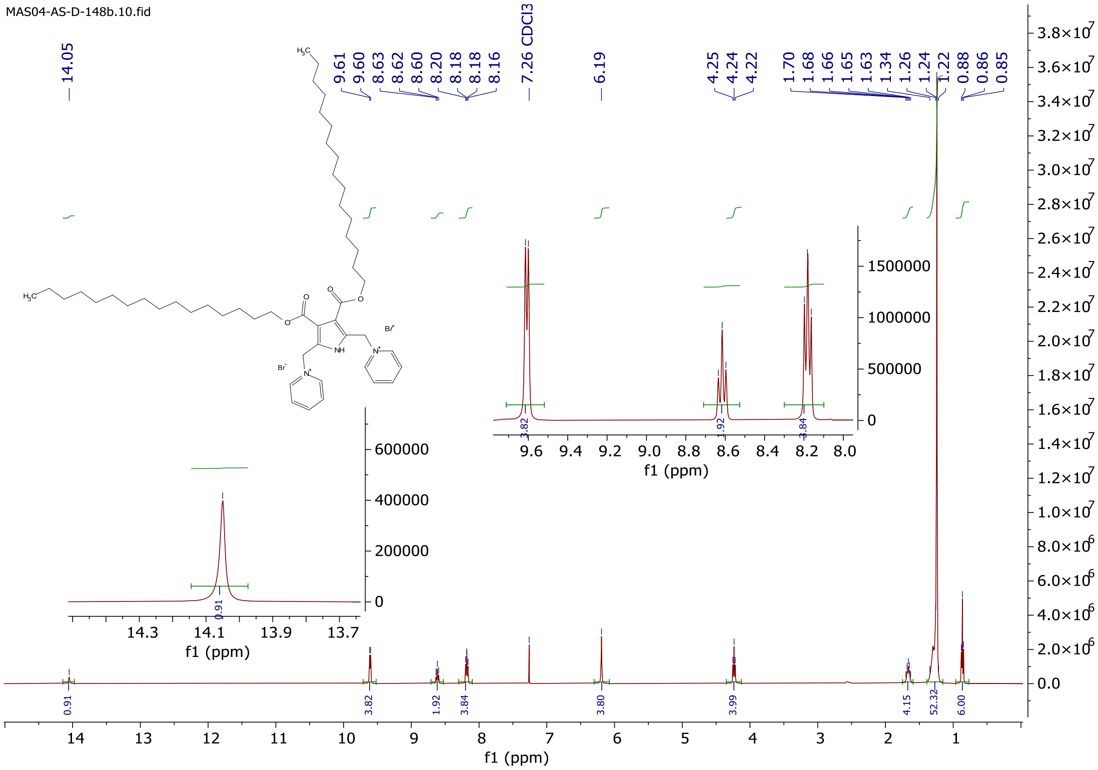

Figure S58: ^{1}H-NMR spectrum of 1,1'-((3,4-bis((hexadecyloxy)carbonyl)-1*H*-pyrrole-2,5-diyl)bis(methylene))bis(pyridin-1-ium) dibromide (**5c**)

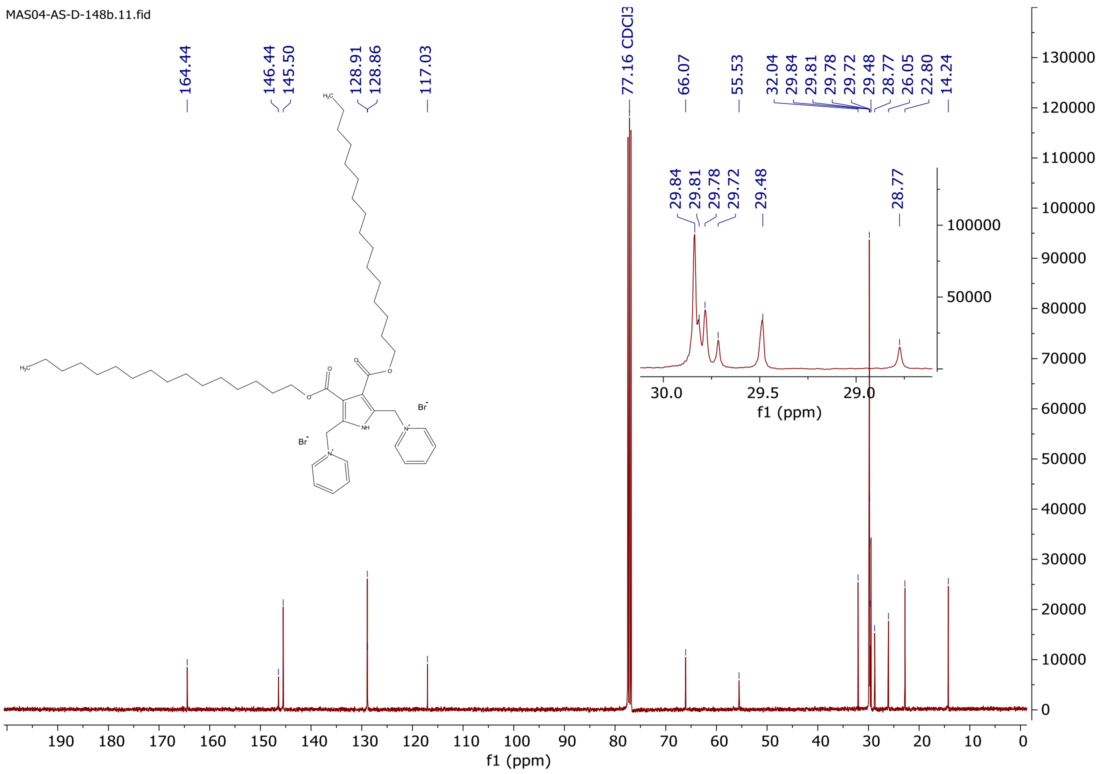

Figure S59: ^{13}C-NMR spectrum of 1,1'-((3,4-bis((hexadecyloxy)carbonyl)-1*H*-pyrrole-2,5-diyl)bis(methylene))bis(pyridin-1-ium) dibromide (**5c**)

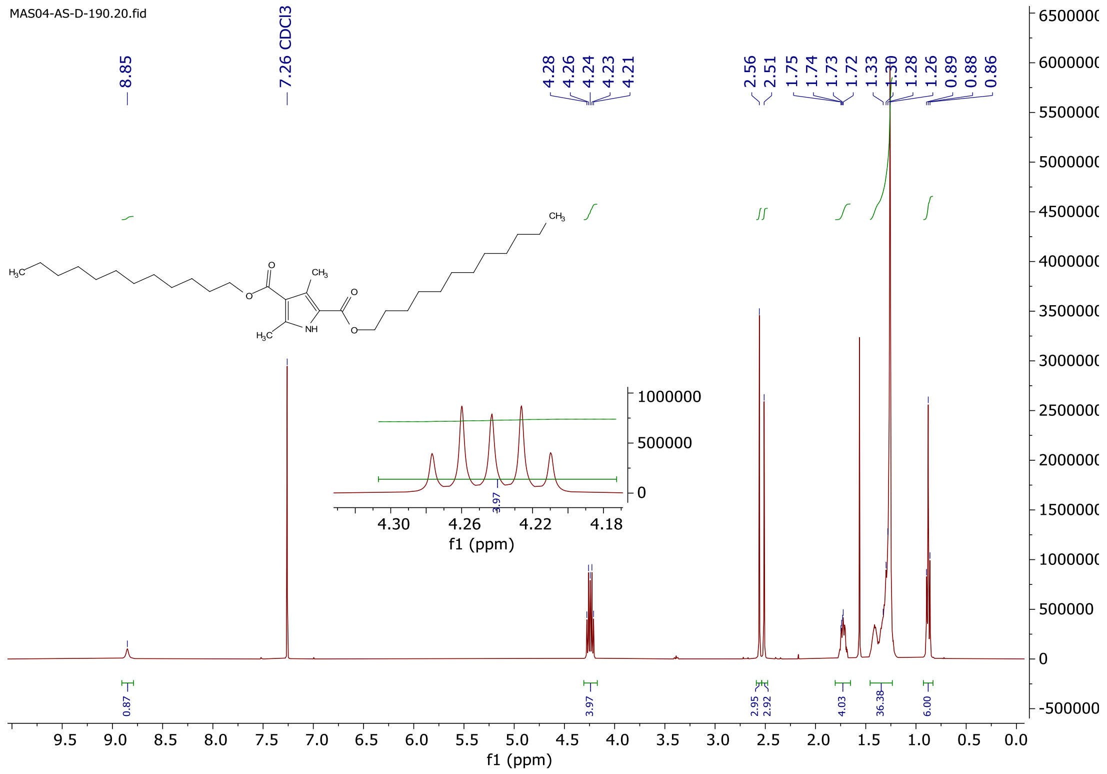

Figure S60: ^{1}H-NMR spectrum of didodecyl 3,5-dimethyl-1*H*-pyrrole-2,4-dicarboxylate (**26a**)

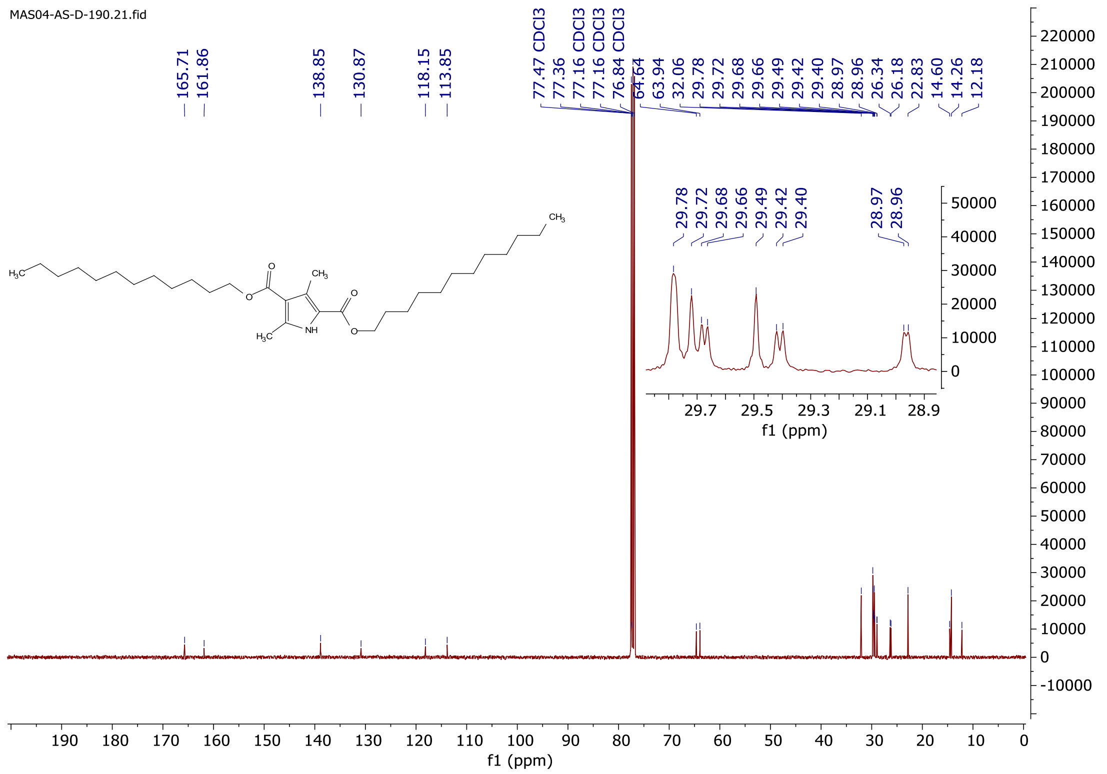

Figure S61: ^{13}C-NMR spectrum of didodecyl 3,5-dimethyl-1*H*-pyrrole-2,4-dicarboxylate (**26a**)

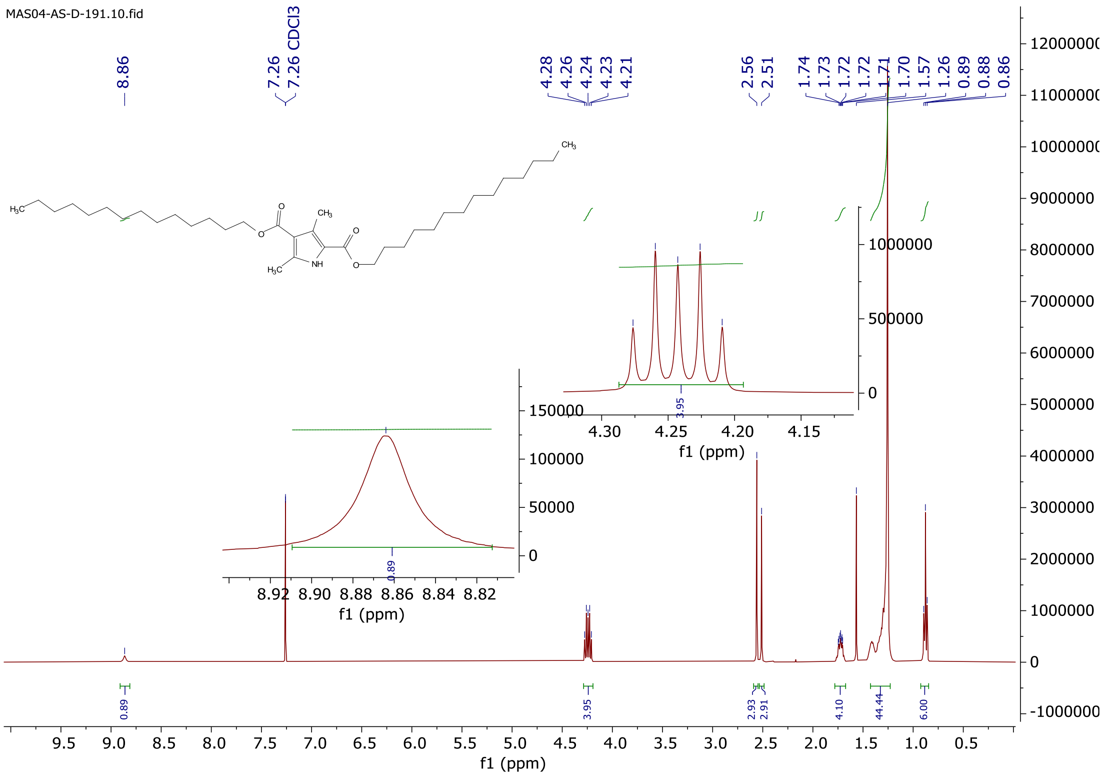

Figure S62: ^{1}H-NMR spectrum of ditetradecyl 3,5-dimethyl-1*H*-pyrrole-2,4-dicarboxylate (**26b**)

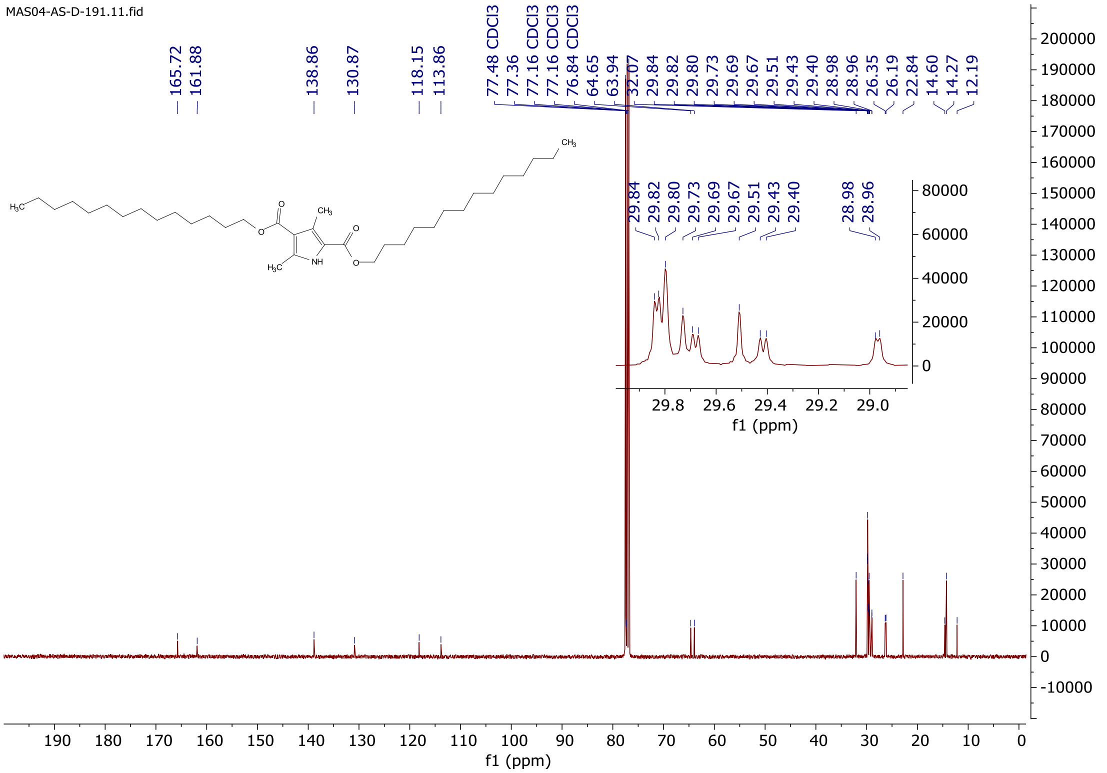

Figure S63: ^{13}C-NMR spectrum of ditetradecyl 3,5-dimethyl-1*H*-pyrrole-2,4-dicarboxylate (**26b**)

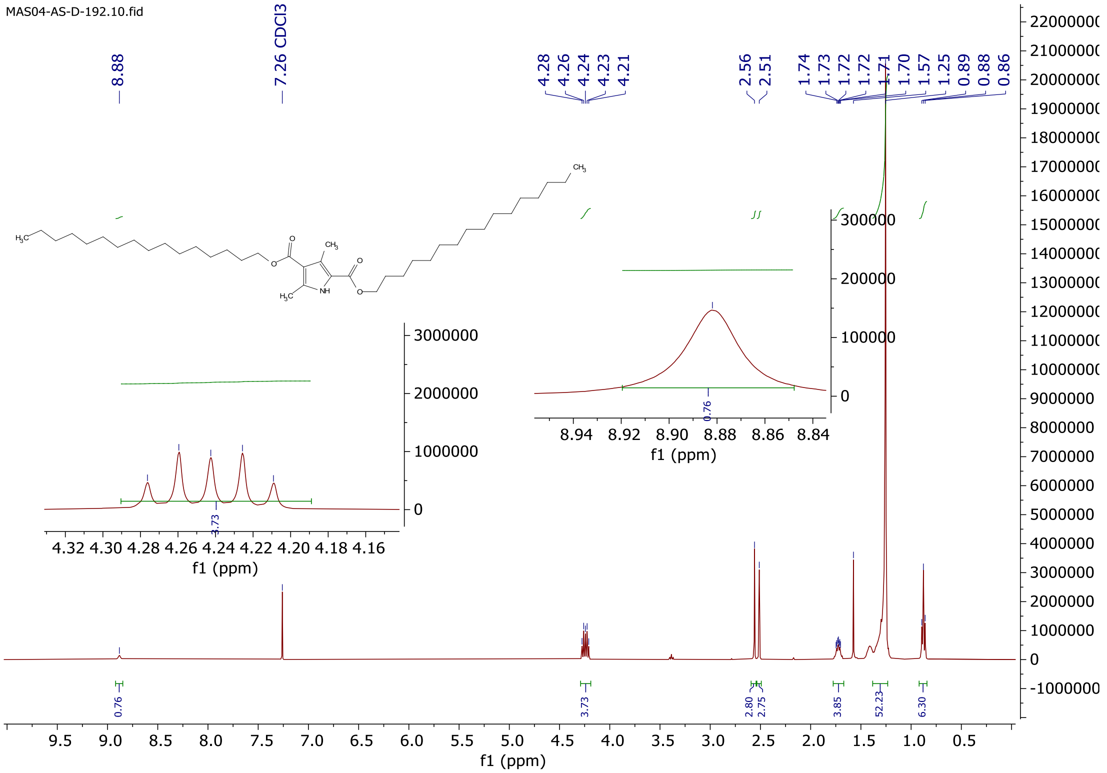

Figure S64: ^{1}H-NMR spectrum of dihexadecyl 3,5-dimethyl-1*H*-pyrrole-2,4-dicarboxylate (**26c**)

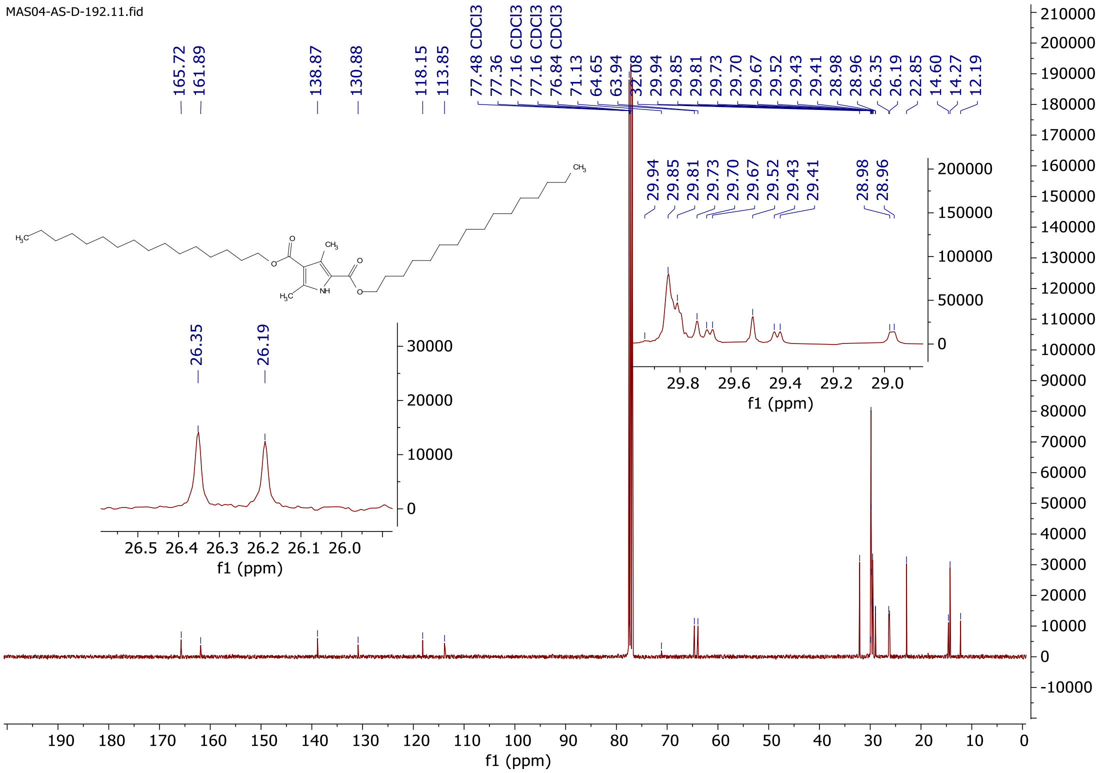

Figure S65: ^{13}C-NMR spectrum of dihexadecyl 3,5-dimethyl-1*H*-pyrrole-2,4-dicarboxylate (**26c**)

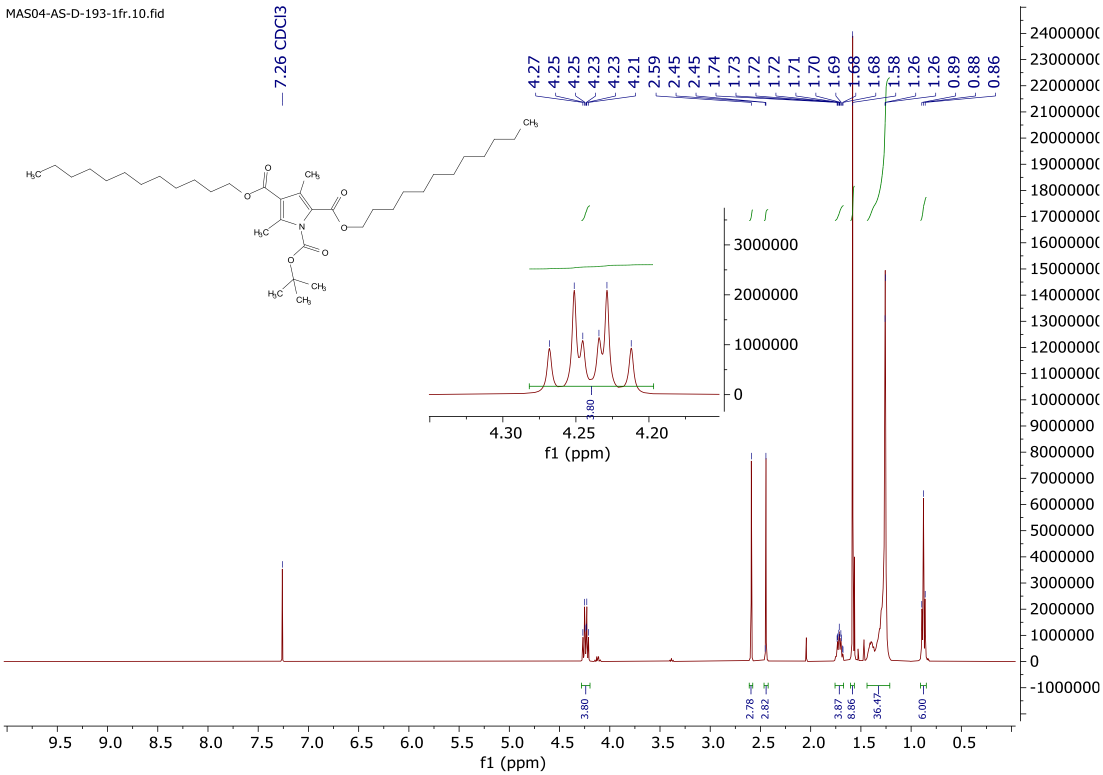

Figure S66: ^{1}H-NMR spectrum of 1-(*tert*-butyl) 2,4-didodecyl 3,5-dimethyl-1*H*-pyrrole-1,2,4-tricarboxylate (**27a**)

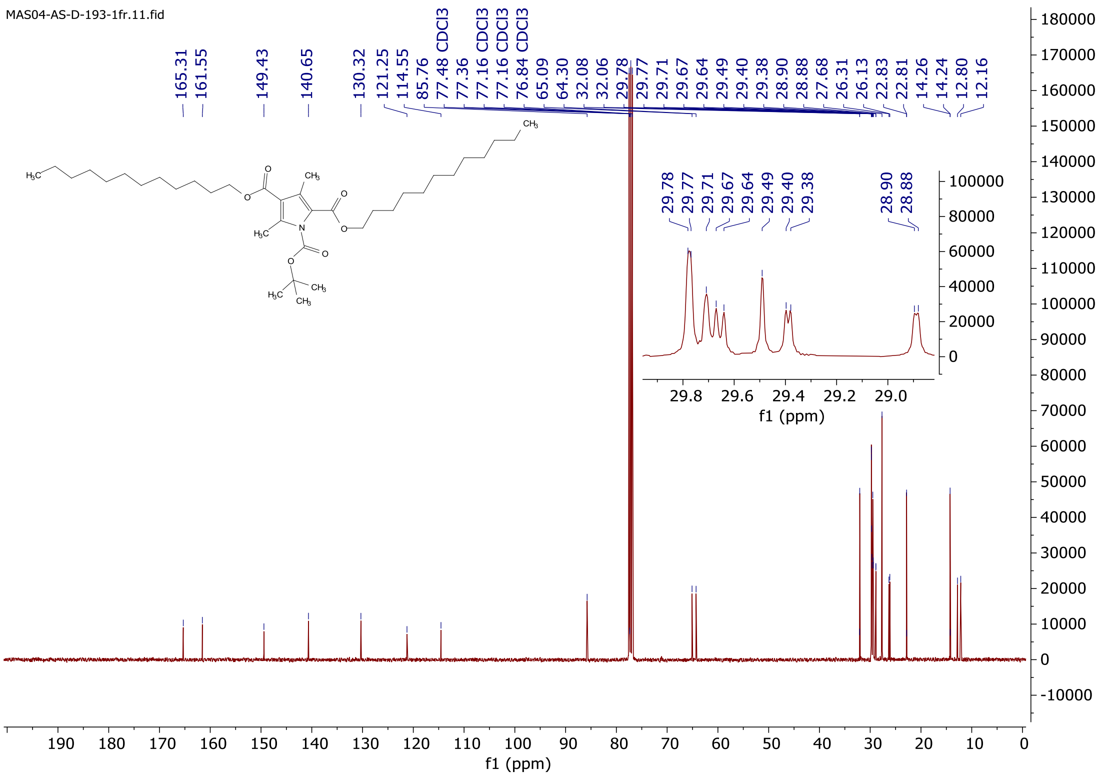

Figure S67: ^{13}C-NMR spectrum of 1-(*tert*-butyl) 2,4-didodecyl 3,5-dimethyl-1*H*-pyrrole-1,2,4-tricarboxylate (**27a**)

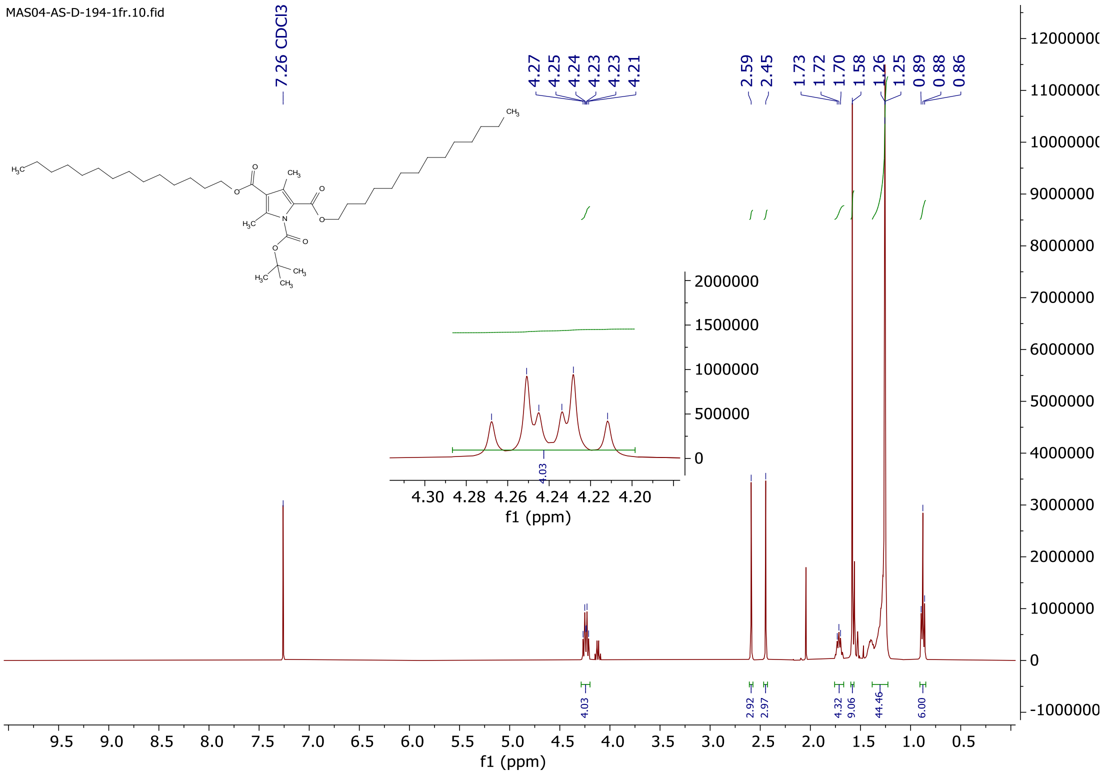

Figure S68: ^{1}H-NMR spectrum of 1-(*tert*-butyl) 2,4-ditetradecyl 3,5-dimethyl-1*H*-pyrrole-1,2,4-tricarboxylate (**27b**)

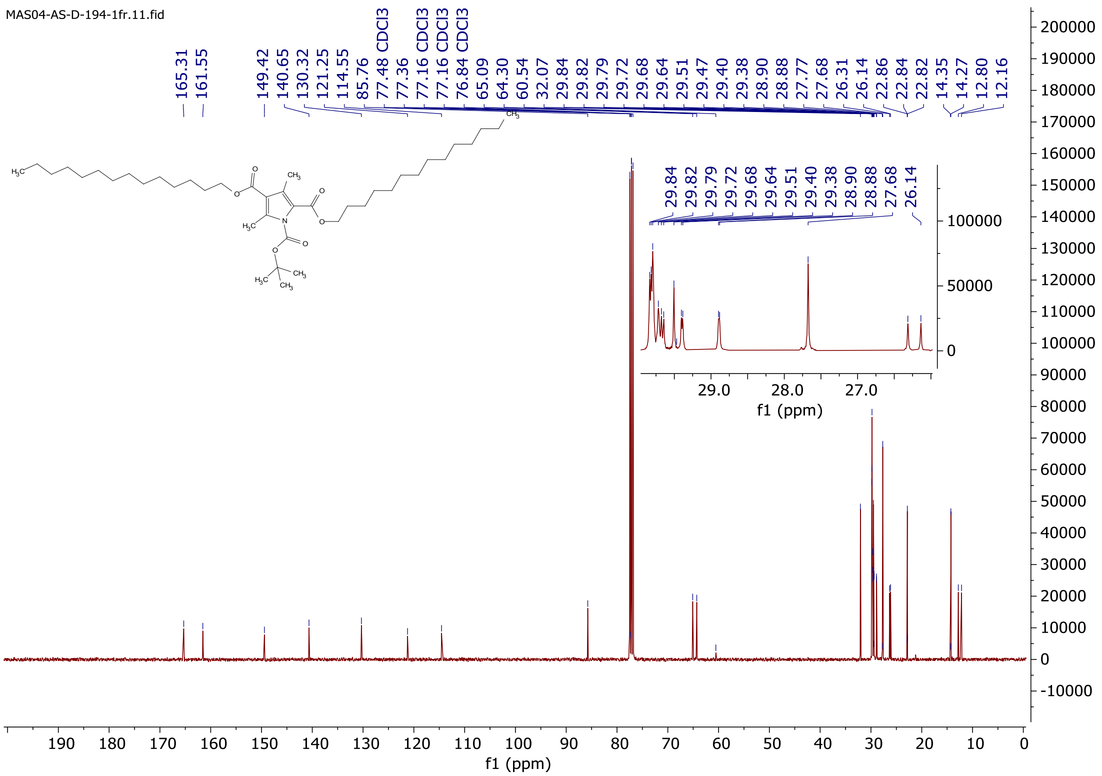

Figure S69: ^{13}C-NMR spectrum of 1-(*tert*-butyl) 2,4-ditetradecyl 3,5-dimethyl-1*H*-pyrrole-1,2,4-tricarboxylate (**27b**)

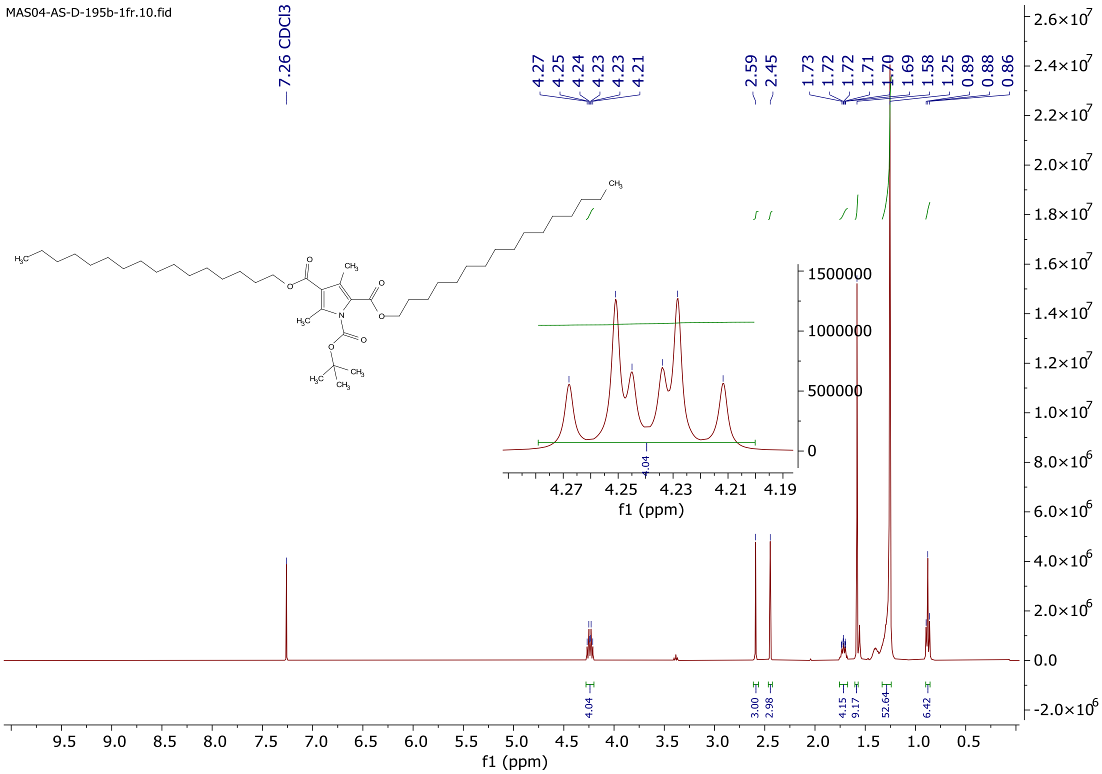

Figure S70: ^{1}H-NMR spectrum of 1-(*tert*-butyl) 2,4-dihexadecyl 3,5-dimethyl-1*H*-pyrrole-1,2,4-tricarboxylate (**27c**)

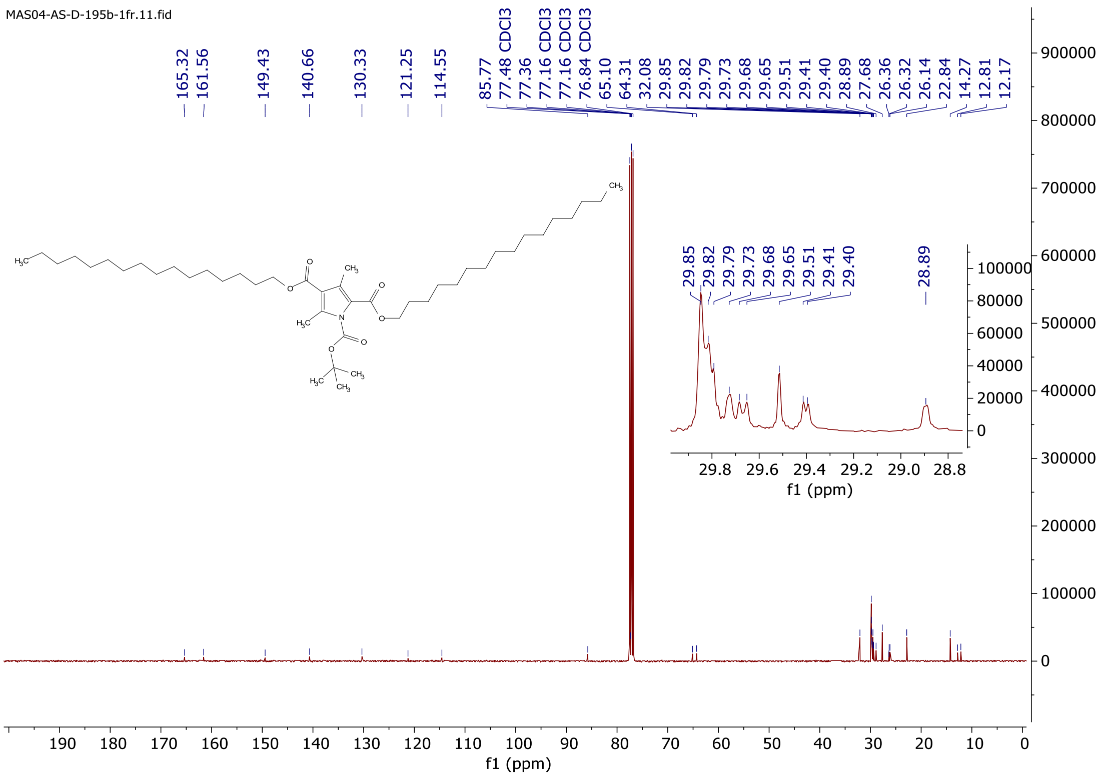

Figure S71: ^{13}C-NMR spectrum of 1-(*tert*-butyl) 2,4-dihexadecyl 3,5-dimethyl-1*H*-pyrrole-1,2,4-tricarboxylate (**27c**)

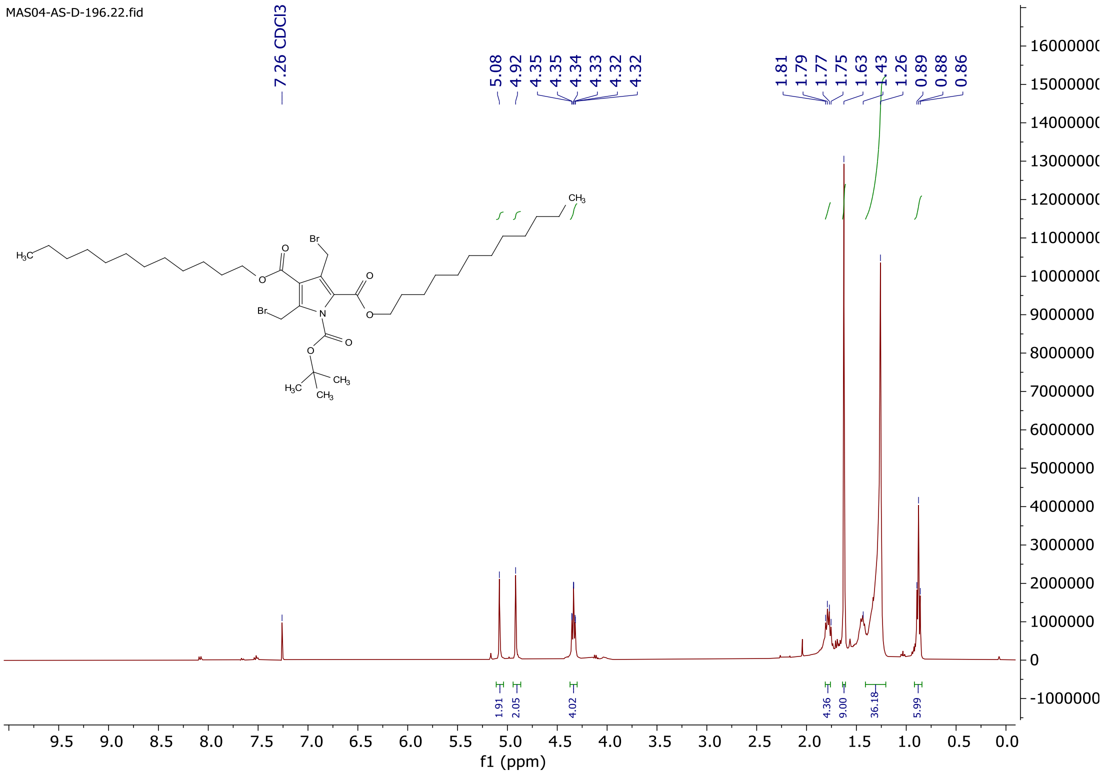

Figure S72: ^{1}H-NMR spectrum of 1-(*tert*-butyl) 2,4-didodecyl 3,5-bis(bromomethyl)-1*H*-pyrrole-1,2,4-tricarboxylate (**28a**)

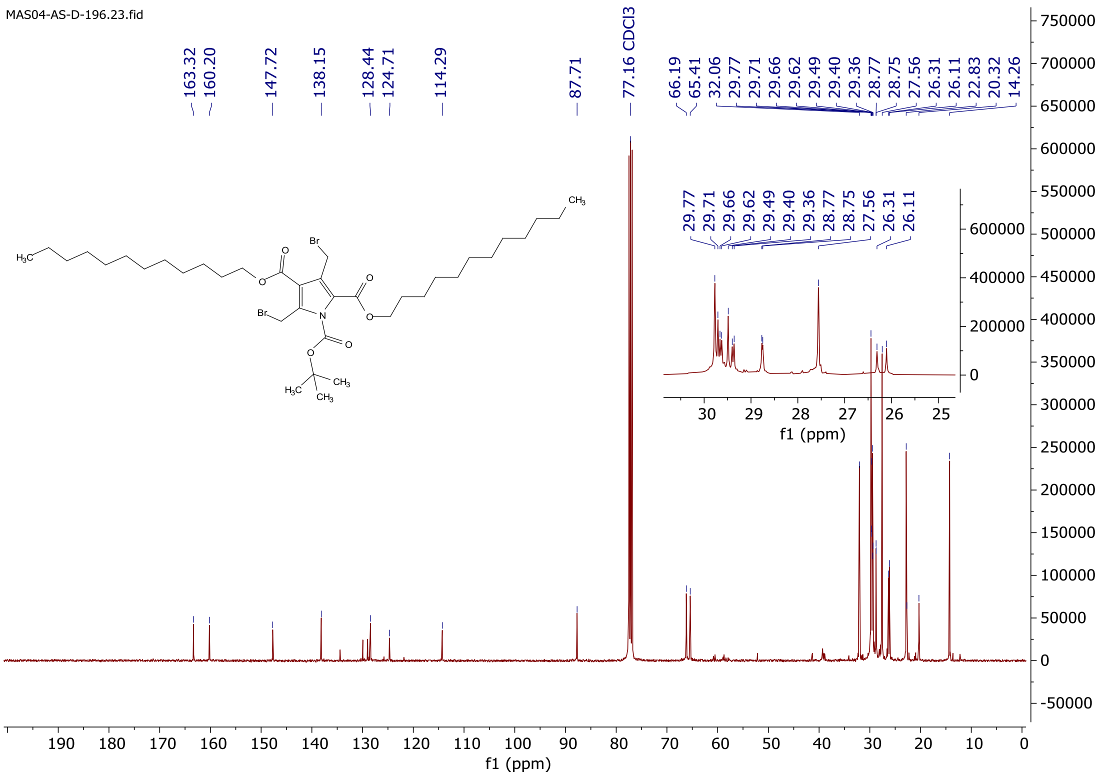

Figure S73: ^{13}C-NMR spectrum of 1-(*tert*-butyl) 2,4-didodecyl 3,5-bis(bromomethyl)-1*H*-pyrrole-1,2,4-tricarboxylate (**28a**)

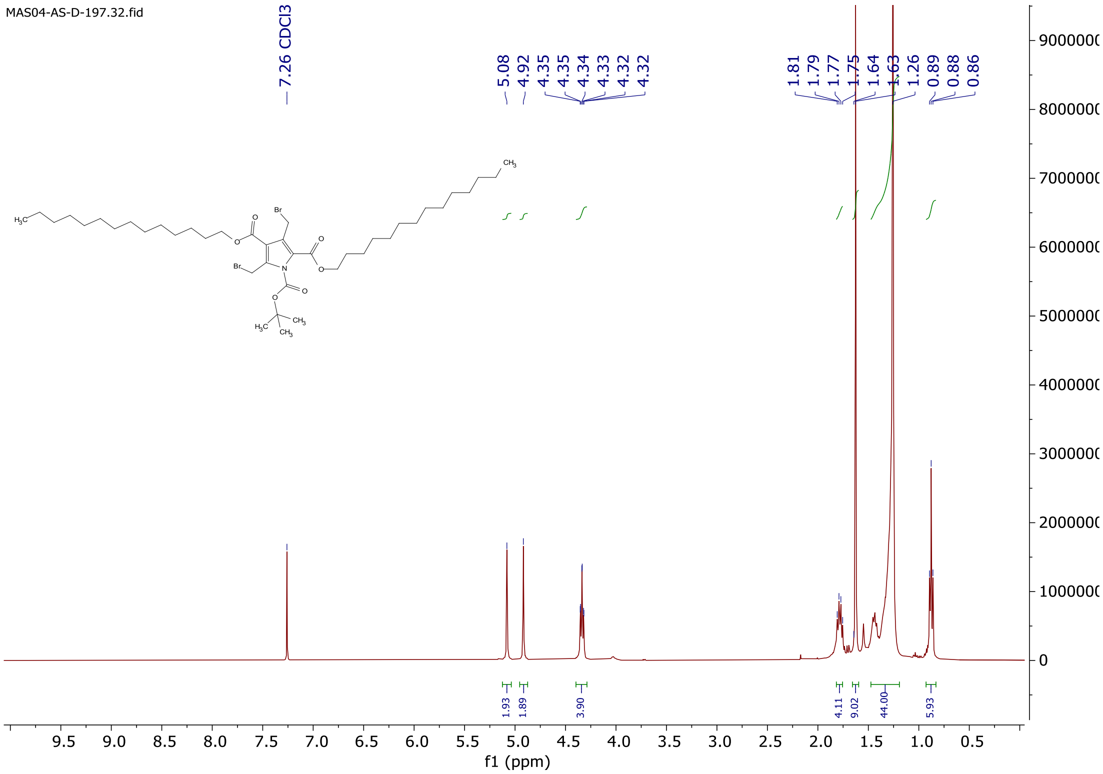

Figure S74: ^{1}H-NMR spectrum of 1-(*tert*-butyl) 2,4-ditetradecyl 3,5-bis(bromomethyl)-1*H*-pyrrole-1,2,4-tricarboxylate (**28b**)

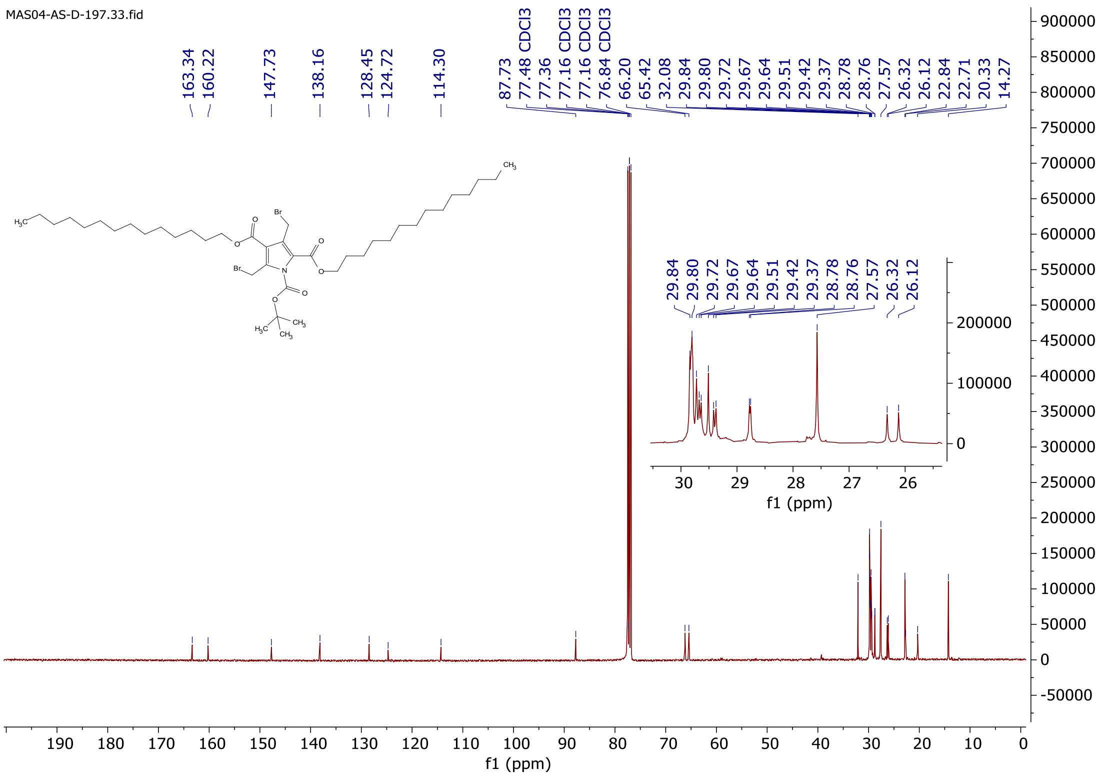

Figure S75: ^{13}C-NMR spectrum of 1-(*tert*-butyl) 2,4-ditetradecyl 3,5-bis(bromomethyl)-1*H*-pyrrole-1,2,4-tricarboxylate (**28b**)

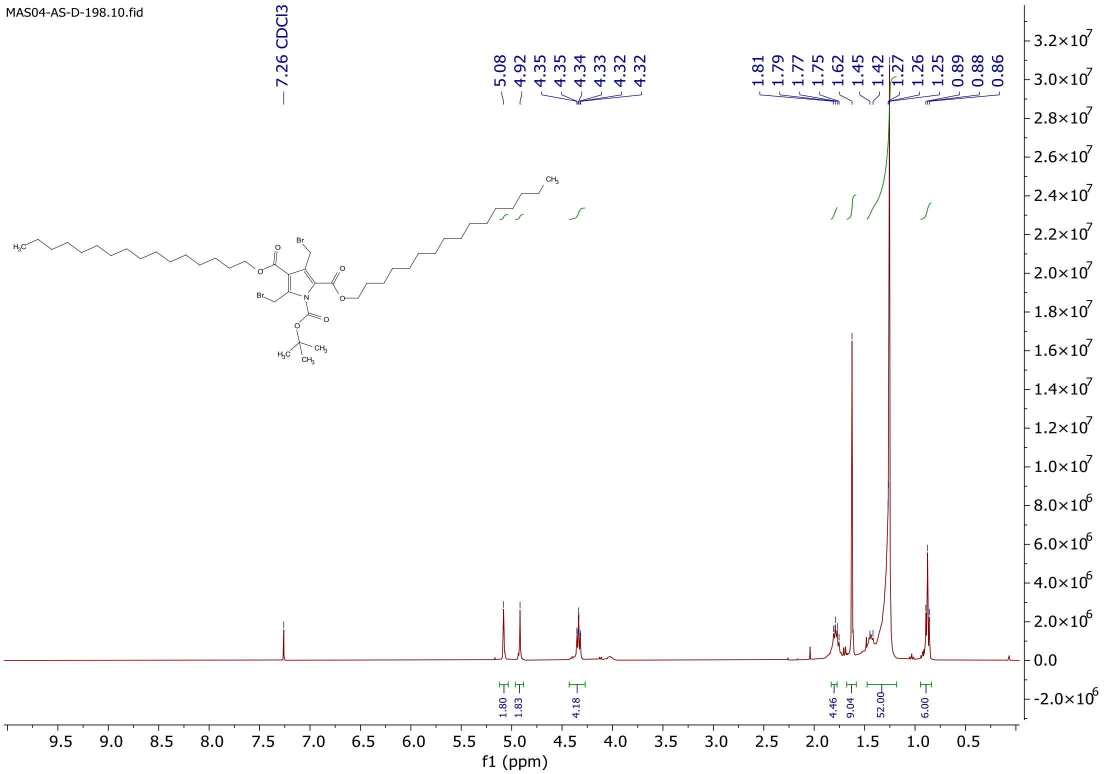

Figure S76: ^{1}H-NMR spectrum of 1-(*tert*-butyl) 2,4-dihexadecyl 3,5-bis(bromomethyl)-1*H*-pyrrole-1,2,4-tricarboxylate (**28c**)

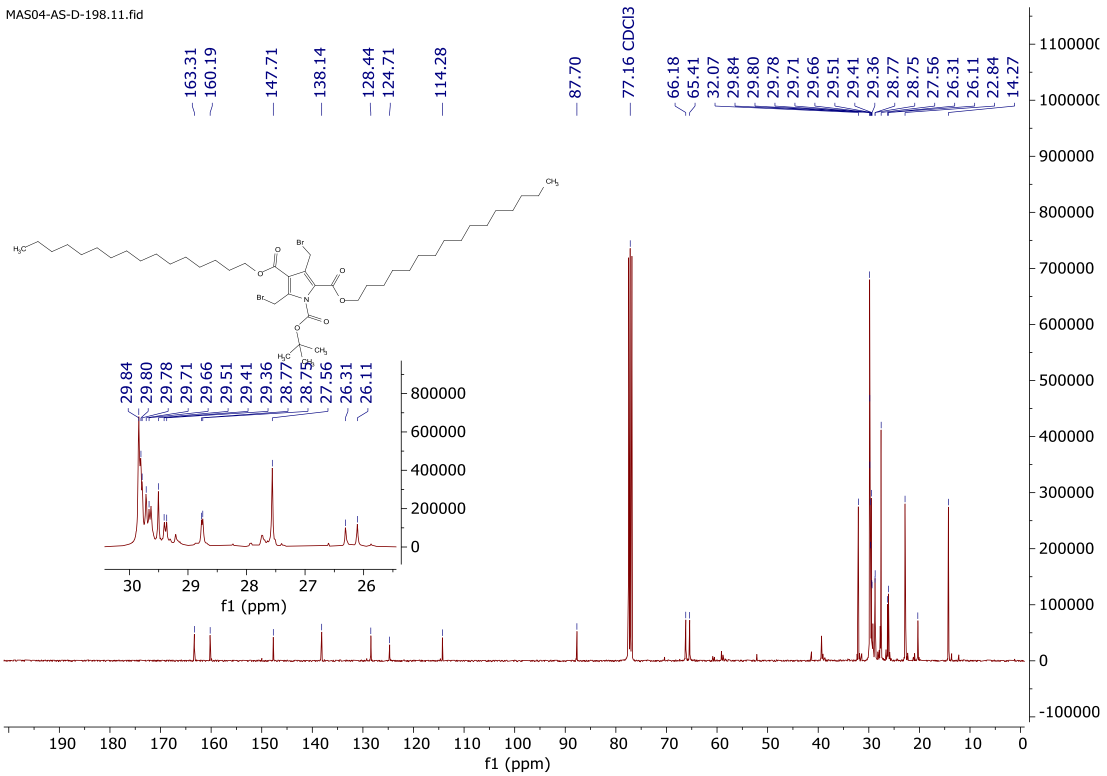

Figure S77: ^{13}C-NMR spectrum of 1-(*tert*-butyl) 2,4-dihexadecyl 3,5-bis(bromomethyl)-1*H*-pyrrole-1,2,4-tricarboxylate (**28c**)

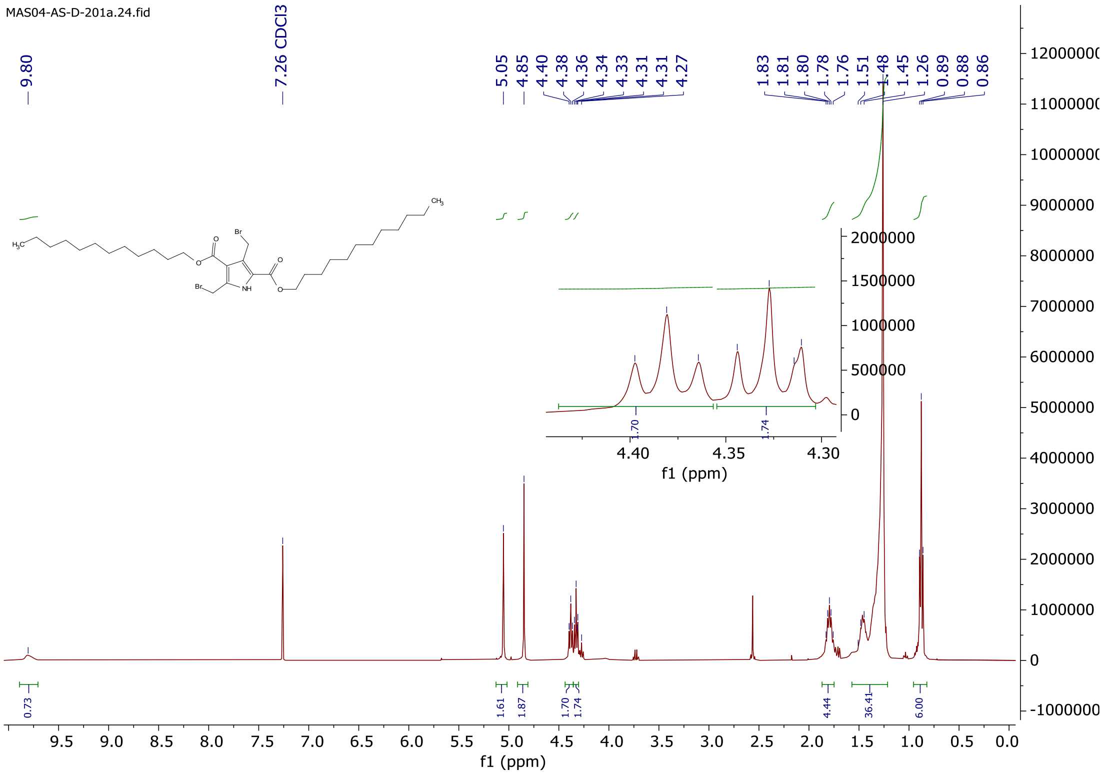

Figure S78: ^{1}H-NMR spectrum of didodecyl 3,5-bis(bromomethyl)-1*H*-pyrrole-2,4-dicarboxylate (**29a**)

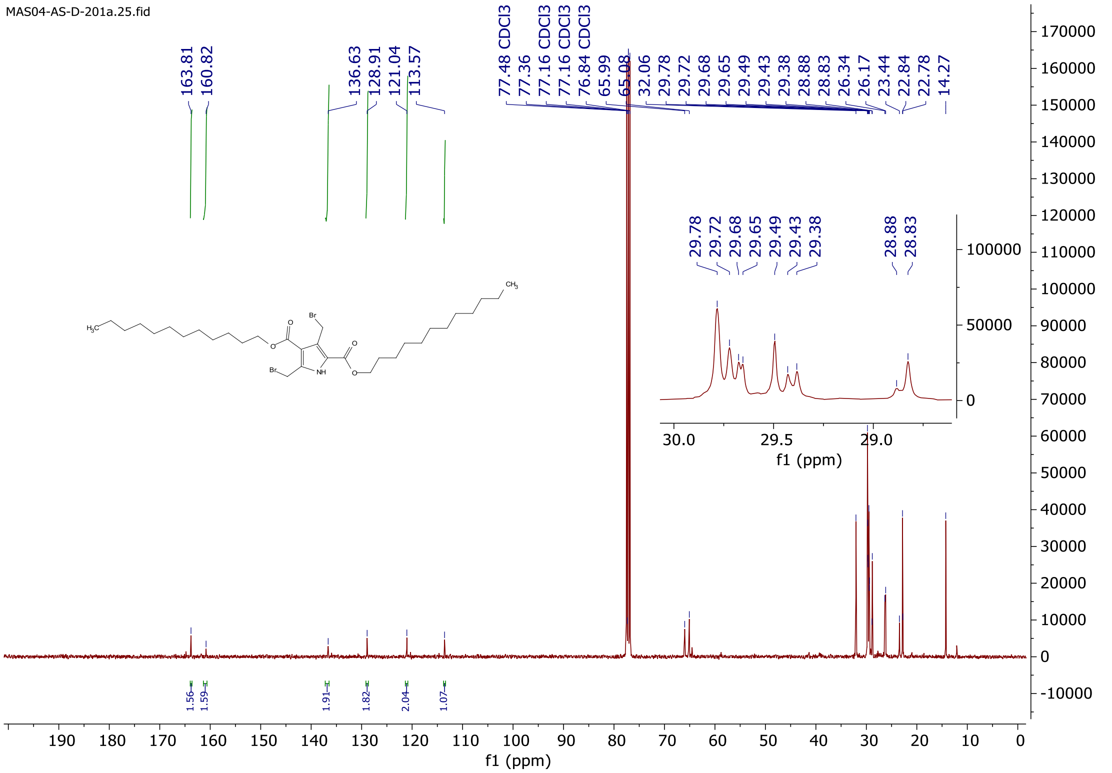

Figure S79: ^{13}C-NMR spectrum of didodecyl 3,5-bis(bromomethyl)-1*H*-pyrrole-2,4-dicarboxylate (**29a**)

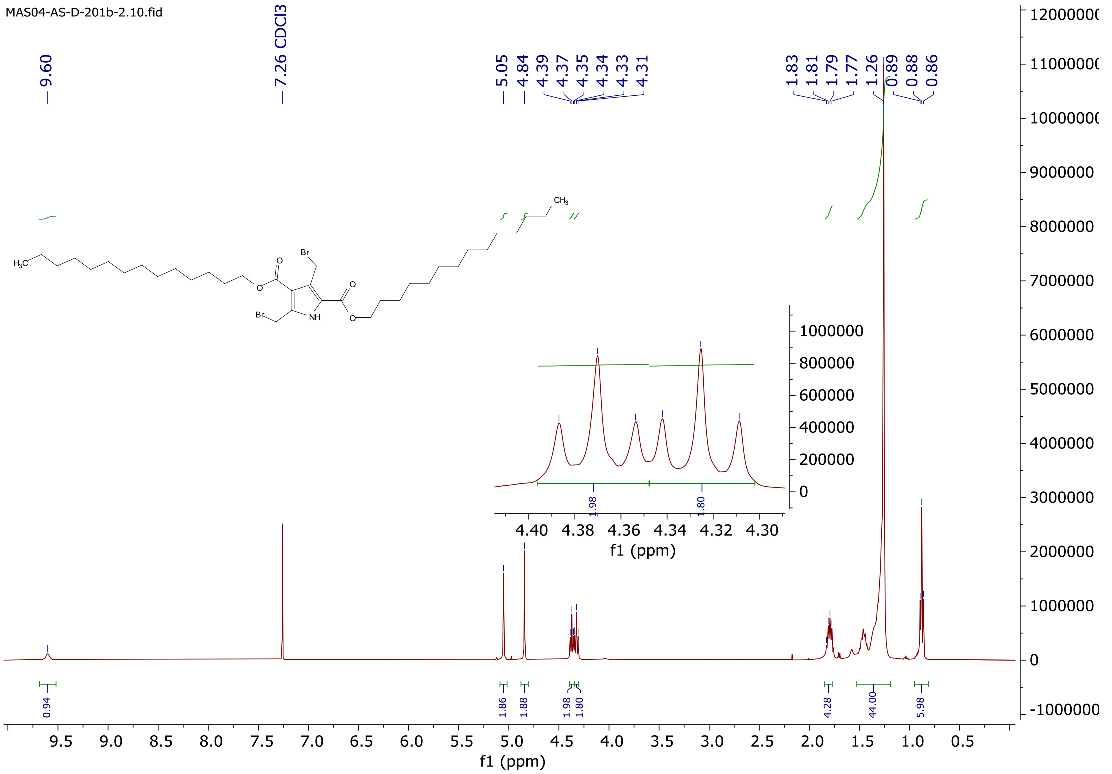

Figure S80: ^{1}H-NMR spectrum of ditetradecyl 3,5-bis(bromomethyl)-1*H*-pyrrole-2,4-dicarboxylate (**29b**)

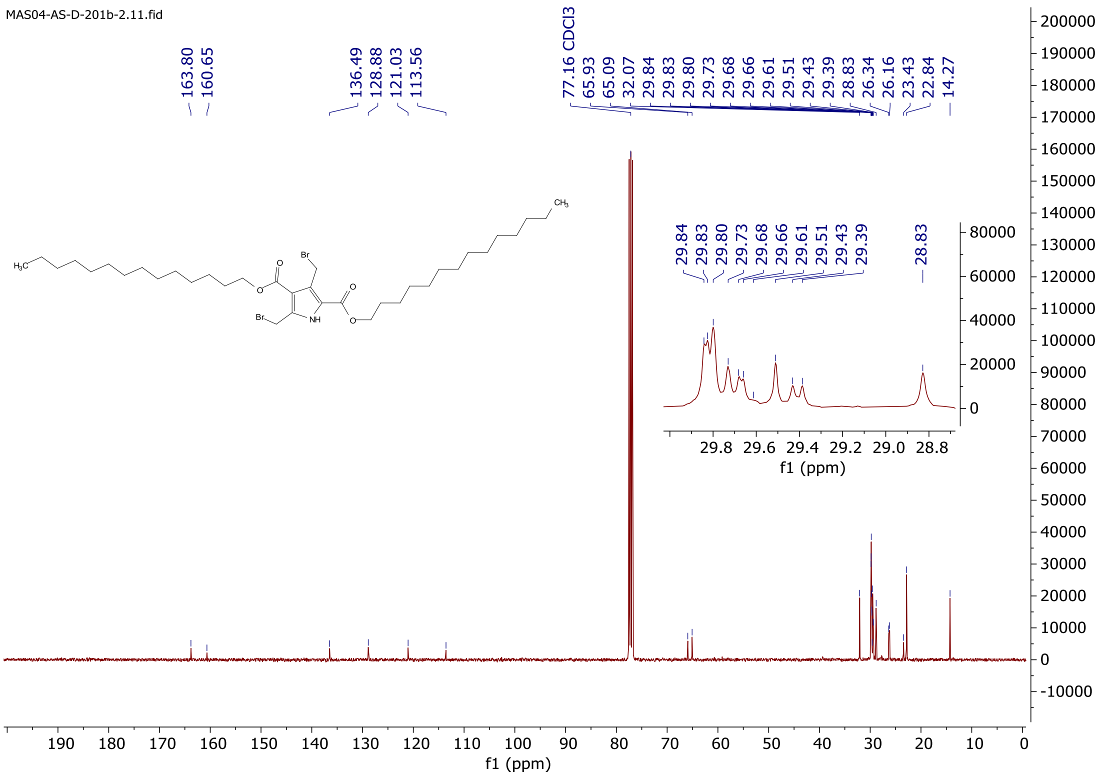

Figure S81: ^{13}C-NMR spectrum of ditetradecyl 3,5-bis(bromomethyl)-1*H*-pyrrole-2,4-dicarboxylate (**29b**)

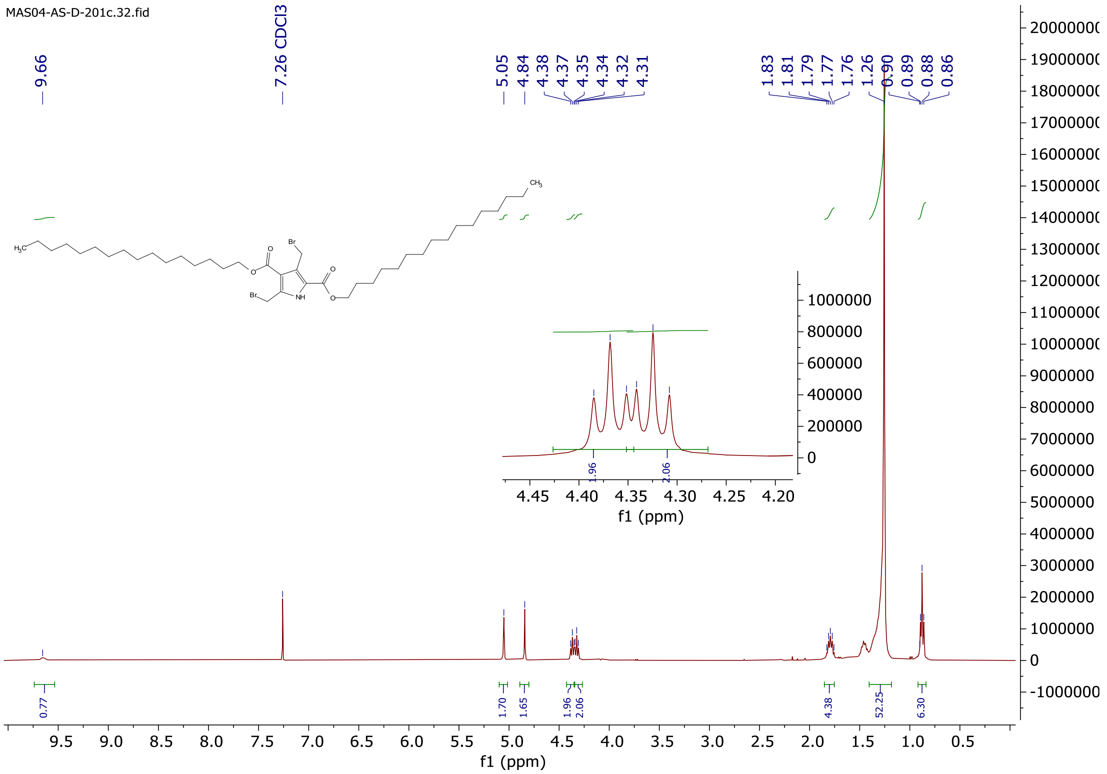

Figure S82: ^{1}H-NMR spectrum of dihexadecyl 3,5-bis(bromomethyl)-1*H*-pyrrole-2,4-dicarboxylate (**29c**)

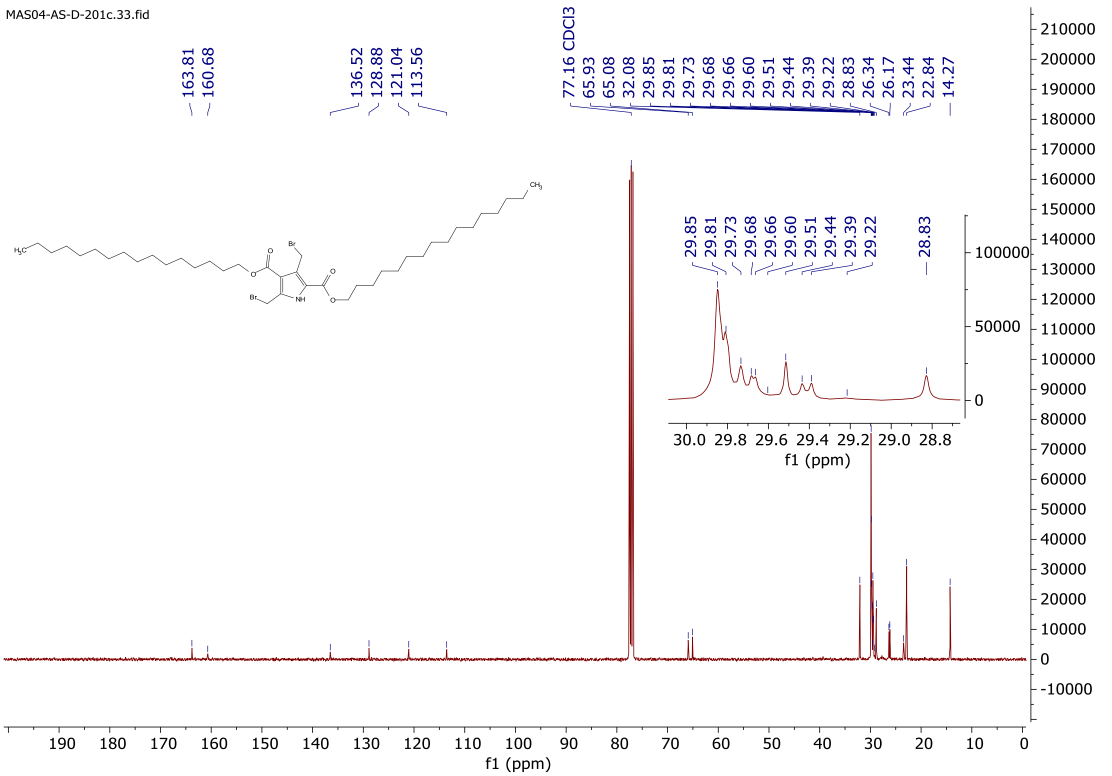

Figure S83: ^{13}C-NMR spectrum of dihexadecyl 3,5-bis(bromomethyl)-1*H*-pyrrole-2,4-dicarboxylate (**29c**)

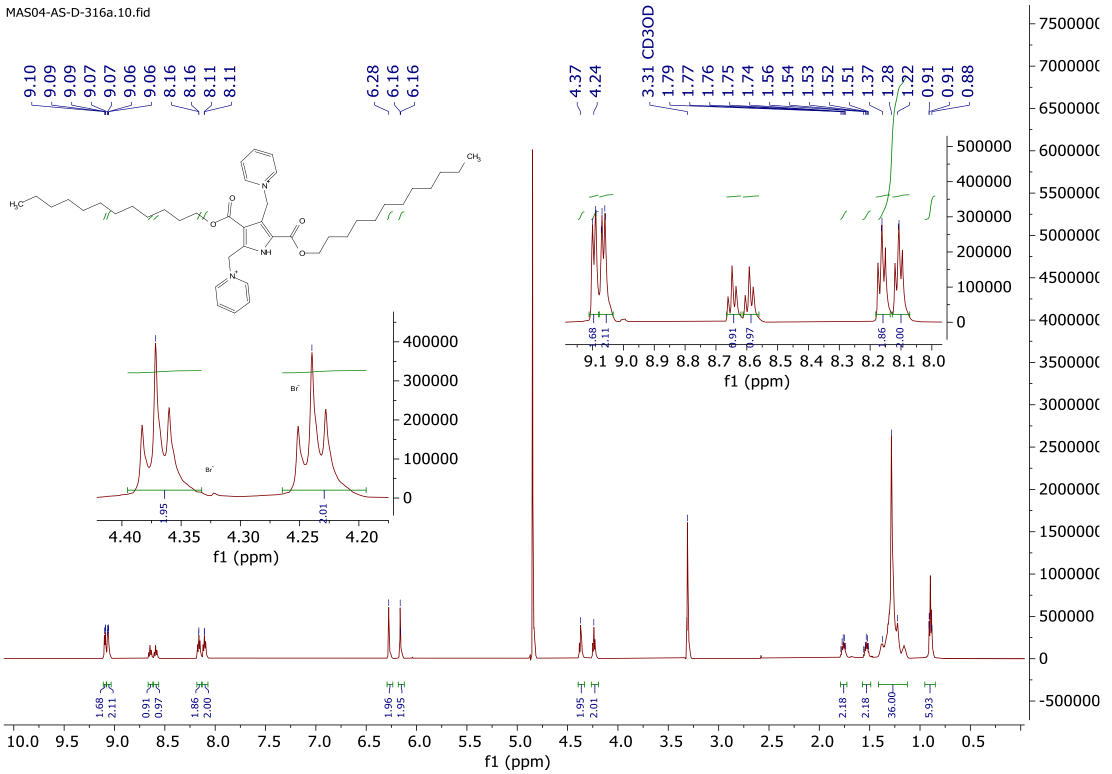

Figure S84: ^{1}H-NMR spectrum of 1,1'-((3,5-bis((dodecyloxy)carbonyl)-1*H*-pyrrole-2,4-diyl)bis(methylene))bis(pyridin-1-ium) dibromide (**6a**)

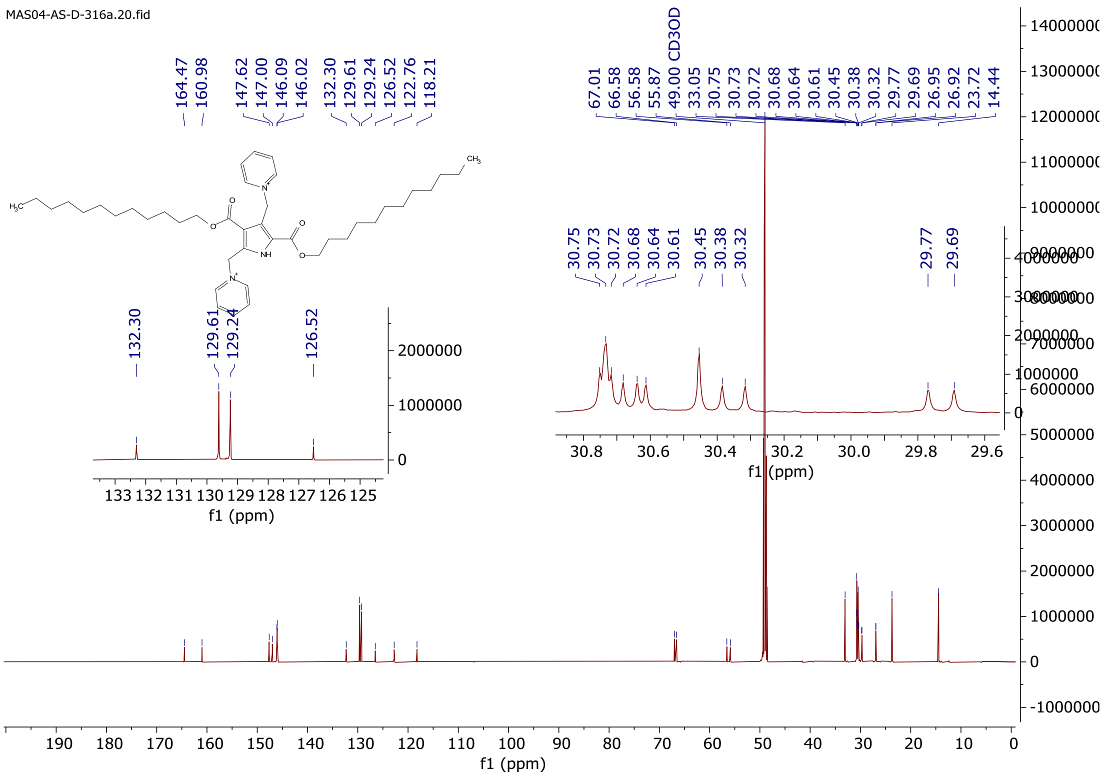

Figure S85: ^{13}C-NMR spectrum of 1,1'-((3,5-bis((dodecyloxy)carbonyl)-1*H*-pyrrole-2,4-diyl)bis(methylene))bis(pyridin-1-ium) dibromide (**6a**)

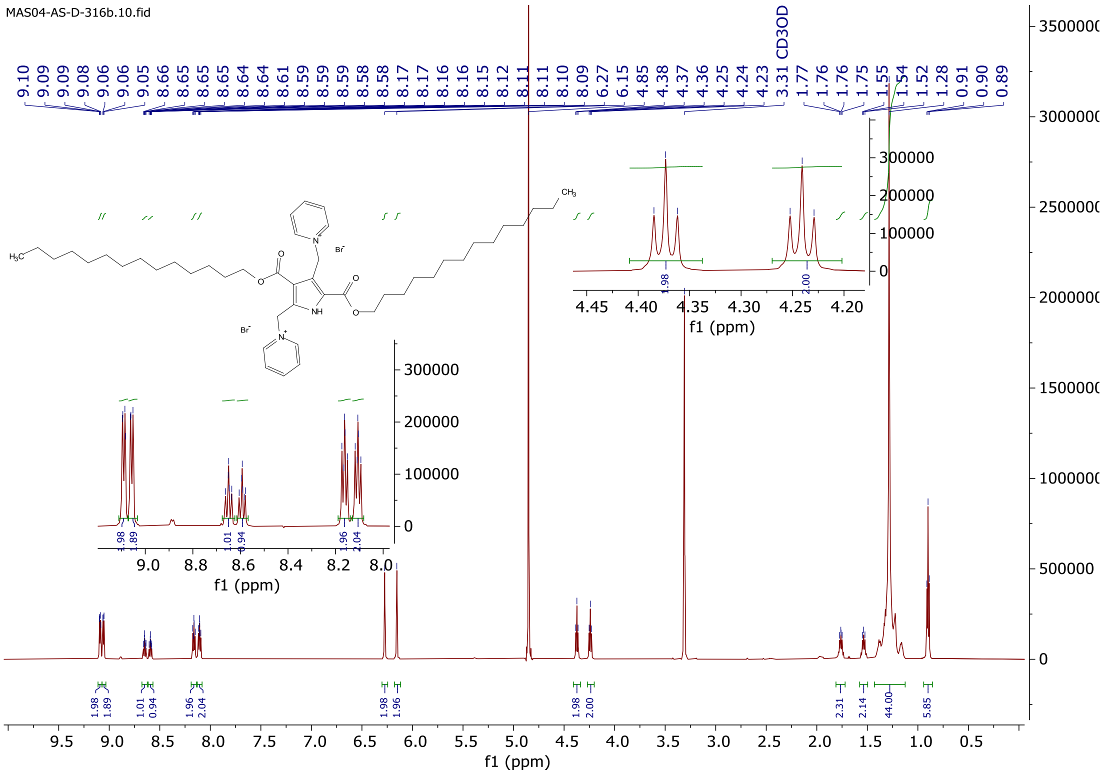

Figure S86: ^{1}H-NMR spectrum of 1,1'-((3,5-bis((tetradecyloxy)carbonyl)-1*H*-pyrrole-2,4-diyl)bis(methylene))bis(pyridin-1-ium) dibromide (**6b**)

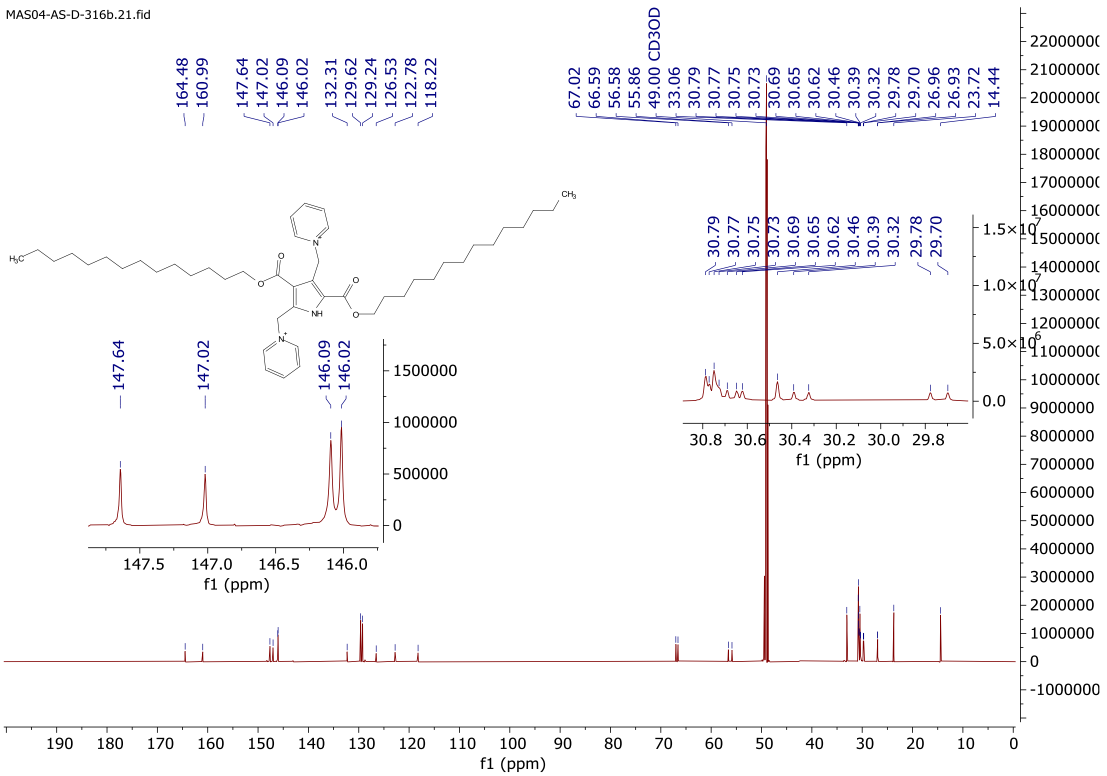

Figure S87: ^{13}C-NMR spectrum of 1,1'-((3,5-bis((tetradecyloxy)carbonyl)-1*H*-pyrrole-2,4-diyl)bis(methylene))bis(pyridin-1-ium) dibromide (**6b**)

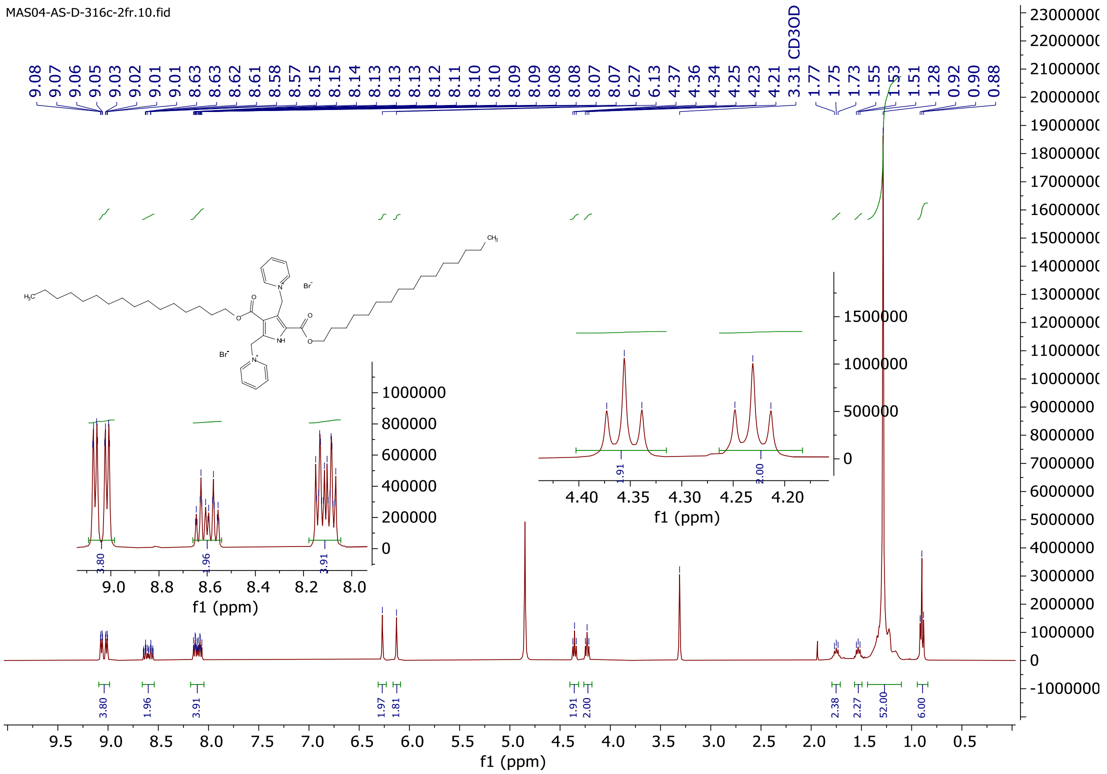

Figure S88: ^{1}H-NMR spectrum of 1,1'-((3,5-bis((hexadecyloxy)carbonyl)-1*H*-pyrrole-2,4-diyl)bis(methylene))bis(pyridin-1-ium) dibromide (**6c**)

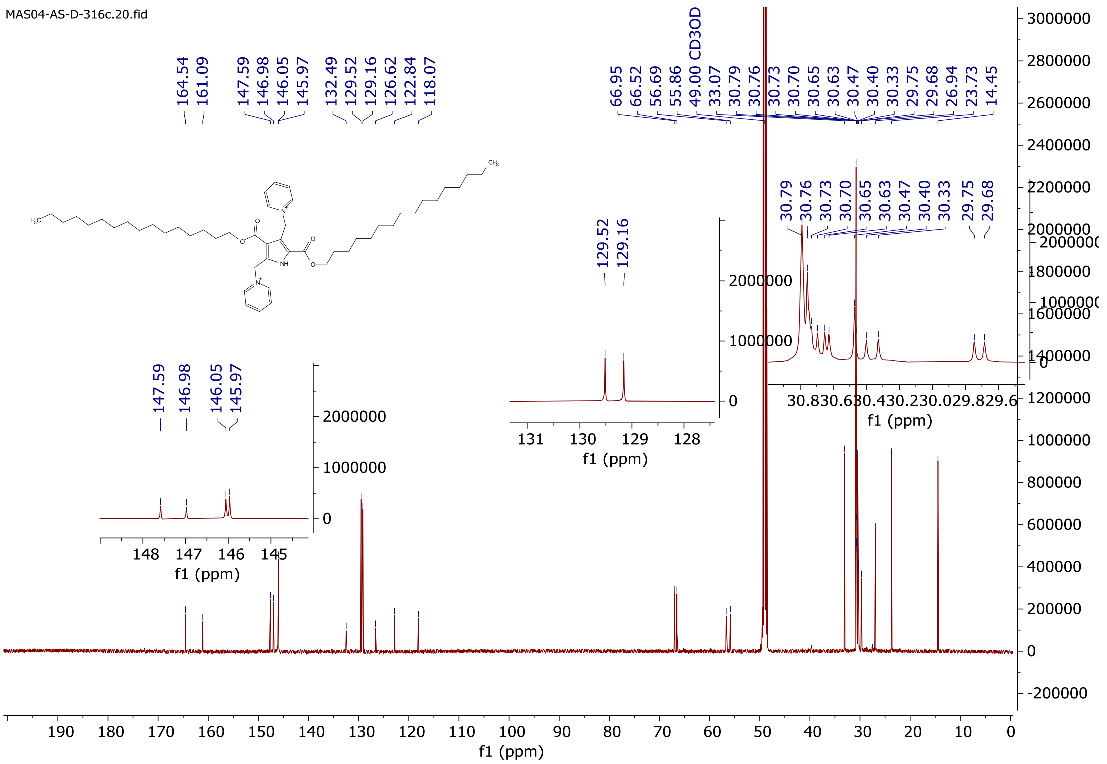

Figure S89: ^{13}C-NMR spectrum of 1,1'-((3,5-bis((hexadecyloxy)carbonyl)-1*H*-pyrrole-2,4-diyl)bis(methylene))bis(pyridin-1-ium) dibromide (**6c**)

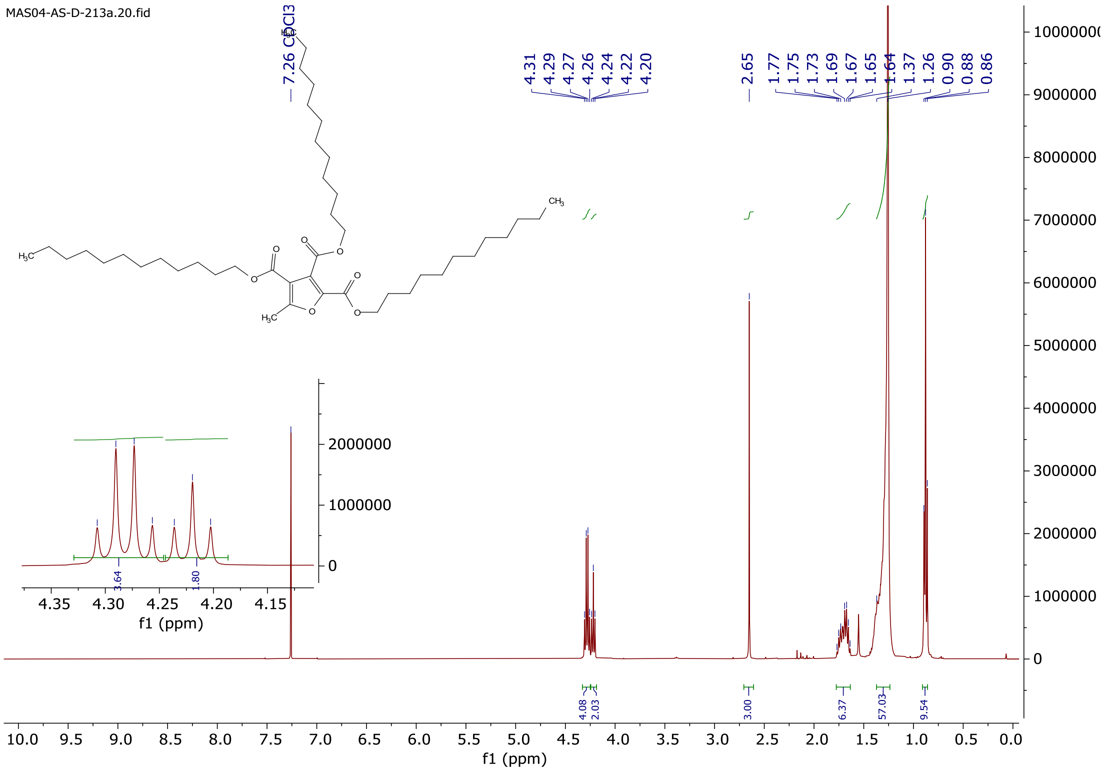

Figure S90: ^{1}H-NMR spectrum of tridodecyl 5-methylfuran-2,3,4-tricarboxylate (**34a**)

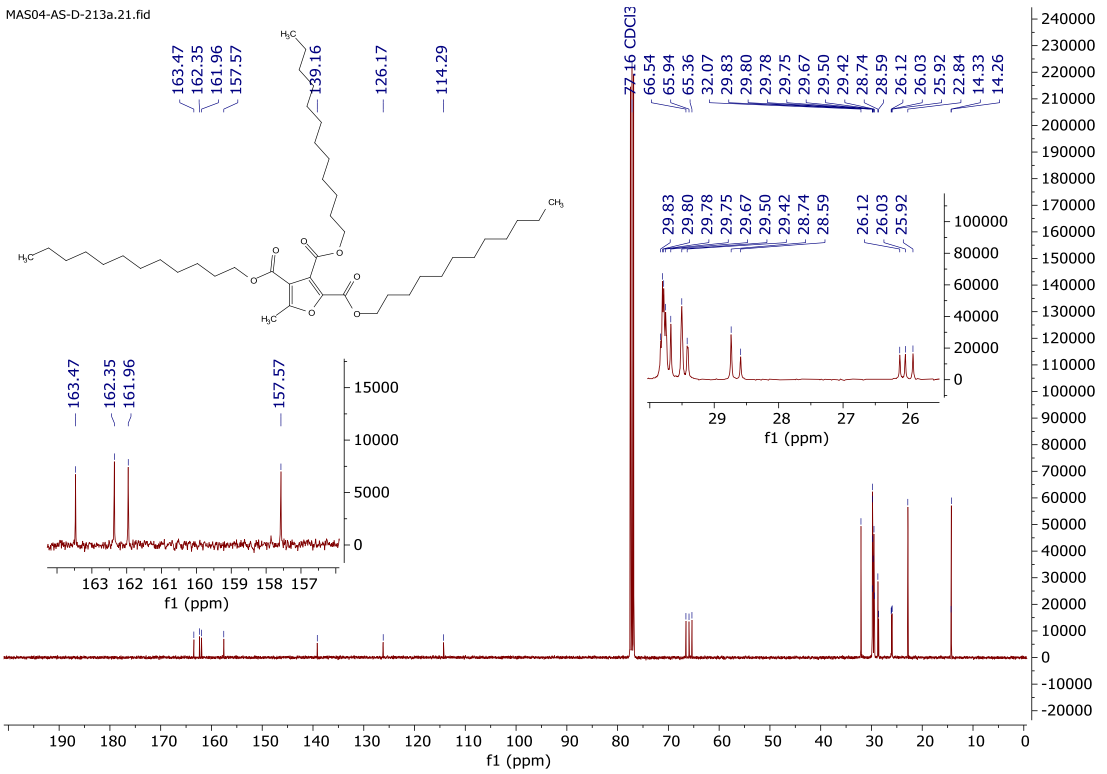

Figure S91: ^{13}C-NMR spectrum of tridodecyl 5-methylfuran-2,3,4-tricarboxylate (**34a**)

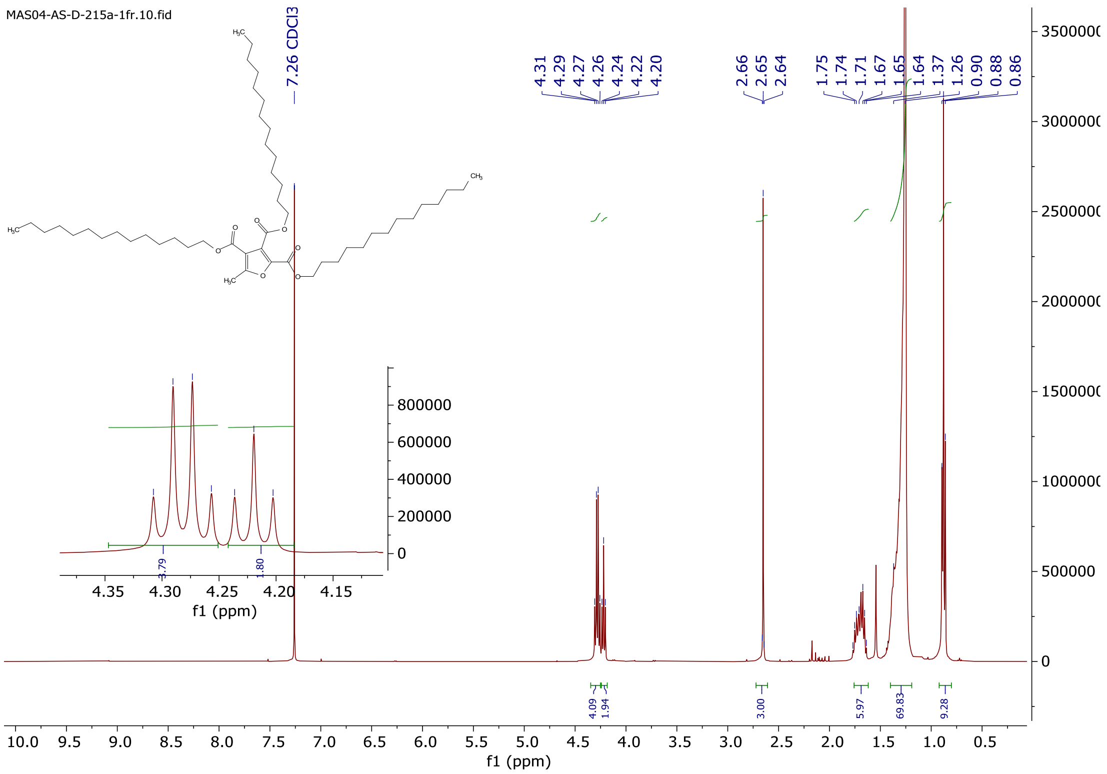

Figure S92: ^{1}H-NMR spectrum of tritetradecyl 5-methylfuran-2,3,4-tricarboxylate (**34b**)

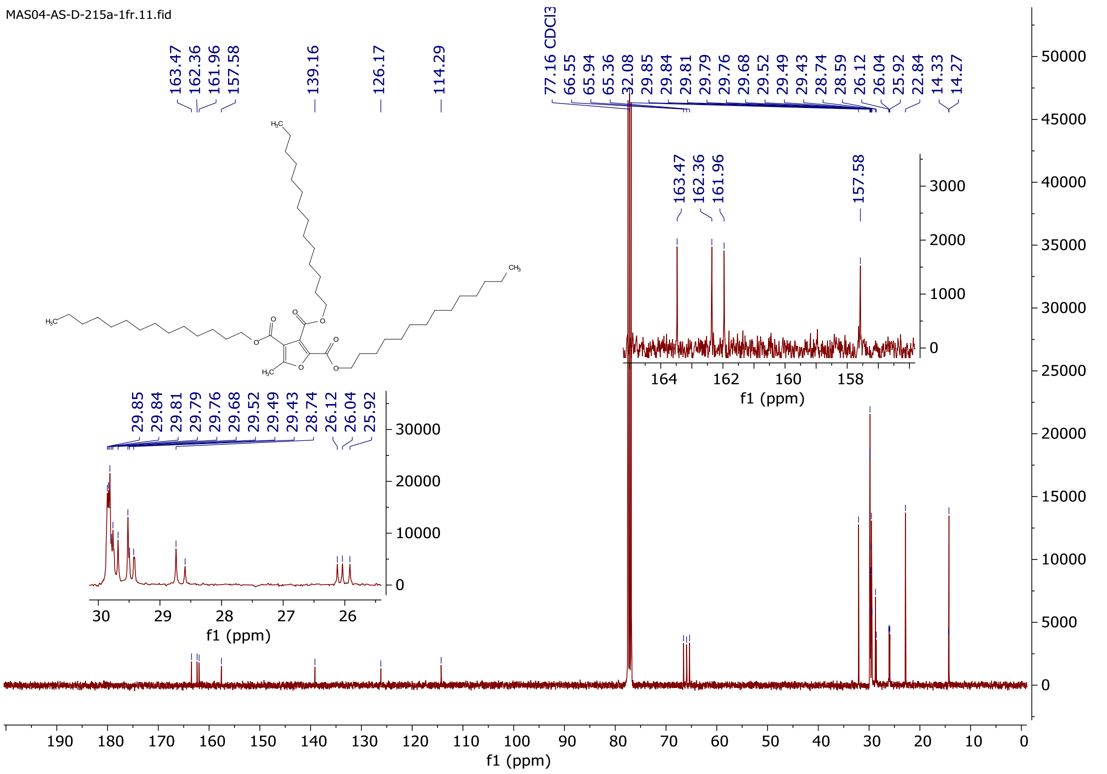

Figure S93: ^{13}C-NMR spectrum of tritetradecyl 5-methylfuran-2,3,4-tricarboxylate (**34b**)

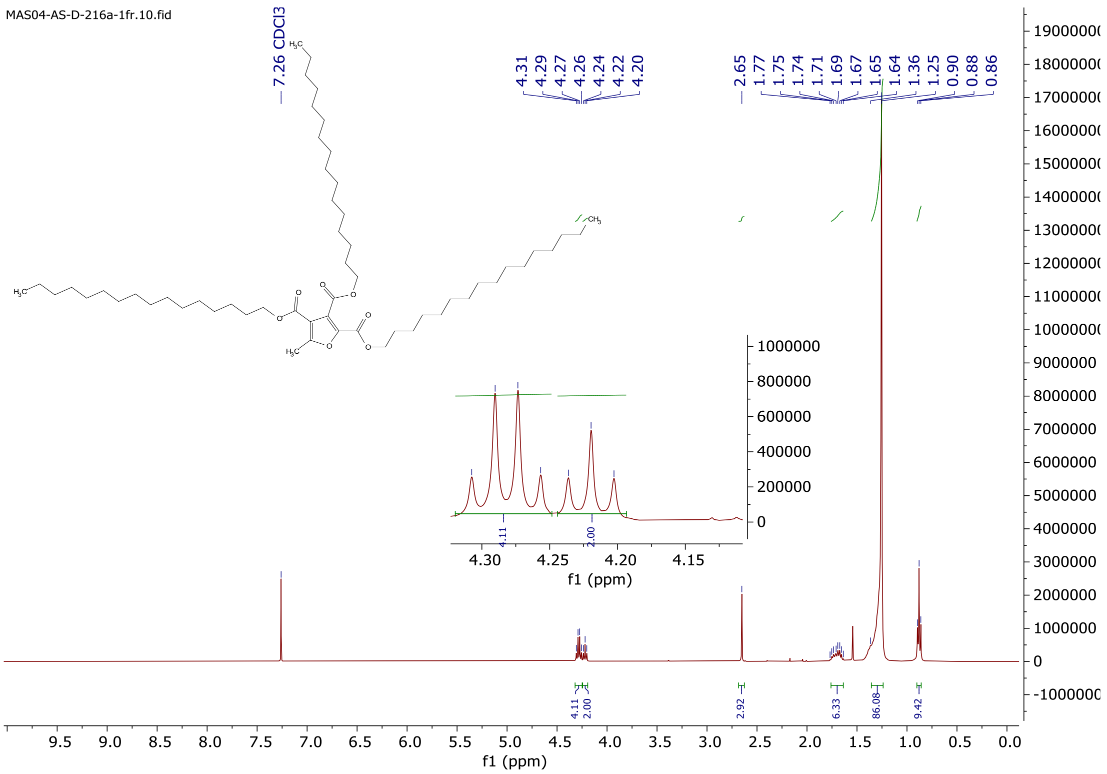

Figure S94: ^{1}H-NMR spectrum of trihexadecyl 5-methylfuran-2,3,4-tricarboxylate (**34c**)

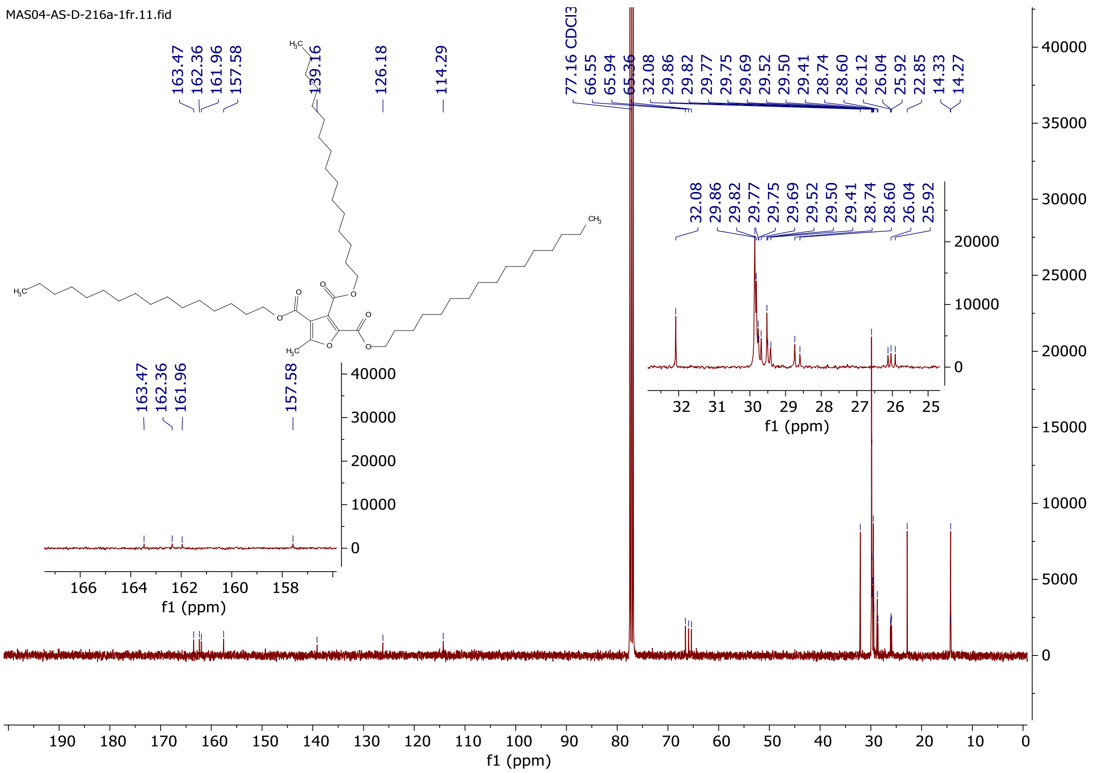

Figure S95: ^{13}C-NMR spectrum of trihexadecyl 5-methylfuran-2,3,4-tricarboxylate (**34c**)

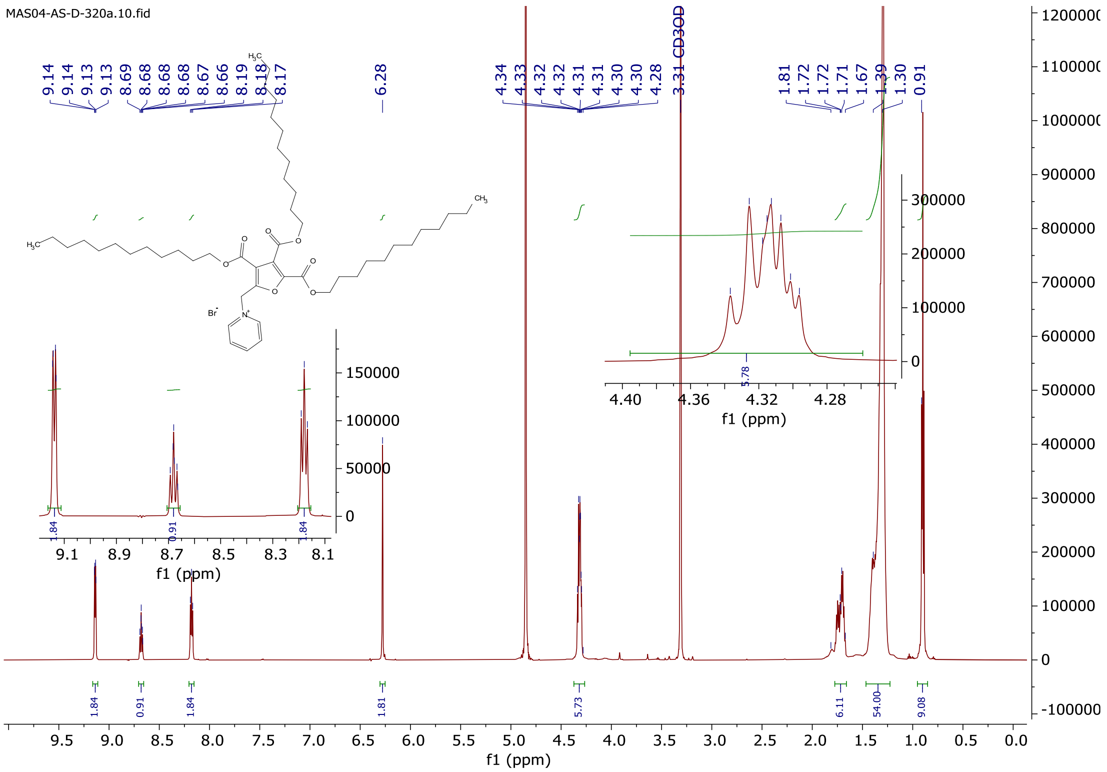

Figure S96: ^{1}H-NMR spectrum of 1-((3,4,5-tris((dodecyloxy)carbonyl)furan-2-yl)methyl)pyridin-1-ium bromide (**7a**)

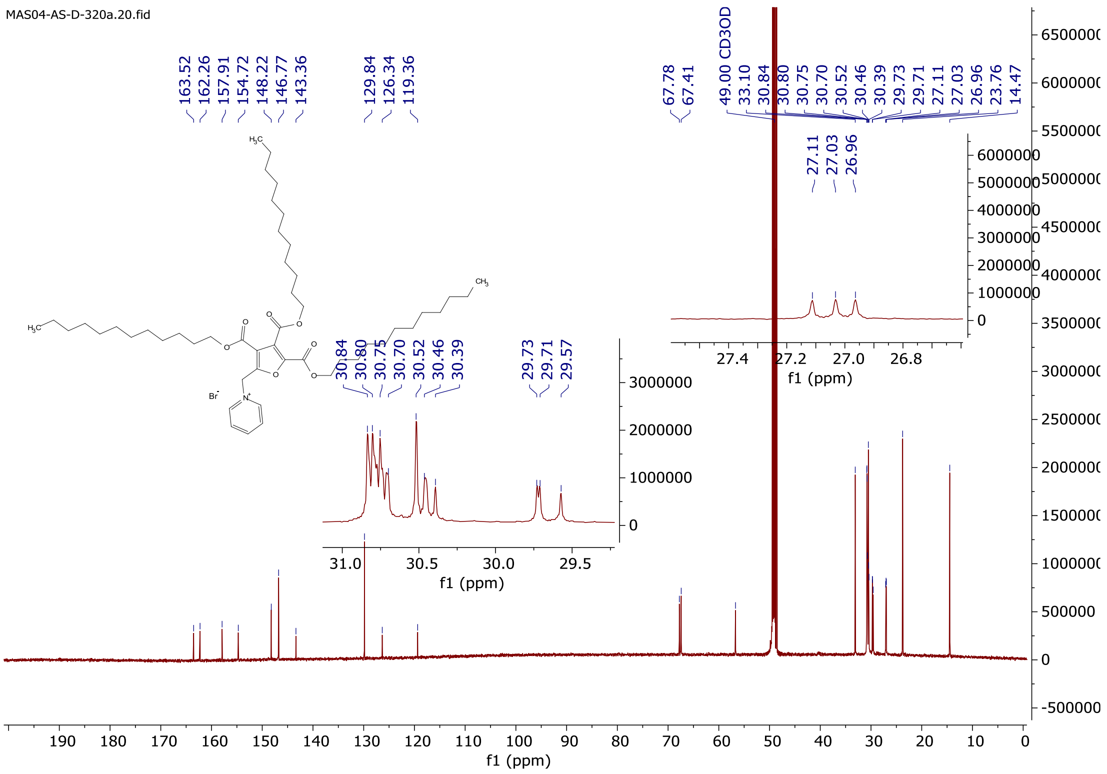

Figure S97: ^{13}C-NMR spectrum of 1-((3,4,5-tris((dodecyloxy)carbonyl)furan-2-yl)methyl)pyridin-1-ium bromide (**7a**)

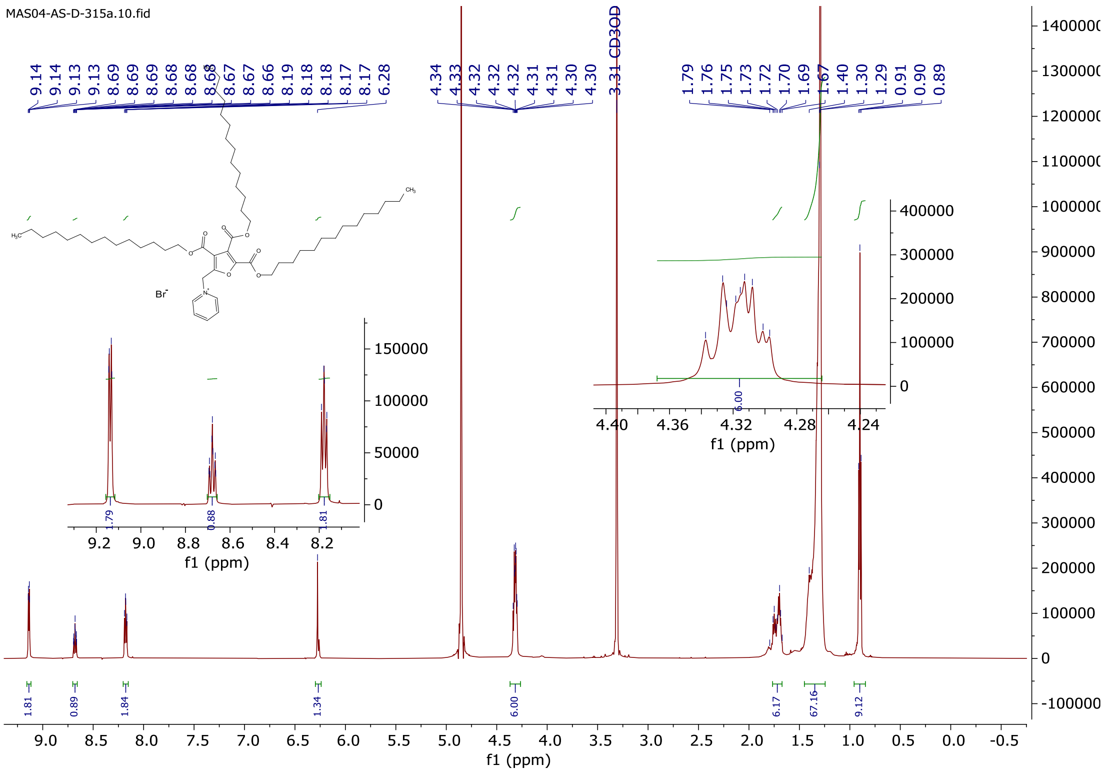

Figure S98: ^{1}H-NMR spectrum of 1-((3,4,5-tris((tetradecyloxy)carbonyl)furan-2-yl)methyl)pyridin-1-ium bromide (**7b**)

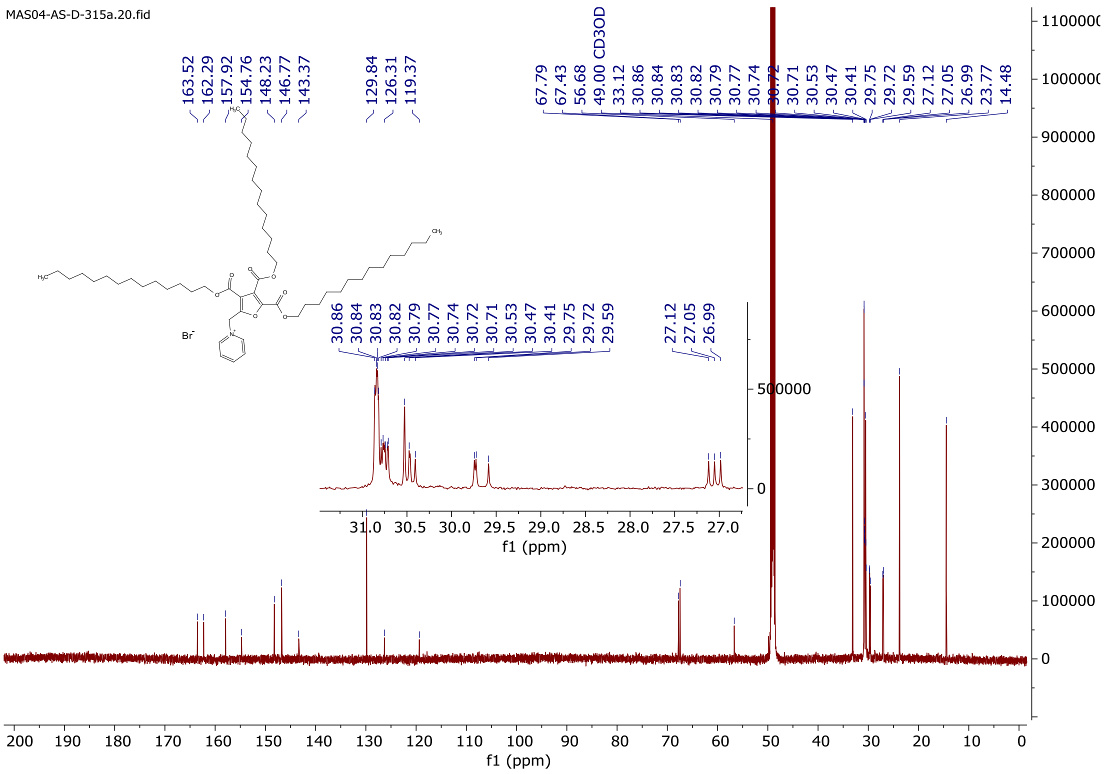

Figure S99: ^{13}C-NMR spectrum of 1-((3,4,5-tris((tetradecyloxy)carbonyl)furan-2-yl)methyl)pyridin-1-ium bromide (**7b**)

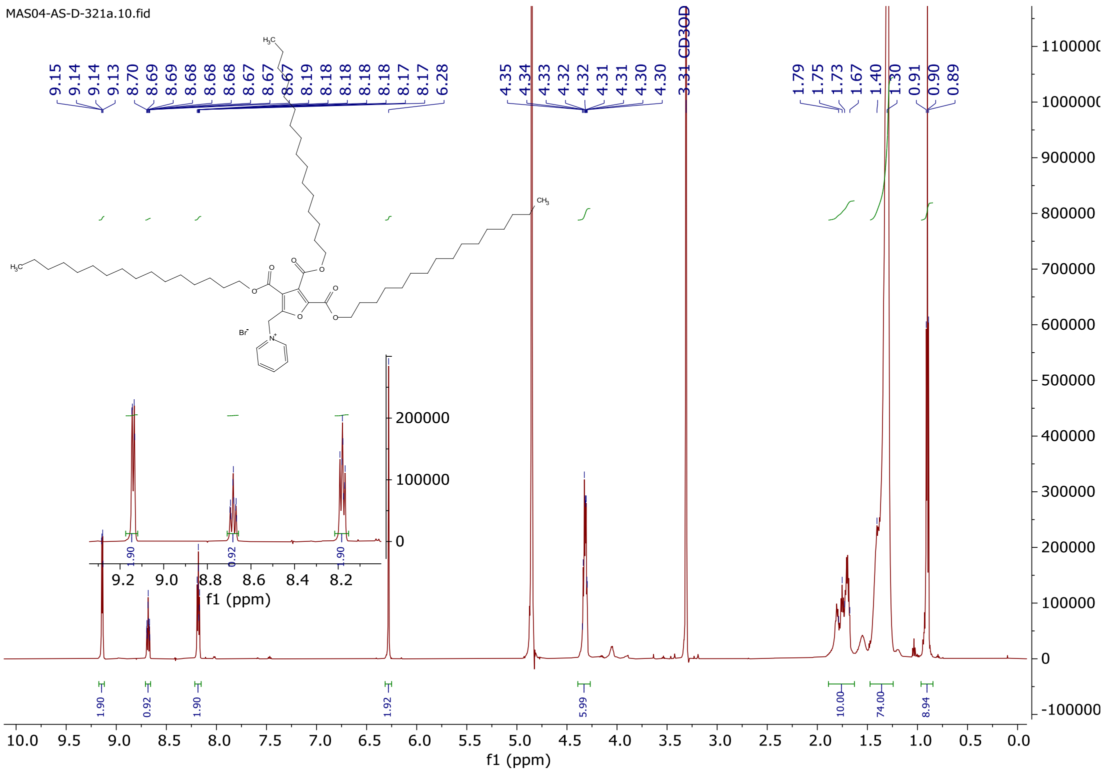

Figure S100: ^{1}H-NMR spectrum of 1-((3,4,5-tris((hexadecyloxy)carbonyl)furan-2-yl)methyl)pyridin-1-ium bromide (**7c**)

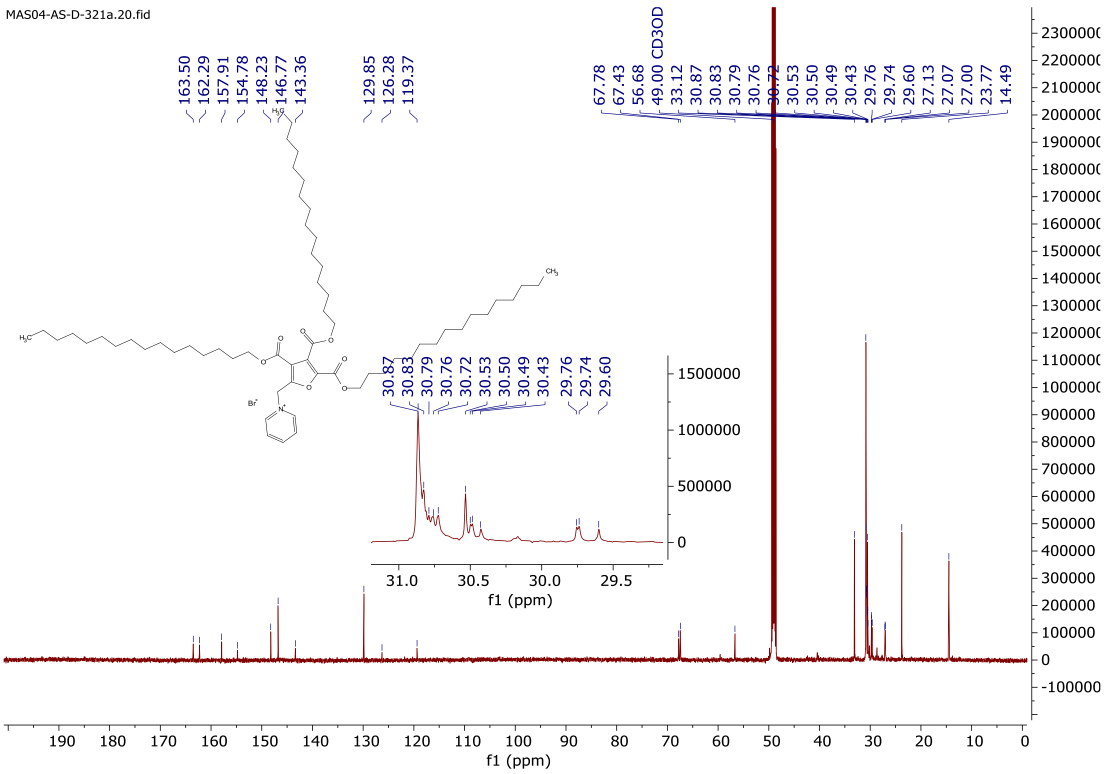

Figure S101: ^{13}C-NMR spectrum of 1-((3,4,5-tris((hexadecyloxy)carbonyl)furan-2-yl)methyl)pyridin-1-ium bromide (**7c**)

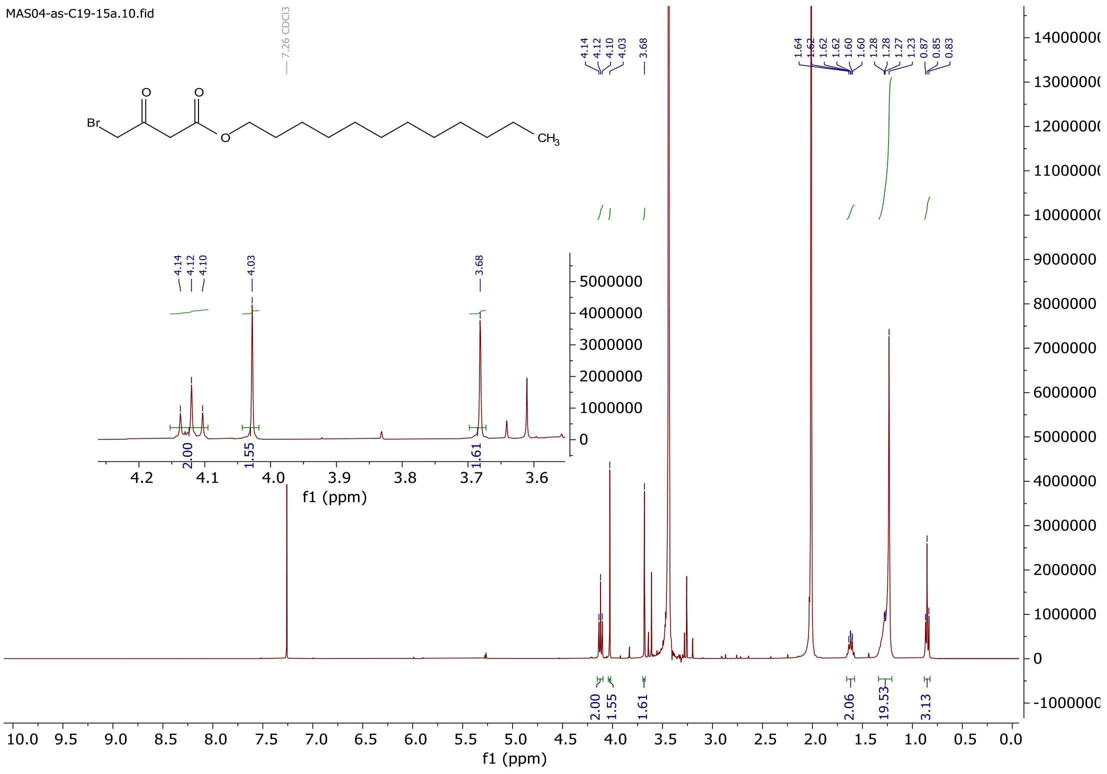

Figure S102 ^{1}H-NMR spectrum of crude intermediate of dodecyl 4-bromo-3-oxobutanoate **(38a)**

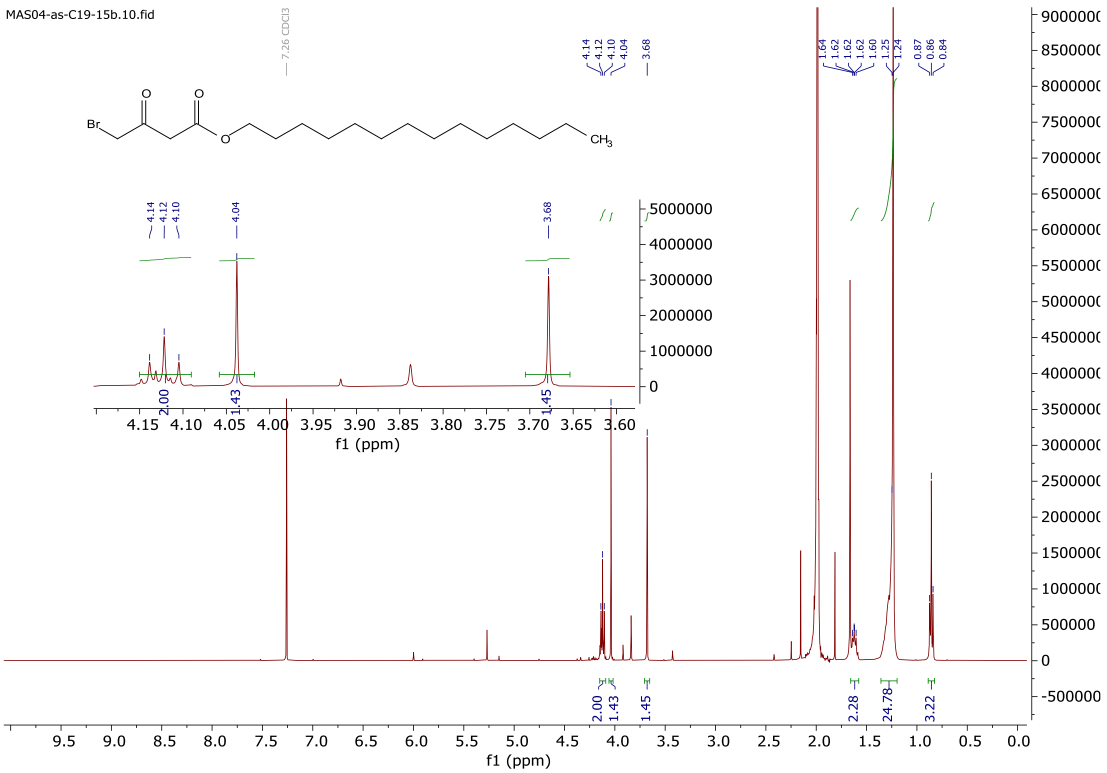

Figure S103 ^{1}H-NMR spectrum of crude intermediate of tetradecyl 4-bromo-3-oxobutanoate **(38b)**

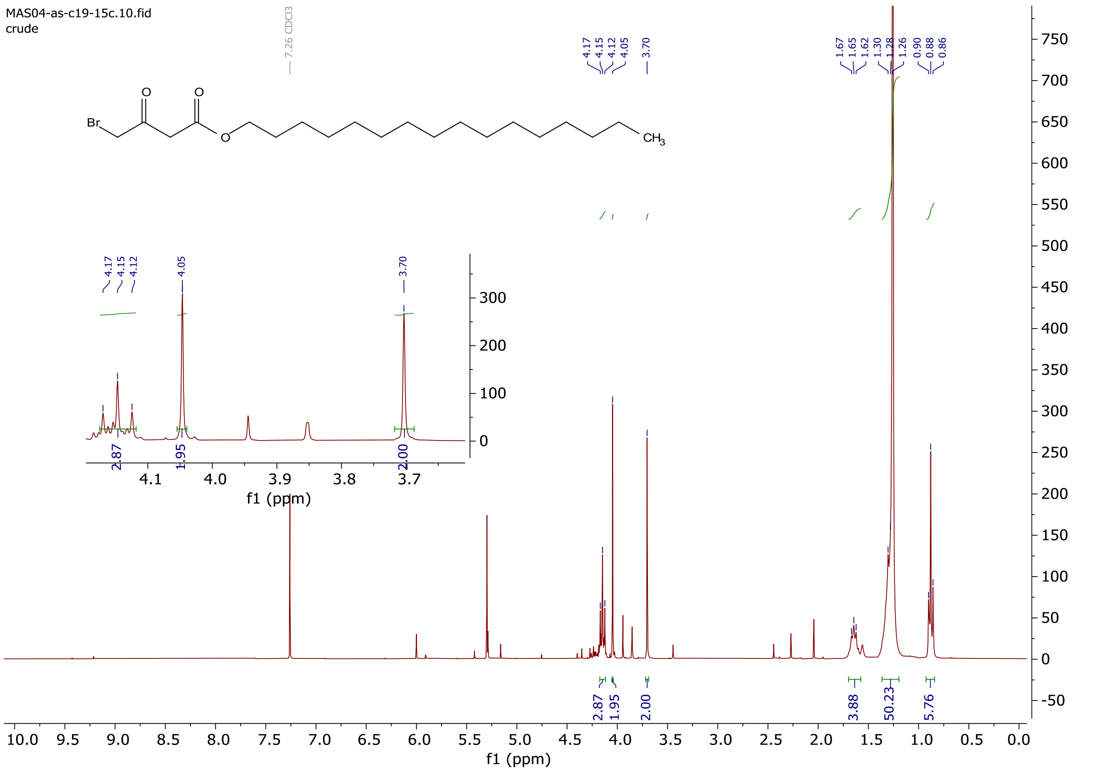

Figure S104 ^{1}H-NMR spectrum of crude intermediate of hexadecyl 4-bromo-3-oxobutanoate **(38c)**

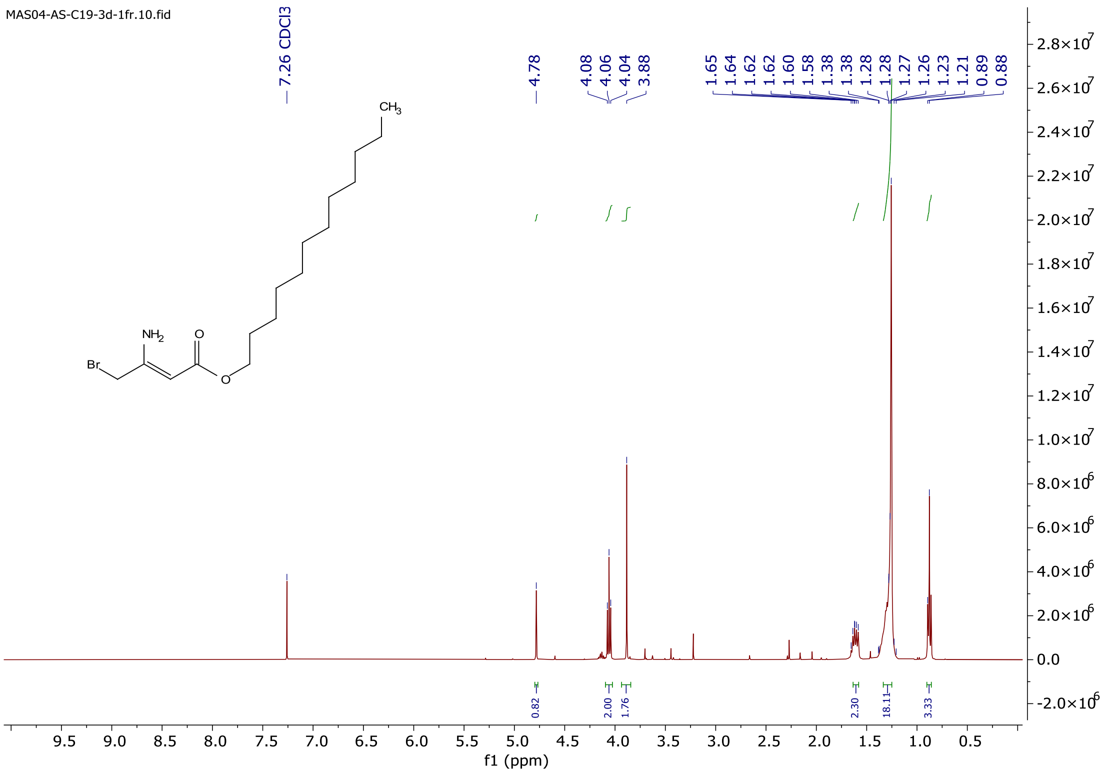

Figure S105: ^{1}H-NMR spectrum of dodecyl (*Z*)-3-amino-4-bromobut-2-enoate (**40a**)

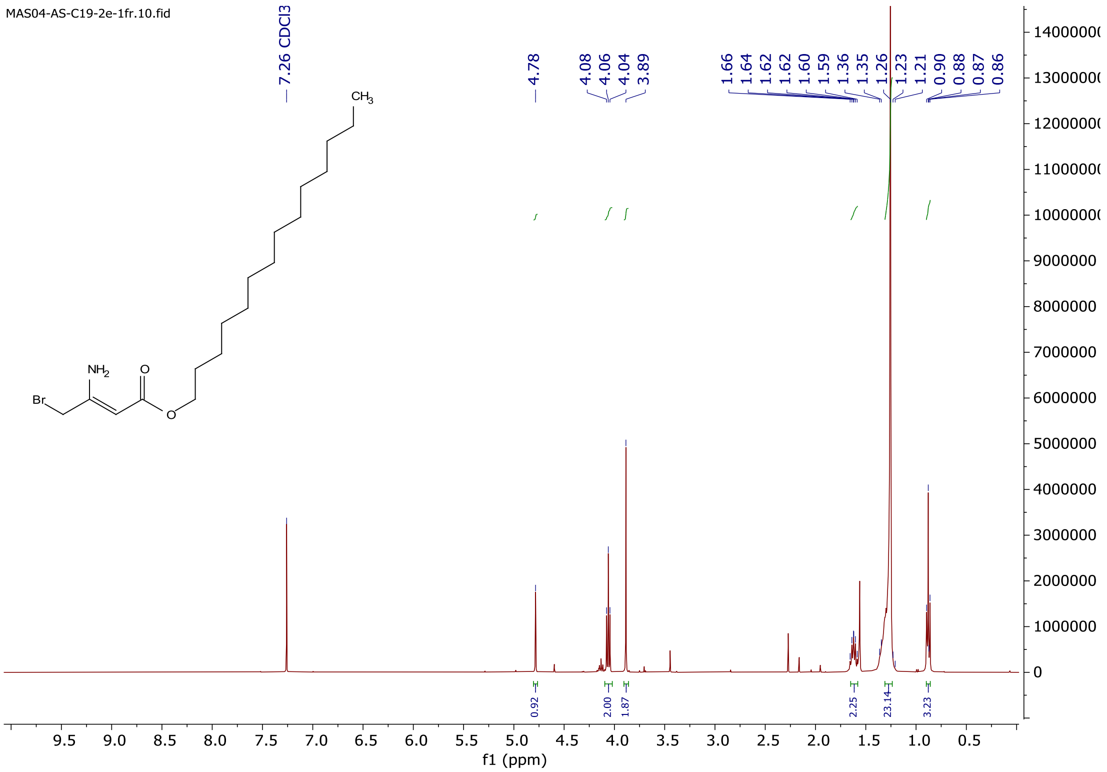

Figure S106: ^{1}H-NMR spectrum of tetradecyl (*Z*)-3-amino-4-bromobut-2-enoate (**40b**)

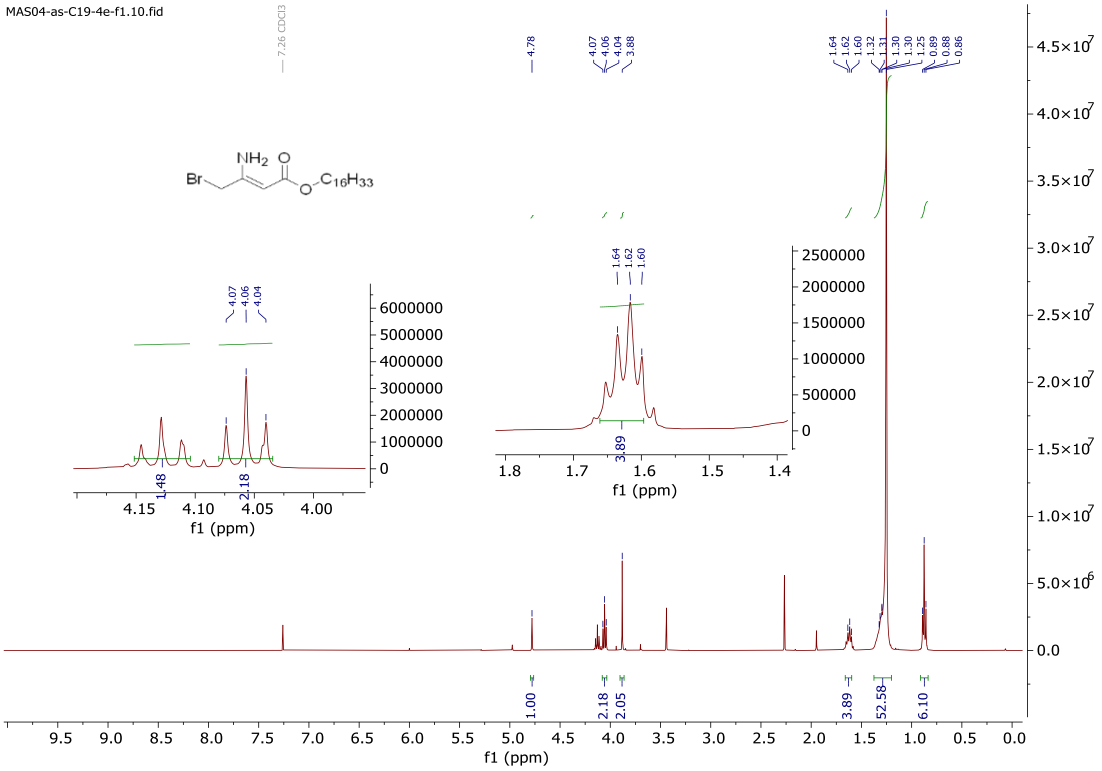

Figure S107: ^{1}H-NMR spectrum of hexadecyl (*Z*)-3-amino-4-bromobut-2-enoate (**40c**)

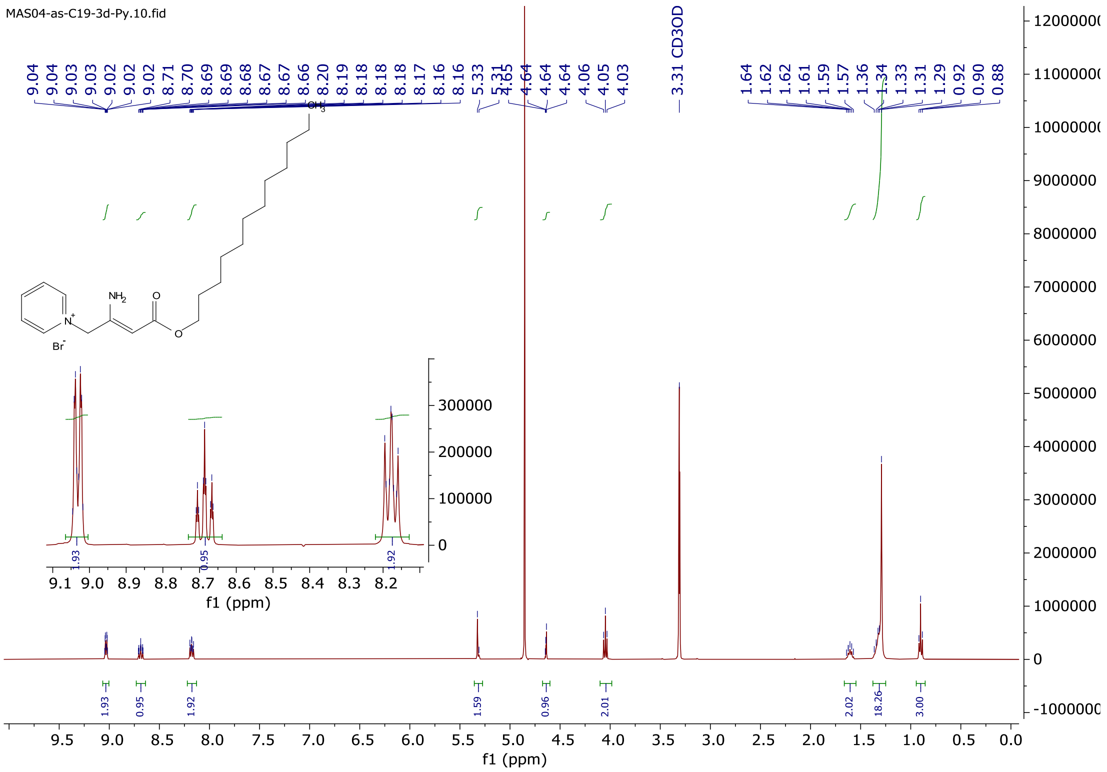

Figure S108: ^{1}H-NMR spectrum of (*Z*)-1-(2-amino-4-(dodecyloxy)-4-oxobut-2-en-1-yl)pyridin-1-ium bromide (**9a**)

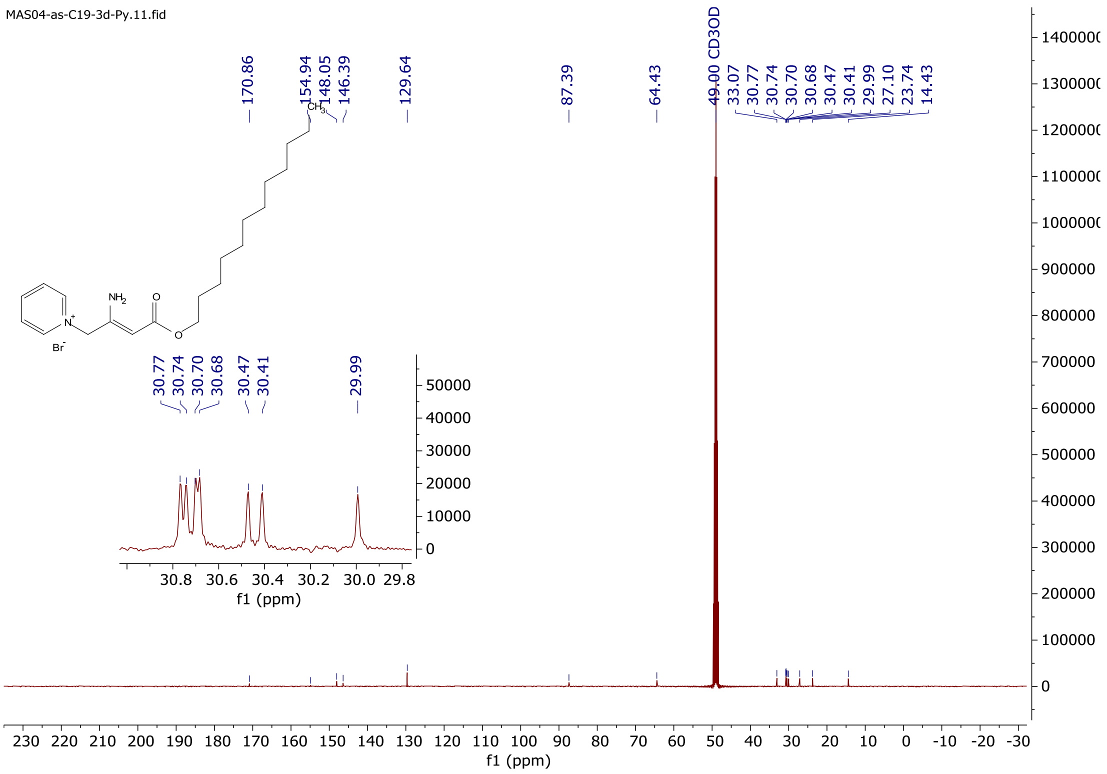

Figure S109: ^{13}C-NMR spectrum of (*Z*)-1-(2-amino-4-(dodecyloxy)-4-oxobut-2-en-1-yl)pyridin-1-ium bromide (**9a**)

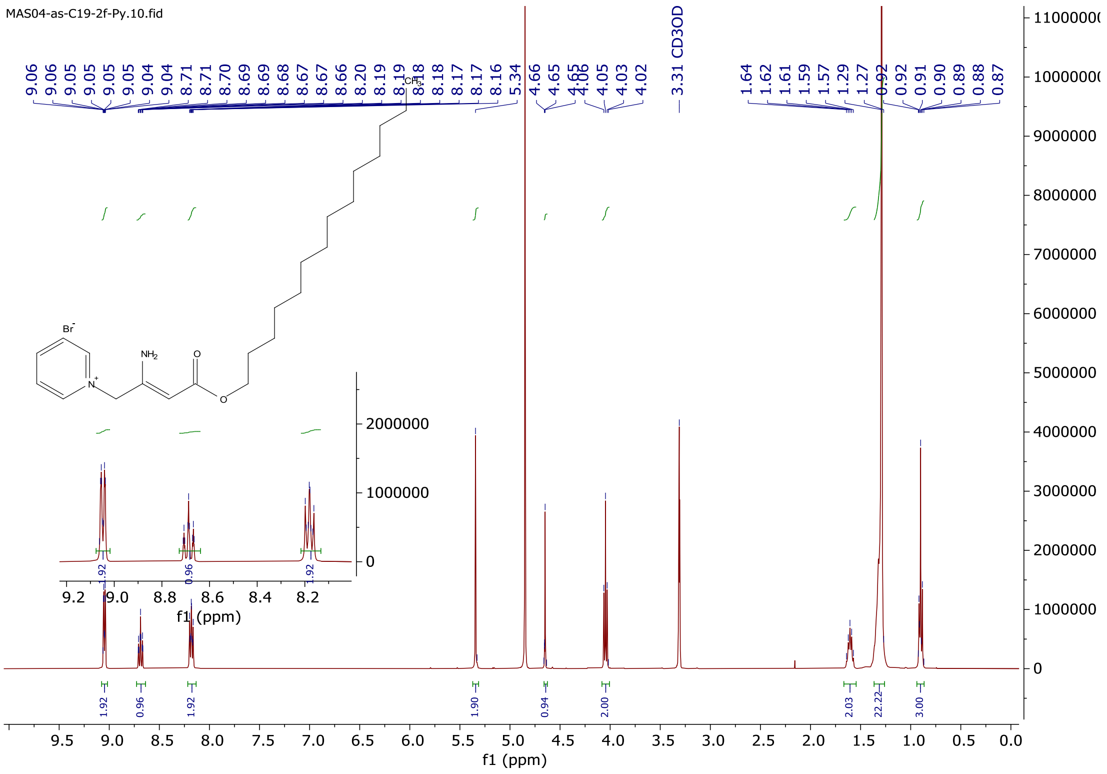

Figure S110: ^{1}H-NMR spectrum of (*Z*)-1-(2-amino-4-oxo-4-(tetradecyloxy)but-2-en-1-yl)pyridin-1-ium bromide (**9b**)

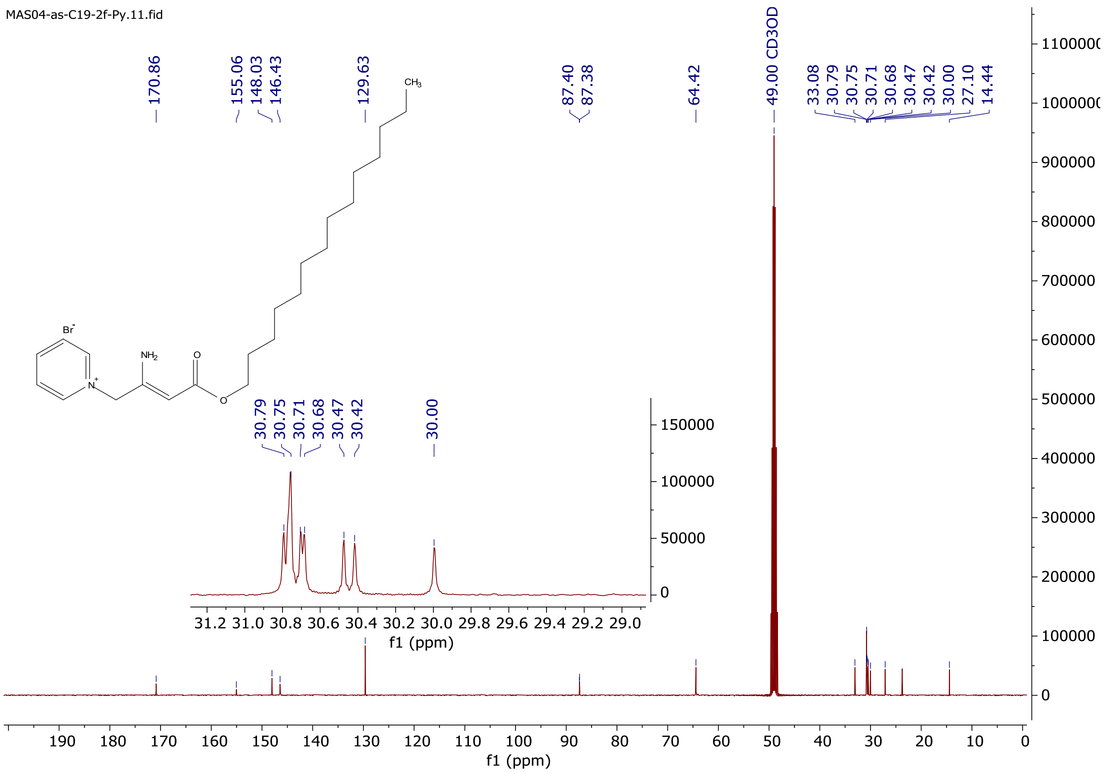

Figure S111: ^{13}C-NMR spectrum of (*Z*)-1-(2-amino-4-oxo-4-(tetradecyloxy)but-2-en-1-yl)pyridin-1-ium bromide (**9b**)

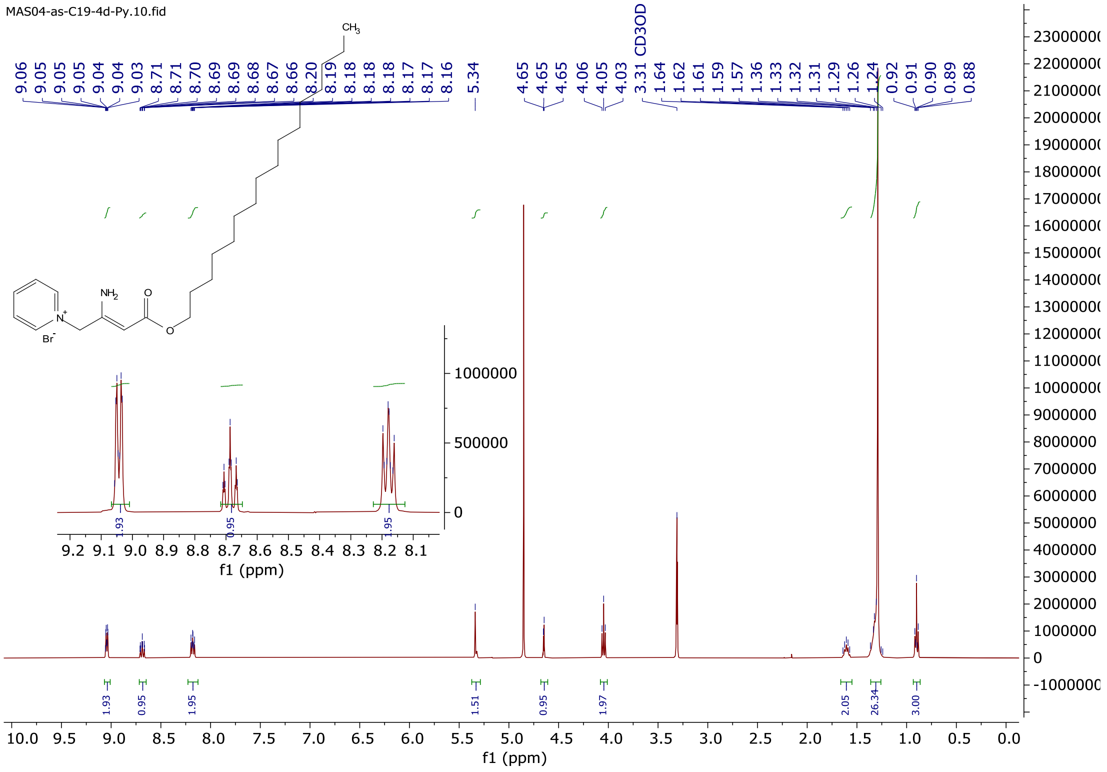

Figure S112: ^{1}H-NMR spectrum of (*Z*)-1-(2-amino-4-(hexadecyloxy)-4-oxobut-2-en-1-yl)pyridin-1-ium bromide (**9c**)

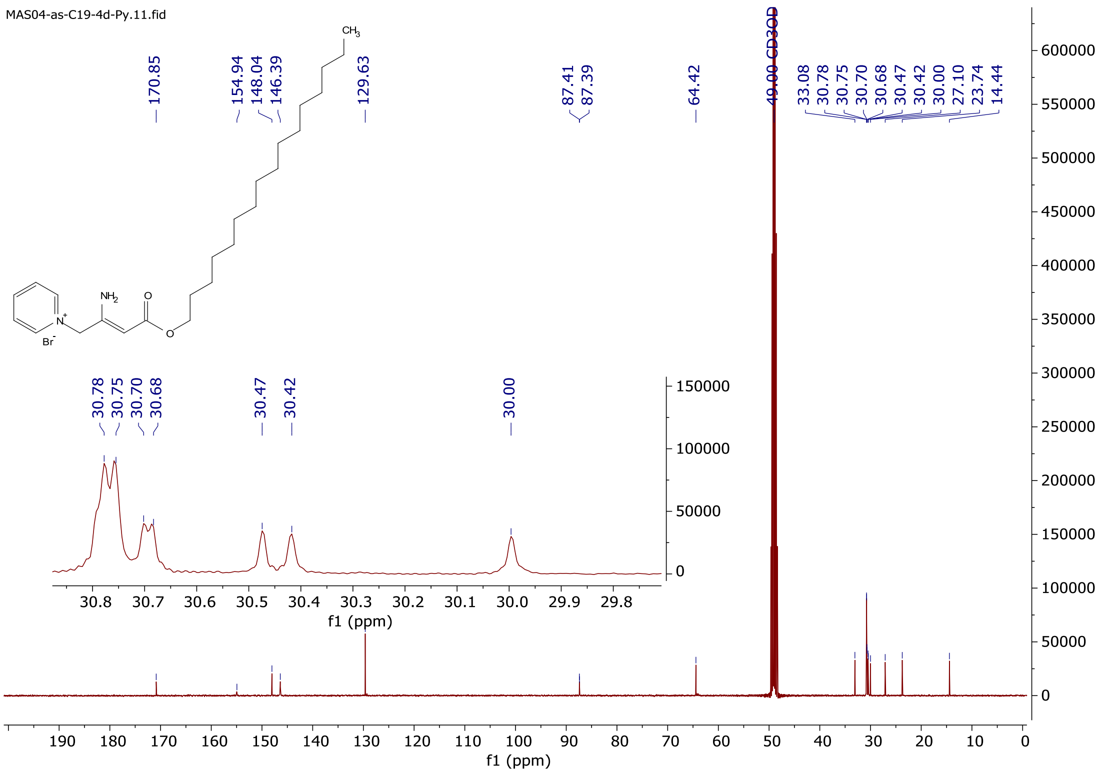

Figure S113: ^{13}C-NMR spectrum of (*Z*)-1-(2-amino-4-(hexadecyloxy)-4-oxobut-2-en-1-yl)pyridin-1-ium bromide (**9c**)

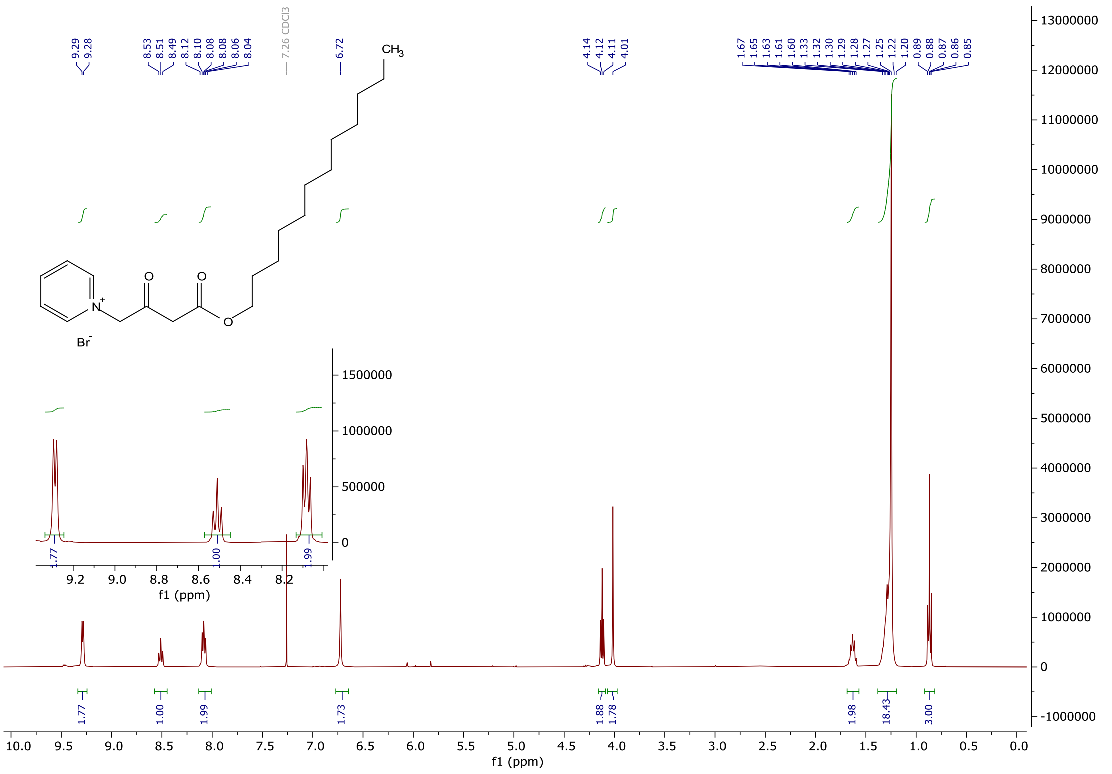

Figure S114: ^{1}H-NMR spectrum of 1-(4-(dodecyloxy)-2,4-dioxobutyl)pyridin-1-ium bromide (**10a**)

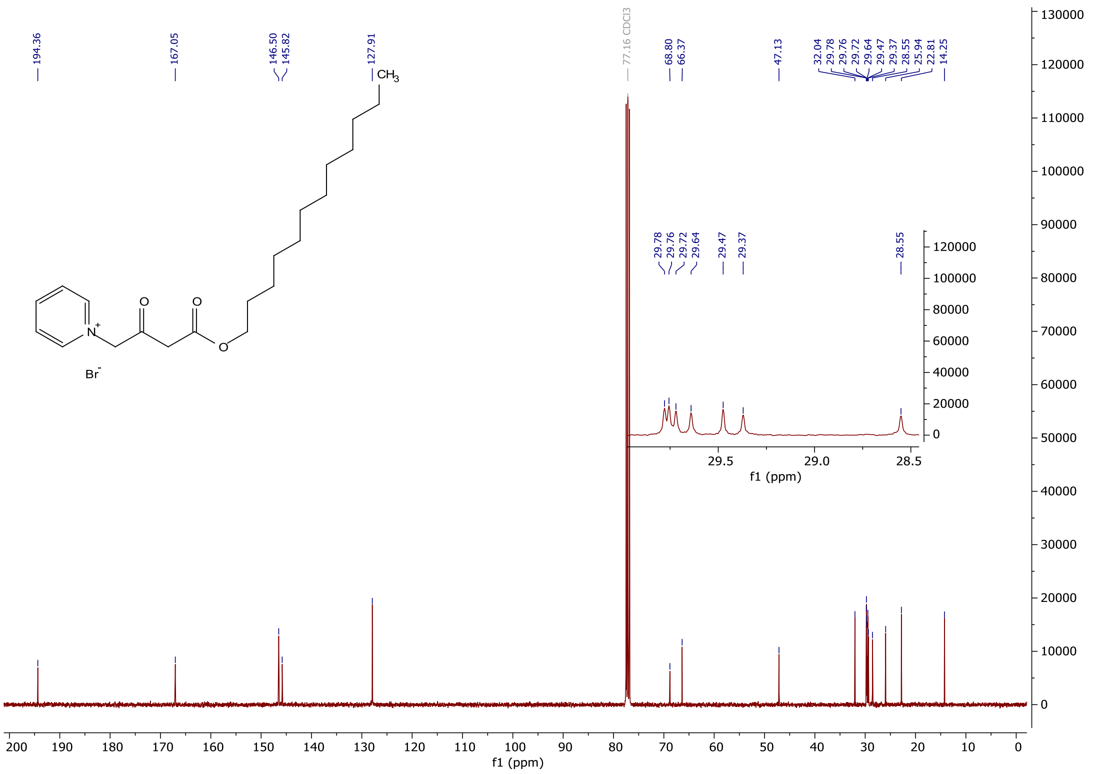

Figure S115: ^{13}C-NMR spectrum of 1-(4-(dodecyloxy)-2,4-dioxobutyl)pyridin-1-ium bromide (**10a**)

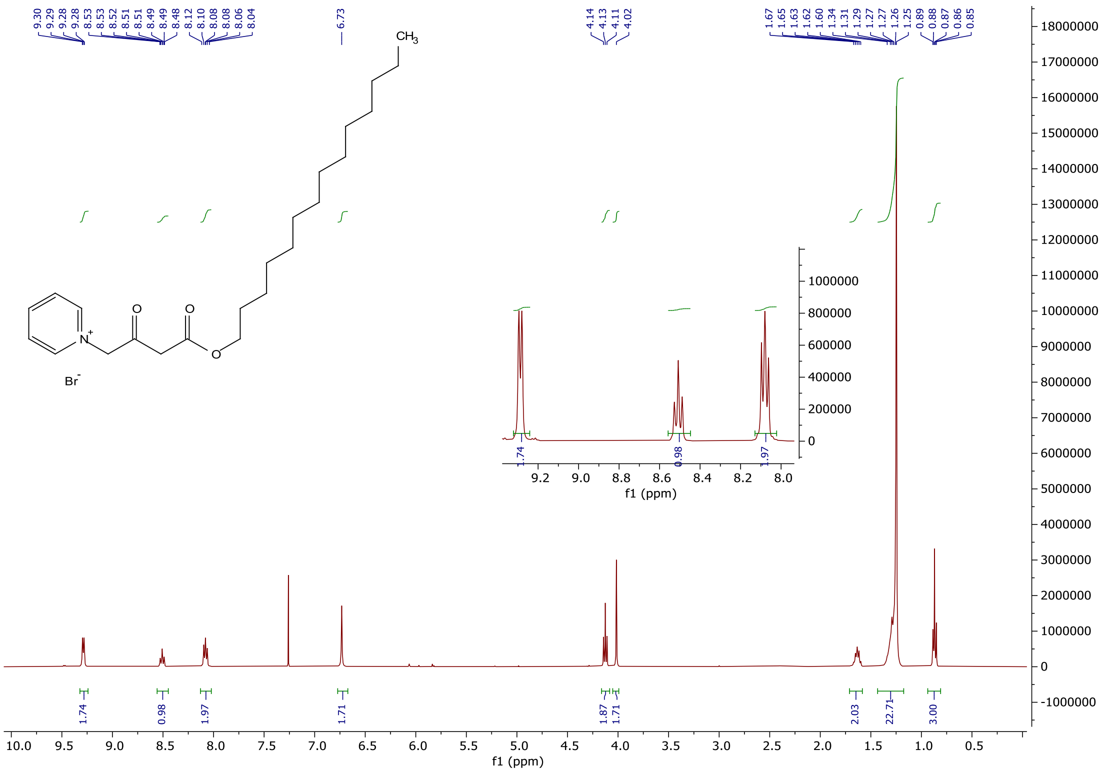

Figure S116: ^{1}H-NMR spectrum of 1-(2,4-dioxo-4-(tetradecyloxy)butyl)pyridin-1-ium bromide (**10b**)

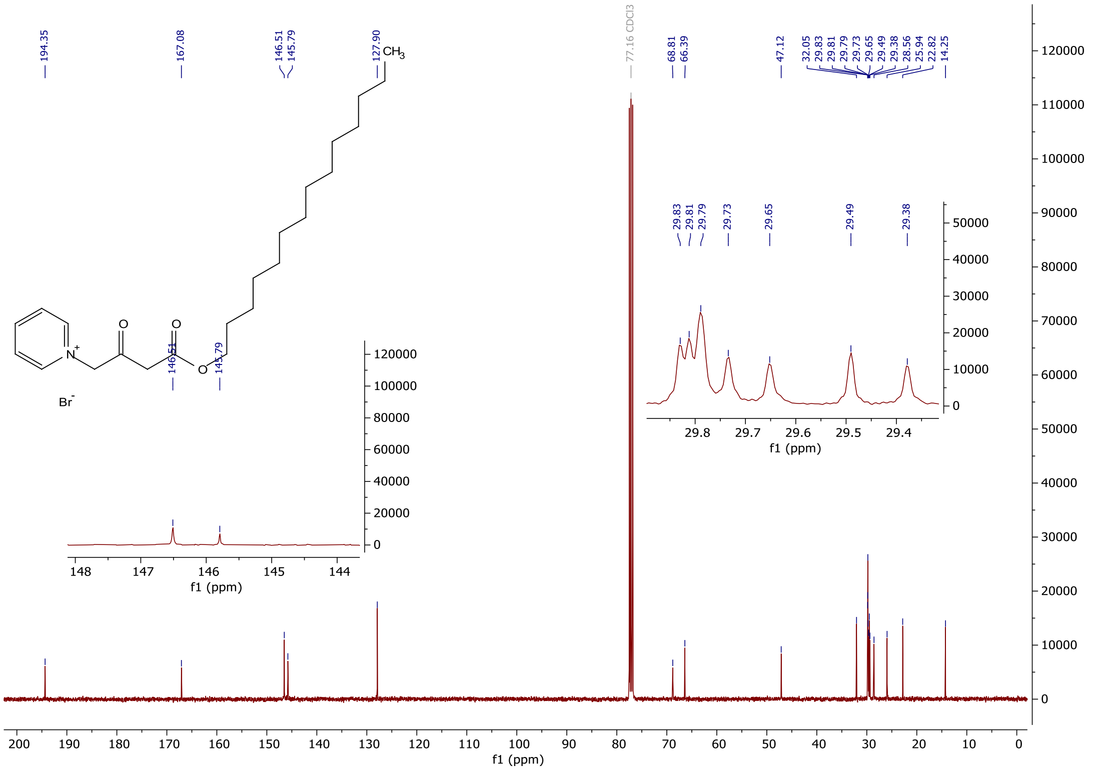

Figure S117: ^{13}C-NMR spectrum of 1-(2,4-dioxo-4-(tetradecyloxy)butyl)pyridin-1-ium bromide (**10b**)

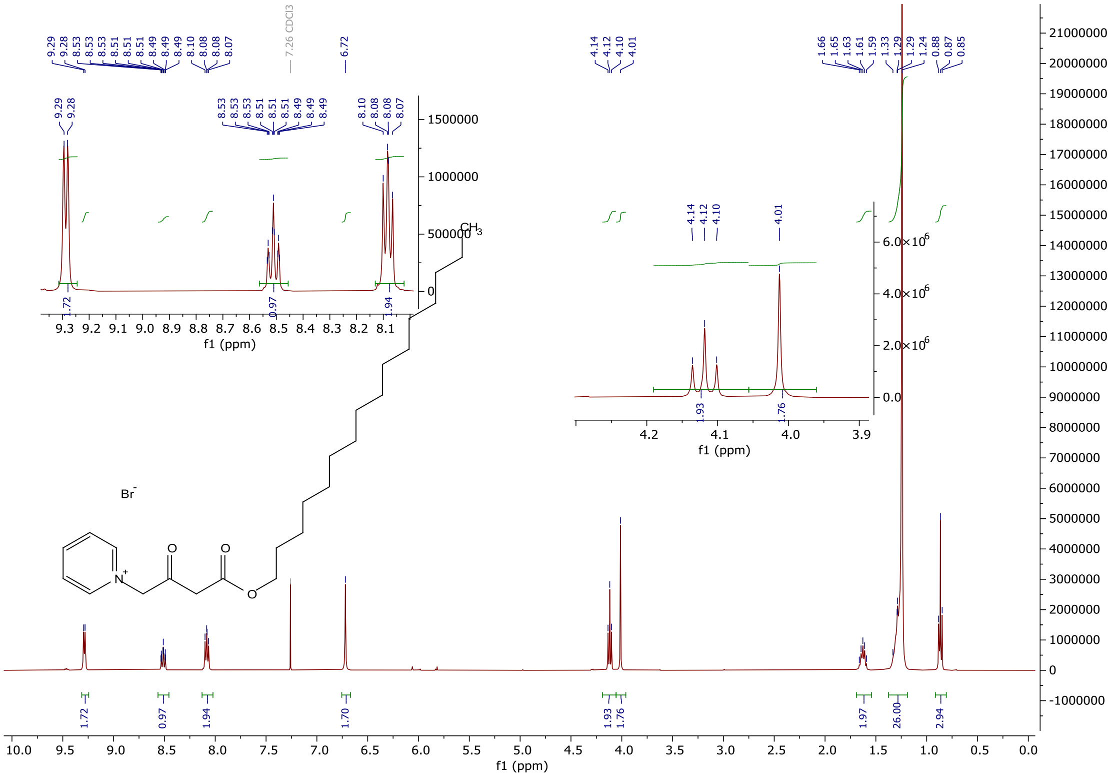

Figure S118: ^{1}H-NMR spectrum of 1-(4-(hexadecyloxy)-2,4-dioxobutyl)pyridin-1-ium bromide (**10c**):

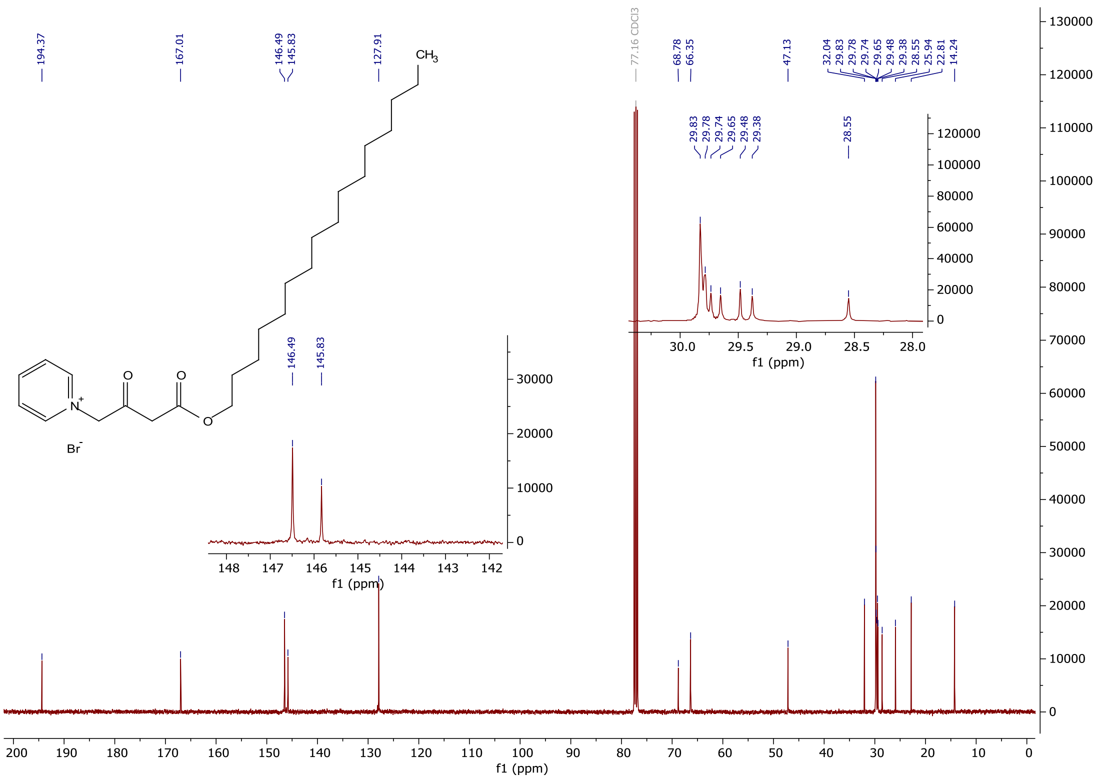

Figure S119: ^{13}C-NMR spectrum of 1-(4-(hexadecyloxy)-2,4-dioxobutyl)pyridin-1-ium bromide (**10c**):

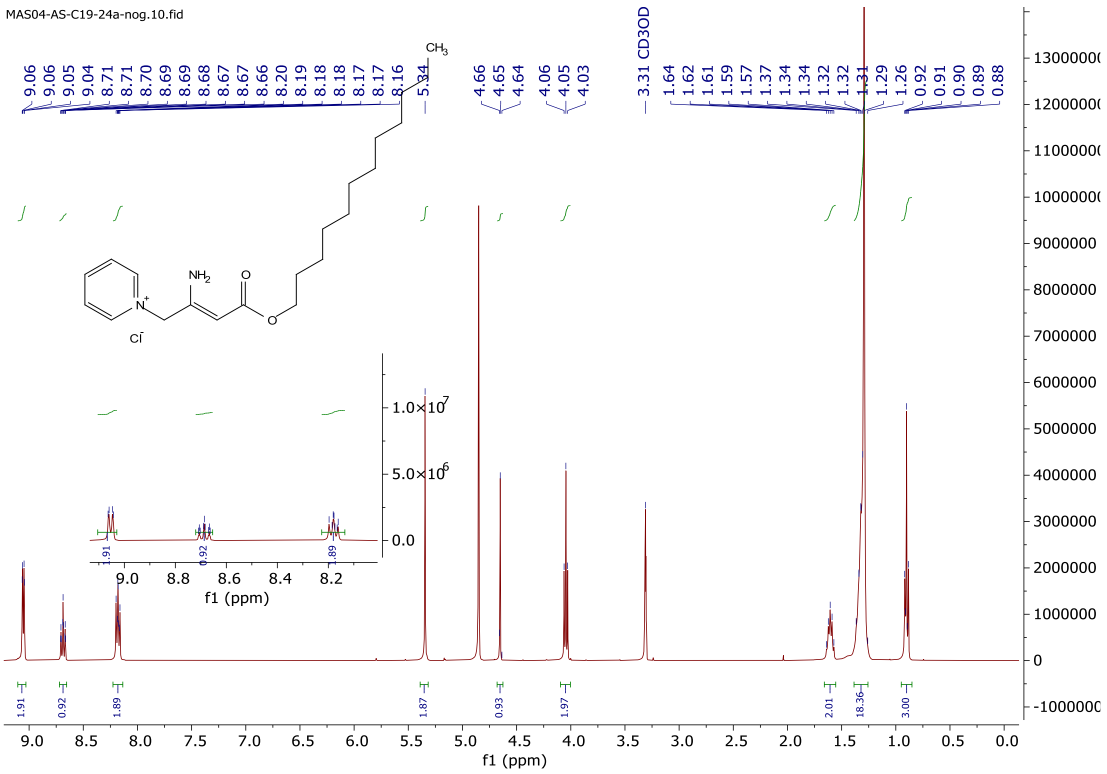

Figure S120: ^{1}H-NMR spectrum of (*Z*)-1-(2-amino-4-(dodecyloxy)-4-oxobut-2-en-1-yl)pyridin-1-ium chloride (**8a**)

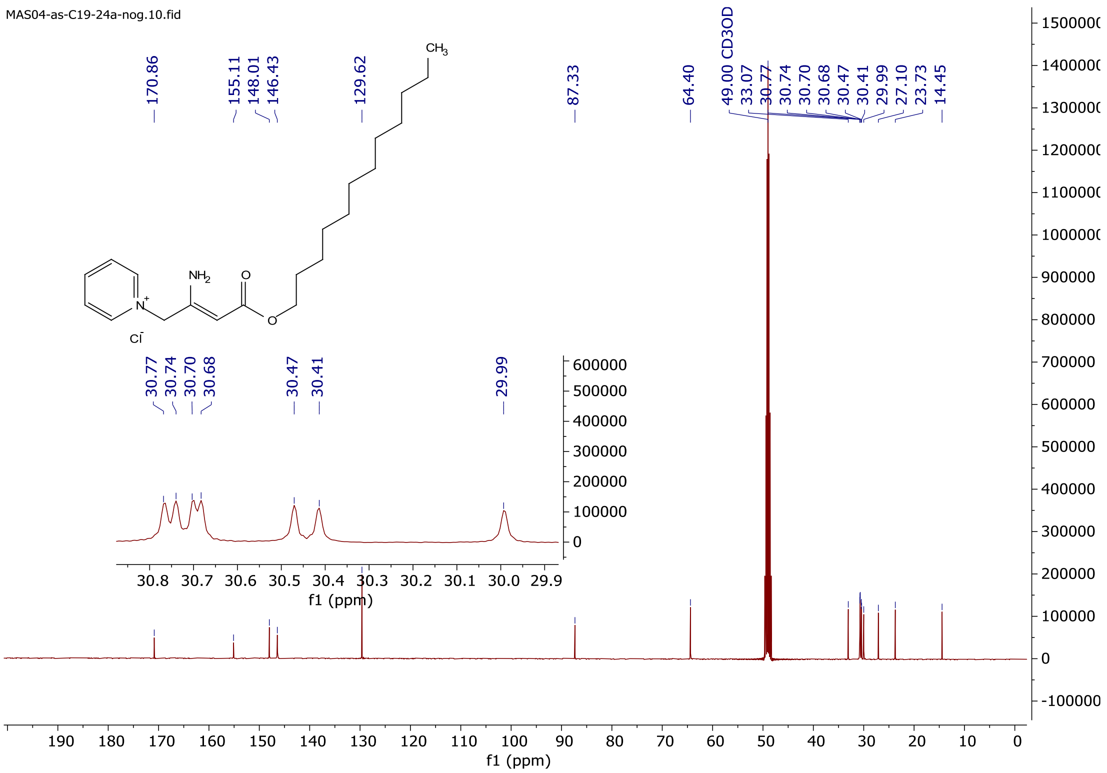

Figure S121: ^{13}C-NMR spectrum of (*Z*)-1-(2-amino-4-(dodecyloxy)-4-oxobut-2-en-1-yl)pyridin-1-ium chloride (**8a**)

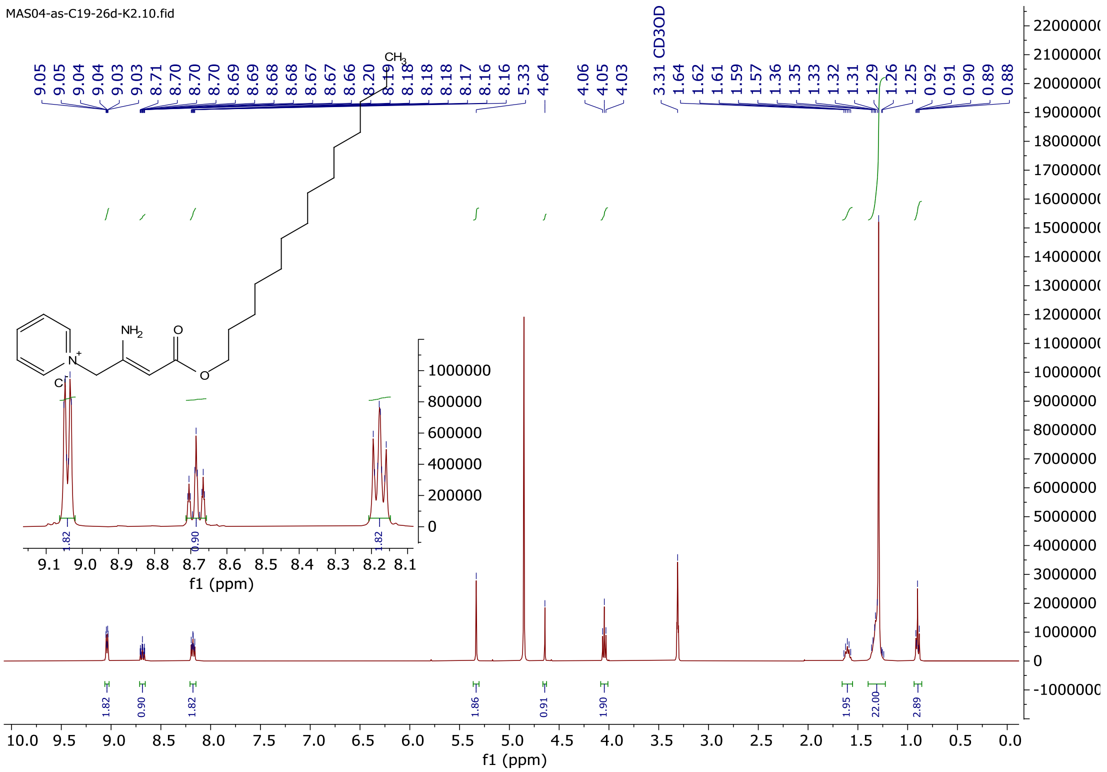

Figure S122: ^{1}H-NMR spectrum of (*Z*)-1-(2-amino-4-oxo-4-(tetradecyloxy)but-2-en-1-yl)pyridin-1-ium chloride (**8b**)

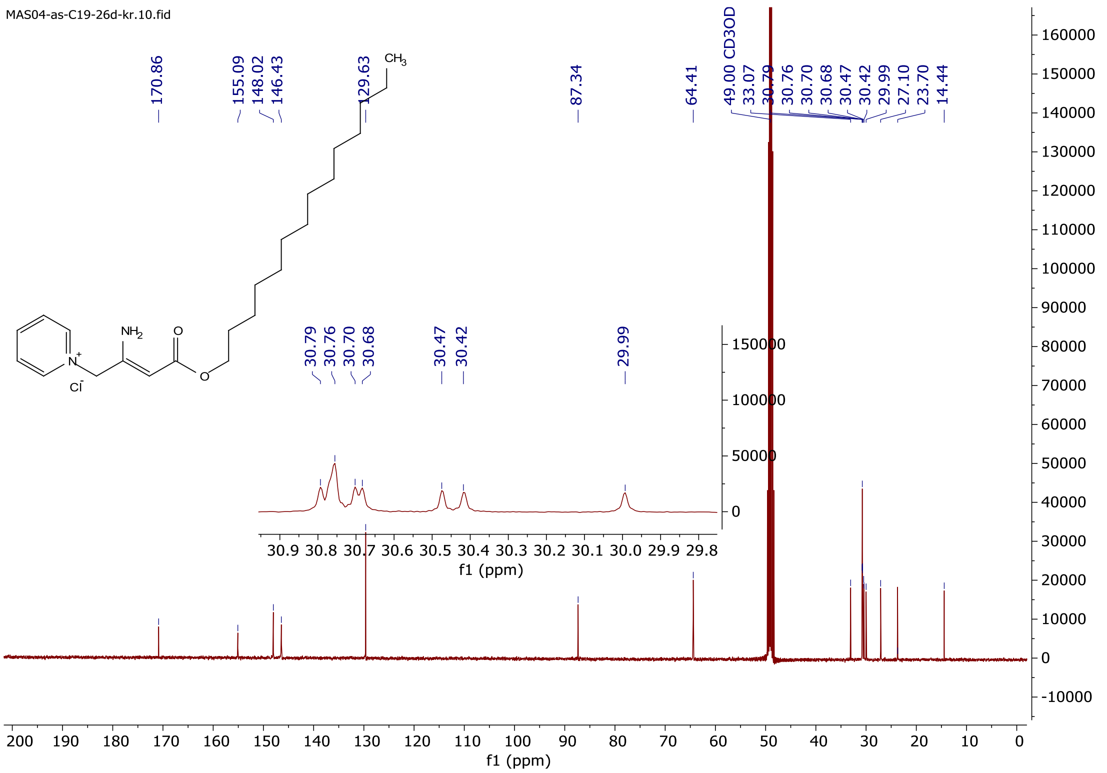

Figure S123: ^{13}C-NMR spectrum of (*Z*)-1-(2-amino-4-oxo-4-(tetradecyloxy)but-2-en-1-yl)pyridin-1-ium chloride (**8b**)

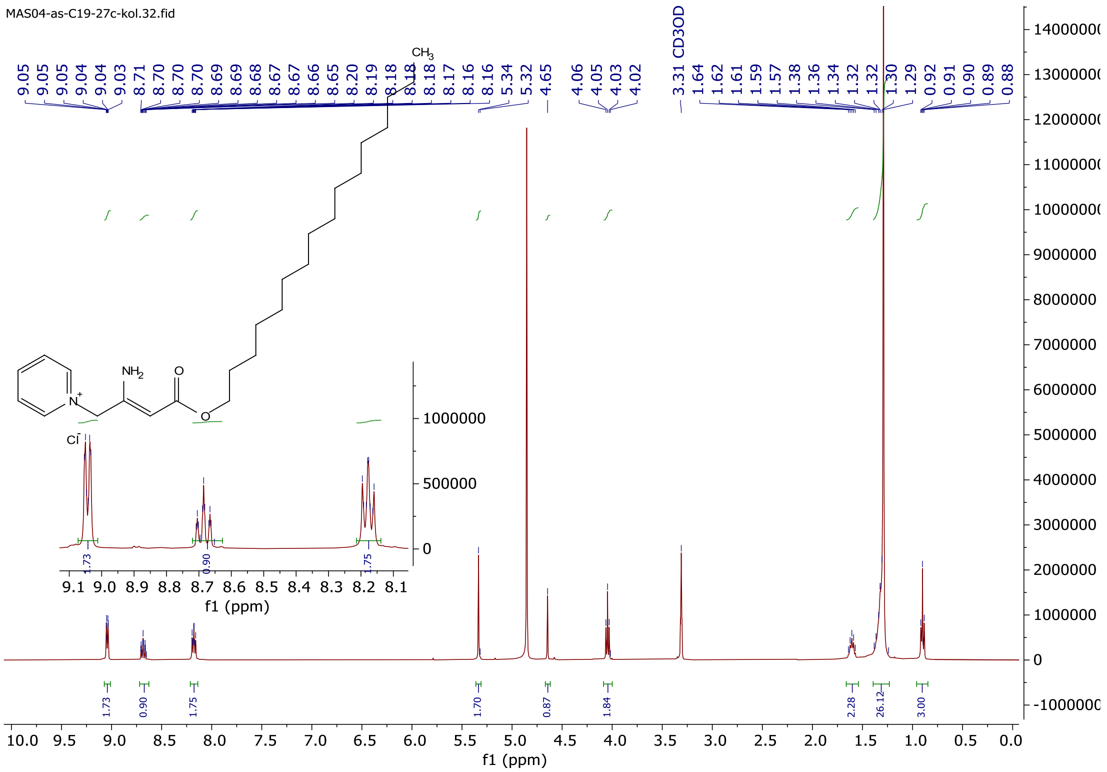

Figure S124: ^{1}H-NMR spectrum of (*Z*)-1-(2-amino-4-(hexadecyloxy)-4-oxobut-2-en-1-yl)pyridin-1-ium chloride (**8c**)

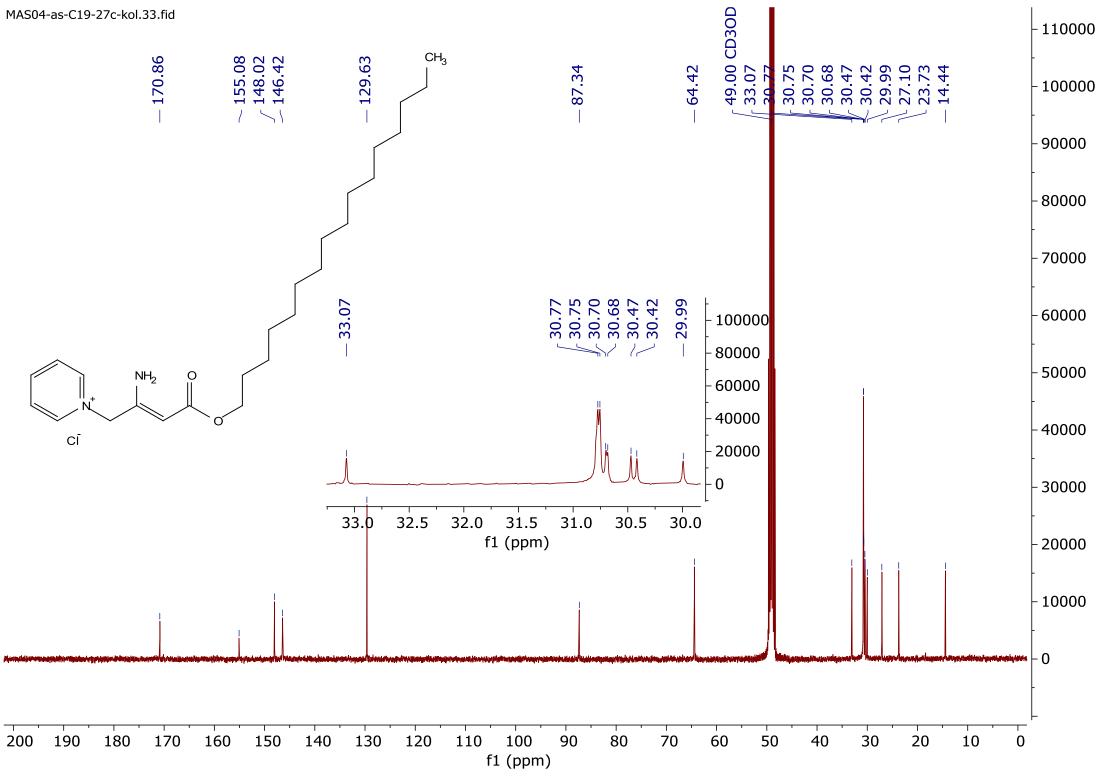

Figure S125: ^{13}C-NMR spectrum of (*Z*)-1-(2-amino-4-(hexadecyloxy)-4-oxobut-2-en-1-yl)pyridin-1-ium chloride (**8c**)

References

36. Amanullah, S.; Das, P.K.; Samanta, S.; Dey, A. Tuning the thermodynamic onset potential of electrocatalytic O2 reduction reaction by synthetic iron-porphyrin complexes. *Chem. Commun.* **2015**, *51*, 10010–10013. https://doi.org/10.1039/c5cc01938a.
